# Why Selection Might Be Stronger When Populations Are Small: Intron Size and Density Predict within and between-Species Usage of Exonic Splice Associated *cis-*Motifs

**DOI:** 10.1093/molbev/msv069

**Published:** 2015-03-13

**Authors:** XianMing Wu, Laurence D. Hurst

**Affiliations:** ^1^Department of Biology and Biochemistry, University of Bath, Bath, Somerset, United Kingdom

**Keywords:** synonymous mutation, exonic splice enhancer, purifying selection, intron density

## Abstract

The nearly neutral theory predicts that small effective population size provides the conditions for weakened selection. This is postulated to explain why our genome is more “bloated” than that of, for example, yeast, ours having large introns and large intergene spacer. If a bloated genome is also an error prone genome might it, however, be the case that selection for error-mitigating properties is stronger in our genome? We examine this notion using splicing as an exemplar, not least because large introns can predispose to noisy splicing. We thus ask whether, owing to genomic decay, selection for splice error-control mechanisms is stronger, not weaker, in species with large introns and small populations. In humans much information defining splice sites is in *cis-*exonic motifs, most notably exonic splice enhancers (ESEs). These act as splice-error control elements. Here then we ask whether within and between-species intron size is a predictor of the commonality of exonic *cis-*splicing motifs. We show that, as predicted, the proportion of synonymous sites that are ESE-associated and under selection in humans is weakly positively correlated with the size of the flanking intron. In a phylogenetically controlled framework, we observe, also as expected, that mean intron size is both predicted by *N*_e_*.μ* and is a good predictor of *cis-*motif usage across species, this usage coevolving with splice site definition. Unexpectedly, however, across taxa intron density is a better predictor of *cis*-motif usage than intron size. We propose that selection for splice-related motifs is driven by a need to avoid decoy splice sites that will be more common in genes with many and large introns. That intron number and density predict ESE usage within human genes is consistent with this, as is the finding of intragenic heterogeneity in ESE density. As intronic content and splice site usage across species is also well predicted by *N*_e_*.μ*, the result also suggests an unusual circumstance in which selection (for *cis-*modifiers of splicing) might be stronger when population sizes are smaller, as here splicing is noisier, resulting in a greater need to control error-prone splicing.

## Introduction

Classical nearly neutral theory proposes that selection will be less efficient as the effective population size (*N*_e_) goes down (Ohta 1973, 1992, 1996). In this context, we can, for example, interpret the finding that humans have a more “bloated” genome than seen in a species such as yeast which has a large effective population size and a correspondingly “lithe” genome (Lynch and Conery 2003). A lithe genome is one with short intergene spacer, relatively little repetitive sequence, few introns with the few found being relatively small. Might it, however, be the case that, as genomes decay owing to reduced *N*_e_, the error rates of critical processes go up (cf. Frank 2007)? This might include increased mistranscription, mistranslation, missplicing, incorrect protein folding, incorrect phosphorylation, incorrect subcellular localization, etc. (Lynch 2007). Might this in turn then result in otherwise paradoxical stronger selection on error mitigation phenotypes when populations are small? Were this so, this would add a novel dimension to the nearly neutral hypothesis, as it would suggest that selection can sometimes be stronger when effective populations sizes are small, because, in this instance, the error rates are higher.

In the article, we examine this possibility by considering splicing error as an exemplar. In particular, we assume 1) that intron sizes tend to increase as *N*_e_ declines and that this is largely attributable to genome bloating (Lynch and Conery 2003) and 2) that within a genome exons flanked by larger introns have noisier splicing. As a consequence, we hypothesize that selection to reduce splice error rates will be more common in species with large introns, typically those with low *N*_e_. Put differently, might humans have gradually expanded their introns through multiple small insertions, each being unable to be resisted by purifying selection, but in the process increased selection on modifiers of splicing in a ratchet-like process (cf. Frank 2007). The selection to reduce splice error rates we suggest will be manifested, in part, as a higher density of exonic *cis*-modifiers of splicing.

The two assumptions of our hypothesis appear to be reasonable, although the first of these has proven controversial. From phylogenetically uncontrolled correlation based analysis Lynch and Conery (2003) noted that across a wide span of species, as *N*_e_*.μ* declines introns tend to get larger and more common (higher density). *N*_e_*.μ* note is the product of effective population size (*N*_e_) and the mutation rate (*μ*), the single statistic being estimated from population heterozygozity data. The trend in intron size Lynch and Conery attribute to weakening selection as *N*_e_ declines, that is, species with low *N*_e_ are less able to eliminate, through purifying selection, weakly deleterious insertion mutations when they occur in introns (and intergenic sequence). This study has, however, been criticized for failing to allow for phylogenic nonindependence between data points (Whitney and Garland 2010). Indeed, it was argued that the key result is not robust to proper phylogenetic control (Whitney and Garland 2010). As this *N*_e_*.μ* intron size/number correlation is a central tenet of the nearly neutral interpretation of genome anatomy, we return to this issue employing a phylogenetically controlled mode of analysis and more up to date estimates of *N*_e_*.μ*, employing both more data and multiple modes of estimation. We show that with these updated estimates, in a phylogenetically controlled framework, *N*_e_*.μ* does indeed predict intron dimensions as Lynch and Conery (2003) postulated. We also show, however, that Whitney and Garland had an important objection, as we do not robustly recover this result using the original Lynch and Conery estimates of *N*_e_*.μ.*

Our second supposition, that larger introns pose a threat to accurate splicing, has received experimental and comparative support. Notably, it is observed that experimental insertion of sequence into introns can reduce splice rates (Klinz and Gallwitz 1985; Luehrsen and Walbot 1992; Fox-Walsh et al. 2005; Sironen et al. 2006) and the exons hardest to splice consistently are those flanked by large introns (Bell et al. 1998; Fox-Walsh et al. 2005). Exons flanked by short introns, also associated with high expression levels, tend by contrast to be subject to less noisy splicing (Pickrell et al. 2010). In the longer term, exons flanked by long introns tend to be those most commonly lost (Kandul and Noor 2009), consistent with splice error rates being too high to sustain the exon. Exactly why exons flanked by larger introns are harder to splice is not fully understood, but one can speculate that if an intron is large, the splice site is harder to locate and the possibility for cryptic splice sites contained within the intron would be higher. The true splice sites need the reinforcement afforded by serine/arginine-rich (SR) proteins binding to exonic splice enhancers (ESEs).

Our hypothesis that selection to reduce splice error rates will be manifested in part as a higher density of exonic *cis*-modifiers of splicing is, in part, predicated upon the knowledge that *cis*-modifiers of splicing are known to be important in humans. For our genes, only approximately 50% of the information defining splice sites is at the splice site, the rest being in *cis-*motifs (Lim and Burge 2001). Possibly, the most importance of these motifs are ESEs (Blencowe 2000). The importance of ESEs is well demonstrated by the influence they have on selection on synonymous mutations (Carlini and Genut 2006; Parmley et al. 2006; Cáceres and Hurst 2013). Recent estimates suggest that around 4–5% of synonymous mutations in humans are under purifying selection because they disrupt ESEs (Cáceres and Hurst 2013). Our hypothesis might also predict that this figure might be a little lower in mice than in humans, as humans have on average larger introns. This has yet to be established, but suggestively, while standard nearly neutral *N*_e_-based arguments would more obviously have predicted that selection on synonymous sites should be less common in humans than in rodents (Sharp et al. 1995; Keightley et al. 2005), the reverse seems to be true: An estimated 20% of synonymous mutations under selection in humans but only 10% in mice (Eory et al. 2010).

As prima facie support for the notion that selection for splice-error proofing can be more intense when populations are small, we note that the inferred centrality of ESEs to splicing in humans contrasts with species, such as yeast, with few/small introns and large populations. *Saccharomyces cerevisiae*, for example, appears not to employ ESEs to reinforce splicing (Spingola et al. 1999; Warnecke et al. 2008). More generally, the modes of selection on synonymous mutations in yeast and mammals appear to be rather different. Although in yeast there is easily identified translational selection (whereby codon usage evolves in accord with the tRNA pool), most acute in highly expressed genes (Ikemura 1982, 1985; Kanaya et al. 2001), the same is not robustly found in mammals (Bernardi et al. 1985; Sharp et al. 1995; Kanaya et al. 2001; Duret 2002). Rather, in mammals, selection on synonymous mutations is predominantly at exonic ends where ESEs aggregate (Carlini and Genut 2006; Parmley et al. 2006, 2007; Cáceres and Hurst 2013). In addition, however, there is evidence for selection on synonymous mutations in mammals mediated by miRNA pairing (Hurst 2006; Brest et al. 2011; Gartner et al. 2013), cotranslational folding (Lawrie et al. 2013), and mRNA structure modulation (Chamary and Hurst 2005; Nackley et al. 2006; Bartoszewski et al. 2010).

Our hypothesis makes a series of intra- and interspecific predictions. We expect, for example, that within a genome selection on ESEs might be more common in exons neighboring larger introns. Prior evidence supports the possibility that intron size is an important predictor of ESE density, at least within the human genome, ESEs being at a higher density at exon ends in proximity to longer introns (Dewey et al. 2006; Cáceres and Hurst 2013). It is not, however, known whether the higher density also implies more ESEs under selection. More generally, it is not known whether all putative ESE sites are functional. The apparent excess near long introns may, for example, reflect simple biased nucleotide content covarying with intron size (Duret et al. 1995). Here then we first ask whether selection on ESE-related synonymous sites might be more common in the vicinity of large introns, controlling for nucleotide usage. To this end we estimate the absolute number of ESE-related synonymous sites in proximity to an exon–intron junction that are under selection, as a function of the size of the flanking intron.

Our hypothesis also predicts that ESE usage should vary greatly between species, being greater when populations are small and introns large. Prior evidence suggests that there is indeed considerable between-species variation in exonic *cis*-motif usage. Although ESEs are only well described in a handful of species, trends in *k*-mer usage across species in the vicinity of exon ends can be employed as a surrogate measure (Warnecke et al. 2008; Wu et al. 2013). Many *k*-mers are either enriched or depleted in the vicinity of exon junctions, trends in amino acid and codon usage in the vicinity of exon ends being a case in point. These trends are typically well predicted by underlying nucleotide content of the *k*-mers and the extent to which such nucleotides are employed in ESEs (Parmley and Hurst 2007; Cáceres and Hurst 2013), these being commonly purine-rich (Cáceres and Hurst 2013). Indeed, even in a species as distant from humans as *Ectocarpus* (a brown algae), 6-mer trends accord well with known human-described ESEs (Wu et al. 2013). Moreover, species lacking such distortion in *k*-mer usage also tend to be those that do not employ SR proteins to aid splicing, SR proteins being the binding partners of ESEs (Warnecke et al. 2008; Wu et al. 2013). Conversely, trends in *k*-mer usage in the vicinity of exon ends have been employed to define novel splice-related exonic motifs (Lim et al. 2011).

Taking the degree of distortion on *k*-mer usage in the vicinity of exon ends as a metric of the extent of *cis*-motif usage for splice control, prior studies report considerable variation between taxa in the number of *k*-mers affected (Warnecke et al. 2008; Wu et al. 2013). Here then, we ask whether we can account for this variation in terms of between-species variation in the size of introns and the effective population size. For compatibility with prior studies we employ in frame 3-mers, that is, codons. Prior evidence suggests that *cis-*motif usage, measured this way, may be most prevalent in species with more intronic sequence (Warnecke et al. 2008), but whether it is intron size or number that matters is not clear. Also suggestive of a relationship between ESE usage and intron dimensions, we recently showed that *Ectocarpus*, a species very distant from mammals and unusual in also having large introns, has extensive *cis-*motif usage, these motifs corresponding to ESEs well described in humans (Wu et al. 2013).

These prior analyses have, however, been confounded by a difference between species in the number of exons sampled and by not controlling for phylogeny. They also do not distinguish between intron density and intron size as predictors, whereas our model relates to intron size. We here ask in a phylogenetically explicit framework 1) whether mean intron size is a predictor of a species usage of *cis-*motifs and 2) whether it is a stronger predictor than intron density (the number of introns per bp of coding sequences [CDS]). To date, we are unaware of experimental evidence suggesting that intron density should predict ESE usage. This being so, if the selection across taxa for ESEs is mediated by changes in intron size alone, then intron density should not be a good predictor. In addition we employ a compound predictor, this being the ratio of CDS size to gene size that factors both intron density and mean intron size. If only intron size is relevant, then this compound predictor should be no better a predictor than mean intron size. Finally, we can ask how such trends in *cis-*motif usage correlate with *N*_e_ or rather *N*_e_*.μ*, this metric estimated from intraspecific polymorphism levels. Given prior evidence that ESE usage and the nucleotides defining the splice site coevolve (Fairbrother et al. 2002; Dewey et al. 2006), we also address splice site usage as a function of *N*_e_*.μ* and intron size.

## Results

### Selection on Synonymous Mutations Is More Common When the Flanking Intron Is Large

The question as to whether selection on *cis-*splice motifs is more commonplace when the flanking intron is larger has two components: First, to what extent are such motifs under purifying selection as a function of the size of the flanking intron and second, how common are such motifs as a function of the size of the flanking intron.

#### Human ESEs Are Slower Evolving When the Flanking Intron Is Larger, but This Is Likely to Be Mutational

Are ESEs slower evolving than non-ESE sequence toward exon ends and are both the rate of evolution and the degree of constraint modulated by the size of the flanking intron? To address this, we consider human–macaque aligned sequence and classified exon ends in terms of the size of the flanking intron. As the exon ends are so small, to minimize estimation noise we consider for each intron size range the concatenation of all exon end alignments so as to provide a single estimate of Ks for each intron size class. We compare the synonymous rate of evolution in and out of ESE sequence.

To consider whether a hexamer might be an ESE motif, we took advantage of a recent analysis which derived two sets of motifs that were agreed on by the majority of ESE discovery analyses as being ESEs (Cáceres and Hurst 2013) and hence provide gold standard data sets with low false positive rates. As these are data sets that are intersects of independent data sets, in which at least three of four putative ESE data sets agree that a given hexamer motif is an ESE motif, we follow that prior nomenclature and refer to these as INT3 and INT3_400. Of the four original input data sets, one (Ke et al. 2011) presented a liberally defined set of ESEs and a more conservative top 400 set. As these two input sets are nonindependent, the prior authors (Cáceres and Hurst 2013) built two intersect data sets: One where the liberal set was employed and one in which the more conservative 400 strong data set was employed. The two resulting three-way intersect sets were thus termed INT3 (84 hexameric motifs with the liberal set employed) and INT3_400 (54 hexameric motifs with the top 400 hexamers employed), respectively.

**Fig. 1.**
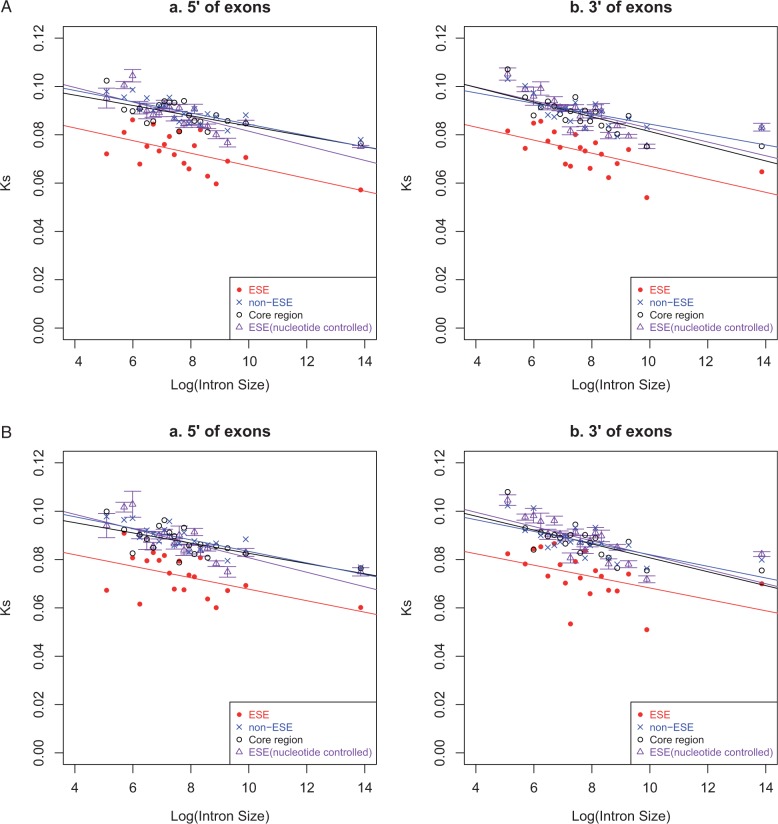
Rate of synonymous evolution in ESE and non-ESE sequences at exon ends as a function of the Log of flanking intron size for two ESE data sets (*A*: INT3, *B*: INT3_400). In addition to Ks of ESE and non-ESE we also show Ks of exon core domains and pseudo-ESE, that is, hexamers of the same underlying nucleotide content as ESEs but not necessarily identified as being functional ESE. We consider 20 intron size bins apportioned so that all bins contain the same number of exon ends for concatenation, the numbers given reflecting the upper intron size limit of each bin.

As can be seen (fig. 1*A* and *B*) the ESE sequence evolves slower at synonymous sites than does non-ESE, as previously shown (Parmley et al. 2006; Cáceres and Hurst 2013). The difference between ESE and non-ESE may be a consequence of differences in mutation rate owing to skewed nucleotide content of ESEs. To examine this, we simulated sets of randomized pseudo-ESE sets that are the same size and drawn from the same underlying nucleotide content as the true ESE sets. We then match these pseudoESEs against the sequence alignments to determine the rate of evolution associated with these. These sets evolve faster than the true ESEs suggesting that ESEs are indeed (as commonly reported) under purifying selection (fig. 1), even allowing for biased nucleotide content.

More striking, we observe an evident negative correlation between Ks of ESEs at exon ends and the size of the flanking intron (INT3_400: 5′ rho = −0.50, *P* = 0.027; 3′ rho = −0.64, *P* = 0.003; INT3: 5′ rho = −0.53, *P* = 0.018; 3′ rho = −0.74, *P* = 3 × 10^−^^4^). This is consistent with stronger purifying selection on *cis-*splicing motifs or mutation rate differences covarying with intron size. That we see a commensurate decrease in Ks of the “non-ESE” sequence as a function of intron size might reflect either 1) purifying selection in exon ends is generally stronger in the vicinity of large introns, possibly because the definition of non-ESE is too liberal and includes much sequence that is functional splice related motif or 2) the mutation rate in exons is lower in the vicinity of larger introns. To examine the latter possibility we compare Ks of exon cores as a function of the size of neighboring introns (we consider the size of the 5′ and 3′ intron separately), under the presumption that little or no sequence in exon cores will modulate splicing. We observe that Ks of cores also show a decreasing tendency as intron sizes increases (fig. 1). Although we can conclude that the reduced rate of evolution of ESEs, compared with non-ESE and pseudo-ESE in the same exons, is not solely mutational in origin, we cannot then exclude the possibility that the low rate of synonymous evolution at exon ends in the vicinity of large intron is at least in part owing to genomically regional mutation rate biases in the vicinity of large introns.

Consideration of the rate of synonymous evolution of exon cores also permits us to define the approximate degree of constraint operating on ESE at exon ends as:

$$\text{Flank ESE constraint}=\frac{\left[\text{Ks core}-\text{Ks ESE flank}\right]}{\text{Ks core}}.$$

**Fig. 2.**
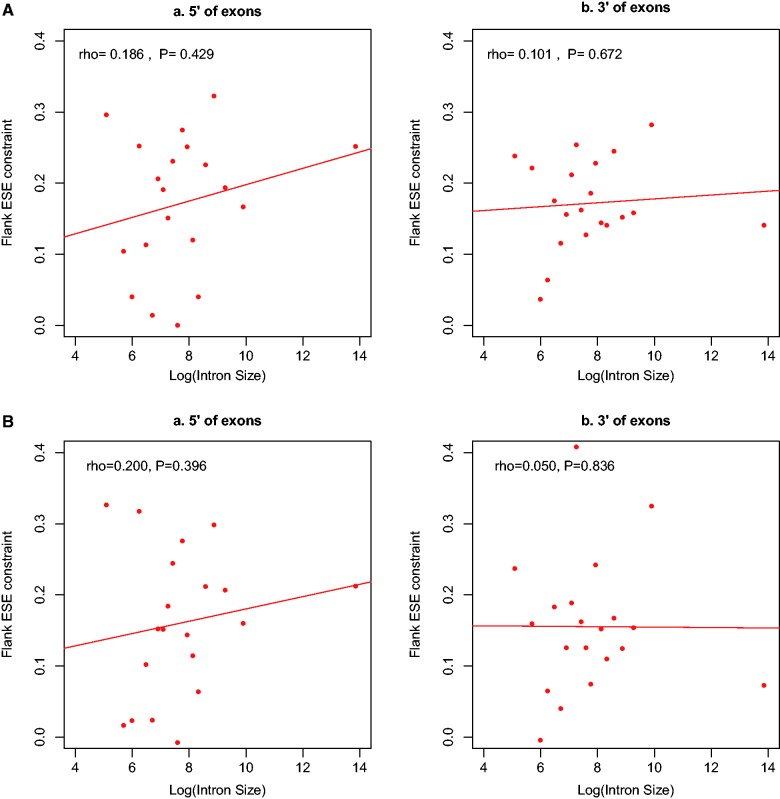
The degree of selective constraint on ESE sequences at exon ends as a function of the Log of flanking intron size for two ESE data sets (*A*: INT3, *B*: INT3_400). For definition of constraint, see main text. For intron size definition, see figure 1. Note that in all cases constraint appears stronger when intron sizes are larger, although using 20 bins the trends are not significant.

This may be conservative, but it is noteworthy that Ks non-ESE flank, Ks pseudoESE, and Ks core are all approximately of the same magnitude (fig. 1*A* and *B*), much higher than Ks ESE flank. In the absence of purifying selection on ESE at exon flanks, in excess of that at exonic cores, the degree of constraint should be zero. We observe that the level of constraint, thus defined, operating on ESEs at exon flanks is not significantly related to the size of the flanking intron, although Spearman’s rho is positive in all incidences (fig. 2*A* and *B*; supplementary table S1.1). What can reasonably be concluded is that selection on ESEs is not obviously weaker in the vicinity of large introns. To estimate the number of sites under selection at exon flanks, we need in addition to factor in not just the level of constraint but also ESE density. This we consider next.

#### Allowing for Increased ESE Density in Proximity to Large Introns, Selection on Synonymous Sites Associated with ESEs Is (slightly) More Common When Introns Are Larger

It has previously been reported that ESE density tends to be a little higher in the vicinity of longer introns (Dewey et al. 2006; Cáceres and Hurst 2013). We replicate this by partial correlation analysis between ESE density and three intronic dimensions (supplementary table S2.1). For ESE data set INT3, both 5′ and 3′ show significant correlation between ESE density and mean intron size (5′ rho = 0.03, *P* = 9 × 10^−^^4^; 3′ rho = 0.03, *P* = 9 × 10^−^^4^). However for the smaller INT3_400 ESE data set, 3′ correlation is not significant (INT3_400: 5′ rho = 0.06, *P* = 2 × 10^−^^13^; 3′ rho = −0.01, *P* = 0.14). More marginal results at exonic 3′-ends is a common theme in our analyses which we comment on later.

To evaluate the net effect of flanking intron size (constraint and increased density), we calculate the proportion of synonymous sites under ESE-related constraint at exon flanks as flank ESE constraint × ESE density. It is no surprise that the net effect of flanking intron size on proportion of sites under selection is an increasing function, albeit only weakly so, as both underlying trends are positive. However, using the conservative binning method (*N* = 20) the trend is not significant. This may well reflect a limited sample size (*N* = 20). To avoid this problem, we instead calculate the regression line of logarithm value of flank intron size versus ESE constraint (using unbinned data). Using this regression line we then estimate the mean ESE constraint for exon flanks given the size of the neighbor intron. For each exon individually, we then calculate ESE density × regression estimated constraint. We find in all cases a positive and highly significant Spearman’s rank correlation (INT3_400: 5′ rho = 0.439, *P* = 0; 3′ rho = 0.123, *P* = 2.7 × 10^−^^31^; INT3: 5′ rho = 0.070, *P* = 2.5 × 10^−^^13^; 3′ rho = 0.646, *P* = 0).

Some of these latter values appear unusually high, which may relate to our interpolation method which smoothes out noise resulting from the tiny number of sites contributing to constraint estimates at individual exon ends. Moreover, this method does not allow for potential covariance with intron number and intron density. To examine this, we analyze gene level (rather than individual exon end) metrics. For each gene, we consider the intron density, intron number, and mean intron size. In addition, we consider the constraint revealed in concatenated exon flanks and concatenated exon cores. A small minority of genes have no synonymous site evolution in exon flank ESEs giving a constraint of unity. As Spearman’s correlation is not necessarily robust to tied values, we thus also replicated analyses using Goodman–Kruskall gamma test with *P* determined by simulation (supplementary table S3.1). To minimize estimation noise, we require for all genes a minimum of 102 bp of concatenated sequence. We then ask whether mean intron size is related to constraint on ESE at flanks.

We find that mean intron size is positively and significantly correlated with 5′ but not 3′ ESE constraint (supplementary table S3.1). This trend is weak (rho 0.048–0.056) but is reported using both statistics and both ESE data sets. Intron density and intron number are not significant predictors. Partial spearman correlation also reports mean intron size as a significant predictor (supplementary table S3.2). To some degree, these results are not robust to increasing the minimum threshold length for analysis from 102 to 150 bp or higher. This, however, appears to be a consequence of reduced sample size. We resampled genes by the number of 150-bp cutoff group from the gene pool of 102-cutoff group and repeated 1,000 times to find how often the intron dimensions can significantly predict the constraints. For mean intron size, only in a little over than 60% of resamplings can we still see significant Spearman partial correlation with 5′-ends ESE constraint for both data sets. For Goodman and Kruskal’s gamma, the commensurate figure is around 42% (supplementary table S3.3). This accords with the trends being weak and hence sensitive to sample size reductions.

We conclude that in the human genome mutations in ESEs at exon ends are probably more commonly under selection when the flanking intron is larger, the effect being mostly mediated by an increased ESE density. This result in turn suggests that disease-associated mutations might be slightly more common in exon ends in the vicinity of large introns, but the effect appears to be modest.

We note that as regards this result we are agnostic as to the cause. This may be a direct effect of intron size or owing to a covariance between intron size and splice site strength, possibly with expression level as a covariate. Our intention here is not to distinguish between these explanations, but simply to suppose that this evidence provides prima facie support to the hypothesis that an increase in intronic dimensions within a species can be coupled with more selection for *cis-*modifiers of splicing. We note, however, that a model that supposes that ESEs are used more in exons next to long introns might be a means to increase elongation rate, to compensate for the time to process the longer intron, is not well supported (see supplementary tables S4.1–S4.2).

### The Ratio of Mature CDS to Gene Size Is the Best Predictor of between-Species *cis*-Motif Usage

Given the above result and prior experimental and comparative data on the difficulty of splicing exons when the neighboring intron is large (see Introduction), we might expect that mean intron size would be a predictor of the commonality of the usage of exon flank *cis-*modifiers of splicing. To establish the latter we consider, for 30 highly phylogenetically dispersed species, the proportion of codons or amino acids that show significant trends in their usage as a function of the distance from an exon–intron junction, these metrics having been shown previously to correspond well with ESE motif usage (Parmley and Hurst 2007; Parmley et al. 2007; Warnecke et al. 2008; Cáceres and Hurst 2013). Using a Bayesian comparative framework, we can then ask whether mean intron size is indeed a good predictor of *cis-*motif usage. We find that it is (table 1). Although our measure of the degree of this trend controlling for the size of exonic input data set is the preferred metric, we show that usage of an uncontrolled metric (employing all valid exons within a species) does not distort the picture (table 1). Hereafter we employ the sample size controlled metric exclusively, unless mentioned otherwise.

**Table 1.** Evidence for Phylogenetically Controlled Correlation between Amino Acid/Codon Usage Trends and the Genomic Traits.

	All Exons (AA)	All Exons (codon)	Random 5,000 Exons (AA)	Random 5,000 Exons (codon)
Log BF (*Y* ∼ *X*)^a^	48.241	39.394	31.923	42.027
Log BF (*Y* ∼ *N*)	37.484	29.202	24.055	32.294
Log BF (*Y* ∼ *M*)	20.145	15.018	12.214	18.410

Note.—We employ two metrics of skews at exon ends, the number of codons showing a significant skew and the number of amino acids showing a significant skew. For each, in addition we report results wherein for each species all relevant exons are employed and a second metric where the input sample size is the same for all species (5,000 randomly chosen exons). In the latter instance, we consider the mean number of significant trends from multiple samplings of 5,000 randomly chosen exons. *Y*, proportion of amino acids/codons showing significant trends; *X*, mean CDS length/gene length; *N*, introns per kb exon; *M*, mean intron size.

^a^Log BF (log Bayes factor) = 2*(log [harmonic mean (complex model)] − log [harmonic mean (simple model)]), is the test statistic of BayesTraits which gives the information of evidence for correlated evolution: Weak evidence (<2), positive evidence (>2), strong evidence (5–10), very strong evidence (>10). All Log BF values in the table are greater than 10, so the evidence from all correlations is very strong.

As a control test, we consider intron density. Intron density, measured as number of introns per kilobase of mature CDS, holds no information regarding the size of the introns and hence if size is the key variable density should be irrelevant. Unexpectedly, not only do we find that density is a predictor of the extent of *cis-*motif usage, we also observe that it is consistently a better predictor than intron size (the BayesTrait score is higher in all modes of analysis; table 1). Given this surprising result we ask whether a metric that considers the net effect of density and size might be an even better predictor. To this end, we employ the ratio of mature CDS to gene size (alias immature transcript size). This is consistently the best predictor (table 1). We conclude that intron size alone is not adequate to describe the between-species trends in *cis-*motif usage and that density effects are also of relevance. The logic of the importance of the density effects we discuss below.

### Evidence for Coevolution of Splice Site and *cis*-Motif Usage

Prior evidence suggests that ESE usage is higher in proximity to certain splice sites (Berget 1995; Graveley 2000). One possibility is that “weak” splice sites might be more in need of the reinforcement offered by flanking ESEs (Fairbrother et al. 2002). In support of this, ESE density appears to be stronger in proximity to “weak” splice sites (Dewey et al. 2006; Plass et al. 2008; Cáceres and Hurst 2013). To ask whether ESE usage across species was predicted by relative usage of different splice sites, we investigated all splice sites across 30 species. The splice sites we represented as four-letter nucleotide strings, nucleotides of exons in upper case, nucleotides of introns in lower case. After phylogenetic correction, BayesTraits provided very strong evidence for correlation between usage of *cis*-motif and usage of two splice sites (“AGgt” and “agGT”) (table 2). This indicates a preference of exonic splice associated *cis*-motifs to these specific splice sites. These results indicate that the trends in *cis*-motif usage across species reflect in part coevolution with splice site usage.

**Table 2.** *Cis*-Motif Usage Correlates Significantly with Usage of “AGgt” and “agGT” Splice Sites.

	All Exons (AA)	All Exons (codon)	Random 5,000 Exons (AA)	Random 5,000 Exons (codon)
Log BF (*Y* ∼ *P*1)	39.0359	31.7091	26.4632	33.7518
Log BF^a^ (*Y* ∼ *P*2)	52.1594	64.0366	76.8355	58.1153

Note.—*Y*, proportion of amino acids/codons showing significant trends; *P*1, proportion of AGgt (Capital letter: exon, small letter: intron); *P*2, proportion of agGT.

^a^Log BF (log Bayes factor) = 2*(log [harmonic mean (complex model)] − log [harmonic mean (simple model)]). All Log BF values in the table are greater than 10, so the evidences of all correlations (positive) are very strong.

### *N*_e_.*μ* Predicts Intronic Dimensions

Given that intronic dimensions predict *cis-*motif usage across taxa, what, we can ask, predicts intronic dimensions across taxa? An attractive proposal is that introns and intronic sequence accumulate owing to weakened selection against insertions associated with reduced *N*_e_. Previously, Lynch and Conery (2003) have argued, in a phylogenetically uncontrolled analysis, that intronic size can be well understood in the context of such a nearly neutral model. They posit that as *N*_e_ reduces selection becomes weaker and the ability of a species to resist weakly deleterious insertions (both new introns and new sequence within extant introns) is in turn reduced. Thus, they predict large introns and high density of introns in species with low *N*_e_.

Their analysis has been criticized on numerous fronts, not least of which is the assumption of *N*_e_*.μ* is a good predictor of the behavior of *N*_e_ alone (Daubin and Moran 2004) (a problem our analysis is also sensitive to). Further, they estimated *N*_e_*.μ* for a sample of species often employing limited sequence data. Perhaps most importantly, their analysis was criticized for failing to control for phylogenetic structure, in effect assuming a star phylogeny (Whitney and Garland 2010). This same follow-up analysis, employing a phylogenetically explicit method failed to observe a relationship between genome size parameters and *N*_e_. We return to this issue employing three methods to estimate *N*_e_*.μ*, three metrics of intronic content, and a fully controlled phylogenetic methodology.

Three *N*_e_*.μ* values of this study show very significant correlations between themselves; however, our estimates of *N*_e_*.μ* do not correlate well with those of Lynch and Conery (table 3, supplementary fig. S1, the blue line indicates the standard major axis [SMA] regression). We find that our *N*_e_*.μ* estimates robustly predict all three intronic dimensions in the expected direction (table 4). By contrast, we can replicate Whitney and Garland’s failure to detect such a correspondence: After phylogenetic correction, although there is a strong evidence to support the correlation between *N*_e_*.μ* values of Lynch and Conery’s study and the ratio of mature CDS to gene size, these *N*_e_*.μ* values do not correlate well with intron density and mean intron size (table 4). We suggest that the paucity of data contributing to the Lynch and Conery estimates of *N*_e_*.μ* is the major issue with their analysis.

**Table 3.** Spearman’s Correlation Analysis Results for *N*_e_*.μ* Values of This Study and the Prior Study of Lynch and Conery.

	rho	rho^2^	*P*
*N*_e_*.μ*_Eta ∼ *N*_e_*.μ*_Lynch	0.093	0.009	0.765
*N*_e_*.μ*_Pi ∼ *N*_e_*.μ*_Lynch	0.165	0.027	0.591
*N*_e_*.μ*_S ∼ *N*_e_*.μ*_Lynch	0.093	0.009	0.765
*N*_e_*.μ*_Eta ∼ *N*_e_*.μ*_S	0.996	0.991	0.000
*N*_e_*.μ*_Pi ∼ *N*_e_*.μ*_Eta	0.970	0.941	0.000
*N*_e_*.μ*_Pi ∼ *N*_e_*.μ*_S	0.975	0.951	0.000

Note.—We compare our three different estimators for *N*_e_*.μ*, (Eta, Pi, and S) and Lynch’s single estimate.

**Table 4.** Evidence for Phylogenetically Controlled Correlation between *N*_e_*.μ* Values and Splice-Related Genomic Traits.

	X	N	M
Log BF (*N*_e_*.μ*_Pi ∼ Splice-related Genomic Traits)^a^	15.762	23.424	41.057
Log BF (*N*_e_*.μ*_S ∼ Splice-related Genomic Traits)	14.572	22.590	39.944
Log BF (*N*_e_*.μ*_Eta ∼ Splice-related Genomic Traits)	13.988	22.695	40.367
Log BF (*N*_e_*.μ*_Lynch^b^ ∼ Splice-related Genomic Traits)	5.290	0.989	−0.587

Note.—We employ our three different estimators for *N*_e_*.μ* (Eta, Pi, and S) and Lynch’s single estimate. *X*, mean CDS length/gene length; *N*, introns per kb exon; *M*, mean intron size.

^a^Log BF (log Bayes factor) = 2*(log [harmonic mean (complex model)] − log [harmonic mean (simple model)]), is the test statistic of BayesTraits which gives the information of evidence for correlated evolution: weak evidence (<2), positive evidence (>2), strong evidence (5–10), very strong evidence (>10). All Log BF values in the table are greater than 10, so the evidences of all correlations are very strong.

^b^This *N*_e_*.μ* value is from previous study (Lynch and Conery 2003).

### *N*_e_.*μ* Predicts Splice Site Usage but Not *cis*-Motif Usage

The above sets of results suggest a simple narrative to explain *cis-*motif usage across species. As *N*_e_ declines, so introns become more abundant and larger, owing to the weakening of purifying selection (result 4 above). A consequence of this is that small insertions may accumulate in a ratchet-like manner. Similarly, splice sites might decay. Both splice site decay and the increase in intron size cause increases in the rate of missplicing compensated by increased usage of exonic *cis*-motifs. Within genomes, the argument goes, this is reflected in a higher density of functional *cis-*motifs in the flanks of exons that neighbor large introns (result 1) and associated with particular splice sites (Cáceres and Hurst 2013). Thus, selection on synonymous mutations at exon flanks is more common when the flanking intron is large (result 1 above) and species with on average larger introns have more *cis*-modifiers (result 2), these being especially common when certain splice sites become more common (result 3). Additionally, consistent with ESE-splice site coevolution, we see intraspecifically that AGgt exons are flanked by larger introns (supplementary table S5.1), consistent with splice site—ESE - intron size three-way coevolution. We would thus expect that *N*_e_*.μ* should also in turn predict the usage of *cis-*splice modifiers and splice sites.

The latter result we find to be robustly supported, at least for 5′-end splice site usage. More specifically, the correlations between *N*_e_*.μ* values and the usage of “AGgt” (i.e., 5′-splice site) are very strong, whereas those about the usage of “agGT” (3′-splice site) are weak (table 5).

**Table 5.** Evidence for Phylogenetically Controlled Correlations between *N*_e_*.μ* Values and Usage of “AGgt” (very strong) and “agGT” (weak) Splice Sites Using Three Estimators of *N*_e_*.μ*, Namely Pi, S, and Eta.

	*N*_e_*.μ*_Pi	*N*_e_*.μ*_S	*N*_e_*.μ*_Eta
Log BF (*N*_e_*.μ*^a^ ∼ *P*1)	22.7225	19.1016	20.6161
Log BF (*N*_e_*.μ* ∼ *P*2)	1.6456	−0.1762	0.6543

Note.—*P*1, proportion of AGgt (Capital letter: exon, small letter: intron); *P*2, proportion of agGT; Log BF (log Bayes factor) = 2*(log [harmonic mean (complex model)] − log [harmonic mean (simple model)]).

^a^Three types of *N*_e_*.μ* (*N*_e_*.μ*_Pi, *N*_e_*.μ*_S, *N*_e_*.μ*_Eta).

Do we also find that *N*_e_*.μ* predicts *cis*-motif usage? This result we have yet to demonstrate. The prediction we make is that species with low *N*_e_*.μ* will be species with more common skews in codon or amino acid usage owing to selection for *cis-*modifiers of splicing. Unexpectedly, despite having observed all prior correlations (*N*_e_*.μ* predicts intron dimensions and splice site usage, intron dimensions and splice site usage predict *cis-*motif usage), we fail to recover a trend whereby *cis-*motif usage is predicted by *N*_e_*.μ* (table 6). For the *N*_e_*.μ* estimator *S,* there may be a weak trend but for others there is no evidence. Employing the sample size uncorrected measure of the number of trends removes any weak trend reported for *S* (table 6). We conclude that we find evidence that splice site usage, but not *cis*-motif usage, correlates with *N*_e_*.μ*.

**Table 6.** Little Evidence for a Phylogenetically Controlled Correlation between *N*_e_*.μ* Values and Amino Acid/Codon Usage Trends (Y).

	All Exons (AA)	All Exons (codon)	Random 5,000 Exons (AA)	Random 5,000 Exons (codon)
Log BF (*N*_e_*.μ*_Pi ∼ Y)^a^	−0.486	−2.065	−2.693	−4.436
Log BF (*N*_e_*.μ*_*S* ∼ Y)	−1.383	−0.206	1.514	0.728
Log BF (*N*_e_*.μ*_Eta ∼ Y)	0.534	−0.520	−2.038	1.079

Note.—We employ our three different estimators for *N*_e_*.μ* (Eta, Pi, and S) and four metrics of *k*-mer usage. *Y*, proportion of amino acids/codons showing significant trends.

^a^Log BF (log Bayes factor) = 2*(log [harmonic mean (complex model)] − log [harmonic mean (simple model)]), is the test statistic of BayesTraits which gives the information of evidence for correlated evolution: weak evidence (<2), positive evidence (>2), strong evidence (5–10), very strong evidence (>10). All Log BF values in the table are less than 2, so the evidences of all correlations are weak.

### Alternative Splicing Rate Does Not Explain *cis*-Motif Usage

One reason that *N*_e_*.μ* might not predict *cis*-motif usage is that other covariates are important and mask any effect. A potentially key covariable might be the frequency of alternative splicing. We observed previously that the brown algae *Ectocarpus* has a striking number of codons and amino acids showing skews in usage in the vicinity of exon junctions, many more indeed than humans (Wu et al. 2013). This we hypothesized may reflect the low rate of alternative splicing that we could detect. If alternative splicing is rare in a species, then more of the annotated exons will be under selection to be properly spliced more of the time. Alternatively, ESEs might modulate alternative splicing, which is more common in “complex” species (Chen et al. 2014), typically with low *N*_e_. Note that these two models make opposite predictions.

To provide an assessment of this, we consider transcript depth-controlled estimates of the rate of alternative splicing for 14 species (Chen et al. 2014). We find strong evidence to support the correlation between alternative splicing rates with the ratio of mature CDS to gene size. Although intron density is a better predictor of *cis-*motifs than is intron size, the correlation between alternative splicing rates and mean intron size is better than that with intron density (table 7). Between-species differences in alternative splicing rates do not, however, predict between-species trends in *cis-*motif usage very well (supplementary table S5.2). We conclude that although alternative splicing rates and intronic dimensions covary, the former appears not to explain trends in *cis*-motif usage.

**Table 7.** Evidence for Correlation between Alternative Splicing Rates and Splice-Related Genomic Traits.

	X	N	M
Log BF (ASL1 ∼ Splice-related Genomic Traits)^a^	5.259	2.782	7.299
Log BF (ASL2 ∼ Splice-related Genomic Traits)	8.714	4.589	9.500

ASL1, average number of ASEs per gene (residual of the polynomial regression between num of ESTs [col. O] and ASL [col. U]); ASL2, average number of ASEs per gene (residual of the linear regression between the log-transformed num of ESTs [col. O] and ASL [col. U]); *X*, mean CDS length/gene length; *N*, introns per kb exon; *M*, mean intron size.

^a^Log BF (log Bayes factor) = 2*(log [harmonic mean (complex model)] − log [harmonic mean (simple model)]), is the test statistic of BayesTraits which gives the information of evidence for correlated evolution: Weak evidence (<2), positive evidence (>2), strong evidence (5–10), very strong evidence (>10).

### Is the Commonality of Decoy Splice Sites the Main Driver of Splice Associated *cis*-Motif Usage?

Why might it be that the best between-species predictor of *cis-*motif usage was not simply mean intron size, but an aggregate measure of size and density? From the logic that we laid out (difficulty of exon junction recognition in the context a large flanking intron), this is perhaps unexpected. We suggest that the problem may be one of decoy splice sites. Imagine a gene with one large intron and no residues elsewhere downstream of the true 3′ splice site that might be recognized as a possible acceptor site. Would such a gene have error-prone splicing? We would suggest not if there is a unique strong site (the true acceptor site) compatible with splicing. By contrast, by definition, the same gene with two introns must have at least two putative acceptor sites. Thus the more introns and the weaker the splice sites, the more potential there is for missplicing.

This suggests then a simple explanation for why intron density matters. We assume that SR proteins bound to the immature RNA accumulate at exon ends bound to ESEs. A given 5′ splice site, we assume also, tends to attach to the perceived nearest 3′ splice site, this being identified by the accumulation of ESEs and SR proteins. The extent of accumulation of ESEs we suggest is a function of the chance the splice site might be “missed.” Strong splice sites in close proximity (short introns) are unlikely to be missed and hence need little reinforcement. By contrast ESEs are needed more in the vicinity of larger introns as the ability to find the nearest 3′ splice site is harder owing to the distance and because the number of decoy sites is higher, that is, when the density of introns is higher. However, whether it is density per se or absolute number of introns that is key is not immediately transparent, as it is unclear whether the absolute proximity of decoy splice sites to the “real” splice site is relevant. If physical proximity is relevant then density may matter, if not absolute number may be more important.

Such a model makes an intragenomic prediction, namely that controlling for intron length, intron density or number should predict ESE density. From the partial correlation analysis between ESE density and three intronic dimensions (mean intron size, intron density, and intron number), all 5′-end calculations show very significant partial correlations, regardless of the choice of ESE data set. At the 3′-end, the result is less clear. For INT3 ESE data set, the correlation between ESE density and intron density is not significant, whereas intron number and intron size are predictors. At 3′–ends, all partial correlations for INT3_400 are not significant (supplementary table S2.1). The correlation with intron number is perhaps the most revealing, suggesting that density per se functions as a proxy to absolute number and hence that exon size considerations are not so relevant. Further, these results suggest that, although ESE usage at 5′- and 3′-ends of exons is usually considered to be symmetrical in humans (motifs commonly found at 5′-ends tend to be common at 3′-ends [Warnecke et al. 2008; Lim et al. 2011]), that at least as regards intron density mediated effects 5′- and 3′-ends are under different modes of selection. The suggestive evidence that net selection on ESEs is better correlated with intron length for 5′- ends than 3′-ends supports the same proposition, as does the 5′–3′ difference in splice site predicted by *N*_e_*.μ*.

If the problem faced is one in which downstream exons and introns presenting decoy splice sites, then we might also expect a difference in ESE density within a gene, as different exons have a different number of downstream introns and exons and hence a different number of potential decoy splice sites. We address this by comparing the ESE density at the 5′-end of the second exon in a gene and the 5′-ESE density at the last but one exon in genes with at least four exons. We do not employ the very last exon owing to possible constraints on nucleotide content in the vicinity of the stop codon.

We find strong evidence that intragene location matters, with ESE density higher earlier in a gene. From comparing the ESE density at the 5′-end of the second exon in a gene and the 5′-ESE density at the last but one exon in genes with at least four exons, the medians of ESE density of last but one exons and second exons are about 2-fold different (INT3 ESE density: 0.0909 and 0.1739, INT3_400 ESE density: 0.0882 and 0.1739), in last but one and second exon, respectively (supplementary table S6.1). To examine the significance of this we perform a paired test, comparing the ESE density within the same gene between the two exon 5′-flanks. Results are as expected of the decoy splice site model. For INT3 data set, the number of genes which show ESE density of the second exon to be higher than that of last but one exon, reaches 491 and the number where ESE density of second exon is relatively lower is 381 (binomial test *P* = 2.6 × 10^−^^5^). For INT3_400 data set, the corresponding values are 332 (ESE density of second exon is higher) and 240 (ESE density of second exon is lower), again supporting a higher density in second exons (binomial test *P* = 2.0 × 10^−^^5^).

To test whether the trend is owing to confounding effects of proximal intron size, we employed Mann–Whitney *U* test to analyze residuals of a loess regression while 5′ proximal intron size is being controlled. Results are again as predicted by the decoy model. We again find a significantly higher ESE density at 5′ end of second exons compared with last but one exons (Mann–Whitney *U* test comparing residuals of 5′ intron size vs. ESE density, INT3: *P* = 2.42 × 10^−^^71^, INT3_400: *P* = 5.32 × 10^−^^46^). A within-gene paired test on residuals from above loess regression supports the same conclusions (INT3_400: number of higher second exon residuals = 386, number of lower second exon residuals = 289, binomial test *P* = 2.85 × 10^−^^5^; INT3: number of higher second exon residuals = 566, number of lower second exon residuals = 404, *P* = 3.25 × 10^−^^8^). A higher density of ESEs earlier in a gene is, we suggest, consistent with the decoy model given that early exons by definition have more downstream splice sites than do later ones. It also suggests a novel (to our knowledge) model of splicing reinforcement that is different in different sections of the same gene.

## Discussion

We conjectured that reduced *N*_e_ might lead to larger introns and weakened splice sites, which in turn could lead to stronger selection for motifs that keep in check the increase in the degree of error-prone splicing. All results bar one support this. We find that synonymous sites are more commonly under selection within humans when exons are flanked by larger introns (largely because more sites function as *cis-*motifs), that intronic dimensions and splice site usage predict *cis-*motif usage across species, and that *N*_e_*.μ* predicts intronic dimensions and splice site usage (we note that this tidies up the prior objection that in a phylogenetic framework the results of Lynch and Conery do not hold [Whitney and Garland 2010]). In addition, we find that intraspecifically, exons flanked by large introns both have higher ESE density and greater usage of AGgt, consistent with coevolution between splice site, ESEs and intron size. What we do not observe is that *N*_e_*.μ* predicts *cis-*motif usage.

Given the support for the hypothesis from all but one of the tests, we suggest that it would be premature to reject the hypothesis out of hand. Indeed, one possibility is that our estimation of *N*_e_*.μ* is either too rough or otherwise flawed. It is striking, for example, that our estimation and that of Lynch and Conery do not correlate well, despite being based on the same underlying premise. Moreover there might be a systematic issue with all polymorphism-based attempts to estimate *N*_e_*.μ*, this being that the expected correlation between *N*_e_ and heterozygozity appears to be much weaker than predicted by the neutral model (which forms the basis for *N*_e_*.μ* estimation). Gillespie (2001) argues that the approximate invariance (or weak positive correlation) between *N*_e_ and heterozygozity is owing to an increased rate of positive selection when populations are large, thereby causing regular collapses of heterozygozity owing to hitchhiking type effects. We do not wish to comment on the veracity of this claim, but simply wish to note that of all the variables that we have employed, *N*_e_*.μ* is the one we have least confidence in, both as regards its estimation and its interpretation. Recent evidence that intraspecific diversity is predicted by life-history traits (Romiguier et al. 2014) adds to the notion that a relationship between *N*_e_*.μ*, deduced from heterozygozity data, and the strength of selection may be compounded by covariates. Nonetheless, we observe that *N*_e_*.μ* robustly predicts intronic dimensions and splice site usage, suggesting that it is perhaps not too poor an estimator.

Although we have framed the above hypotheses and results in the context of the nearly neutral model, the same results might, however, also be consistent with a model in which increasing *cis*-motif usage across taxa reflects greater tissue or cell type diversity, ESEs then operating as providers of tissue-specific alternative splice patterns. It is indeed observed that species with more cell types do have more alternative transcripts (Chen et al. 2014). Might this coupling be explained by increased usage of ESEs? Our and other results suggest not. We observe no relationship between *cis*-motif usage and alternative splicing rates. Moreover, there is no strong prior evidence to suppose that ESE usage is a modulator of alternative splicing. Indeed, although our intersect data sets find no difference in ESE density between alternative and constitutive exons (Cáceres and Hurst 2013), an experimentally defined set of exonic splice modifiers (Ke et al. 2011) found a much higher ESE density in constitutive than in alternative exons. Earlier reports also indicated that, although conserved alternative exons have very low rates of evolution, this was not owing to especially strong constraint on ESEs (Parmley et al. 2006; Cáceres and Hurst 2013). These results thus suggest that ESEs are not there as elements to control alternative splicing forms, but rather to make more robust the splicing of constitutive exons, especially those with weak splice sites. For these reasons, we suggest that higher transcript diversity in species with small population sizes/multiple tissue types is not an easily defendable explanation for the trends in *cis*-motif usage.

An unexpected result was that in the between-species comparison, intron size is by no means the best intron-dimension predictor of *cis*-motif usage. Rather a combination of size and density is a much better predictor. We propose a decoy splice spite model as a potential explanation. This model correctly predicts intragenomic and intragenic trends, highlighting the selection on the earliest exons as being especially acute. The intragenic trend may however have an alternative explanation, namely that it is simply more damaging to missplice an early exon than it is to missplice a later exon. For example, the downstream effects of a frame-shifting splice event may be different for the two. It is not so obvious that such an argument can explain the intragenomic, intergenic trends (i.e., mean intron size, intron density, and intron number all independently predict 5′-ESE usage). This model and the apparent asymmetry between 5′- and 3′-effects are, we suggest, worthy of further scrutiny.

## Materials and Methods

### Exon and Intron Sequences from 30 Species

From “Table Browser” of UCSC (http://genome.ucsc.edu/cgi-bin/hgTables, last accessed January 23, 2014) and FTP site of NCBI (ftp://ftp.ncbi.nlm.nih.gov/genomes, last accessed January 23, 2014), we obtained all available genes from 30 species (*Anolis carolinensis, Anopheles gambiae, Arabidopsis thaliana, Brachypodium distachyon, Caenorhabditis elegans, Callithrix jacchus, Cryptococcus neoformans, Dictyostelium discoideum, Drosophila melanogaster, Danio rerio, Ectocarpus siliculosus, Gallus gallus, Gorilla gorilla, Homo sapiens, Ictidomys tridecemlineatus, Meleagris gallopavo, Macaca mulatta, Mus musculus, Oryzias latipes, Oryza sativa, Pongo abelii, Plasmodium falciparum, Paramecium tetraurelia, Pan troglodytes, Saccharomyces cerevisiae, Schizosaccharomyces pombe, Strongylocentrotus purpuratus, Sus scrofa, Takifugu rubripes, Xenopus tropicalis*). Sequences without the normal start (ATG) and stop codons (TAA, TAG, and TGA), with internal stop codons, ambiguous nucleotides (“N”), and without introns were all removed from the data set (supplementary table S7.1).

### Determining Trends in Amino Acid and Codon Usage

In previous analyses, codons preferred near exon ends were well predicted by the composition of experimentally defined ESEs (Parmley and Hurst 2007; Cáceres and Hurst 2013). We thus presume that the frequency of distorted codon or amino acid usage in vicinity of exon junctions is a fair measure of *cis-*splice motif usage. The trend in usage of each codon and amino acid was investigated as a function of the distance from the exon–intron boundary up to a distance of 34 codons (to accord with an earlier analysis [Warnecke et al. 2008]). The 5′- and 3′-ends were analyzed separately with the codon in direct proximity to the boundary being eliminated and the first and last exons being excluded. For each codon and amino acid under consideration, we determined, after Bonferroni correction, rho and *P* value by two-tailed Spearman correlation of proportional usage as a function of distance from the boundary. A negative rho indicates a codon or amino acid that is preferred near exon ends, whereas a positive value implies a codon or amino acid preferred at exonic cores and avoided at the ends. For each species, we then calculate the proportion of codons or amino acids showing significant skew both at 5′- and 3′-ends across all exons and consider this the metric of *cis*-motif usage for that species.

In order to ensure that these trend comparisons are not affected by the different number of exons in different species, for each species, we made a pool of exons and abstracted 5,000 exons from it randomly with replacement (for each repeat of 30 species, 30 data sets were established with each containing 5,000 exons). After 100 repetitions of this sampling process, we obtained the mean usage trends of amino acids and codons for each species by the same method mentioned above. We counted up the number of amino acids or codons that showed a significant rho score in the sample size controlled subsampling and employed this as our metric of the extent of *cis-*motif usage (supplementary table S7.2). We also report results for a sample size uncorrected metric.

### Splice-Related Genomic Traits

Based on the data sets of genes saved, we calculated three parameters: *X* (mean CDS length/gene length), *N* (introns per kb exon), and *M* (mean intron size) for each species (supplementary table S7.3).

### Phylogenetic Tree with Branch Length

A text file containing an ID list of the 30 species was uploaded to “Taxonomy Browser” of NCBI (http://www.ncbi.nlm.nih.gov/Taxonomy/CommonTree/wwwcmt.cgi, last accessed January 23, 2014), and then we saved the Taxonomy Common Tree, which has no branch length, in PHYLIP format. To obtain the branch lengths of the phylogenetic tree, a multiple sequence alignment was needed. We searched for candidate orthologs through the orthologous database OrthoDB (http://cegg.unige.ch/orthodb7, last accessed January 23, 2014) and HomoloGene (http://www.ncbi.nlm.nih.gov/homologene, last accessed January 23, 2014) of NCBI, by taking gene function (related with temperature, air pressure, oxygen concentration, or acid-base properties) into consideration. Ten orthologous genes (supplementary table S7.4), conserved in Eukaryotes, were finally used to make alignments by M-Coffee (http://tcoffee.crg.cat/apps/tcoffee/do:mcoffee, last accessed January 23, 2014) separately. All the ten alignments were merged into one. Gblocks (Castresana 2000; Talavera and Castresana 2007) was employed to eliminate/minimize poorly aligned positions and divergent regions (Supplementary material S1) and converted from Newick format into Nexus within R (parameters used can be found in Supplementary material S2).

Through the RelTime application (Kumar et al. 2012; Tamura et al. 2012, 2013), a phylogenetic tree with branch lengths was constructed by loading the taxonomy common tree and the alignment into MEGA (version 6). We tested the correlation of results from two models (Jones–Taylor–Thornton model and WAG [Whelan and Goldman] model) when selecting “Gamma Distributed” of “Rates and Patterns” and other default parameters. There is a very strong correlation between the branch length estimates from the two models (Spearman correlation: ρ = 0.9970, *P* = 4.17 × 10^−^^65^; supplementary fig. S2). We regarded the mean of the results as the final branch lengths (supplementary fig. S3).

### Correlation between Amino Acid/Codon Usage Trends and the Genomic Traits after Phylogenetic Correction

The application “Continuous” of BayesTraits (Pagel 1999) was used to study correlations between amino acid/codon usage trends and the genomic traits by Markov chain Monte Carlo method. According to the suggestion from the manual of BayesTraints, we abstracted the last harmonic mean from the result file, and took it as an estimation of marginal likelihood, to calculate the “Log BF” value and further test whether there is strong evidence for the correlation after phylogenetic correction.

### Correlation Analysis of *N*_e_.*μ* Values in Phylogenetic Manner

To calculate *N*_e_*.μ* values of the species, several R packages (ape [Paradis et al. 2004], PopGenome [Pfeifer et al. 2014], adegenet [Jombart 2008; Jombart and Ahmed 2011], pegas [Paradis 2010], Geiger [Harmon et al. 2008]) and DnaSP (Rozas and Rozas 1995; Librado and Rozas 2009) were used to analyze the published allelic sequences from “PopSet” (http://www.ncbi.nlm.nih.gov/popset, last accessed January 23, 2014) and “Nucleotide” (http://www.ncbi.nlm.nih.gov/nuccore, last accessed January 23, 2014) database of NCBI. Intron sequences were considered as candidates first and, if there is no intron sequence, CDS were chosen for the analysis in which only synonymous sites number, as the segregating sites number, were input the program (supplementary table S8.1). Finally, *N*_e_*.μ* values (*N*_e_*.μ* for per site from Pi, *N*_e_*.μ* for per site from S, and *N*_e_*.μ* for per site from Eta) of each species were obtained for further correlation analysis (supplementary table S8.2). *N*_e_*.μ* values from this study were compared, by Spearman’s correlation and SMA regression of R package “lmodel2,” with the *N*_e_*.μ* values (Lynch and Conery 2003) published previously (supplementary table S8.3).

By using BayesTraits, in phylogenetic manner, we did correlation analysis of *N*_e_*.μ* values (both the values from our study and from Lynch and Conery’s study) with amino acid/codon usage trends and three intronic dimensions (mean CDS length/gene length, intron density, and mean intron size) (supplementary table S8.4).

### Comparison of Selection on Synonymous Mutations with Different Flanking Intron Size

A list of human–macaque orthologs was obtained from ENSEMBL (Flicek et al. 2014). Only those defined as 1:1 orthologs were employed. The respective genes were extracted from human CDS build GRCh37.74 and Macaque build MMUL_1.74. These were aligned using MUSCLE 3.8.31 (Edgar 2004) at the protein level, the nucleotide alignment being built from the protein alignment using a custom script (AA2NUC). Exon and intron sizes for the relevant human genes were obtained through ENSEMBL. Any gene whose CDS length did not match that specified in the BioMart (Kasprzyk 2011) derived annotation file was excluded. The alignment of the exons was derived from the exon dimensions specified (naturally with allowance for indels). Only internal (not first or last exons) exons from the macaque–human comparison were employed.

We considered only exons longer than 2 × 69 bp and considered the 5′ 69 bp as the 5′-end and 3′ 69 bp as the 3′-end. The alignments were masked with two consensus ESE candidate data sets, INT3 and INT3-400, these being intersect data sets between four high coverage databases of putative ESE sequences (Cáceres and Hurst 2013). One of the data sets presents a large sample of putative ESEs and a second (*N* = 400) top hit sample. As these are nonindependent the intersect data sets employ either the full sample (INT3) or the reduced sample (INT3-400). We could thus, employing these two separately, define sites that were ESE and sites that were possibly not ESE (although as these two sets were conservative, there are likely to be true ESEs in the non-ESE class of sequence). For both ESE and non-ESE masking of the alignments, we then concatenate all exon ends as a function of 20 different flanking intron sizes, thereby making estimation of Ks less noisy. We also compared Ks of exon cores (69 bp of core region in each exon) as a function of the size of neighboring introns after concatenating core sequences in each bin. For each of the sets of concatenated exon ends and cores, both ESE and non-ESE, we estimate Ks using PAML (version: PAML 4.7, Default parameters are used, codon model = 2) (Yang 2007).

To exclude the possibility that any trends seen are not artifacts of skewed nucleotide content between ESE and non-ESE sequence, we generated pseudo-ESE sets containing the same number of random hexamers with, on average, the same nucleotide content as each ESE set. Then, the same test as above was performed 100 times repeatedly for each pseudo-ESE set. The average value with standard error bar from these nucleotide controls is displayed in the plots (fig. 1*A* and *B*).

### Partial Correlation between ESE Density and Three Intronic Dimensions

ESE density and three intronic dimensions (mean intron size, intron density, and intron number) were obtained using custom perl scripts. When two of the intronic dimensions are controlled, partial correlation between ESE density and another intronic dimension was analyzed by R program, pcor.test (Kim and Soojin 2006) (supplementary table S2.1).

### Correlation between Flank ESE Constraint and Three Intronic Dimensions

Based on the alignment data set of human–macaque orthologs, Ks core and Ks ESE flank (both 5′ 69 bp and 3′ 69 bp, exons are shorter than 138 bp were all regarded as flank region), of each gene, were calculated after concatenating all flank and core sequences. We set up three criteria for concatenating sequence to select genes for correlation analysis (1. ESE flank > 102 bp, Core region > 102 bp; 2. ESE flank > 150 bp, Core region > 150 bp; 3. ESE flank > 201 bp, Core region > 201 bp). Then, three intronic dimensions (mean intron size, intron density, and intron number) and Flank ESE constraint of each gene were obtained by our perl script. For INT3 and INT3_400 data sets, we explored the correlation between Flank ESE constraint (defined in results) and three intronic dimensions, considering 5′- and 3′-ends of exons separately, by partial Spearman’s correlation (supplementary table S3.2).

We evaluate the net effect of flanking intron size (constraint and increased density) by calculating the proportion of synonymous sites under ESE-related constraint at exon flanks as flank ESE constraint × ESE density. Instead of using the conservative binning method (*N* = 20), we calculated the regression line of logarithm value of flank intron size versus ESE constraint and for each exon individually calculate ESE density × constraint, where constraint is estimated through interpolation of this regression line, given the intron size. Then, we examine whether the Spearman’s rank correlation between ESE density × constraint and the logarithm value of intron size is significant.

Furthermore, Goodman and Kruskal’s gamma, by using program “rcorr.cens” from R package “Hmisc” (http://biostat.mc.vanderbilt.edu/Hmisc, https://github.com/harrelfe/Hmisc, last accessed January 23, 2014) was carried out in above analysis to avoid affects of tied observations. *P* value, which shows whether Goodman and Kruskal’s gamma is significant, comes from *p* = (*n* + 1)/(*m* + 1) where *n* is the number of gamma values calculated after randomly shuffled the variables representing flank ESE constraint and meanwhile greater than the observed gamma and *m* is 1,000, this being the number of times of shuffling (supplementary table S3.1).

To make sure the above result is not affected by sample size artifacts, we did a resampling test by abstracting genes by the number in the 150-bp cutoff group from the gene pool in the 120-bp cutoff group and repeated the two types of correlation analysis 1,000 times. We report the proportion of random subsamplings that still provide a significant correlation prior to multitest correction.

### Comparison of ESE Density between Second Exons and Last but One Exons

In the human gene data set, we selected from genes with four or more exons, second exons and last but one exons, which are all greater than 138 bp. We calculated 5′-exon end ESE density and 5′-flank intron size of these two exon categories in each gene. To control for the effect of flank intron size, we analyzed the residuals from loess regression of 5′-end ESE density predicted by 5′-intron size (supplementary table S6.1). Both analyses were repeated for the two ESE data sets (INT3 and INT3_400). Significance was assayed through a binomial test counting the absolute number of genes having a higher density at the second exon than the last but one, versus the opposite. If ESE density was no different, these were ignored.

### Relationship between Transcriptional Elongation Rate and ESE Density

We used publicly available data from a genome-wide elongation rate study (Veloso et al. 2014) to investigate the relationship of ESE density with transcriptional elongation rate (around 450 genes were selected due to requirement of ESE density calculation; supplementary table S4.1) and also correlated the elongation rates with several genic dimensions used in our study (supplementary table S4.2).

## Supplementary Material

Supplementary tables S1–S8, figures S1–S3, and files S1 and S2 are available at *Molecular Biology and Evolution* online (http://www.mbe.oxfordjournals.org/).

Supplementary Data
