# Supplementary material for: Why Selection Might Be Stronger When Populations Are Small: Intron Size and Density Predict within and between-Species Usage of Exonic Splice Associated cis-Motifs
Source: Mol Biol Evol. 2015 Mar 13;32(7):1847–61. doi: 10.1093/molbev/msv069 (PMC4476162; doi:10.1093/molbev/msv069)
Supplement: Supplementary Data [file supp_32_7_1847__index.html]

Why Selection Might Be Stronger When Populations Are Small: Intron Size and Density Predict within and between-Species Usage of Exonic Splice Associated cis-Motifs — Why Selection Might Be Stronger When Populations Are Small: Intron Size and Density Predict within and between-Species Usage of Exonic Splice Associated cis-Motifs — Supplementary Data 

# Why Selection Might Be Stronger When Populations Are Small: Intron Size and Density Predict within and between-Species Usage of Exonic Splice Associated *cis-*Motifs

## Supplementary Data

files

**Files in this Data Supplement:**

- Supplementary Data - zip file
