# Supplementary material for: Why Selection Might Be Stronger When Populations Are Small: Intron Size and Density Predict within and between-Species Usage of Exonic Splice Associated cis-Motifs
Source: Mol Biol Evol. 2015 Mar 13;32(7):1847–61. doi: 10.1093/molbev/msv069 (PMC4476162; doi:10.1093/molbev/msv069)
Supplement: Supplementary Data [file supp_msv069_suppl_data.zip › Supplementary_Tables_7.docx]

**Supplementary table 7.1. Number of genes downloaded and used**

| Species | Genes_used | Gene_total |
| --- | --- | --- |
| *A.carolinensis* ([Alfoldi et al. 2011](#_ENREF_1)) | 2093 | 12985 |
| *A.gambiae* ([Holt et al. 2002](#_ENREF_12)) | 3877 | 15802 |
| *A.thaliana* ([Schneeberger et al. 2011](#_ENREF_24)) | 27875 | 27961 |
| *B.distachyon* ([Lucas 2010](#_ENREF_22)) | 17932 | 23318 |
| *C.elegans* ([*C. elegans* Sequencing Consortium 1998](#_ENREF_3)) | 24972 | 26415 |
| *C.jacchus* ([Worley KC 2014](#_ENREF_28)) | 1701 | 8630 |
| *C.neoformans* ([Loftus et al. 2005](#_ENREF_21)) | 6234 | 6271 |
| *D.discoideum* ([Eichinger et al. 2005](#_ENREF_8)) | 8877 | 9000 |
| *D.melanogaster* ([Celniker et al. 2002](#_ENREF_4)) | 23287 | 28138 |
| *D.rerio* ([Howe et al. 2013](#_ENREF_13)) | 10801 | 13696 |
| *E.siliculosus* ([Cock et al. 2010](#_ENREF_6)) | 11198 | 15099 |
| *G.gallus* ([International Chicken Genome Sequencing Consortium. 2004](#_ENREF_15)) | 4224 | 5901 |
| *G.gorilla* ([Scally et al. 2012](#_ENREF_23)) | 1292 | 10011 |
| *H.sapiens* (gh19, ([Lander et al. 2001](#_ENREF_18))) | 7233 | 7894 |
| *I.tridecemlineatus* ([Lindblad-Toh et al. 2011](#_ENREF_19)) | 3680 | 13218 |
| *M.gallopavo* ([Dalloul et al. 2010](#_ENREF_7)) | 780 | 10347 |
| *M.mulatta* ([Yan et al. 2011](#_ENREF_29)) | 1233 | 5896 |
| *M.musculus* ([Waterston et al. 2002](#_ENREF_26)) | 11332 | 13022 |
| *O.latipes* ([Kasahara et al. 2007](#_ENREF_17)) | 251 | 601 |
| *O.sativa* ([Yu et al. 2005](#_ENREF_30)) | 51108 | 66171 |
| *P.abelii* ([Locke et al. 2011](#_ENREF_20)) | 648 | 3540 |
| *P.falciparum* ([Gardner et al. 2002](#_ENREF_10)) | 2886 | 2903 |
| *P.tetraurelia* ([Aury et al. 2006](#_ENREF_2)) | 24079 | 39034 |
| *P.troglodytes* ([Hughes et al. 2005](#_ENREF_14)) | 2616 | 6605 |
| *S.cerevisiae* (S288c, ([Cherry et al. 1997](#_ENREF_5))) | 310 | 6692 |
| *S.pombe* ([Wood et al. 2002](#_ENREF_27)) | 2104 | 2253 |
| *S.purpuratus* ([Sodergren et al. 2006](#_ENREF_25)) | 111 | 576 |
| *S.scrofa* ([Fang et al. 2012](#_ENREF_9)) | 9111 | 22313 |
| *T.rubripes* ([Kai et al. 2011](#_ENREF_16)) | 7025 | 36144 |
| *X.tropicalis* ([Hellsten et al. 2010](#_ENREF_11)) | 2873 | 9013 |

Reference of Supplementary table 7.1

Alfoldi J, Di Palma F, Grabherr M, Williams C, Kong L, Mauceli E, Russell P, Lowe CB, Glor RE, Jaffe JD, et al. 2011. The genome of the green anole lizard and a comparative analysis with birds and mammals. Nature 477:587-591.

Aury JM, Jaillon O, Duret L, Noel B, Jubin C, Porcel BM, Segurens B, Daubin V, Anthouard V, Aiach N, et al. 2006. Global trends of whole-genome duplications revealed by the ciliate Paramecium tetraurelia. Nature 444:171-178.

C. elegans Sequencing Consortium. 1998. Genome sequence of the nematode C. elegans: a platform for investigating biology. Science 282:2012-2018.

Celniker SE, Wheeler DA, Kronmiller B, Carlson JW, Halpern A, Patel S, Adams M, Champe M, Dugan SP, Frise E, et al. 2002. Finishing a whole-genome shotgun: release 3 of the Drosophila melanogaster euchromatic genome sequence. Genome Biol 3:RESEARCH0079.

Cherry JM, Ball C, Weng S, Juvik G, Schmidt R, Adler C, Dunn B, Dwight S, Riles L, Mortimer RK, et al. 1997. Genetic and physical maps of Saccharomyces cerevisiae. Nature 387:67-73.

Cock JM, Sterck L, Rouze P, Scornet D, Allen AE, Amoutzias G, Anthouard V, Artiguenave F, Aury JM, Badger JH, et al. 2010. The Ectocarpus genome and the independent evolution of multicellularity in brown algae. Nature 465:617-621.

Dalloul RA, Long JA, Zimin AV, Aslam L, Beal K, Blomberg Le A, Bouffard P, Burt DW, Crasta O, Crooijmans RP, et al. 2010. Multi-platform next-generation sequencing of the domestic turkey (Meleagris gallopavo): genome assembly and analysis. PLoS Biol 8.

Eichinger L, Pachebat JA, Glockner G, Rajandream MA, Sucgang R, Berriman M, Song J, Olsen R, Szafranski K, Xu Q, et al. 2005. The genome of the social amoeba Dictyostelium discoideum. Nature 435:43-57.

Fang X, Mou Y, Huang Z, Li Y, Han L, Zhang Y, Feng Y, Chen Y, Jiang X, Zhao W, et al. 2012. The sequence and analysis of a Chinese pig genome. Gigascience 1:16.

Gardner MJ, Hall N, Fung E, White O, Berriman M, Hyman RW, Carlton JM, Pain A, Nelson KE, Bowman S, et al. 2002. Genome sequence of the human malaria parasite Plasmodium falciparum. Nature 419:498-511.

Hellsten U, Harland RM, Gilchrist MJ, Hendrix D, Jurka J, Kapitonov V, Ovcharenko I, Putnam NH, Shu S, Taher L, et al. 2010. The genome of the Western clawed frog Xenopus tropicalis. Science 328:633-636.

Holt RA, Subramanian GM, Halpern A, Sutton GG, Charlab R, Nusskern DR, Wincker P, Clark AG, Ribeiro JM, Wides R, et al. 2002. The genome sequence of the malaria mosquito Anopheles gambiae. Science 298:129-149.

Howe K, Clark MD, Torroja CF, Torrance J, Berthelot C, Muffato M, Collins JE, Humphray S, McLaren K, Matthews L, et al. 2013. The zebrafish reference genome sequence and its relationship to the human genome. Nature 496:498-503.

Hughes JF, Skaletsky H, Pyntikova T, Minx PJ, Graves T, Rozen S, Wilson RK, Page DC. 2005. Conservation of Y-linked genes during human evolution revealed by comparative sequencing in chimpanzee. Nature 437:100-103.

International Chicken Genome Sequencing Consortium. 2004. Sequence and comparative analysis of the chicken genome provide unique perspectives on vertebrate evolution. Nature 432:695-716.

Kai W, Kikuchi K, Tohari S, Chew AK, Tay A, Fujiwara A, Hosoya S, Suetake H, Naruse K, Brenner S, et al. 2011. Integration of the genetic map and genome assembly of fugu facilitates insights into distinct features of genome evolution in teleosts and mammals. Genome Biol Evol 3:424-442.

Kasahara M, Naruse K, Sasaki S, Nakatani Y, Qu W, Ahsan B, Yamada T, Nagayasu Y, Doi K, Kasai Y, et al. 2007. The medaka draft genome and insights into vertebrate genome evolution. Nature 447:714-719.

Lander ES, Linton LM, Birren B, Nusbaum C, Zody MC, Baldwin J, Devon K, Dewar K, Doyle M, FitzHugh W, et al. 2001. Initial sequencing and analysis of the human genome. Nature 409:860-921.

Lindblad-Toh K, Garber M, Zuk O, Lin MF, Parker BJ, Washietl S, Kheradpour P, Ernst J, Jordan G, Mauceli E, et al. 2011. A high-resolution map of human evolutionary constraint using 29 mammals. Nature 478:476-482.

Locke DP, Hillier LW, Warren WC, Worley KC, Nazareth LV, Muzny DM, Yang SP, Wang Z, Chinwalla AT, Minx P, et al. 2011. Comparative and demographic analysis of orang-utan genomes. Nature 469:529-533.

Loftus BJ, Fung E, Roncaglia P, Rowley D, Amedeo P, Bruno D, Vamathevan J, Miranda M, Anderson IJ, Fraser JA, et al. 2005. The genome of the basidiomycetous yeast and human pathogen Cryptococcus neoformans. Science 307:1321-1324.

Lucas S, Harmon-Smith,M., Lail,K., Tice,H. 2010. Genome sequencing and analysis of the model grass Brachypodium distachyon. Nature 463:763-768.

Scally A, Dutheil JY, Hillier LW, Jordan GE, Goodhead I, Herrero J, Hobolth A, Lappalainen T, Mailund T, Marques-Bonet T, et al. 2012. Insights into hominid evolution from the gorilla genome sequence. Nature 483:169-175.

Schneeberger K, Ossowski S, Ott F, Klein JD, Wang X, Lanz C, Smith LM, Cao J, Fitz J, Warthmann N, et al. 2011. Reference-guided assembly of four diverse Arabidopsis thaliana genomes. Proc Natl Acad Sci U S A 108:10249-10254.

Sodergren E, Weinstock GM, Davidson EH, Cameron RA, Gibbs RA, Angerer RC, Angerer LM, Arnone MI, Burgess DR, Burke RD, et al. 2006. The genome of the sea urchin Strongylocentrotus purpuratus. Science 314:941-952.

Waterston RH, Lindblad-Toh K, Birney E, Rogers J, Abril JF, Agarwal P, Agarwala R, Ainscough R, Alexandersson M, An P, et al. 2002. Initial sequencing and comparative analysis of the mouse genome. Nature 420:520-562.

Wood V, Gwilliam R, Rajandream MA, Lyne M, Lyne R, Stewart A, Sgouros J, Peat N, Hayles J, Baker S, et al. 2002. The genome sequence of Schizosaccharomyces pombe. Nature 415:871-880.

Worley KC WW, Rogers J, Locke D, Muzny DM. 2014. The common marmoset genome provides insight into primate biology and evolution. Nat Genet.

Yan G, Zhang G, Fang X, Zhang Y, Li C, Ling F, Cooper DN, Li Q, Li Y, van Gool AJ, et al. 2011. Genome sequencing and comparison of two nonhuman primate animal models, the cynomolgus and Chinese rhesus macaques. Nat Biotechnol 29:1019-1023.

Yu J, Wang J, Lin W, Li S, Li H, Zhou J, Ni P, Dong W, Hu S, Zeng C, et al. 2005. The Genomes of Oryza sativa: a history of duplications. PLoS Biol 3:e38.

**Supplementary table 7.2. Proportion of amino acids/Codon showing significant trends as a metric of the extent of *cis* motif usage**

| Species | All exons | |  | Random 5000 exons | |
| --- | --- | --- | --- | --- | --- |
|  | **Y_AA^a^** | **Y_Codon^b^** |  | **Y_AA** | **Y_Codon** |
| *O.sativa* | 0.261 | 0.356 |  | 0.072 | 0.068 |
| *A.thaliana* | 0.457 | 0.339 |  | 0.082 | 0.038 |
| *C.elegans* | 0.435 | 0.407 |  | 0.116 | 0.077 |
| *P.tetraurelia* | 0.042 | 0.016 |  | 0.006 | 0.002 |
| *D.melanogaster* | 0.391 | 0.373 |  | 0.105 | 0.124 |
| *B.distachyon* | 0.543 | 0.568 |  | 0.096 | 0.098 |
| *M.musculus* | 0.391 | 0.398 |  | 0.120 | 0.086 |
| *E.siliculosus* | 0.783 | 0.534 |  | 0.421 | 0.209 |
| *D.rerio* | 0.457 | 0.602 |  | 0.131 | 0.132 |
| *S.scrofa* | 0.565 | 0.559 |  | 0.175 | 0.116 |
| *D.discoideum* | 0.065 | 0.102 |  | 0.058 | 0.056 |
| *H.sapiens* | 0.217 | 0.237 |  | 0.063 | 0.054 |
| *T.rubripes* | 0.500 | 0.636 |  | 0.140 | 0.174 |
| *C.neoformans* | 0.609 | 0.551 |  | 0.312 | 0.173 |
| *G.gallus* | 0.283 | 0.339 |  | 0.059 | 0.048 |
| *A.gambiae* | 0.109 | 0.432 |  | 0.066 | 0.243 |
| *I.tridecemlineatus* | 0.391 | 0.347 |  | 0.136 | 0.070 |
| *P.falciparum* | 0.348 | 0.178 |  | 0.198 | 0.107 |
| *X.tropicalis* | 0.087 | 0.068 |  | 0.030 | 0.017 |
| *P.troglodytes* | 0.261 | 0.331 |  | 0.073 | 0.067 |
| *S.pombe* | 0.000 | 0.000 |  | 0.003 | 0.000 |
| *A.carolinensis* | 0.196 | 0.254 |  | 0.044 | 0.040 |
| *C.jacchus* | 0.065 | 0.008 |  | 0.033 | 0.010 |
| *G.gorilla* | 0.065 | 0.025 |  | 0.047 | 0.019 |
| *M.mulatta* | 0.196 | 0.093 |  | 0.112 | 0.053 |
| *M.gallopavo* | 0.022 | 0.034 |  | 0.029 | 0.013 |
| *P.abelii* | 0.022 | 0.042 |  | 0.034 | 0.025 |
| *S.cerevisiae* | 0.000 | 0.000 |  | 0.000 | 0.000 |
| *O.latipes* | 0.043 | 0.025 |  | 0.039 | 0.028 |
| *S.purpuratus* | 0.022 | 0.025 |  | 0.018 | 0.036 |
| ^a^Proportion of amino acids showing significant trends  ^b^Proportion of codons showing significant trends | | | | | |

**Supplementary table 7.3. Splice-related Genomic Traits**

| Species | X^a^ | N^b^ | M^c^ |
| --- | --- | --- | --- |
| *O.sativa* | 0.514 | 3.773 | 462.862 |
| *A.thaliana* | 0.615 | 4.694 | 184.868 |
| *C.elegans* | 0.545 | 4.608 | 317.982 |
| *P.tetraurelia* | 0.946 | 2.317 | 24.999 |
| *D.melanogaster* | 0.585 | 2.804 | 1003.051 |
| *B.distachyon* | 0.462 | 4.411 | 538.626 |
| *M.musculus* | 0.114 | 6.010 | 6257.081 |
| *E.siliculosus* | 0.302 | 4.945 | 716.683 |
| *D.rerio* | 0.152 | 6.109 | 3223.267 |
| *S.scrofa* | 0.170 | 5.684 | 3082.724 |
| *D.discoideum* | 0.817 | 2.093 | 140.329 |
| *H.sapiens* | 0.099 | 6.115 | 6950.522 |
| *T.rubripes* | 0.349 | 6.465 | 618.061 |
| *C.neoformans* | 0.794 | 4.144 | 71.380 |
| *G.gallus* | 0.172 | 6.467 | 2607.353 |
| *A.gambiae* | 0.555 | 3.176 | 1053.054 |
| *I.tridecemlineatus* | 0.194 | 5.655 | 2295.826 |
| *P.falciparum* | 0.742 | 2.828 | 194.802 |
| *X.tropicalis* | 0.176 | 5.910 | 1541.562 |
| *P.troglodytes* | 0.156 | 5.751 | 3790.599 |
| *S.pombe* | 0.846 | 2.463 | 91.307 |
| *A.carolinensis* | 0.130 | 5.847 | 2676.540 |
| *C.jacchus* | 0.230 | 5.852 | 2244.667 |
| *G.gorilla* | 0.325 | 5.083 | 1184.249 |
| *M.mulatta* | 0.239 | 5.590 | 1532.298 |
| *M.gallopavo* | 0.214 | 5.767 | 1329.885 |
| *P.abelii* | 0.189 | 6.089 | 1884.371 |
| *S.cerevisiae* | 0.768 | 1.766 | 201.503 |
| *O.latipes* | 0.448 | 4.758 | 596.522 |
| *S.purpuratus* | 0.232 | 4.131 | 2616.989 |
| ^a^mean CDS length/gene length  ^b^introns per kb exon  ^c^mean intron size | | | |

**Supplementary table 7.4. 10 orthologous genes used for a multiple sequence alignment.**

| ID | Official Symbol | Official Full Name |
| --- | --- | --- |
|  | EEF2 | Eukaryotic translation elongation factor 2 |
|  | HSP70 | Heat shock 70kDa protein |
|  | WDR1 | WD repeat domain 1 |
|  | CAP | RNA guanylyltransferase and 5'-phosphatase |
|  | RNA polymerase III | RNA polymerase III |
|  | P450 | Cytochrome P450 |
|  | DARS2 | Aspartyl-tRNA synthetase 2 |
|  | CSNK2A1 | Casein kinase 2, alpha 1 polypeptide |
|  | MTFMT | Mitochondrial methionyl-tRNA formyltransferase |
|  | COX3 | Cytochrome C oxidase subunit III |
