# Supplementary material for: Why Selection Might Be Stronger When Populations Are Small: Intron Size and Density Predict within and between-Species Usage of Exonic Splice Associated cis-Motifs
Source: Mol Biol Evol. 2015 Mar 13;32(7):1847–61. doi: 10.1093/molbev/msv069 (PMC4476162; doi:10.1093/molbev/msv069)
Supplement: Supplementary Data [file supp_msv069_suppl_data.zip › Supplementary_Figure_S1-S3.docx]

**Supplementary Fig. S1.**  Comparison of *N*_e_*.μ* values of this study and that from Lynch’s study. a-c reflect correlation between Lynch and Conery’s estimates of *N*_e_*.μ*. d-f represent correlations between our three different estimators (S, Eta and Pi).

**Supplementary Fig. S2.** Comparison of the branch length estimates from the two models of protein evolution as implemented in MEGA 6

**Supplementary Fig. S3. Phylogenetic Tree of 30 species With Branch Length**
