# Supplementary material for: Why Selection Might Be Stronger When Populations Are Small: Intron Size and Density Predict within and between-Species Usage of Exonic Splice Associated cis-Motifs
Source: Mol Biol Evol. 2015 Mar 13;32(7):1847–61. doi: 10.1093/molbev/msv069 (PMC4476162; doi:10.1093/molbev/msv069)
Supplement: Supplementary Data [file supp_msv069_suppl_data.zip › supplementary_material_S1.htm]

10gene\_ori.fas


## Gblocks 0.91b Results

Processed file: **10gene\_ori.fas**  
Number of sequences: **30**  
Alignment assumed to be: **Protein**  
New number of positions: **1055** (selected positions are underlined in blue)

```
                         10        20        30        40        50        60
                 =========+=========+=========+=========+=========+=========+
A.carolinensis   M-----------------------------------------------------------
A.gambiae        M-----------------------------------------------------------
A.thaliana       ------------------------------------------------------------
B.distachyon     M-----------------------------------------------------------
C.elegans        M-----------------------------------------------------------
C.jacchus        M-----------------------------------------------------------
C.neoformans     ------------------------------------------------------------
P.tetraurelia    M-----------------------------------------------------------
D.rerio          M-----------------------------------------------------------
D.discoideum     M-----------------------------------------------------------
D.melanogaster   M-----------------------------------------------------------
E.siliculosus    M-----------------------------------------------------------
G.gallus         ------------------------------------------------------------
G.gorilla        M-----------------------------------------------------------
H.sapiens        M-----------------------------------------------------------
I.tridecemlinea  M-----------------------------------------------------------
M.mulatta        M-----------------------------------------------------------
M.gallopavo      MGPGDSMLQLWESCVALQLMATEPQRPAAPMRRKKQQRSTRGVEMESDVPPQPCLDPLFA
M.musculus       M-----------------------------------------------------------
O.sativa         M-----------------------------------------------------------
O.latipes        M-----------------------------------------------------------
P.troglodytes    M-----------------------------------------------------------
P.falciparum     ------------------------------------------------------------
P.abelii         M-----------------------------------------------------------
S.cerevisiae     M-----------------------------------------------------------
S.pombe          M-----------------------------------------------------------
S.purpuratus     M-----------------------------------------------------------
S.scrofa         M-----------------------------------------------------------
T.rubripes       M-----------------------------------------------------------
X.tropicalis     M-----------------------------------------------------------
                                                                             


                         70        80        90       100       110       120
                 =========+=========+=========+=========+=========+=========+
A.carolinensis   -------------------------------------------------VNFTVDQIRAI
A.gambiae        -------------------------------------------------VNFTVDEIRAM
A.thaliana       ------------------------------------------------------------
B.distachyon     -------------------------------------------------VKFTAEELRRI
C.elegans        -------------------------------------------------VNFTVDEIRAL
C.jacchus        -------------------------------------------------VNFTVDQIRAI
C.neoformans     ------------------------------------------------------------
P.tetraurelia    -------------------------------------------------VNFSVDQVREI
D.rerio          -------------------------------------------------VNFTVDQIREI
D.discoideum     -------------------------------------------------VNFTIDQIRAI
D.melanogaster   -------------------------------------------------VNFTVDEIRGL
E.siliculosus    -------------------------------------------------VNFTTDQLREI
G.gallus         ------------------------------------------------MVNFTVDQIRAI
G.gorilla        -------------------------------------------------VNFTVDQIRAI
H.sapiens        -------------------------------------------------VNFTVDQIRAI
I.tridecemlinea  -------------------------------------------------VNFTVDQIRAI
M.mulatta        -------------------------------------------------VNFTVDQIRAI
M.gallopavo      RLRWAPAAGAAPRSAPGCTPCCGDPPHPGLLRAAPGLRIFLSSLLLFSKVNFTVDQIRAI
M.musculus       -------------------------------------------------VNFTVDQIRAI
O.sativa         -------------------------------------------------VKFTVEELRRI
O.latipes        -------------------------------------------------VNFTVDQIRAI
P.troglodytes    -------------------------------------------------VNFTVDQIRAI
P.falciparum     ------------------------------------------------------------
P.abelii         -------------------------------------------------VNFTVDQIRAI
S.cerevisiae     -------------------------------------------------VAFTVDQMRSL
S.pombe          -------------------------------------------------VAFTPEEVRNL
S.purpuratus     -------------------------------------------------VNFTTDQIRAI
S.scrofa         -------------------------------------------------VNFTVDQIRAI
T.rubripes       -------------------------------------------------VNFTVDQIRAI
X.tropicalis     -------------------------------------------------VNFTVDQIRAI
                                                                             


                        130       140       150       160       170       180
                 =========+=========+=========+=========+=========+=========+
A.carolinensis   MDKKSNIRNMSVIAHVDHGKSTLTDSLVCKAGIIASARAGETRFTDTRKDEQERCITIKS
A.gambiae        MDKKRNIRNMSVIAHVDHGKSTLTDSLVSKAGIIAGAKAGETRFTDTRKDEQERCITIKS
A.thaliana       ---------MSVIAHVDHGKSTLTDSLVAAAGIIAQETAGDVRMTDTRADEAERGITIKS
B.distachyon     MDKKNNIRNMSVIAHVDHGKSTLTDSLVAAAGIIAQEVAGDVRMTDTRADEAERGITIKS
C.elegans        MDRKRNIRNMSVIAHVDHGKSTLTDSLVSKAGIIAGSKAGETRFTDTRKDEQERCITIKS
C.jacchus        MDKKANIRNMSVIAHVDHGKSTLTDSLVCKAGIIASARAGETRFTDTRKDEQERCITIKS
C.neoformans     MDKPTNIRNMSVIAHVDHGKSTLTDSLVSKAGIIASSKAGEMRFTDTRQDEIDRGITIKS
P.tetraurelia    MEKRHNIRNISIIAHVDHGKSTLTDSLVAAAGIISLDSAGNARLTDTRPDEQERGITIKS
D.rerio          MDKKSNIRNMSVIAHVDHGKSTLTDSLVCKAGIIASARAGETRFTDTRKDEQERCITIKS
D.discoideum     MDRRENIRNMSVIAHVDHGKTTLSDSLIQRAGIIADKVSGDMRYMSCRADEQERGITIKS
D.melanogaster   MDKKRNIRNMSVIAHVDHGKSTLTDSLVSKAGIIAGAKAGETRFTDTRKDEQERCITIKS
E.siliculosus    MDKKHNIRNMSVIAHVDHGKSTLTDSLVSKAGIIAAKNAGEARFTDTRQDEQDRCITIKS
G.gallus         MDKKANIRNMSVIAHVDHGKSTLTDSLVCKAGIIASARAGETRFTDTRKDEQERCITIKS
G.gorilla        MDKKANIRNMSVIAHVDHGKSTLTDSLVCKAGIIASARAGETRFTDTRKDEQERCITIKS
H.sapiens        MDKKANIRNMSVIAHVDHGKSTLTDSLVCKAGIIASARAGETRFTDTRKDEQERCITIKS
I.tridecemlinea  MDKKANIRNMSVIAHVDHGKSTLTDSLVCKAGIIASARAGETRFTDTRKDEQERCITIKS
M.mulatta        MDKKANIRNMSVIAHVDHGKSTLTDSLVCKAGIIASARAGETRFTDTRKDEQERCITIKS
M.gallopavo      MDKKANIRNMSVIAHVDHGKSTLTDSLVCKAGIIASARAGETRFTDTRKDEQERCITIKS
M.musculus       MDKKANIRNMSVIAHVDHGKSTLTDSLVCKAGIIASARAGETRFTDTRKDEQERCITIKS
O.sativa         MDKKNNIRNMSVIAHVDHGKSTLTDSLVAAAGIIAQEVAGDVRMTDTRADEAERGITIKS
O.latipes        MDKKSNIRNMSVIAHVDHGKSTLTDSLVSKAGIIASARAGETRFTDTRKDEQERCITIKS
P.troglodytes    MDKKANIRNMSVIAHVDHGKSTLTDSLVCKAGIIASARAGETRFTDTRKDEQERCITIKS
P.falciparum     -----------------HGKSTLTDSLVSKAGIISSKNAGDARFTDTRQDEQERCITIKS
P.abelii         MDKKANIRNMSVIAHVDHGKSTLTDSLVCKAGIIASARAGETRFTDTRKDEQERCITIKS
S.cerevisiae     MDKVTNVRNMSVIAHVDHGKSTLTDSLVQRAGIISAAKAGEARFTDTRKDEQERGITIKS
S.pombe          MGKPSNVRNMSVIAHVDHGKSTLTDSLVQKAGIISAAKAGDARFMDTRADEQERGVTIKS
S.purpuratus     MDKKTNIRNMSVIAHVDHGKSTLTDSLVSKAGIIAQSKAGEARFTDTRKDEQERCITIKS
S.scrofa         MDKKANIRNMSVIAHVDHGKSTLTDSLVCKAGIIASARAGETRFTDTRKDEQERCITIKS
T.rubripes       MDKKANIRNMSVIAHVDHGKSTLTDSLVSKAGIIASARAGETRFTDTRKDEQERCITIKS
X.tropicalis     MDKKSNIRNMSVIAHVDHGKSTLTDSLVCKAGIIASARAGETRFTDTRKDEQERCITIKS
                                  ###########################################


                        190       200       210       220       230       240
                 =========+=========+=========+=========+=========+=========+
A.carolinensis   TAISLYYELSENDLAFIK------------------------QS----------------
A.gambiae        TAISMYFELDEKDLVFITN------------PDQ--------RD----------------
A.thaliana       TGISLYYEMTDASLKSFT------------------------GA----------------
B.distachyon     TGISLFYEMTDESLQMYK------------------------GE----------------
C.elegans        TAISLFFELEKKDLEFVKG------------ENQFETVEVDGKK----------------
C.jacchus        TAISLFYELSENDLNFIK------------------------QS----------------
C.neoformans     TAISMYFPLDKDDVAEIK------------------------QK----------------
P.tetraurelia    TGISLFFEIQEDFLL--P------------------------KE----------------
D.rerio          TAISLYYELSENDSAFIK------------------------QC----------------
D.discoideum     SSVSLHFEMPKEDKLPAG------------------------CT----------------
D.melanogaster   TAISMYFEVEEKDLVFITH------------PDQ--------RE----------------
E.siliculosus    TGISMFFEYNLDAGEKVARQELEAKASKSAGESAEDAKV-AAEAAAAAGEANDTPKADHV
G.gallus         TAISLFYELSENDLAFIK------------------------QS----------------
G.gorilla        TAISLFYELSENDLNFIK------------------------QS----------------
H.sapiens        TAISLFYELSENDLNFIK------------------------QS----------------
I.tridecemlinea  TAISLFYELSENDLNFIK------------------------QS----------------
M.mulatta        TAISLFYELSENDLNFIK------------------------QS----------------
M.gallopavo      TAISLFYELSENDLAFIK------------------------QS----------------
M.musculus       TAISLFYELSENDLNFIK------------------------QS----------------
O.sativa         TGISLFYEMSDESLKLYK------------------------GE----------------
O.latipes        TAISMYYELGDNDLAFIK------------------------QS----------------
P.troglodytes    TAISLFYELSENDLNFIK------------------------QS----------------
P.falciparum     TGISMYFEHDLEDGE---------------------------------------------
P.abelii         TAISLFYELSENDLNFIK------------------------QS----------------
S.cerevisiae     TAISLYSEMSDEDVKEIK------------------------QK----------------
S.pombe          TAISLFAEMTDDDMKDMK------------------------EP----------------
S.purpuratus     TAISMYYELSDKDMTFIE------------------------QE---------------K
S.scrofa         TAISLFYELSENDLNFIK------------------------QS----------------
T.rubripes       TAISLFYELAENDLAFIK------------------------QD----------------
X.tropicalis     TAISLYYELSENDLAFIK------------------------QC----------------
                 #############                                               


                        250       260       270       280       290       300
                 =========+=========+=========+=========+=========+=========+
A.carolinensis   -KDGSGFLINLIDSPGHVDFSSEVTAALRVTDGALVVVDCVSGVCVQTETVLRQAIAERI
A.gambiae        -KDCKGFLINLIDSPGHVDFSSEVTAALRVTDGALVVVDCVSGVCVQTETVLRQAIAERI
A.thaliana       -RDGNEYLINLIDSPGHVDFSSEVTAALRITDGALVVVDCIEGVCVQTETVLRQSLGERI
B.distachyon     -RDGNEYLINLIDSPGHVDFSSEVTAALRITDGALVVVDCIEGVCVQTETVLRQALGERI
C.elegans        -EKYNGFLINLIDSPGHVDFSSEVTAALGVTDGALVVVDCVSGVCVQTETVLRQAIAERI
C.jacchus        -KDGAGFLINLIDSPGHVDFSSEVTAALRVTDGALVVVDCVSGVCVQTETVLRQAIAERI
C.neoformans     -TDGNEFLINLIDSPGHVDFSSEVTAALRVTDGALVVVDCVEGVCVQTETVLRQSLGERV
P.tetraurelia    -INGNKFLINLIDSPGHVDFSSEVTAALRVTDGALVIIDCIEGVCVQTETVLRQALSERI
D.rerio          -KDGSGFLINLIDSPGHVDFSSEVTAALRVTDGALVVVDCVSGVCVQTETVLRQAIAERI
D.discoideum     ---SHEFLINLIDSPGHVDFSSEVTAALRVTDGALVVIDCVEGVCVQTETVLRQAVAERI
D.melanogaster   -KECKGFLINLIDSPGHVDFSSEVTAALRVTDGALVVVDCVSGVCVQTETVLRQAIAERI
E.siliculosus    QIDETSFLINLIDSPGHVDFSSEVTAALRVTDGALVVVDCVEGVCVQTETVLRQAIGERV
G.gallus         -KDGSGFLINLIDSPGHVDFSSEVTAALRVTDGALVVVDCVSGVCVQTETVLRQAIAERI
G.gorilla        -KDGAGFLINLIDSPGHVDFSSEVTAALRVTDGALVVVDCVSGVCVQTETVLRQAIAERI
H.sapiens        -KDGAGFLINLIDSPGHVDFSSEVTAALRVTDGALVVVDCVSGVCVQTETVLRQAIAERI
I.tridecemlinea  -KDGSGFLINLIDSPGHVDFSSEVTAALRVTDGALVVVDCVSGVCVQTETVLRQAIAERI
M.mulatta        -KDGAGFLINLIDSPGHVDFSSEVTAALRVTDGALVVVDCVSGVCVQTETVLRQAIAERI
M.gallopavo      -KDGSGFLINLIDSPGHVDFSSEVTAALRVTDGALVVVDCVSGVCVQTETVLRQAIAERI
M.musculus       -KDGSGFLINLIDSPGHVDFSSEVTAALRVTDGALVVVDCVSGVCVQTETVLRQAIAERI
O.sativa         -RDGNEYLINLIDSPGHVDFSSEVTAALRITDGALVVVDCIEGVCVQTETVLRQALGERI
O.latipes        -KDGNGFLINLIDSPGHVDFSSEVTAALRVTDGALVVVDCVSGVCVQTETVLRQAIAERI
P.troglodytes    -KDGAGFLINLIDSPGHVDFSSEVTAALRVTDGALVVVDCVSGVCVQTETVLRQAIAERI
P.falciparum     --GKKPFLINLIDSPGHVDFSSEVTAALRVTDGALVVVDTIEGVCVQTETVLYQALGERI
P.abelii         -KDGAGFLINLIDSPGHVDFSSEVTAALRVTDGALVVVDCVSGVCVQTETVLRQAIAERI
S.cerevisiae     -TDGNSFLINLIDSPGHVDFSSEVTAALRVTDGALVVVDTIEGVCVQTETVLRQALGERI
S.pombe          -ADGTDFLVNLIDSPGHVDFSSEVTAALRVTDGALVVVDTIEGVCVQTETVLRQALGERI
S.purpuratus     DVNERGFLINLIDSPGHVDFSSEVTAALRVTDGALVVVDCVSGVCVQTETVLRQAIAERI
S.scrofa         -KDGSGFLINLIDSPGHVDFSSEVTAALRVTDGALVVVDCVSGVCVQTETVLRQAIAERI
T.rubripes       -KDGSGFLINLIDSPGHVDFSSEVTAALRVTDGALVVVDCVSGVCVQTETVLRQAIGERI
X.tropicalis     -KEGSGFLINLIDSPGHVDFSSEVTAALRVTDGALVVVDCVSGVCVQTETVLRQAIAERI
                       ######################################################


                        310       320       330       340       350       360
                 =========+=========+=========+=========+=========+=========+
A.carolinensis   KPVLMMNKMDRALLELQLDREELYQTFQRIVENVNVIISTYGEGETGPMGNIMIDPVIGT
A.gambiae        KPVLFMNKMDRALLELQLDPEDLYQTFQRIVENVNVIIATYND-DGGPMGEVRIDPSRGS
A.thaliana       RPVLTVNKMDRCFLELKVDGEEAYQNFQRVIENANVIMATHED---PLLGDVQVYPEKGT
B.distachyon     RPVLTVNKMDRCFLELQVEGEEAYQTFSRVIENANVIMATYED---KLLGDVQVYPEKGT
C.elegans        KPVLFMNKMDRALLELQLGAEELFQTFQRIVENINVIIATYGD-DDGPMGPIMVDPSIGN
C.jacchus        KPVLMMNKMDRALLELQLEPEELYQTFQRIVENVNVIISTYGEGESGPMGNIMIDPVLGT
C.neoformans     KPILIINKVDRALLELQVSKEDLYQSFCRTIESVNVIISTYTD---PALGDSMVYPEQGT
P.tetraurelia    KPVVVVNKLDRGFLELQADAESMYRNFSRVVENINVLIATYRD---DVFGEMQVYPEQNT
D.rerio          KPVLMMNKMDRALLELQLEPDELFQTFQRIVENVNVIISTYGEGEHGPMGNIMVDPVIGT
D.discoideum     KPVLFVNKVDRFLLELQLNTEEAYLSFRRAIESVNVIVGNTED---KEFGDVTVSPEKGT
D.melanogaster   KPILFMNKMDRALLELQLDAEELYQTFQRIVENVNVIIATYND-DGGPMGEVRVDPSKGS
E.siliculosus    RPVLMVNKVDRALLELHLPPEEMYQSFARAIESVNVIIATYND---ELLGDVQVYPDKGT
G.gallus         KPVLMMNKMDRALLELQLDPEELYQTFQRIVENVNVIISTYGEGESGPMGNIMIDPVLGT
G.gorilla        KPVLMMNKMDRALLELQLEPEELYQTFQRIVENVNVIISTYGEGESGPMGNIMIDPVLGT
H.sapiens        KPVLMMNKMDRALLELQLEPEELYQTFQRIVENVNVIISTYGEGESGPMGNIMIDPVLGT
I.tridecemlinea  KPVLMMNKMDRALLELQLEPEELYQTFQRIVENVNVIISTYGEGESGPMGNIMIDPVLGT
M.mulatta        KPVLMMNKMDRALLELQLEPEELYQTFQRIVENVNVIISTYGEGESGPMGNIMIDPVLGT
M.gallopavo      KPVLMMNKMDRALLELQLDPEELYQTFQRIVENVNVIISTYGEGESGPMGNIMIDPVLGT
M.musculus       KPVLMMNKMDRALLELQLEPEELYQTFQRIVENVNVIISTYGEGESGPMGNIMIDPVLGT
O.sativa         RPVLTVNKMDRCFLELQVEGEEAYQTFSRVIENANVIMATYED---TLLGDVQVYPEKGT
O.latipes        KPVLMMNKMDRALLELQLEPDELYQTFQRIVENVNVIISTYGEDESGPMGSIMIDPVIGT
P.troglodytes    KPVLMMNKMDRALLELQLEPEELYQTFQRIVENVNVIISTYGEGESGPMGNIMIDPVLGT
P.falciparum     KPVLHVNKVDRALLELQMEVEDIYQTFARTIESVNVIISTYTD---KLMGDIQVYPEKGT
P.abelii         KPVLMMNKMDRALLELQLEPEELYQTFQRIVENVNVIISTYGEGESGPMGNIMIDPVLGT
S.cerevisiae     KPVVVINKVDRALLELQVSKEDLYQTFARTVESVNVIVSTYAD---EVLGDVQVYPARGT
S.pombe          RPVVVVNKVDRALLELQISQEELYQNFARVVESVNVVISTYYD---KVLGDCQVFPDKGT
S.purpuratus     KPVVFMNKMDRALLELQLEMEDLYQTFQRIVESINVIVATYAD-EDGPMGNIQVAPSRGT
S.scrofa         KPVLMMNKMDRALLELQLEPEELYQTFQRIVENVNVIISTYGEGESGPMGNIMIDPVLGT
T.rubripes       KPVLMMNKMDRALLELQLEPEDLYQTFQRIVESVNVIICTYGEVETGPMGNVMVEPVCGT
X.tropicalis     KPVLMMNKMDRALLELQLEPEELYLTFQRIVENVNVIISTYGEGESGPMGNIMIDPVIGT
                 ###########################################   ##############


                        370       380       390       400       410       420
                 =========+=========+=========+=========+=========+=========+
A.carolinensis   VGFGSGLHGWAFTLKQFAEMYVAKFAAKGEK--AQPSAAERAKKVEDMMKKLWGDKYFDP
A.gambiae        VGFGSGLHGWAFTLKQFAEMYSAMFKI----------------DVVKLMNRLWGENFFNS
A.thaliana       VAFSAGLHGWAFTLTNFAKMYASKFGV----------------SESKMMERLWGENFFDS
B.distachyon     VAFSAGLHGWAFTLTNFAKMYASKFGV----------------DESKMMERLWGENFFDP
C.elegans        VGFGSGLHGWAFTLKQFAEMYAGKFGV----------------QVDKLMKNLWGDRFFDL
C.jacchus        VGFGSGLHGWAFTLKQFAEMYVAKFAAKGE---GQLGPAERAKKVEDMMKKLWGDRYFDP
C.neoformans     VAFGSGLHGWAFSLRNFAGRYSKKFGV----------------DKAKLMPKLWGDNYFNP
P.tetraurelia    VAFSAGLHGWAFTLGQFARMYAKKWKIEKE---------KKLDFIEKLTSRLWGDNFFDI
D.rerio          VGFGSGLHGWAFTLKQFAEMYVAKFAAKGDKKKGDLPPAERAKKVEEMMKKLWGDKYFDP
D.discoideum     VAFGSGLHGWGFTLGRFAKLYAAKFGD----------------PEDKLMGRLWGDSYFDA
D.melanogaster   VGFGSGLHGWAFTLKQFSEMYSEKFKI----------------DVVKLMNRLWGENFFNA
E.siliculosus    VAFGSGLHQWGFTLKKFAKIYAAKFGT----------------QEEKMMQKLWGDWYFDA
G.gallus         VGFGSGLHGWAFTLKQFAEMYVAKFAAKGD---AQMNPTERAKKVEDMMKKLWGDRYFDP
G.gorilla        VGFGSGLHGWAFTLKQFAEMYVAKFAAKGE---GQLGPAERAKKVEDMMKKLWGDRYFDP
H.sapiens        VGFGSGLHGWAFTLKQFAEMYVAKFAAKGE---GQLGPAERAKKVEDMMKKLWGDRYFDP
I.tridecemlinea  VGFGSGLHGWAFTLKQFAEMYVAKFAAKGE---GQLGPAERAKKVEDMMKKLWGDRYFDP
M.mulatta        VGFGSGLHGWAFTLKQFAEMYVAKFAAKGE---GQLGPAERAKKVEDMMKKLWGDRYFDP
M.gallopavo      VGFGSGLHGWAFTLKQFAEMYVAKFAAKGD---AQMNPSERAKKVEDMMKKLWGDRYFDP
M.musculus       VGFGSGLHGWAFTLKQFAEMYVAKFAAKGE---GQLSAAERAKKVEDMMKKLWGDRYFDP
O.sativa         VAFSAGLHGWAFTLSSFAKMYASKFGV----------------DEFKMMERLWGENFFDP
O.latipes        VGFGSGLHGWAFTLKQFAEMYVAKFTAKGV---AQLGPAERCKKVEDMMKKLWGDRYFDP
P.troglodytes    VGFGSGLHGWAFTLKQFAEMYVAKFAAKGE---GQLGPAERAKKVEDMMKKLWGDRYFDP
P.falciparum     VSFGSGLQGWAFTLETFSRIYSKKFGI----------------EKKKMMQRLWGNSFYDA
P.abelii         VGFGSGLHGWAFTLKQFAEMYAAKFAAKGE---GQLGPAERAKKVEDMMKKLWGDRYFDP
S.cerevisiae     VAFGSGLHGWAFTIRQFATRYAKKFGV----------------DKAKMMDRLWGDSFFNP
S.pombe          VAFASGLHGWAFTVRQFANRYAKKFGI----------------DRNKMMQRLWGENYFNP
S.purpuratus     VGFGSGLHGWAFTLKQFAEIYASKFKI----------------EPAKLMKRLWGDQFFNP
S.scrofa         VGFGSGLHGWAFTLKQFAEMYVAKFAAKGE---GQLGPAERAKKVEDMMKKLWGDRYFDP
T.rubripes       VGFGSGLHGWAFTLKQFAEMYTSKMLAKGA---DKMTATERCQKVEDMMKKLWGDRYYDA
X.tropicalis     VGFGSGLHGWAFTLKQFAEMYVAKFASKGEK--TKLNPADRAKKVEDMMKKLWGDKYFDP
                 #########################                      #############


                        430       440       450       460       470       480
                 =========+=========+=========+=========+=========+=========+
A.carolinensis   ANGKFSKTANSADGKKLPRTFCQLILDPIFKVFDAIMNFKKEEASKLIEKLDIKLDAEDR
A.gambiae        KTKKWAKVKDDDN----KRSFVMYILDPIYKVFDAIMNYKTDEIPKLLEKIKVSLKHEDK
A.thaliana       ATRKWTTK-TGSPT--CKRGFVQFCYEPIKIMINTCMNDQKDKLWPMLEKLGIQMKPDEK
B.distachyon     TTKKWTTKNTGSAT--CKRGFVQFCYEPIKQIINTCMNDQKDKLWPMLKKLGVTMKNDEK
C.elegans        KTKKWSSTQTDES----KRGFCQFVLDPIFMVFDAVMNIKKDKTAALVEKLGIKLANDEK
C.jacchus        ATGKFSKSASSPDGKKLPRTFCQLILDPIFKVFDAIMNFKKEETAKLIEKLDIKLDSEDK
C.neoformans     KTRKWTKSADAG----VERAFNMFVLDPIFRLFDSIMNFKKDEIPKLLEKLEIKLTSEER
P.tetraurelia    NSKRWIKRSKQEH----PRAFCHFIINPIKKIIEFSMADKIEELEHILSTFDIKLNSEDK
D.rerio          SCGKFSKTANNADGKKLPRTFCQLVLDPIFKVFDAIMNFKKEETQKLIEKLEVKLDAEDK
D.discoideum     TAKKWTSNPQSADGKALPRAFCQFVLEPIYQLTRAIVDEDAVKLEKMMKTLQITLAPEDA
D.melanogaster   KTKKWQKQKEADN----KRSFCMYILDPIYKVFDAIMNYKKEEIGTLLEKIGVTLKHEDK
E.siliculosus    AGKKWKKNSDNGK---LERAFCQWIMSPICKMFDAIMDDKKQKIQKMLTAVGVTLKGEEK
G.gallus         ATGKFSKSATGPDGKKLPRTFCQLILDPIFKVFDAIMTFKKEEAAKLIEKLDIKLDSEDK
G.gorilla        ANGKFSKSATSPEGKKLPRTFCQLILDPIFKVFDAIMNFKKEETAKLIEKLDIKLDSEDK
H.sapiens        ANGKFSKSATSPEGKKLPRTFCQLILDPIFKVFDAIMNFKKEETAKLIEKLDIKLDSEDK
I.tridecemlinea  ANGKFSKSATSPDGKKLPRTFCQLILDPIFKVFDAIMNFKKEETAKLIEKLDIKLDSEDK
M.mulatta        ANGKFSKSATSPDGKKLPRTFCQLILDPIFKVFDAIMNFKKEETAKLIEKLDIKLDSEDK
M.gallopavo      ATGKFSKSATGPDGKKLPRTFCQLILDPIFKVFDAIMTFKKEEAAKLIEKLDIKLDSEDK
M.musculus       ANGKFSKSANSPDGKKLPRTFCQLILDPIFKVFDAIMNFRKEETAKLIEKLDIKLDSEDK
O.sativa         ATKKWTNKNTGSAT--CKRGFVQFCYEPIKQIINTCMNDQKDKLWPMLQKLGVVMKADEK
O.latipes        SAGKFSKTATGPDGQKFPRTFSQLVLDPIFKVFDAIMNFRKEETAKLIDKLDVKLDSEDK
P.troglodytes    ANGKFSKSATSPEGKKLPRTFCQLILDPIFKVFDAIMNFKKEETAKLIEKLDIKRXXXDR
P.falciparum     KTKKWSKNQQEG----YKRGFCQFIMEPILNLCQSIMNDDKEKYTKMLTNIGVELKGDDK
P.abelii         ANGKFSKSATSPEGKKLPRTFCQLILDPIFKVFDAIMNFKKEETAKLIEKLDIKLDSEDK
S.cerevisiae     KTKKWTNKDTDAEGKPLERAFNMFILDPIFRLFTAIMNFKKDEIPVLLEKLEIVLKGDEK
S.pombe          KTKKWSKSATDANGNSNQRAFNMFILDPIYRIFDAVMNSRKDEVFTLLSKLEVTIKPDEK
S.purpuratus     KEKKWNKVGGE----GYVKGFNQFVLDPIYKMFDAVMNFKKPETEKLLEKLKVNLKSEEK
S.scrofa         ANGKFSKSATSPDGKKLPRTFCQLILDPIFKVFDAIMNFKKEETAKLIEKLDIKLDSEDK
T.rubripes       KNGKFLKTSTAADGTKLPRTFVALVLDPIFKVFDAIMNFKKEETAKMIQKLDIKLDSEDK
X.tropicalis     STGKFSKTATNAEGKKLPRTFSQLILDPIFKIFDAIMNFKKEETAKLIEKLDIKLDTEDK
                 #######          ###########################################


                        490       500       510       520       530       540
                 =========+=========+=========+=========+=========+=========+
A.carolinensis   EKEGKPLLKAVMRRWLPAGDALLQMITIHLPSPVTAQKYRCELLYEGPPDDEAAMGVKNC
A.gambiae        DKDGKNLLKVVMRTWLPAGEALLQMIAIHLPSPVVAQKYRMEMLYEGPHDDEAAVAVKNC
A.thaliana       ELMGKPLMKRVMQAWLPASTALLEMMIFHLPSPYTAQRYRVENLYEGPLDDKYAAAIRNC
B.distachyon     DLMGKALMKRVMQTWLPASRALLEMMVFHLPSPSKAQRYRVENLYEGPLDDIYATAIRNC
C.elegans        DLEGKPLMKVFMRKWLPAGDTMLQMIAFHLPSPVTAQKYRMEMLYEGPHDDEAAVAIKTC
C.jacchus        DKEGKPLLKAVMRRWLPAGDALLQMITIHLPSPVTAQKYRCELLYEGPPDDEAAMGIKSC
C.neoformans     DLEGKQLLKVVMRKFLPAGDSLLEMICINLPSPVTAQKYRVETLYEGPMDDESAIGIRDC
P.tetraurelia    KLKQKNLMKRTMQKFLSADKALLEMIVLKLPSPAEAQSYRIDNLYQGPLDDFVAQSIKNC
D.rerio          EKEGKPLLKAVMRRWLPAGDALLQMITIHLPSPVTAQRYRCELLYEGPGDDEAAMGIKNC
D.discoideum     EIKGKQLVKAVMRKFLPAADAILSMIVTHLPSPLVAQKYRCANLYEGPMDDECAVAIQKC
D.melanogaster   DKDGKALLKTVMRTWLPAGEALLQMIAIHLPSPVVAQKYRMEMLYEGPHDDEAAIAVKSC
E.siliculosus    ELVGKPLLKRVMQKWLPAADAVLEMIVVHLPSPPQAQKYRVENLYDGPLDDEVANSIRTC
G.gallus         DKEGKPLLKAVMRRWLPAGDALLQMITIHLPSPVTAQKYRCELLYEGPPDDEAAIGIKNC
G.gorilla        DKEGKPLLKAVMRRWLPAGDALLQMITIHLPSPVTAQKYRCELLYEGPPDDEAAMGIKSC
H.sapiens        DKEGKPLLKAVMRRWLPAGDALLQMITIHLPSPVTAQKYRCELLYEGPPDDEAAMGIKSC
I.tridecemlinea  DKEGKPLLKAVMRRWLPAGDALLQMITIHLPSPVTAQKYRCELLYEGPPDDEAAMGIKSC
M.mulatta        DKEGKPLLKAVMRRWLPAGDALLQMITIHLPSPVTAQKYRCELLYEGPPDDEAAMGIKSC
M.gallopavo      DKEGKPLLKAVMRRWLPAGDALLQMITIHLPSPVTAQKYRCELLYEGPPDDEAAIGIKNC
M.musculus       DKEGKPLLKAVMRRWLPAGDALLQMITIHLPSPVTAQKYRCELLYEGPPDDEAAMGIKSC
O.sativa         ELMGKALMKRVMQTWLPASNALLEMMIYHLPSPSKAQRYRVENLYEGPLDDVYATAIRNC
O.latipes        EKEGKPLLKAVMRRWLPAGEALLQMITIHLPSPVTAQKYRCELLYEGPGDDEAAMGIKNC
P.troglodytes    DKEGKPLLKAVMRRWLPAGDALLQMITIHLPSPVTAQKYRCELLYEGPPDDEAAMGIKSC
P.falciparum     LLTGKQLLKKAMQLWLPAGDTLLEMIVTHLPSPADAQKYRVENLYEGPMDDEAANAIRNC
P.abelii         DKEGKPLLKAVMRRWLPAGDALLQMITIHLPSPVTAQKYRCELLYEGPPDDEAAMGIKSC
S.cerevisiae     DLEGKALLKVVMRKFLPAADALLEMIVLHLPSPVTAQAYRAEQLYEGPADDANCIAIKNC
S.pombe          ELEGKALLKVVMRKFLPAADALMEMIVLHLPSPKTAQQYRAETLYEGPMDDECAVGIRNC
S.purpuratus     DLEGKPLIKVIMRNWLPAGETMLQMITIHLPSPATAQKYRMEMLYEGPLDDPVAMGIKTC
S.scrofa         DKEGKPLLKAVMRRWLPAGDALLQMITIHLPSPVTAQKYRCELLYEGPPDDEAAMGIKSC
T.rubripes       DKEGKPLLKAVMRRWLPAGDALLQMITIHLPSPVTAQKYRCEFLYEGPPDDDVAMGIKNC
X.tropicalis     EKEGKPLLKAVMRRWLPAGDALLQMITIHLPSPVTAQKYRCELLYEGPPDDEAALGVKSC
                 ############################################################


                        550       560       570       580       590       600
                 =========+=========+=========+=========+=========+=========+
A.carolinensis   D--PKGPLMMYISKMVPTSDKGRFYAFGRVFSGVVSTGQKVRIMGPNYTPGKKEDLYLKP
A.gambiae        D--PEGPLMMYVSKMVPTSDKGRFYAFGRVFAGKVATGQKCRIMGPNFTPGKKEDLYEKA
A.thaliana       D--PDGPLMLYVSKMIPASDKGRFFAFGRVFSGTVSTGMKVRIMGPNYVPGEKKDLYVKS
B.distachyon     D--PEGPLMLYVSKMIPASDKGRFFAFGRVFAGRVATGMKVRIMGPNYVPGQKKDLYVKS
C.elegans        D--PNGPLMMYISKMVPTSDKGRFYAFGRVFSGKVATGMKARIQGPNYVPGKKEDLYEKT
C.jacchus        D--PKGPLMMYISKMVPTSDKGRFYAFGRVFSGLVSTGLKVRIMGPNYTPGKKEDLYLKP
C.neoformans     D--PKGPLMVYVSKMVPTSDKGRFYAFGRVFSGTVSSGPKVRIQGPNFVPGKKDDSVIKS
P.tetraurelia    D--PQGPLMVYISKMIPSTDKGRFIAFGRVFSGTVKTGQKVRIMGPNYVFGKKNDLAIKN
D.rerio          D--PKAPLMMYISKMVPTTDKGRFYAFGRVFSGIVSTGQKVRIMGPNFTPGKKEDLYLKP
D.discoideum     D--PNGPLMMYVSKMVPTSDKGRFYAFGRVFSGIIVPVKRSELWVSTYVPGKKDDLFLKS
D.melanogaster   D--PDGPLMMYISKMVPTSDKGRFYAFGRVFAGKVATGQKCRIMGPNYTPGKKEDLYEKA
E.siliculosus    DTSPGAPLCMYVSKMVPTSDKGRFYAFGRVFAGTIATGQKVRILGPNYVPGKKSDLWVKN
G.gallus         D--PRGSLMMYISKMVPTSDKGRFYAFGRVFSGLVSTGLKVRIMGPNYTPGKKEDLYLKP
G.gorilla        D--PKGPLMMYISKMVPTSDKGRFYAFGRVFSGLVSTGLKVRIMGPNYTPGKKEDLYLKP
H.sapiens        D--PKGPLMMYISKMVPTSDKGRFYAFGRVFSGLVSTGLKVRIMGPNYTPGKKEDLYLKP
I.tridecemlinea  D--PKGPLMMYISKMVPTSDKGRFYAFGRVFSGLVSTGLKVRIMGPNYTPGKKEDLYLKP
M.mulatta        D--PKGPLMMYISKMVPTSDKGRFYAFGRVFSGLVSTGLKVRIMGPNYTPGKKEDLYLKP
M.gallopavo      D--PKGPLMMYISKMVPTSDKGRFYAFGRVFSGLVSTGLKVRIMGPNYTPGKKEDLYLKP
M.musculus       D--PKGPLMMYISKMVPTSDKGRFYAFGRVFSGVVSTGLKVRIMGPNYTPGKKEDLYLKP
O.sativa         D--PEGPLMLYVSKMIPASDKGRFFAFGRVFSGRVATGMKVRIMGPNYVPGQKKDLYVKS
O.latipes        D--PKAPLMMYISKMVPTTDKGRFYAFGRVFSGCVSTGQKVRIMGPNFTPGKKEDLYIKP
P.troglodytes    D--PKGPLMMYISKMVPTSDKGRFYAFGRVFSGLVSTGLKVRIMGPNYTPGKKEDLYLKP
P.falciparum     D--PNGPLMMYISKMVPTSDKGRFYAFGRVFSGTVATGQKVRIQGPHYVPGEKTDLYEKN
P.abelii         D--PKGPLMMYISKMVPTSDKGRFYAFGRVFSGLVSTGLKVRIMGPNYTPGKKEDLYLKP
S.cerevisiae     D--PKADLMLYVSKMVPTSDKGRFYAFGRVFAGTVKSGQKVRIQGPNYVPGKKDDLFIKA
S.pombe          D--ANAPLMIYVSKMVPTSDRGRFYAFGRVFSGTVRSGLKVRIQGPNYVPGKKDDLFIKA
S.purpuratus     D--PKAPLCMYVSKMVPTTDKGRFFAFGRVFSGTIGTGQKCRIMGPNFIPGKKEDLYLKN
S.scrofa         D--PKGPLMMYISKMVPTSDKGRFYAFGRVFSGLVSTGLKVRIMGPNYTPGKKEDLYLKP
T.rubripes       D--SKAPLMIYISKMVPTSDKGRFYAFGRVFSGSVSTGLKVRIMGPNYVPGKKDDLYTKP
X.tropicalis     D--PKGPLVMYISKMVPTSDKGRFYAFGRVFSGIVSTGQKVRIMGPNYTPGKKEDLYLKP
                 #  #########################################################


                        610       620       630       640       650       660
                 =========+=========+=========+=========+=========+=========+
A.carolinensis   IQRTILMMGRYVEPIEDVPCGNIVGLVGVDQFLVKTGTITT--FEHAHNMRVMKFSVSPV
A.gambiae        IQRTILMMGRYVEAIEDVPCGNICGLVGVDQFLVKTGTIST--FKDAHNMKVMKFSVSPV
A.thaliana       VQRTVIWMGKKQETVEDVPCGNTVAMVGLDQFITKNGTLTNEKEVDAHPLRAMKFSVSPV
B.distachyon     VQRTVIWMGKKQESVEDVPCGNTVALVGLDQFITKNATLTNEKETDACPIRAMKFSVSPV
C.elegans        IQRTILMMGRFIEPIEDIPSGNIAGLVGVDQYLVKGGTITT--YKDAHNMRVMKFSVSPV
C.jacchus        IQRTILMMGRYVEPIEDVPCGNIVGLVGVDQFLVKTGTITT--FEHAHNMRVMKFSVSPV
C.neoformans     IQRTVLMMGRSTEAIEDCPAGNIIGLVGVDQFLLKSGTLTT--SETAHNMRVMKFSVSPV
P.tetraurelia    IQRTLLMMGRKAEIIESVPCGNTVGLVGLDQSIVKSGTITDHE--DAYPFRNMKYSISPV
D.rerio          IQRTILMMGRYVEPIEDVPCGNIVGLVGVDQFLVKTGTITT--FENSHNMRVMKFSVSPV
D.discoideum     IQRTVLMMGRKTEQIEDCPCGNIVGLVGVDQFLVKSGTITT--SEVAHNIRVMKFSVSPV
D.melanogaster   IQRTILMMGRYVEAIEDVPSGNICGLVGVDQFLVKTGTITT--FKDAHNMKVMKFSVSPV
E.siliculosus    IQRTIIMMGRYVEQVQDIPAGNTCGLVGVDQYLLKSGTITTS--DTGHCIKTMKFSVSPV
G.gallus         IQRTILMMGRYVEPIEDVPCGNIVGLVGVDQFLVKTGTITT--FEHAHNMRVMKFSVSPV
G.gorilla        IQRTILMMGRYVEPIEDVPCGNIVGLVGVDQFLVKTGTITT--FEHAHNMRVMKFSVSPV
H.sapiens        IQRTILMMGRYVEPIEDVPCGNIVGLVGVDQFLVKTGTITT--FEHAHNMRVMKFSVSPV
I.tridecemlinea  IQRTILMMGRYVEPIEDVPCGNIVGLVGVDQFLVKTGTITT--FEHAHNMRVMKFSVSPV
M.mulatta        IQRTILMMGRYVEPIEDVPCGNIVGLVGVDQFLVKTGTITT--FEHAHNMRVMKFSVSPV
M.gallopavo      IQRTILMMGRYVEPIEDVPCGNIVGLVGVDQFLVKTGTITT--FEHAHNMRVMKFSVSPV
M.musculus       IQRTILMMGRYVEPIEDVPCGNIVGLVGVDQFLVKTGTITT--FEHAHNMRVMKFSVSPV
O.sativa         VQRTVIWMGKKQESVEDVPCGNTVAMVGLDQFITKNATLTNEKESDACPIRAMKFSVSPV
O.latipes        IQRTILMMGRYVEPIEDVPCGNIVGLVGVDQFLVKTGTITT--FDQAHNMRVMKFSVSPV
P.troglodytes    IQR---------------------------------------------------------
P.falciparum     IQRTVLMMGRYTEQVQDVPCGNTCCLVGVDQYIVKSGTITTF--KEAHNIADMKYSVSPV
P.abelii         IQRTILMMGRYVEPIEDVPCGNIVGLVGVDQFLVKTGTITT--FEHAHNMRVMKFSVSPV
S.cerevisiae     IQRVVLMMGRFVEPIDDCPAGNIIGLVGIDQFLLKTGTLTT--SETAHNMKVMKFSVSPV
S.pombe          IQRTVLMMGSRIEPIEDCPAGNIIGLVGVDQFLVKSGTLTT--SEVAHNMKVMKFSVSPV
S.purpuratus     IQRTILMMGRYQEAIEDVPCGNICGLVGVDQFLVKTGTITT--YEYAHNIKTMKFSVSPV
S.scrofa         IQRTILMMGRYVEPIEDVPCGNIVGLVGVDQFLVKTGTITT--FEHAHNMRVMKFSVSPV
T.rubripes       IQRTILMMGRYVEPIEDVPCGNIVGLVGVDQYLVKTGTITT--YEQAHNMRVMKFSVSPV
X.tropicalis     IQRTILMMGRYVEPIEDVPCGNIVGLVGVDQFLVKTGTITT--FEHAHNMRVMKFSVSPV
                 ###                                                         


                        670       680       690       700       710       720
                 =========+=========+=========+=========+=========+=========+
A.carolinensis   VRVAVEAKNPADLPKLVEGLKRLAKSDPMVQCIIEESGEHIIAGAGELHLEICLKDLEED
A.gambiae        VRVAVEPKNPADLPKLVEGLKRLAKSDPMVQCIIEESGEHIIAGAGELHLEICLKDLEED
A.thaliana       VRVAVKCKLASDLPKLVEGLKRLAKSDPMVLCTMEESGEHIVAGAGELHIEICVKDLQD-
B.distachyon     VRVAVQCKVASDLPKLVEGLKRLAKSDPMVLCTIEESGEHIIAGAGELHLEICLKDLQDD
C.elegans        VRVAVEAKNPADLPKLVEGLKRLAKSDPMVQCIFEESGEHIIAGAGELHLEICLKDLEED
C.jacchus        VRVAVEAKNPADLPKLVEGLKRLAKSDPMVQCIIEESGEHIIAGAGELHLEICLKDLEED
C.neoformans     VQVAVECKNASDLPKLVEGLKRLSKSDPCVKTWMGDSGEIIVAGAGELHLEICLNDLEND
P.tetraurelia    VRVAVEPKAPGDLPKLVEGLKRLAKSDPLIQCTIEESGEHIIAGAGELHLEICLKDLQED
D.rerio          VRVAVEAKNPADLPKLVEGLKRLAKSDPMVQCIIEESGEHIVAGAGELHLEICLKDLEED
D.discoideum     VRVAVEPKNPSDLPKLVEGLKRLAKSDPCVLCYSEESGEHIVAGAGELHLEICLKDLAED
D.melanogaster   VRVAVEPKNPADLPKLVEGLKRLAKSDPMVQCIIEESGEHIIAGAGELHLEICLKDLEED
E.siliculosus    VRVAVEPKNQADLPKLVEGMKRLSKSDPMVLCYTEESGEHIIAGCGELHLEICLKDLQED
G.gallus         VRVAVEAKNPADLPKLVEGLKRLAKSDPMVQCIIEESGEHIIAGAGELHLEICLKDLEED
G.gorilla        VRVAVEAKNPADLPKLVEGLKRLAKSDPMVQCIIEESGEHIIAGAGELHLEICLKDLEED
H.sapiens        VRVAVEAKNPADLPKLVEGLKRLAKSDPMVQCIIEESGEHIIAGAGELHLEICLKDLEED
I.tridecemlinea  VRVAVEAKNPADLPKLVEGLKRLAKSDPMVQCIIEESGEHIIAGAGELHLEICLKDLEED
M.mulatta        VRVAVEAKNPADLPKLVEGLKRLAKSDPMVQCIIEESGEHIIAGAGELHLEICLKDLEED
M.gallopavo      VRVAVEAKNPADLPKLVEGLKRLAKSDPMVQCIIEESGEHIIAGAGELHLEICLKDLEED
M.musculus       VRVAVEAKNPADLPKLVEGLKRLAKSDPMVQCIIEESGEHIIAGAGELHLEICLKDLEED
O.sativa         VRVAVQCKVASDLPKLVEGLKRLAKSDPMVLCTIEESGEHIIAGAGELHLEICLKDLQED
O.latipes        VRVAVEAKNPADLPKLVEGLKRLAKSDPMVQCIIEESGEHIIAGAGELHLEICLKDLEED
P.troglodytes    ------------------------------------------------------------
P.falciparum     VRVAVKPKDSKQLPKLVDGLKKLAKSDPLVLCTTDESGEHIISGCGELHIEICLKDLKDE
P.abelii         VRVAVEAKNPADLPKLVEGLKRLAKSDPMVQCIIEESGEHIIAGAGELHLEICLKDLEED
S.cerevisiae     VQVAVEVKNANDLPKLVEGLKRLSKSDPCVLTYMSESGEHIVAGTGELHLEICLQDLEHD
S.pombe          VQVAVEVKNGNDLPKLVEGLKRLSKSDPCVLCTTSESGEHIVAGAGELHLEICLKDLQED
S.purpuratus     VRVAVEAKDPSQLPKLVEGLKRLAKSDPMVQCTIEESGEHIVAGAGELHLEICLKDLEED
S.scrofa         VRVAVEAKNPADLPKLVEGLKRLAKSDPMVQCIIEESGEHIIAGAGELHLEICLKDLEED
T.rubripes       VRVAVEVKNPSDLPKLVEGLKRLSKSDPMVQCIIEESGEHIVAGAGELHLEICLKDLEED
X.tropicalis     VRVAVEAKNPADLPKLVEGLKRLAKSDPMVQCIIEESGEHIIAGAGELHLEICLKDLEED
                                                                             


                        730       740       750       760       770       780
                 =========+=========+=========+=========+=========+=========+
A.carolinensis   HAC-IPIKKSDPVVSYRETVCEES----NQMCLSKSPNKHNRLYMKARPFPEGLAEDIDK
A.gambiae        HAC-IPLKKSDPVVSYRETVSDES----DQMCLSKSPNKHNRLFMKAVPMPDGLPDDIDN
A.thaliana       FMGGADIIVSDPVVSLRETVFERS----CRTVMSKSPNKHNRLYMEARPMEDGLAEAIDE
B.distachyon     FMGGAEIIVSPPVVSFRETVLEKS----SRTVMSKSPNKHNRLYMEARPLEEGLAEAIDD
C.elegans        HAC-IPLKKSDPVVSYRETVQSES----NQICLSKSPNKHNRLHCTAQPMPDGLADDIEG
C.jacchus        HAC-IPIKKSDPVVSYRETVSEES----NVLCLSKSPNKHNRLYMKARPFPDGLAEDIDK
C.neoformans     HAG-VPLRKSDPVVGYRETVTAES----SMIALSKSQNKHNRLYVKAEPLGEELTRDIEE
P.tetraurelia    FMNGAELIVSQPIVSYRETVLGVSNPELNSVCISKSPNKHNRIYCFAEPLKQGLAEAIEE
D.rerio          HAC-IPLKKSDPVVSYRETVSDES----DQVCLSKSPNKHNRLYMKSRPFPDGLAEDIDK
D.discoideum     HAG-IEIKTTDPVVSFRESV-------------------------KASPISMELQDLIEA
D.melanogaster   HAC-IPLKKSDPVVSYRETVSEES----DQMCLSKSPNKHNRLLMKALPMPDGLPEDIDN
E.siliculosus    FMG-TDVKISEPVVSYRETVSAES----STQCLSKSPNKHNRLYLSACPLESGIPEDVEE
G.gallus         HAC-IPIKKSDPVVSYRETVSEES----NVMCLSKSPNKHNRLYMKARPFPDGLAEDIDK
G.gorilla        HAC-IPIKKSDPVVSYRETVSEES----NVLCLSKSPNKHNRLYMKARPFPDGLAEDIDK
H.sapiens        HAC-IPIKKSDPVVSYRETVSEES----NVLCLSKSPNKHNRLYMKARPFPDGLAEDIDK
I.tridecemlinea  HAC-IPIKKSDPVVSYRETVSEES----NVLCLSKSPNKHNRLYMKARPFPDGLAEDIDK
M.mulatta        HAC-IPIKKSDPVVSYRETVSEES----NVLCLSKSPNKHNRLYMKARPFPDGLAEDIDK
M.gallopavo      HAC-IPIKKSDPVVSYRETVSEES----NVMCLSKSPNKHNRLYMKARPFPDGLAEDIDK
M.musculus       HAC-IPIKKSDPVVSYRETVSEES----NVLCLSKSPNKHNRLYMKARPFPDGLAEDIDK
O.sativa         FMGGAEIIVSPPVVSFRETVLEKS----CRTVMSKSPNKHNRLYMEARPLEEGLAEAIDD
O.latipes        HAC-IPLKKSDPVVSYRETVSEES----DQMCLSKSPNKHNRLFMKARPFPDGLAEDIEK
P.troglodytes    ------------------------------------------------------------
P.falciparum     YAQ-IDFIVSDPVVSYRETVTEES----TITCLGKSPNKHNRLFMKAYPLAEGLPEAIDK
P.abelii         HAC-IPIKKSDPVVSYRETVSEES----NVLCLSKSPNKHNRLYMKARPFPDGLAEDIDK
S.cerevisiae     HAG-VPLKISPPVVAYRETVESES----SQTALSKSPNKHNRIYLKAEPIDEEVSLAIEN
S.pombe          HAG-IPLKISPPVVSYRESVSEPS----SMTALSKSPNKHNRIFMTAEPMSEELSVAIET
S.purpuratus     HAG-IPLKKSDPVVSYREGVTAES----DRMCLSKSPNKHNRLFMRAAPLPDGLAEDIDN
S.scrofa         HAC-IPIKKSDPVVSYRETVSEES----NVLCLSKSPNKHNRLYMKARPFPDGLAEDIDK
T.rubripes       HAC-VPLKKSDPVVSYRETVSAES----NVMCLSKSPNKHNRLFMKARPFEDGLAEDIEK
X.tropicalis     HAC-IPLKKSDPVVSYRETVSEES----SQLCLSKSPNKHNRLFMKARPFPDGLAEDIDK
                                                                             


                        790       800       810       820       830       840
                 =========+=========+=========+=========+=========+=========+
A.carolinensis   G-DVSSRQELKQRARYLAEKYEWDVSEARKIWCFGPDGTGPNILVDITKGVQYLNEIKDS
A.gambiae        G-DVNARDEFKQRARYLSEKYDYDVTEARKIWCFGPDGTGPNIVVDCTKGVQYLNEIKDS
A.thaliana       G-RIGPSDDPKIRSKILAEEFGWDKDLAKKIWAFGPDTTGPNMVVDMCKGVQYLNEIKDS
B.distachyon     G-RIGPRDDPKVRSKILSEEFGWDKDLAKKIWCFGPETTGPNMVVDMCKGVQYLNEIKDS
C.elegans        G-TVSARDEFKARAKYPGEKYEYAVTEARKIWCFGPDGTGPNLLMDVTKGVQYLNEIKDS
C.jacchus        G-EVSARQELKQRARYLAEKYEWDVAEARKIWCFGPDGTGPNILTDITKGVQYLNEIKDS
C.neoformans     G-KVAPRDDPKIRARYLADTYGWDVTEARKIWCFGPDTTGPNVFLDGSKAVQYMNEIKDS
P.tetraurelia    G-KIKFNDEPKIRAKQLKKEFGMDEESAKKIWSFGPDMNGPNLLIDKTKGIQYLNEIKDS
D.rerio          G-DVSSRQELKLRARYLAEKYEWEVAEARKIWCFGPDGTGPNILVDITKGVQYLNEIKDS
D.discoideum     GSDISSKDDPKARANYLADNHEWDKNDAMNIWSFGPEGNGANLLVNVTKGVQYLNEIKDS
D.melanogaster   G-DVSAKDEFKARARYLSEKYDYDVTEARKIWCFGPDGTGPNFILDCTKSVQYLNEIKDS
E.siliculosus    G-RLNPRDDAKTRARYLADTYSWDVSEARKIWAFGPEGTGTNIFVDVTKGVNYLGEIRES
G.gallus         G-EVSARQELKQRARYLAEKYEWDVTEARKIWCFGPDGTGPNILTDITKGVQYLNEIKDS
G.gorilla        G-EVSARQELKQRARYLAEKYEWDVAEARKIWCFGPDGTGPNILTDITKGVQYLNEIKDS
H.sapiens        G-EVSARQELKQRARYLAEKYEWDVAEARKIWCFGPDGTGPNILTDITKGVQYLNEIKDS
I.tridecemlinea  G-EVSARQELKQRARYLAEKYEWDVAEARKIWCFGPDGTGPNILTDITKGVQYLNEIKDS
M.mulatta        G-EVSARQELKQRARYLAEKYEWDVAEARKIWCFGPDGTGPNILTDITKGVQYLNEIKDS
M.gallopavo      G-EVSARQELKQRARYLAEKYEWDVTEARKIWCFGPDGTGPNILTDITKGVQYLNEIKDS
M.musculus       G-EVSARQELKARARYLAEKYEWDVAEARKIWCFGPDGTGPNILTDITKGVQYLNEIKDS
O.sativa         G-RIGPRDDPKVRSKILSEEFGWDKDLAKKIWCFGPETTGPNMVVDMCKGVQYLNEIKDS
O.latipes        G-DVSARQELKARARYLADKYEWEVTEARKIWCFGPDGSGPNLLIDVTKGVQYLNEIKDS
P.troglodytes    ------------------------------------------------------------
P.falciparum     N-KVSDKDDPKTRANYLHSNFQWDKNLALKIWAFGPETIGPNLLTDNTSGIQYMNEIKVH
P.abelii         G-EVSARQELKQRARYLAEKYEWDVAEARKIWCFGPDGTGPNILTDITKGVQYLNEIKDS
S.cerevisiae     G-IINPRDDFKARARIMADDYGWDVTDARKIWCFGPDGNGPNLVIDQTKAVQYLHEIKDS
S.pombe          G-HVNPRDDFKVRARIMADEFGWDVTDARKIWCFGPDTTGANVVVDQTKAVAYLNEIKDS
S.purpuratus     G-EVSSKQDFKLRSRYLIDKYNFEAQESRKIWCFGPEGTGPNLLVDCAKGVQYLNEIKDS
S.scrofa         G-EVSARQELKQRARYLAEKYEWDVAEARKIWCFGPDGTGPNILTDITKGVQYLNEIKDS
T.rubripes       G-DVTARQELKARARHLVEKHSWEVGEARKIWCFGPDGTGPNLLVDVTKGVQYLNEIKDS
X.tropicalis     G-DVSSRQELKQRARYLAEKYEWDVAEARKIWCFGPDGTGPNILVDVTKGVQYLNEIKDS
                                                                             


                        850       860       870       880       890       900
                 =========+=========+=========+=========+=========+=========+
A.carolinensis   VVAGFQWATKEG-----VLCEENMRGVRFDIHDVTLHADAIHRGGGQIIPTARRVLYASA
A.gambiae        VVAGFQWASKEG-----VLAEENMRGVRFNIYDVALHADAIHRGGGQIIPTARRVLYASY
A.thaliana       VVAGFQWASKEG-----PLAEENMRGVCYEVCDVVLHADAIHRGCGQMISTARRAIYASQ
B.distachyon     VVAGFQWASKEG-----ALAEENMRGICFEVCDVVLHTDAIHRGGGQVIPTARRVIYASQ
C.elegans        VVAGFQWATREG-----VLSDENMRGVRFNVHDVTLHADAIHRGGGQIIPTARRVFYASV
C.jacchus        VVAGFQWATKEG-----ALCEENMRGVRFDVHDVTLHADAIHRGGGQIIPTARRCLYASV
C.neoformans     CVAAFQWATKEG-----GVAEEPMRGVRFNILDCTLHADAIHRGGGQIIPTARRVCYAAQ
P.tetraurelia    CVSAFQWVSKEG-----VLCSENIRNISFNIVDVILHADSIHRGGGQIIPTARRSFYGAQ
D.rerio          VVAGFQWATKEG-----ALCEENMRAVRFDIHDVTLHADAIHRGGGQIIPTARRVLYASV
D.discoideum     FVGAFQWATKEG-----VVCDENMRGIRFNLYDVTLHTDAIHRGGGQIIPTARRVLYAAE
D.melanogaster   VVAGFQWASKEG-----ILADENLRGVRFNIYDVTLHADAIHRGGGQIIPTTRRCLYAAA
E.siliculosus    VIGGFNWAMNEG-----PMTEEKVRGVRFNLLDVVLHADAIHRGMGQIMPTARRVVYASM
G.gallus         VVAGFQWATKEG-----VLCEENMRGVRFDVHDVTLHADAIHRGGGQIIPTARRCLYACV
G.gorilla        VVAGFQWATKEG-----ALCEENMRGVRFDVHDVTLHADAIHRGGGQIIPTARRCLYASV
H.sapiens        VVAGFQWATKEG-----ALCEENMRGVRFDVHDVTLHADAIHRGGGQIIPTARRCLYASV
I.tridecemlinea  VVAGFQWATKEG-----ALCEENMRGVRFDVHDVTLHADAIHRGGGQIIPTARRCLYASV
M.mulatta        VVAGFQWATKEG-----ALCEENMRGVRFDVHDVTLHADAIHRGGGQIIPTARRCLYASV
M.gallopavo      VVAGFQWATKEXGWCCGVLCEENMRGVRFDVHDVTLHADAIHRGGGQIIPTARRCLYACV
M.musculus       VVAGFQWATKEG-----ALCEENMRGVRFDVHDVTLHADAIHRGGGQIIPTARRCLYASV
O.sativa         VVAGFQWASKEG-----ALAEENMRGICFEVCDVVLHADAIHRGGGQVIPTARRVIYASQ
O.latipes        VVAGFQWASKEG-----ALCEENMRAVRFDIHDVTLHADAIHRGGGQIIPTARRVLYACQ
P.troglodytes    ------------------------------------------------------------
P.falciparum     CVAAFQWASKEG-----VLCEENMRGIEFRMLDVHMHADAIHRGAGQIMPACKKCIYACE
P.abelii         VVAGFQWATKEG-----ALCEENMRGVRFDVHDVTLHADAIHRGGGQIIPTARRCLYASV
S.cerevisiae     VVAAFQWATKEG-----PIFGEEMRSVRVNILDVTLHADAIHRGGGQIIPTMRRATYAGF
S.pombe          VVAAFAWASKEG-----PMFEENLRSCRFNILDVVLHADAIHRGGGQIIPTARRVVYAST
S.purpuratus     VIAGFQWASKEG-----VLSEENLRGVRYNIYDVTLHTDAIHRGGGQIIPTTRRCLLACQ
S.scrofa         VVAGFQWATKEG-----ALCEENMRGVRFDVHDVTLHADAIHRGGGQIIPTARRCLYASV
T.rubripes       VVAGFQWAVKEG-----VLCEENMRAIRFDIHDVTLHTDAIHRGGGQIIPTARRALYACE
X.tropicalis     VVAGFQWATKEG-----VLCEENLRGARFDIHDVTLHADAIHRGGGQIIPTARRVLYASA
                                                                             


                        910       920       930       940       950       960
                 =========+=========+=========+=========+=========+=========+
A.carolinensis   LTAQPRLMEPIYLVEIQCPEQVVGGIYGVLNRKRGHVFEESQVAGTPMFVVKAYLPVNES
A.gambiae        ITASPRIMEPVYLCEIQCPEAAVGGIYGVLNRRRGHVFEDSQVAGTPMFVVKAYLPVNES
A.thaliana       LTAKPRLLEPVYMVEIQAPEGALGGIYSVLNQKRGHVFEEMQRPGTPLYNIKAYLPVVES
B.distachyon     LTAKPRLLEPVYLVEIQAPENALGGIYGVLNQKRGHVFEEMQRPGTPLYNIKAYLPVIES
C.elegans        LTAEPRLLEPVYLVEIQCPEAAVGGIYGVLNRRRGHVFEESQVTGTPMFVVKAYLPVNES
C.jacchus        LTAQPRLMEPIYLVEIQCPEQVVGGIYGVLNRKRGHVFEESQVAGTPMFVVKAYLPVNES
C.neoformans     LLATPAFQEPMFLVEIAVPESAQGGVYSCLNVRRGHVFSAEQRPGTPMYTLKAYLPVSES
P.tetraurelia    LLAKPRLLEPVYLVEIQCPEQVVSSVYSVLNRKRGQVFEETKKVGTPMFTLKAFLPVQES
D.rerio          LTAQPRLMEPIYLVEIQCPEQVVGGIYGVLNRKRGHVFEESQVAGTPIFVVKAYLPVNES
D.discoideum     LTASPTLLEPIYLVEITAPENAIGGIYSVLNRRRGIVIGEERRIGSPLFSVKAHLPVLES
D.melanogaster   ITAKPRLMEPVYLCEIQCPEVAVGGIYGVLNRRRGHVFEENQVVGTPMFVVKAYLPVNES
E.siliculosus    LTASPMLLEPVFLCEISCPQDAMGGCYGVLTQRRGHVFAEEQRPGTPMMTLKAYLPVMES
G.gallus         LTAQPRLMEPIYLVEIQCPEQVVGGIYGVLNRKRGHVFEESQVAGTPMFVVKAYLPVNES
G.gorilla        LTAQPRLMEPIYLVEIQCPEQVVGGIYGVLNRKRGHVFEESQVAGTPMFVVKAYLPVNES
H.sapiens        LTAQPRLMEPIYLVEIQCPEQVVGGIYGVLNRKRGHVFEESQVAGTPMFVVKAYLPVNES
I.tridecemlinea  LTAQPRLMEPIYLVEIQCPEQVVGGIYGVLNRKRGHVFEESQVAGTPMFVVKAYLPVNES
M.mulatta        LTAQPRLMEPIYLVEIQCPEQVVGGIYGVLNRKRGHVFEESQVAGTPMFVVKAYLPVNES
M.gallopavo      LTAQPRLMEPIYLVEIQCPEQVVGGIYGVLNRKRGHVFEESQVAGTPMFVVKAYLPVNES
M.musculus       LTAQPRLMEPIYLVEIQCPEQVVGGIYGVLNRKRGHVFEESQVAGTPMFVVKAYLPVNES
O.sativa         LTAKPRLLEPVYLVEIQAPENALGGIYGVLNQKRGHVFEEMQRPGTPLYNIKAYLPVIES
O.latipes        LTAQPRLMEPIYLVEIQCPEQVVGGIYGVLNRKRGHVFEESQVMGTPMFVVKAYLPVNES
P.troglodytes    ------------------------------------------------------------
P.falciparum     LTAFPRLVEPIYLVDISCPQDVVSGVYGVLNKRRGIVISEEQKLGTPLLKIQSHLPVSES
P.abelii         LTAQPRLMEPIYLVEIQCPEQVVGGIYGVLNRKRGHVFEESQVAGTPMFVVKAYLPVNES
S.cerevisiae     LLADPKIQEPVFLVEIQCPEQAVGGIYSVLNKKRGQVVSEEQRPGTPLFTVKAYLPVNES
S.pombe          LLASPIIQEPVFLVEIQVSENAMGGIYSVLNKKRGHVFSEEQRVGTPLYNIKAYLPVNES
S.purpuratus     LTATPRVMEPVYLVEIQCPESAVGGIYGVLNRRRGHVFEENQKIGTPMFFVKAYLPVNES
S.scrofa         LTAQPRLMEPIYLVEIQCPEQVVGGIYGVLNRKRGHVFEESQVAGTPMFVVKAYLPVNES
T.rubripes       LTAQPKIMEPVYLVEIQCPETALGGIYQVLNKRRGHLFDDVNITGTPMHLVKAYLPVNES
X.tropicalis     LTAQPRLMEPIYLVEIQCPEQVVGGIYGVLNRKRGHVFEESQVAGTPMFVVKAYLPVNES
                                                                             


                        970       980       990      1000      1010      1020
                 =========+=========+=========+=========+=========+=========+
A.carolinensis   FGFTADLRSNTGGQAFPQCVFDHWQVLPGDPYDPNSRPCQVVAETRKRKGLKESISPLDN
A.gambiae        FGFTADLRSNTGGQAFPQCVFDHWQIFPGDPTDPSTKPYQIIQDIRKRKGLKEGLPDLSQ
A.thaliana       FGFSGQLRAATSGQAFPQCVFDHWDMMSSDPLETGSQAATLVADIRKRKGLKLQMTPLSD
B.distachyon     FGFSSTLRAATSGQAFPQCVFDHWDIMSSDPLEAGTQSATLVTEIRKRKGLKEQMTPLSE
C.elegans        FGFTADLRSNTGGQAFPQCVFDHWQVLPGDPLEAGTKPNQIVLDTRKRKGLKEGVPALDN
C.jacchus        FGFTADLRSNTGGQAFPQCVFDHWQILPGDPFDNTSRPSQVVAETRKRKGLKEGIPALDN
C.neoformans     FGFNADLRAATGGQAFPQAVFDHWEEMNSNPTEVGSKTNLLAVNIRTRKGLKPDVPPYDT
P.tetraurelia    FGFTTDLRASTAGQAFPQCVFDHWQIIQGNPLDKTDKSFELVKNIRKRKGMKDDIPTIDV
D.rerio          FGFTADLRSNTGGQAFPQCVFDHWQILPGDPYDVNSKPSQIVADTRKRKGLKEGIPALDN
D.discoideum     LRFTADLRSHTAGQAFPQCVFDHWASIG--VVNKDKKATEVALATRKRKGLAPEIPALDK
D.melanogaster   FGFTADLRSNTGGQAFPQCVFDHWQVLPGDPSEPSSKPYAIVQDTRKRKGLKEGLPDLSQ
E.siliculosus    FGFTKDLRSNTGGKAFPQCVFDHWQEMSGDPQSEGSKSYTVVREVRKRKGLVEDIPPLDR
G.gallus         FGFTADLRSNTGGQAFPQCVFDHWQILPGDPFDSASRPSQVVAETRKRKGLKEGIPALDN
G.gorilla        FGFTADLRSNTGGQAFPQCVFDHWQILPGDPFDNSSRPSQVVAETRKRKGLKEGIPALDN
H.sapiens        FGFTADLRSNTGGQAFPQCVFDHWQILPGDPFDNSSRPSQVVAETRKRKGLKEGIPALDN
I.tridecemlinea  FGFTADLRSNTGGQAFPQCVFDHWQILPGDPFDNTSRPSQVVAETRKRKGLKEGIPALDN
M.mulatta        FGFTADLRSNTGGQAFPQCVFDHWQILPGDPFDNSSRPSQVVAETRKRKGLKEGIPALDN
M.gallopavo      FGFTADLRSNTGGQAFPQCVFDHWQILPGDPFDSTSRPSQVVAETRKRKGLKEGIPALDN
M.musculus       FGFTADLRSNTGGQAFPQCVFDHWQILPGDPFDNSSRPSQVVAETRKRKGLKEGIPALDN
O.sativa         FGFSSQLRAATSGQAFPQCVFDHWDMMTSDPLEAGSQASTLVQDIRKRKGLKEQMTPLSD
O.latipes        FGFTADLRSNTGGQAFPQCVFDHWQILQGDPNDPATRPCQVVAEIRKRKGLKEGIPALDN
P.troglodytes    ------------------------------------------------------------
P.falciparum     FGFTSALRAATSGQA---------------------------------------------
P.abelii         FGFTADLRSNTGGQAFPQCVFDHWQILPGDPFDNSSRPSQVVAETRKRKGLKEGIPALDN
S.cerevisiae     FGFTGELRQATGGQAFPQMVFDHWSTLGSDPLDPTSKAGEIVLAARKRHGMKEEVPGWQE
S.pombe          FGFTGELRQATAGQAFPQLVFDHWSPMSGDPLDPTSKPGQIVCEARKRKGLKENVPDYTE
S.purpuratus     FGFTADLRSNTGGQAFPQCVFDHWQVMGDDPIDPTTKSGIIVTGIRKRKALSEEVPHLEK
S.scrofa         FGFTADLRSNTGGQAFPQCVFDHWQILPGDPFDNTSRPSQVVAETRKRKGLKEGIPALDN
T.rubripes       FGFTADLRSSTGGQAFPQCVFDHWQILPGNPFEADSKPGLVVAETRKRKGLKEEIPALDN
X.tropicalis     FGFTADLRSNTGGQAFPQCVFDHWQILPGDPFDSTSRPSQVVGETRKRKGLKEGIPPLDN
                                                                             


                       1030      1040      1050      1060      1070      1080
                 =========+=========+=========+=========+=========+=========+
A.carolinensis   FLDKL------------M---------SG-K--APAIGIDLGTTYSCVGVFQHGKVEIIA
A.gambiae        YLDKL------------MA--------AA-K--APAVGIDLGTTYSCVGVFQHGKVEIIA
A.thaliana       YEDKL------------MA--------G--KGEGPAIGIDLGTTYSCVGVWQHDRVEIIA
B.distachyon     FEDKL----------------------------MSVVGFDLGNESCIVGVARQRGIDVVL
C.elegans        YLDKM--------------------MSTC-K----AIGIDLGTTYSCVGIYQNGKVEILA
C.jacchus        FLDKL----------------------MA-K--AAAIGIDLGTTYSCVGVFQHGKVEIIA
C.neoformans     YYDKL------------MV--------K-------AVGIDLGTTYSCVAVWQNDRVEIIA
P.tetraurelia    FYDKI-------------------------M--KKIVGIDLGTTNSVVAVMEGGKPTVIP
D.rerio          FLDKL------------MS--------SP-K--GIAIGIDLGTTYSCVGVFQHGKVEIIA
D.discoideum     FHRKTINNLSHTLSFQIMS----------------SIGIDLGTTYSCVGVWQNDRVEIIA
D.melanogaster   YLDKL----------------------------MPAIGIDLGTTYSCVGVYQHGKVEIIA
E.siliculosus    YLDRL----------------------------MSVIGIDFGNDTCVIGMAARGGIDIIL
G.gallus         FLDKL------------M---------SG-K--GPAIGIDLGTTYSCVGVFQHGKVEIIA
G.gorilla        FLDKL----------------------MA-K--AAAIGIDLGTTYSCVGVFQHGKVEIIA
H.sapiens        FLDKL----------------------MA-K--AAAIGIDLGTTYSCVGVFQHGKVEIIA
I.tridecemlinea  FLDKL------------MA--------AA-K--GTAIGIDLGTTYSCVGVFQHGKVEIIA
M.mulatta        FLDKL----------------------MA-K--AAAIGIDLGTTYSCVGVFQHGKVEIIA
M.gallopavo      FLDKL------------M---------SG-K--GPAIGIDLGTTYSCVGVFQHGKVEIIA
M.musculus       FLDKL----------------------MA-K--NTAIGIDLGTTYSCVGVFQHGKVEIIA
O.sativa         FEDKL------------MA--------GN-KGEGPAIGIDLGTTYSCVGVWQHDRVEIIA
O.latipes        YLDKL----------------------MS-K--GPAVGIDLGTTYSCVGIFQHGKVEIIA
P.troglodytes    ---------------------------MA-K--AAAIGIDLGTTYSCVGVFQHGKVEIIA
P.falciparum     -----------------MASAKGSKPNLPES--NIAIGIDLGTTYSCVGVWRNENVDIIA
P.abelii         FLDKL----------------------MA-K--AAAIGIDLGTTYSCVGVFQHGKVEIIA
S.cerevisiae     YYDKL------------MS--------K-------AVGIDLGTTYSCVAHFANDRVDIIA
S.pombe          YYDRL------------MS--------K-------SIGIDLGTTYSCVGHFSNNRVEIIA
S.purpuratus     YLDKM------------MS--------N-----PTAIGIDLGTTYSCVGVFQNGRVEIIA
S.scrofa         FLDKL------------MS--------AA-R--EVAIGIDLGTTYSCVGVFQHGRVEILA
T.rubripes       YLDKL------------MS--------AA-K--GLAIGIDLGTTYSCVGVSQHGKVEIIA
X.tropicalis     FLDKL-------------M--------AS-K--GVAVGIDLGTTYSCVGVFQHGKVEIIA
                                                    #########################


                       1090      1100      1110      1120      1130      1140
                 =========+=========+=========+=========+=========+=========+
A.carolinensis   NDQGNRTTPSYVAFT-DTERLIGDAAKNQVAMNPNNTIFDAKRLIGRKFEDATVQSDMKH
A.gambiae        NDQGNRTTPSYVAFT-DTERLIGDAAKNQVAMNPTNTIFDAKRLIGRKFDDPAIQADMKH
A.thaliana       NDQGNRTTPSYVAFT-DSERLIGDAAKNQVAMNPINTVFDAKRLIGRRFTDSSVQSDIKL
B.distachyon     NEESKRETPAIVCFG-DKQRFIGTAGAANSTMNPKNSISQIKRLLGRKFTDPELQHDLQS
C.elegans        NSEGNKTTPSYVAFT-DTERLVGDAAKDQAARNPENTVFDAKRLIGRRFDEETVQSDIKH
C.jacchus        NDQGNRTTPSYVAFT-DTERLIGDAAKNQVALNPQNTVFDAKRLIGRKFGDPVVQSDMKH
C.neoformans     NDQGNRTTPSYVAFN-DSERLIGDAAKNQVAMNPYNTVFDAKRLIGRKFEDAEVQADMKH
P.tetraurelia    NAEGFRTTPSIVAYTKNGERLVGQIAKRQAVINPDNTFYSVKRFIGRKSEE--VSEELKQ
D.rerio          NDQGNRTTPSYVAFT-DTERLIGDAAKNQVAMNPNNTVFDAKRLIGRRFDDPVVQSDMKH
D.discoideum     NDQGNRTTPSYVAFT-DTERLIGDAAKNQVAMNPTNTVFDAKRLIGRKFSDKEVQSDMKH
D.melanogaster   NDQGNRTTPSYVAFT-DSERLIGDPAKNQVAMNPRNTVFDAKRLIGRKYDDPKIAEDMKH
E.siliculosus    NENSNRKNPTLVSFQ-GKQRLMGEGAASIARSNFKNTAREVKRLVGRVWGTPDLEADLAR
G.gallus         NDQGNRTTPSYVAFT-DTERLIGDAAKNQVAMNPTNTIFDAKRLIGRKYDDPTVQSDMKH
G.gorilla        NDQGNRTTPSYVAFT-DTERLIGDAAKNQVALNPQNTVFDAKRLIGRKFGDPVVQSDMKH
H.sapiens        NDQGNRTTPSYVAFT-DTERLIGDAAKNQVALNPQNTVFDAKRLIGRKFGDPVVQSDMKH
I.tridecemlinea  NDQGNRTTPSYVAFT-DTERLIGDAAKNQVAMNPQNTVFDAKRLIGRKFNDPVVQSDMKL
M.mulatta        NDQGNRTTPSYVAFT-DTERLIGDAAKNQVALNPQNTVFDAKRLIGRKFGDPVVQSDMKH
M.gallopavo      NDQGNRTTPSYVAFT-DTERLIGDAAKNQVAMNPTNTIFDAKRLIGRKYDDPTVQSDMKH
M.musculus       NDQGNRTTPSYVAFT-DTERLIGDAAKNQVALNPQNTVFDAKRLIGRKFGDAVVQSDMKH
O.sativa         NDQGNRTTPSYVAFT-DTERLIGDAAKNQVAMNPTNTVFDAKRLIGRRFSDPSVQADMKM
O.latipes        NDQGNRTTPSYVAFT-DTERLIGDAAKNQVAMNPANTVFDAKRLIGRKFDDAVVQSDMKH
P.troglodytes    NDQGNRTTPSYVAFT-DTERLIGDAAKNQVALNPQNTVFDAKRLIGRKFGDPVVQSDMKH
P.falciparum     NDQGNRTTPSYVAFT-DTERLIGDAAKNQVARNPENTVFDAKRLIGRKFTESSVQSDMKH
P.abelii         NDQGNRTTPSYVAFT-DTERLIGDAAKNQVALNPQNTVFDAKRLIGRKFGDPVVQSDMKH
S.cerevisiae     NDQGNRTTPSFVAFT-DTERLIGDAAKNQAAMNPSNTVFDAKRLIGRNFNDPEVQADMKH
S.pombe          NDQGNRTTPSYVAFT-DTERLIGDAAKNQVAMNPHNTIFDAKRLIGRKFDDPEVQSDMKH
S.purpuratus     NDQGNRTTPSYVAFT-DTERLIGDAAKNQVAMNPRNTIFDAKRLIGRRFDDTAVQSDMKH
S.scrofa         NDQGNRTTPSYVAFT-DTERLVGDAAKSQAALNPQNTVFDAKRLIGRKFADPTVQSDLKH
T.rubripes       NDQGNRTTPSYVAFT-DTERLIGDAAKNQVALNPSNTVFDAKRLIGRRLEDPTAQADIKH
X.tropicalis     NDQGNRTTPSYVAFT-DTERLIGDAAKNQVAMNPQNTVFDAKRLIGRKFNDPVVQSDLKH
                 ############### ###################################  #######


                       1150      1160      1170      1180      1190      1200
                 =========+=========+=========+=========+=========+=========+
A.carolinensis   WPFRVVS-E-A--GKPKVQVEYKGELKTFFPEEISSMVLTKMKEIAEAYL-GRKVQSAVI
A.gambiae        WPFEVES-I-E--GKPKIAVEYKGEKKCFFPEEVSSMVLTKMKETAEAYL-GKTVTNAVI
A.thaliana       WPFTLKS-G-PA-EKPMIVVNYKGEDKEFSAEEISSMILIKMREIAEAYL-GTTIKNAVV
B.distachyon     FPFHVSE-GPD--GFPLVHARYLGEERSFTPTQLLAMVLSNLKGIAEGNL-NSAVIDCCI
C.elegans        WPFTVKG-K-Q--GKPVVEVEVKGEKREFNAEEISAMVLQKMKETAEAVL-GHSVRDAVI
C.jacchus        WPFQVIN-D-G--DKPKVQVSYKGETKAFYPEEISSMVLTKMKEIAEAYL-GYPVTNAVI
C.neoformans     WPFKVID-R-AG--KPAIQVEYRGEEKVFTPEEISSMVLIKMKETAEAYL-GGTVSKAVV
P.tetraurelia    VSYIVKTDS-Q--GNIKLECPIL--KRDFAAEELSAEVLRKLVDDASVYL-GEQVKQAVI
D.rerio          WSFKVVS-D-G--GKPKVAVEHKGENKTFNPEEISSMVLVKMKEIAEAYL-GQKVTNAVI
D.discoideum     WPFKVIP-K-DG-DKPHIQVEFKGETKVFSPEEISSMVLLKMKETAEAYL-GKTINNAVI
D.melanogaster   WPFKVVS-D-G--GKPKIGVEYKGESKRFAPEEISSMVLTKMKETAEAYL-GESITDAVI
E.siliculosus    LPFKCIQ-H-PETGGVGIEVNYEDSIKVFTPEQIIACMLTKLIAIAKAANNGIDVADTVM
G.gallus         WPFRVVN-E-G--GKPKVQVEYKGEMKTFFPEEISSMVLTKMKEIAEAYL-GKKVETAVI
G.gorilla        WPFQVIN-D-G--DKPKVQVSYKGETKAFYPEEISSMVLTKMKEIAEAYL-GYPVTNAVI
H.sapiens        WPFQVIN-D-G--DKPKVQVSYKGETKAFYPEEISSMVLTKMKEIAEAYL-GYPVTNAVI
I.tridecemlinea  WPFQVIN-E-G--GKPKVLVSYKGEKKAFYPEEISSMVLTKMKETAEAFL-GHLITNAVI
M.mulatta        WPFQVIN-D-G--DKPKVQVSYKGETKAFYPEEISSMVLTKMKEIAEAYL-GYPVTNAVI
M.gallopavo      WPFRVVN-E-S--GKPKVQVEYKGEMKTFFPEEISSMVLTKMKEIAEAYL-GKKVQNAVI
M.musculus       WPFQVVN-D-G--DKPKVQVNYKGESRSFFPEEISSMVLTKMKEIAEAYL-GHPVTNAVI
O.sativa         WPFKVVP-G-PA-DKPMIVVTYKGEEKKFSAEEISSMVLTKMKEIAEAFL-STTIKNAVI
O.latipes        WPFKVVN-D-S--SKPKVEVEYKGEIKTFYPEEISSMVLTKMKEISEAYL-GKSVTNAVI
P.troglodytes    WPFQVIN-D-G--DKPKVQVSYKGETKAFYPEEISSMVLTKMKEIAEAYL-GYPVTNAVI
P.falciparum     WPFTVKS-G-VD-EKPMIEVTYQGEKKLFHPEEISSMVLQKMKENAEAFL-GKSIKNAVI
P.abelii         WPFQVNN-D-G--DKPKVQVSYKGETKAFYPEEISSMVLTKMKEIAEAYL-GYPVTNAVI
S.cerevisiae     FPFKLID-V-DG--KPQIQVEFKGETKNFTPEQISSMVLGKMKETAESYL-GAKVNDAVV
S.pombe          WPFKVIS-K-DG--KPVLQVEYKGETKTFTPEEISSMVLMKMRETAEAYL-GGKVTDAVV
S.purpuratus     WSFTVTN-N-G--GKPMLQAEYMGETKRFAPEEISSMVLTKMRETAEAYL-GYKINDAVI
S.scrofa         WPFQVVS-E-G--GKPKVRVSYRGEDKAFYPEEISSMVLSKMKETAEAYL-GQPVRHAVI
T.rubripes       WPFKVVG-D-G--GRPKIQVVYKGEEKSFYPEEISSMVLVKMKEIAEAYL-GQRVSSAVI
X.tropicalis     WPFQVVS-D-H--GKPKVKVEFKGEEKTFFPEEISSMVLIKMKETAEAYL-GHAVTNAVI
                 #####                     ######################## #########


                       1210      1220      1230      1240      1250      1260
                 =========+=========+=========+=========+=========+=========+
A.carolinensis   TVPAYFNDSQRQATKDAGTITGLNVLRIINEPTAAAIAYGLDKKGS-GA---GE---KNV
A.gambiae        TVPAYFNDSQRQATKDAGTISGLNVLRIINEPTAAAIAYGLDKK---TA---GE---RNV
A.thaliana       TVPAYFNDSQRQATKDAGVIAGLNVMRIINEPTAAAIAYGLDKKAT-SV---GE---KNV
B.distachyon     GIPVYFTNLQRRAVLDAATIAGLRPLRLFHETTATALAYGIYKTDLPEN---DQ---LNV
C.elegans        TVPAYFNDSQRQATKDAATIAGLNAIRIINEPTAAALAYGLDKG---IT---DE---KNI
C.jacchus        TVPAYFNDSQRQATKDAGVIAGLNVLRIINEPTAAAIAYGLDRT---GK---GE---RNV
C.neoformans     TVPAYFNDSQRQATKDAGAIAGLDVLRIINEPTAAAIAYGLDK--K-SE---GE---KNV
P.tetraurelia    TVPAYFNDSQRQATKDSGKIAGLEVLRIINEPTAASLAYGLDKK--------SN---ETI
D.rerio          TVPAYFNDSQRQATKDAGVIAGLNVLRIINEPTAAAIAYGLDKG---KS---SE---RNV
D.discoideum     TVPAYFNDSQRQATKDAGTISKLNVQRIINEPTAAAIRYGLEK--K-GS---GE---KNI
D.melanogaster   TVPAYFNDSQRQATKDAGHIAGLNVLRIINEPTAAALAYGLDKN---LK---GE---RNV
E.siliculosus    AIPGWYTDAMRNAMMNACSIGGVNCLRLIHEHTATALAYGIYKS---AKGEFSEKEPQFV
G.gallus         TVPAYFNDSQRQATKDAGTITGLNVMRIINEPTAAAIAYGLDKKGT-RA---GE---KNV
G.gorilla        TVPAYFNDSQRQATKDAGVIAGLNVLRIINEPTAAAIAYGLDRT---GK---GE---RNV
H.sapiens        TVPAYFNDSQRQATKDAGVIAGLNVLRIINEPTAAAIAYGLDRT---GK---GE---RNV
I.tridecemlinea  TVPAYFNDSQRQATKDAGVIAGLNVLRIINEPTAAAIAYGLDKA---GQ---GE---RHV
M.mulatta        TVPAYFNDSQRQATKDAGVIAGLNVLRIINEPTAAAIAYGLDRT---GK---GE---RNV
M.gallopavo      TVPAYFNDSQRQATKDAGTITGLNVMRIINEPTAAAIAYGLDKKGT-RA---GE---KNV
M.musculus       TVPAYFNDSQRQATKDAGVIAGLNVLRIINEPTAAAIAYGLDRT---GK---GE---RNV
O.sativa         TVPAYFNDSQRQATKDAGVISGLNVMRIINEPTAAAIAYGLDKKAA-ST---GE---KNV
O.latipes        TVPAYFNDSQRQATKDAGTIAGLNVLRIINEPTAAAIAYGLDKK---VG---GE---RNV
P.troglodytes    TVPAYFNDSQRQATKDAGVIAGLNVLRIINEPTAAAIAYGLDRT---GK---GE---RNV
P.falciparum     TVPAYFNDSQRQATKDAGTIAGLNVMRIINEPTAAAIAYGLHKK---GK---GE---KNI
P.abelii         TVPAYFNDSQRQATKDAGVIAGLNVLRIINEPTAAAIAYGLDRT---GK---GE---RNV
S.cerevisiae     TVPAYFNDSQRQATKDAGTIAGLNVLRIINEPTAAAIAYGLDK--K-GK----E---EHV
S.pombe          TVPAYFNDSQRQATKDAGLIAGLNVLRIINEPTAAAIAYGLDR--S-NQ---GE---SNV
S.purpuratus     TVPAYFNDSQRTATKDAGKIAGLNVLRIINEPTAAALAYGLDKK---LR---GE---QNV
S.scrofa         TVPAYFNDSQRQATKDAGAIAGLNVLRIINEPTAAAIAYGLDRR---GA---GE---RNV
T.rubripes       TVPAYFNDSQRQATKDAGVIAGLNVLRIINEPTAAAIAYGLDKG---KS---GE---RNV
X.tropicalis     TVPAYFNDSQRQATKDAGVIAGLNVLRIINEPTAAAIAYGLDKG---TR---GE---RNV
                 ###########################################               ##


                       1270      1280      1290      1300      1310      1320
                 =========+=========+=========+=========+=========+=========+
A.carolinensis   LIFDLGGGTFDVSILTIEDG-IFEVKSTAGDTHLGGEDFDNRMVSHFVEEFKRKHK-RDI
A.gambiae        LIFDLGGGTFDVSILSIDDG-IFEVKSTAGDTHLGGEDFDNRLVNHFAQEFKRKHK-KDL
A.thaliana       LIFDLGGGTFDVSLLTIEEG-IFEVKATAGDTHLGGEDFDNRMVNHFVQEFKRKNK-KDI
B.distachyon     AFVDVGHASMQVSIVGYKKG-QLKMLSHAYDRSLGGRDFDEALFKHFAAKFKEEYK-IDV
C.elegans        LIFDLGGGTFDVSILSIAEGSIFEVKSTAGDTHLGGEDFDSRVLQHFMTEFKRKTG-KDI
C.jacchus        LIFDLGGGTFDVSILTIDDG-IFEVKATAGDTHLGGEDFDNRLVNHFVEEFKRKHK-KDI
C.neoformans     LIFDLGGGTFDVSLLTIEEG-IFEVKATAGDTHLGGEDFDNRLVNHFVQEFKRKNK-KDL
P.tetraurelia    LVFDLGGGTFDVSVLEVGEG-VFEVLSTAGDTHLGGDDFDDKVVKWLLQEFKNEQG-VDL
D.rerio          LIFDLGGGTFDVSILTIEDG-IFEVKATAGDTHLGGEDFDNRMVNHFVEEFKRKHK-KDI
D.discoideum     LIFDLGGGTFDVSLLTIEDG-VFEVKATAGDTHLGGEDFDNRLVSHFVDEFKRKHK-KDI
D.melanogaster   LIFDLGGGTFDVSILTIDEGSLFEVRSTAGDTHLGGEDFDNRLVTHLADEFKRKYK-KDL
E.siliculosus    LFLDVGHSSFSASVVTFVQG-SLTVKSAAFDSKLGGRDMDWAIAQHAADEFKSKTG-KDP
G.gallus         LIFDLGGGTFDVSILTIEDG-IFEVKSTAGDTHLGGEDFDNRMVNRFVEEFKGKHK-RDN
G.gorilla        LIFDLGGGTFDVSILTIDDG-IFEVKATAGDTHLGGEDFDNRLVNHFVEEFKRKHK-KDI
H.sapiens        LIFDLGGGTFDVSILTIDDG-IFEVKATAGDTHLGGEDFDNRLVNHFVEEFKRKHK-KDI
I.tridecemlinea  LIFDLGGGTFDVSILTIDDG-IFEVKATAGDTHLGGEDFDNRLVNHFVEEFKRKHK-KDI
M.mulatta        LIFDLGGGTFDVSILTIDDG-IFEVKATAGDTHLGGEDFDNRLVNHFVEEFKRKHK-KDI
M.gallopavo      LIFDLGGGTFDVSILTIEDG-IFEVKSTAGDTHLGGEDFDNRMVNHFVEEFKRKHK-RDI
M.musculus       LIFDLGGGTFDVSILTIDDG-IFEVKATAGDTHLGGEDFDNRLVSHFVEEFKRKHK-KDI
O.sativa         LIFDLGGGTFDVSILTIEEG-IFEVKATAGDTHLGGEDFDNRMVNHFVQEFKRKHK-KDI
O.latipes        LIFDLGGGTFDVSILTIEDG-IFEVKATAGDTHLGGEDFDNRMVNHFITEFKRKFK-KEI
P.troglodytes    LIFDLGGGTFDVSILTIDDG-IFEVKATAGDTHLGGEDFDNRLVNHFVEEFKRKHK-KDI
P.falciparum     LIFDLGGGTFDVSLLTIEDG-IFEVKATAGDTHLGGEDFDNRLVNFCVEDFKRKNRGKDL
P.abelii         LIFDLGGGTFDVSILTIDDG-IFEVKATAGDTHLGGEDFDNRLVNHFVEEFKRKHK-KDI
S.cerevisiae     LIFDLGGGTFDVSLLSIEDG-IFEVKATAGDTHLGGEDFDNRLVNHFIQEFKRKNK-KDL
S.pombe          LIFDLGGGTFDVSLLTIEEG-IFEVKATAGDTHLGGEDFDSRLVNHFIQEFKRKNK-KDI
S.purpuratus     LIFDLGGGTFDVSILTIDEG-IFEVRSTAGDTHLGGEDFDNRLVTHLVEEFKRKNK-KDI
S.scrofa         LIFDLGGGTFDVSVLTIDAG-VFEVKATAGDTHLGGEDFDNRLVNHFMEEFRRKHR-KDL
T.rubripes       LIFDLGGGTFDVSILTIEDG-IFEVKATAGDTHLGGEDFDNRMVNHFVEEFKRKQK-KDI
X.tropicalis     LIFDLGGGTFDVSILTIDDG-IFEVKATAGDTHLGGEDFDNRMVNHFMEEFKRKHK-KDI
                 #################### ################################### ###


                       1330      1340      1350      1360      1370      1380
                 =========+=========+=========+=========+=========+=========+
A.carolinensis   AGNKRAVRRLRTACERAKRTLSSS--TQASIEIDSLFDG---IDFYTS-ITRARFEELNA
A.gambiae        STNKRALRRLRTACERAKRTLSSS--TQASIEIDSLFEG---TDFYTS-ITRARFEELNA
A.thaliana       SGNPRALRRLRTACERAKRTLSST--AQTTIEIDSLFDG---IDFYAP-ITRARFEELNI
B.distachyon     YQNARACIRLRVACEKLKKMLSAN--PEAPMNIECLMDE---KDVRGF-IKRDEFEQISG
C.elegans        SSNPRAIRRLRTACERAKRTLSSS--TEATVEVDSLFDG---TDFYSK-ITRARFEELCA
C.jacchus        SQNKRAVRRLRTACERAKRTLSSS--TQASLEIDSLFEG---IDFYTS-ITRARFEELCS
C.neoformans     SSNARALRRLRTACERAKRTLSSA--AQTSIEIDSLFDG---IDFYTS-ITRARFEELCQ
P.tetraurelia    TNDRQALQRLTEASEKAKVELSSL--TQVEINLPFITATNTGPKHLQKVLTRKSFEEICS
D.rerio          SQNKRALRRLRTACERAKRTLSSS--SQASIEIDSLYEG---IDFYTS-ITRARFEELCS
D.discoideum     MGNQRAVRRLRTACERAKRTLSSS--AQASIEIDSLFEG---IDFYTS-ITRARFEELCA
D.melanogaster   RSNPRALRRLRTAAERAKRTLSSS--TEATIEIDALFEG---QDFYTK-VSRARFEELCA
E.siliculosus    RTKPKALLKLLDAGEKAKKQLSPVGVTDAPINIECLWED---LDYNGR-LSLKQFEALIK
G.gallus         AGNKRAVRRLRTACERARRTLSSS--TQASIEIDSLFEG---IDFYTS-ITRARFEELNA
G.gorilla        SQNKRAVRRLRTACERAKRTLSSS--TQASLEIDSLFEG---IDFYTS-ITRARFEELCS
H.sapiens        SQNKRAVRRLRTACERAKRTLSSS--TQASLEIDSLFEG---IDFYTS-ITRARFEELCS
I.tridecemlinea  SQNKRAVRRLRTACERAKRTLSSS--TQANLEIDSLYEG---IDFYTS-ITRARFEELCA
M.mulatta        SQNKRAVRRLRTACERAKRTLSSS--TQASLEIDSLFEG---IDFYTS-ITRARFEELCS
M.gallopavo      AGNKRAVRRLRTACERAKRTLSSS--TQASIEIDSLFEG---IDFYTS-ITRARFEELNA
M.musculus       SQNKRAVRRLRTACERAKRTLSSS--TQASLEIDSLFEG---IDFYTS-ITRARFEELCS
O.sativa         TGNPRALRRLRTACERAKRTLSST--AQTTIEIESLYEG---IDFYAT-ITRARFEELNM
O.latipes        TNNKRAVRRLRTACERAKRTLSSS--TQASIEIDSLYEG---TDFYTS-ITRARFEELNA
P.troglodytes    SQNKRAVRRLRTACERAKRTLSSS--TQASLEIDSLFEG---IDFYTS-ITRARFEELCS
P.falciparum     SKNSRALRRLRTQCERAKRTLSSS--TQATIEIDSLFEG---IDYSVT-VSRARFEELCI
P.abelii         SQNKRAVRRLRTACERAKRTLSSS--TQASLEIDSLFEG---IDFYTS-ITRARFEELCS
S.cerevisiae     STNQRALRRLRTACERAKRTLSSS--AQTSVEIDSLFEG---IDFYTS-ITRARFEELCA
S.pombe          TGNARAVRRLRTACERAKRTLSSS--AQASIEIDSLFEG---IDFYTS-ITRARFEELCA
S.purpuratus     RSNPRALRRLRTAAERAKRTLSSS--TQASIEVDSLYEG---IDFYTS-VTRARFEELCS
S.scrofa         SRNKRALRRLRTACERAKRTLSSS--TQATLEIDSLFEG---VDFYTS-ITRARFEELCS
T.rubripes       SQNKRALRRLRTACERAKRTLSSS--TQASIEIDSLFEG---IDFYTS-ITRARFEELCG
X.tropicalis     SQNKRALRRLRTACERAKRTLSSS--SQASIEIDSLFEG---IDFYTS-ITRARFEELCS
                 ########################  #############          ###########


                       1390      1400      1410      1420      1430      1440
                 =========+=========+=========+=========+=========+=========+
A.carolinensis   DLFRGTLEPVEKALRDAKLDKGQINEIVLVGGSTRIPKIQKLLQDFFNGKE-LN------
A.gambiae        DLFRSTMEPVEKALRDAKMDKASIHDIVLVGGSTRIPKVQKLLQDFFNGKE-LN------
A.thaliana       DLFRKCMEPVEKCLRDAKMDKNSIDDVVLVGGSTRIPKVQQLLVDFFNGKE-LC------
B.distachyon     PVLERVKGPLEKALAEAGLTTESVHFVEVVGSGSRVPAIMRIITEFF-GKE-PR------
C.elegans        DLFRKTLEPVEKALRDAKTDKGRIDEVVLVGGSTRIPKIQKLLKEFFNGKD-LN------
C.jacchus        DLFRSTLEPVEKALRDAKLDKAQIHDLVLVGGSTRIPKVQKLLQDFFNGRD-LN------
C.neoformans     DLFRSTMDPVEKVLRDSKIDKSSVNEIVLVGGSTRIPKIQKLVSDMFSGRE-PN------
P.tetraurelia    TLINRVKIPIENALKDAKLDSKKIDEIVLVGGSSRIPAVKELVKKLL-GKE-LN------
D.rerio          DLFRGTLDPVEKALRDAKMDKAQIHDIVLVGGSTRIPKIQKLLQDFFNGRE-LN------
D.discoideum     DLFRGCLDPVEKVLKDSKLDKKSIHEIVLVGGSTRIPKVQQLLQEFFNGKE-LN------
D.melanogaster   DLFRNTLQPVEKALNDAKMDKGQIHDIVLVGGSTRIPKVQSLLQDFFHGKN-LN------
E.siliculosus    PIIDRMDAPILKALADAGVTKEQLGSVEIVGGSTRVPLVKSHLAELL-GRDKTAINFGLS
G.gallus         DLFRGTLEPVEKALRDAKLDKGQIQEIVLVGGSTRIPKIQKLLQDFFNGKE-LN------
G.gorilla        DLFRSTLEPVEKALRDAKLDKAQIHDLVLVGGSTRIPKVQKLLQDFFNGRD-LN------
H.sapiens        DLFRSTLEPVEKALRDAKLDKAQIHDLVLVGGSTRIPKVQKLLQDFFNGRD-LN------
I.tridecemlinea  DLFRGTLEPVEKALRDAKMDKAKIHDIVLVGGSTRIPKVQKLLQDYFNGRD-LN------
M.mulatta        DLFRSTLEPVEKALRDAKLDKAQIHDLVLVGGSTRIPKVQKLLQDFFNGRD-LN------
M.gallopavo      DLFRGTLEPVEKALRDAKLDKGQIQEIVLVGGSTRIPKIQKLLQDFFNGKE-LN------
M.musculus       DLFRGTLEPVEKALRDAKMDKAQIHDLVLVGGSTRIPKVQKLLQDFFNGRD-LN------
O.sativa         DLFRRCMEPVEKCLRDAKMDKAQIHDVVLVGGSTRIPKVQQLLQDFFNGKE-LC------
O.latipes        DLFRGTIEPVEKALRDAKMDKSQIHDVVLVGGSTRIPKIQKLLQDLFNGRD-LN------
P.troglodytes    DLFRSTLEPVEKALRDAKLDKAQIHDLVLVGGSTRIPKVQKLLQDFFNGRD-LN------
P.falciparum     DYFRDTLIPVEKVLKDAMMDKKSVHEVVLVGGSTRIPKIQTLIKEFFNGKE-AC------
P.abelii         DLFRSTLEPVEKALRDAKLDKAQIHDLVLVGGSTRIPKVQKLLQDFFNGRD-LN------
S.cerevisiae     DLFRSTLDPVEKVLRDAKLDKSQVDEIVLVGGSTRIPKVQKLVTDYFNGKE-PN------
S.pombe          DLFRKTMEPVERVLRDSKVDKASVNEIVLVGGSTRIPRVQKLVSDFFNGKE-PC------
S.purpuratus     DQFRKSLEPVERAIVDAKLDKNQIDTVVLVGGSTRIPKIQKLLQDYLNGKE-LN------
S.scrofa         DLFRSTLEPVEKALRDAKLDKAQIHDIVLVGGSTRIPKIQKLLQDFFNGRE-LN------
T.rubripes       DLFRGTLDPVEKALKDAKMDKGQINDIVLVGGSTRIPRIQKLLQDFFNGRE-LN------
X.tropicalis     DLFRGTLEPVEKALRDAKLDKSQIDEIVLVGGSTRIPKVQKLLQDFFNGRE-LN------
                 ###############################################             


                       1450      1460      1470      1480      1490      1500
                 =========+=========+=========+=========+=========+=========+
A.carolinensis   KSINPDEAVAYGAAVQAAILMGDKSENVQDLLLLDVAPLSLGIET------AGGVMTALI
A.gambiae        KSINPDEAVAYGAAVQAAILHGDKSEEVQDLLLLDVTPLSLGIET------AGGVMSVLI
A.thaliana       KSINPDEAVAYGAAVQAAILSGEGNEKVQDLLLLDVTPLSLGLET------AGGVMTVLI
B.distachyon     RTMNASECVARGCALQCAILS--PTFKVREFQVNEGFPFSIALSWKSDAQSNESQQTVVF
C.elegans        CSINPDEAVAFGAAVQAAVLSGVKDDTIKDVLLVDVVPLSHGIET------AGGVMTNLI
C.jacchus        KSINPDEAVAYGAAVQAAILMGDKSENVQDLLLLDVAPLSLGLET------AGGVMTALI
C.neoformans     RSINPDEAVAYGAAVQAAILTGDTSEATQDLLLLDVAPLSMGIET------AGGIMTPLI
P.tetraurelia    QTVNPDEVVAVGAAVQAGVLAGE----VKDLLLLDVTPLSLGVET------LGGVTTRII
D.rerio          KSINPDEAVAYGAAVQAAILMGDTSGNVQDLLLLDVAPLSLGIET------AGGVMTALI
D.discoideum     KSINPDEAVAYGAAVQAAILSNEGGAKVADLLLLDVAPLSMGLET------AGGVMTTLI
D.melanogaster   LSINPDEAVAYGAAVQAAILSGDQSGKIQDVLLVDVAPLSLGIET------AGGVMTKLI
E.siliculosus    CTLNADESIARGCALQCAMLS--KRLKGKDFLEKEIVPYPTKLTA------GKNS-VNIF
G.gallus         KSINPDEAVAYGAAVQAAILMGDKSENVQDLLLLDVTPLSLGIET------AGGVMTALI
G.gorilla        KSINPDEAVAYGAAVQAAILMGDKSENVQDLLLLDVAPLSLGLET------AGGVMTALI
H.sapiens        KSINPDEAVAYGAAVQAAILMGDKSENVQDLLLLDVAPLSLGLET------AGGVMTALI
I.tridecemlinea  KSINPDEAVAYGAAVQAAILMGDKSEKVQDLLLLDVAPLSLGLET------AGGVMTVLI
M.mulatta        KSINPDEAVAYGAAVQAAILMGDKSENVQDLLLLDVAPLSLGLET------AGGVMTALI
M.gallopavo      KSINPDEAVAYGAAVQAAILMGDKSENVQDLLLLDVTPLSLGIET------AGGVMTALI
M.musculus       KSINPDEAVAYGAAVQAAILMGDKSENVQDLLLLDVAPLSLGLET------AGGVMTALI
O.sativa         KSINPDEAVAYGAAVQAAILSGEGNQRVQDLLLLDVTPLSLGLET------AGGVMTVLI
O.latipes        KSINPDEAVAYGAAVQAAILAGDKSENVQDLLLLDVTPLSLGIET------AGGVMTVLI
P.troglodytes    KSINPDEAVAYGAAVQAAILMGDKSENVQDLLLLDVAPLSLGLET------AGGVMTALI
P.falciparum     RSINPDEAVAYGAAVQAAILSGDQSNAVQDLLLLDVCSLSLGLET------AGGVMTKLI
P.abelii         KSINPDEAVAYGAAVQAAILMGDKSENVQDLLLLDVAPLSLGLET------AGGVMTALI
S.cerevisiae     RSINPDEAVAYGAAVQAAILTGDESSKTQDLLLLDVAPLSLGIET------AGGVMTKLI
S.pombe          KSINPDEAVAYGAAVQAAVLTGDTSEKTQDLLLLDVAPLSMGIET------AGGVMTPLI
S.purpuratus     KSINPDEAVAYGAAVQAAILSGDQSSEVKDVLLVDVAPLSLGIET------AGGVMNKLI
S.scrofa         KSINPDEAVAYGAAVQAAVLMGDKCEKVQDLLLLDVAPLSLGLET------AGGVMTTLI
T.rubripes       KSINPDEAVAYGAAVQAAILTGDTSGNVQDLLLLVVAPLSLGFET------EGGVMPALI
X.tropicalis     KSINPDEAVAYGAAVQAAILMGDKSENVQDLLLLDVAPLSLGLET------AGGVMTVLI
                 ####################       ##################           ####


                       1510      1520      1530      1540      1550      1560
                 =========+=========+=========+=========+=========+=========+
A.carolinensis   KRNTTIPTKQTQTFTTYSDNQSSVLVQVYEGERAMTKD-NNLLGKFDLTG--IP------
A.gambiae        KRNTTIPTKQTQTFTTYSDNQPGVLIQVFEGERAMTKD-NNLLGKFELSG--IP------
A.thaliana       QRNTTIPTKKEQVFSTYSDNQPGVLIQVYEGERARTKD-NNLLGKFELSG--IP------
B.distachyon     PKGNPMPSIKALTF--YRSNTFAVDVLNVDTDDLQITQ-K--ISTYTIGP--FQ------
C.elegans        DRNTRIPAKASKTFTTYADNQPGVSIQVYEGERAMTRD-NHRLGTFELTG--IP------
C.jacchus        KRNSTIPTKQTQIFTTYSDNQPGVLIQVYEGERAMTKD-NNLLGRFELSG--IP------
C.neoformans     KRNTTVPTKKSEVFSTYSDNQPGVLIQVFEGERAKTKD-CNLLGKFDLSG--IP------
P.tetraurelia    ARNTTIPTKKSEVFSTAVDNQPNVEIHVLQGEREFAKD-NKSLGTFKLEG--IL------
D.rerio          KRNTTIPTKQTQTFSTYSDNQPGVLIQVFEGERAMTKD-NNLLGKFELTG--IP------
D.discoideum     PRNTTIPCKKTQTFSTYSDNQPGVLIQVYEGERAMTKD-NNLLGKFELSG--IP------
D.melanogaster   ERNCRIPCKQTKTFSTYADNQPGVSIQVYEGERAMTKD-NNALGTFDLSG--IP------
E.siliculosus    AKGDDTPKVRRVNL--VRDKPFTVTASYDAEAEKLPSGCASEIGTFKVNVQGLRRKAAAG
G.gallus         KRNTTIPTKQTQTFTTYSDNQSSVLVQVYEGERAMTKD-NNLLGKFDLTG--IP------
G.gorilla        KRNSTIPTKQTQIFTTYSDNQPGVLIQVYEGERAMTKD-NNLLGRFELSG--IP------
H.sapiens        KRNSTIPTKQTQIFTTYSDNQPGVLIQVYEGERAMTKD-NNLLGRFELSG--IP------
I.tridecemlinea  KRNSTIPTKQTQIFTTYSDNQPGVLIQVYEGERAMTRD-NNLLGRFDLTG--IP------
M.mulatta        KRNSTIPTKQTQIFTTYSDNQPGVLIQVYEGERAMTKD-NNLLGRFELSG--IP------
M.gallopavo      KRNTTIPTKQTQTFTTYSDNQSSVLVQVYEGERAMTKD-NNLLGKFDLTG--IP------
M.musculus       KRNSTIPTKQTQTFTTYSDNQPGVLIQVYEGERAMTRD-NNLLGRFELSG--IP------
O.sativa         PRNTTIPTKKEQVFSTYSDNQPGVLIQVYEGERTRTKD-NNLLGKFELTG--IP------
O.latipes        KRNTTIPTKQTQTFTTYSDNQPGVLIQVYEGERAMTKD-NNILGKFELTG--IP------
P.troglodytes    KRNSTIPTKQTQIFTTYSDNQPGVLIQVYEGERAMTKD-NNLLGRFELSG--IP------
P.falciparum     ERNTTIPAKKSQIFTTYADNQPGVLIQVYEGERALTKD-NNLLGKFHLDG--IP------
P.abelii         KRNSTIPTKQTQIFTTYSDNQPGVLIQVYEGERAMTKD-NNLLGRFEPSG--IP------
S.cerevisiae     PRNSTIPTKKSEIFSTYADNQPGVLIQVFEGERAKTKD-NNLLGKFELSG--IP------
S.pombe          KRNTTIPTKKSEIFSTYSDNQPGVLIQVFEGERARTKD-CNLLGKFELSG--IP------
S.purpuratus     ERNTRIPTKAQQTFTTYADNQSAVSIQVYEGERSMTKD-NNLLGNFELSG--IP------
S.scrofa         QRNATIPTKQTQTFTTYSDNQPGVLIQVYEGERAMTRD-NNLLGRFELSG--IP------
T.rubripes       KRNTTIPTKQTQIFTTYADNQPGVLIQVYEGERAMTKD-NNLLGKFELSG--LP------
X.tropicalis     KRNTTIPTKQTQTFTTYSDNQPGVLIQVFEGERAMTKD-NNLLGKFELSG--IP------
                 ##############  ######################                      


                       1570      1580      1590      1600      1610      1620
                 =========+=========+=========+=========+=========+=========+
A.carolinensis   -----PAPRGVPQIEVTFDIDANGILNVTAVD----------------------------
A.gambiae        -----PAPRGVPQIEVTFDIDANGILNVTALE----------------------------
A.thaliana       -----PAPRGVPQITVCFDIDANGILNVSAED----------------------------
B.distachyon     -----PSNGEKAKVKVKVRLNIHGIVSLESATMLEEDEVEVPVSSASEVPKDATKMDTDD
C.elegans        -----PAPRGVPQVDVTFDIDANGILNVSAAD----------------------------
C.jacchus        -----PAPRGVPQIEVTFDIDANGILNVTAMD----------------------------
C.neoformans     -----PAPRGVPQIEVSFDVDANGILNVNAAD----------------------------
P.tetraurelia    -----PAPRGIPQIEVTFDIDANGILSVTAKD----------------------------
D.rerio          -----PAPRGVPQIEVTFDIDANGILNVSAAD----------------------------
D.discoideum     -----PAPRGVPQVEVTFDVDANGILNVSAED----------------------------
D.melanogaster   -----PAPRGVPQIEVTFDLDANGILNVSAKE----------------------------
E.siliculosus    TLVDVPEGAGMSKIRVNVKHDIHGMFQVQSADMMQEVXXXXXXXXXXXXPTDAAAPAAAP
G.gallus         -----PAPRGVPQIEVTFDIDANGILNVSAVD----------------------------
G.gorilla        -----PAPRGVPQIEVTFDIDANGILNVTATD----------------------------
H.sapiens        -----PAPRGVPQIEVTFDIDANGILNVTATD----------------------------
I.tridecemlinea  -----PAPRGVPQIEVTFDIDANGILNVTAMD----------------------------
M.mulatta        -----PAPRGVPQIEVTFDIDANGILNVTATD----------------------------
M.gallopavo      -----PAPRGVPQIEVTFDIDANGILNVSAVD----------------------------
M.musculus       -----PAPRGVPQIEVTFDIDANGILNVTATD----------------------------
O.sativa         -----PAPRGVPQINVTFDIDANGILNVSAED----------------------------
O.latipes        -----PAPRGVPQIEVTFDIDANGILNVSAVD----------------------------
P.troglodytes    -----PAPRGVPQIEVTFDIDANGILNVTATD----------------------------
P.falciparum     -----PAPRKVPQIEVTFDIDANGILNVTAVE----------------------------
P.abelii         -----PAPRGVPQIEVTFDIDANGILNVTATD----------------------------
S.cerevisiae     -----PAPRGVPQIEVTFDVDSNGILNVSAVE----------------------------
S.pombe          -----PAPRGVPQIEVTFDVDANGILNVSALE----------------------------
S.purpuratus     -----PAPRGVPKIEITYDIDANGILNVTAKD----------------------------
S.scrofa         -----PAPRGVPQIEVTFDIDANGILSVTATD----------------------------
T.rubripes       -----PAPRGVPQIEVTFDIDANGILNVSAVD----------------------------
X.tropicalis     -----PAPRGVPQIEVTFDIDANGILNVSAVE----------------------------
                      ###########################                            


                       1630      1640      1650      1660      1670      1680
                 =========+=========+=========+=========+=========+=========+
A.carolinensis   ----------------KSTGKENKI-----------------------------------
A.gambiae        ----------------KSTNKENKI-----------------------------------
A.thaliana       ----------------KTTGQKNKI-----------------------------------
B.distachyon     AQ--------------RDPASGNDVNMEDSKGATDTAEGAVENGAHDSEEKSVPMDTDTK
C.elegans        ----------------KSTGRSNRI-----------------------------------
C.jacchus        ----------------KSTGKANKI-----------------------------------
C.neoformans     ----------------KSTGKSSKI-----------------------------------
P.tetraurelia    ----------------KGSGKEQSI-----------------------------------
D.rerio          ----------------KSTGKQNKI-----------------------------------
D.discoideum     ----------------KSTGNKQKI-----------------------------------
D.melanogaster   ----------------MSTGKAKNI-----------------------------------
E.siliculosus    MDVDGENATGEKAAAPEANGSASPT-------------------SPTENGGAPAAVAEAK
G.gallus         ----------------KSTGKENKI-----------------------------------
G.gorilla        ----------------KSTGKANKI-----------------------------------
H.sapiens        ----------------KSTGKANKI-----------------------------------
I.tridecemlinea  ----------------KSTGKANKI-----------------------------------
M.mulatta        ----------------KSTGKANKI-----------------------------------
M.gallopavo      ----------------KSTGKENKI-----------------------------------
M.musculus       ----------------KSTGKANKI-----------------------------------
O.sativa         ----------------KTTGKKNKI-----------------------------------
O.latipes        ----------------KSTGKENKI-----------------------------------
P.troglodytes    ----------------KSTGKANKI-----------------------------------
P.falciparum     ----------------KSTGKQNHI-----------------------------------
P.abelii         ----------------KSTGKANKI-----------------------------------
S.cerevisiae     ----------------KGTGKSNKI-----------------------------------
S.pombe          ----------------KGTGKTQKI-----------------------------------
S.purpuratus     ----------------ESTGRTNKI-----------------------------------
S.scrofa         ----------------RSTGRANKI-----------------------------------
T.rubripes       ----------------KSTGKENKI-----------------------------------
X.tropicalis     ----------------KSSGKQNKI-----------------------------------
                                                                             


                       1690      1700      1710      1720      1730      1740
                 =========+=========+=========+=========+=========+=========+
A.carolinensis   ----------------TITNDKGRLSKDDIDRMVQEAERYKVEDESNRERVVSKNALESY
A.gambiae        ----------------TITNDKGRLSKEDIERMVNEAEKYRTEDEKQKETISAKNALESY
A.thaliana       ----------------TITNDKGRLSKDEIEKMVQEAEKYKSEDEEHKKKVDAKNALENY
B.distachyon     VQPS-KKRV-KKTNVPIAELVYGTLGADELEKAVEKEYEMALQDRVMEETKEKKNSVEAY
C.elegans        ----------------TIRNEKGRLSQADIDRMVNEAKQFEREDAAQRDRISSRNQLEAY
C.jacchus        ----------------TITNDKGRLSKEEIERMVQEAEKYKAEDEVQRERVSAKNALESY
C.neoformans     ----------------TITNDKGRLSKEEIERMLAEAEKFKAEDEAAAATVQAKNGLESY
P.tetraurelia    ----------------TISGSS-TLPTEEVERMVQESQQTAAQDKEKREKIDLKNQADSL
D.rerio          ----------------TITNDKGRLSKEEIERMVQEADNYKAEDDLQREKISAKNSLESY
D.discoideum     ----------------TITNDKGRLSKEEIEKMVADAEKFKQQDEQQKDRVESKNKLENY
D.melanogaster   ----------------TIKNDKGRLSQAEIDRMVNEAEKYADEDEKHRQRITSRNALESY
E.siliculosus    GDEAPKKKVYTKVPL-KIDSKTSAWSKSEIDRAVEMEAQMANQDRVLKETADKRNELESY
G.gallus         ----------------TITNDKGRLSKDDIDRMVQEAEKYKAEDEANRDRVGAKNSLESY
G.gorilla        ----------------TITNDKGRLSKEEIERMVQEAEKYKAEDEVQRERVSAKNALESY
H.sapiens        ----------------TITNDKGRLSKEEIERMVQEAEKYKAEDEVQRERVSAKNALESY
I.tridecemlinea  ----------------TITNDKGRLTKEEIERMVLDAEKYKAEDEMQREKIAAKNALESY
M.mulatta        ----------------TITNDKGRLSKEEIERMVQEAEKYKAEDEVQRERVSAKNALESY
M.gallopavo      ----------------TITNDKGRLSKDDIDRMVQEAEKYKAEDEANRDRVGAKNSLESY
M.musculus       ----------------TITNDKGRLSKEEIERMVQEAERYKAEDEVQRDRVAAKNALESY
O.sativa         ----------------TITNDKGRLSKEEIERMVQEAEKYKAEDEQVRHKVEARNALENY
O.latipes        ----------------TITNDKGRLSKEDIERMVQEAEQFKAEDESQRDKITAKNSLESL
P.troglodytes    ----------------TITNDKGRLSKEEIERMVQEAEKYKAEDEVQRERVSAKNALESY
P.falciparum     ----------------TITNDKGRLSQDEIDRMVNDAEKYKAEDEENRKRIEARNSLENY
P.abelii         ----------------TITNDKGRLSKEEIERMVQEAEKYKAEDEVQRERVSAKNALESY
S.cerevisiae     ----------------TITNDKGRLSKEDIEKMVAEAEKFKEEDEKESQRIASKNQLESI
S.pombe          ----------------TITNDKGRLSKEEIDRMVAEAEKYKAEDEAESGRIQAKNHLESY
S.purpuratus     ----------------TISNDKGRLSKADIDRMVNDADKYKAEDEAQFARVSARNQLESY
S.scrofa         ----------------TITNDKGRLSKEEVERMVREADEYKVEDEAQRDRVAAKNSLEAY
T.rubripes       ----------------TITNDKGRLSKEEIERMVQDADKYKAEDDQQREKIAAKNSLESY
X.tropicalis     ----------------TITNDKGRLSKEDIEKMVQEAERYKADDDAQREKIDAKNSLESY
                                        #####################################


                       1750      1760      1770      1780      1790      1800
                 =========+=========+=========+=========+=========+=========+
A.carolinensis   AYNIKQTVED--------------------------------------------------
A.gambiae        CFNMKATMED--------------------------------------------------
A.thaliana       AYNMRNTIRD--------------------------------------------------
B.distachyon     VYEMRNKLSEKYNDFVMSEDMEVLMAKLQEVEDWLYED-GEDETKGVYVAKLEELKKVGG
C.elegans        AFQVKQALEE--------------------------------------------------
C.jacchus        AFNMKSAVED--------------------------------------------------
C.neoformans     SYSLKTTLSD--------------------------------------------------
P.tetraurelia    CYQAEKQVSE--------------------------------------------------
D.rerio          AFNMKNSVED--------------------------------------------------
D.discoideum     AFTVKNSIKD--------------------------------------------------
D.melanogaster   VFNVKQAVEQ--------------------------------------------------
E.siliculosus    VYAMRDKLVGSLRTYIEGEEADKFGSSLTAAEDWLYSDEGFDSTKSVYAAKLKELMDLGN
G.gallus         TYNMKQTVED--------------------------------------------------
G.gorilla        AFNMKSAVED--------------------------------------------------
H.sapiens        AFNMKSAVED--------------------------------------------------
I.tridecemlinea  AFNMKSAVSD--------------------------------------------------
M.mulatta        AFNMKSAVED--------------------------------------------------
M.gallopavo      TYNMKQTVED--------------------------------------------------
M.musculus       AFNMKSAVED--------------------------------------------------
O.sativa         AYNMRNTVRD--------------------------------------------------
O.latipes        AFNMKSTVDD--------------------------------------------------
P.troglodytes    AFNMKSAVED--------------------------------------------------
P.falciparum     CYGVKSSLED--------------------------------------------------
P.abelii         AFNMKSAVED--------------------------------------------------
S.cerevisiae     AYSLKNTISE--------------------------------------------------
S.pombe          AYSLRNSLDD--------------------------------------------------
S.purpuratus     AFGMKSTVND--------------------------------------------------
S.scrofa         VFHVKGSLHE--------------------------------------------------
T.rubripes       AFNMKSSVQE--------------------------------------------------
X.tropicalis     AFNLKSMVED--------------------------------------------------
                 #####                                                       


                       1810      1820      1830      1840      1850      1860
                 =========+=========+=========+=========+=========+=========+
A.carolinensis   ---------------------------------EKLKGKIGEQDKQRILEKCQEVISWLD
A.gambiae        ---------------------------------DKLKDKITDSDKTLVLDKCNDTIKWLD
A.thaliana       ---------------------------------EKIGEKLAGDDKKKIEDSIEAAIEWLE
B.distachyon     PIEMRYKEWSERGQALEQLVYCIRSFREAALSSDQKFDHIDISEKQKVVNECSGAETWLL
C.elegans        ---------------------------------HG--SLLSAEDAKRAKDAVEDTLRWME
C.jacchus        ---------------------------------EGLKGKISEADKKKVLDKCQEVISWLD
C.neoformans     ---------------------------------N--QDKFDAADHETLSKKVDEVISSLD
P.tetraurelia    ---------------------------------L--GDSLSLSDKNKLTDLVGKLREAIG
D.rerio          ---------------------------------DNLKGKISEEDKKRVIEKCNEAVSWLE
D.discoideum     ---------------------------------EKVAAKISDSDKSTIESETESVLKWLE
D.melanogaster   ---------------------------------AP-AGKLDEADKNSVLDKCNDTIRWLD
E.siliculosus    PVESRFYEANNRQGAATELQKAIDGYMKFANSSDEAYAHIEAEEKAKARDCAKKAEKWLF
G.gallus         ---------------------------------EKLKGKISDQDKQKVLDKCQEVISSLD
G.gorilla        ---------------------------------EGLKGKISEADKKKVLDKCQEVISWLD
H.sapiens        ---------------------------------EGLKGKISEADKKKVLDKCQEVISWLD
I.tridecemlinea  ---------------------------------EGLKDKISESDKKKILSKCNEVLSWLE
M.mulatta        ---------------------------------EGLKGKISEADKKKVLDKCQEVISWLD
M.gallopavo      ---------------------------------DKLKGKISDQDKQKVLDKCREVISWLD
M.musculus       ---------------------------------EGLKGKLSEADKKKVLDKCQEVISWLD
O.sativa         ---------------------------------EKIASKLPADDKKKIEDAIEDAIKWLD
O.latipes        ---------------------------------EKLQDKISSEDKKTIVDKCNEIIAWLD
P.troglodytes    ---------------------------------EGLKGKISEADKKKVLDKCQEVISWLD
P.falciparum     ---------------------------------QKIKEKLQPAEIETCMKTITTILEWLE
P.abelii         ---------------------------------EGLKGKISEADKKKVLDKCQEVISWLD
S.cerevisiae     ---------------------------------A--GDKLEQADKDTVTKKAEETISWLD
S.pombe          ---------------------------------PNLKDKVDASDKETVDKAVKETIEWLD
S.purpuratus     ---------------------------------PALESKLSAEDKQTILKAVEDTIQWLE
S.scrofa         ---------------------------------ESLRDKIPEEDRCKVQDKCQEVLTWLE
T.rubripes       ---------------------------------DNLKDKMSEEDKKKVVGKCEETIAWLE
X.tropicalis     ---------------------------------ENMKGKISEGDKRIISEKCTQIISWLE
                                                       ######################


                       1870      1880      1890      1900      1910      1920
                 =========+=========+=========+=========+=========+=========+
A.carolinensis   RNQM------------AEKEEFEHKQKELEKLCNPIIAKLYQGA----------------
A.gambiae        ANQL------------ADKEEYEHRQKELESVCNPIISKLYQGA----------------
A.thaliana       ANQL------------AECDEFEDKMKELESICNPIIAKMYQGGE-AGG-PAA--G----
B.distachyon     EKKQQQDALPKHVNPVLLVSDIKKKAEALDRFCKPIMTKPKPAPK-PQTPPPAENPAPEA
C.elegans        RNTL------------ADKDEIEAKDKELKSICQDILTKMHQQEA-QSGSGCG--N----
C.jacchus        ANTL------------AEKDEFEHKRKELEQVCNPIISGLYQGAG-GPG---P--G----
C.neoformans     TMQS------------ASKEEFESLQKELEAVANPIMTKFY---G-AQG-GAP--G----
P.tetraurelia    SDSY---------------EQIRILNKNIQDSLMDIGKKIYSGKD-HGK---S--Q----
D.rerio          NNQL------------ADKEEYEHQLKELEKVCNPVISKLYQG-G-MPA-G---------
D.discoideum     SNQT------------AEKDEYEDKMKALEAVVNPIMSKLYQEG------GMP--Q----
D.melanogaster   SNTT------------AEKEEFDHKLEELTRHCSPIMTKMHQQGA-GAGAGGP--G----
E.siliculosus    QKLDQQANVPQSKNPIVTCDEINKQVVAVHATCRSIMNTPKPAPK-PAEPPAAAPTKEEA
G.gallus         RNQM------------AEKEEYEHKQKELEKLCNPIVTKLYQGA----------------
G.gorilla        ANTL------------AEKDEFEHKRKELEQVCNPIISGLYQGAG-GPG---P--G----
H.sapiens        ANTL------------AEKDEFEHKRKELEQVCNPIISGLYQGAG-GPG---P--G----
I.tridecemlinea  ANQL------------AEKDEYDHKRKELEQVCNPIITKLYQG-G-CTG---P--T----
M.mulatta        ANTL------------AEKDEFEHKRKELEQVCNPIISGLYQGAG-GPG---P--G----
M.gallopavo      RNQM------------AEKEEYEHKQKELEKLCNPIVTKLYQGA----------------
M.musculus       SNTL------------ADKEEFVHKREELERVCSPIISGLYQGAG-APG---A--G----
O.sativa         GNQL------------AEADEFEDKMKELESLCNPIISKMYQGG--AGG-PAG-------
O.latipes        KNQM------------AEKDEYEHQQKELEKVCNPIISKLYQGGMPGGM---P--G----
P.troglodytes    ANTL------------AEKDEFEHKRKELEQVCNPIISGLYQGAS-GPG---P--G----
P.falciparum     KNQL------------AGKDEYEAKQKEAESVCAPIMSKIYQDAA-GAAGGMP--G----
P.abelii         ANTL------------AEKDEFEHKRKELEQVCNPIISGLYQGAG-GPG---P--G----
S.cerevisiae     SNTT------------ASKEEFDDKLKELQDIANPIMSKLYQAGG-APG-GAA--G----
S.pombe          SNTT------------AAKDEFEAKQKELESVANPIMAKIYQAGG-APG-GMP--G----
S.purpuratus     SNSL------------ADKDEFKFKTDELQKKCSPIMAKLHQGS----------------
S.scrofa         HNQL------------AEKEEYEHQKRELEQICRPIFSRLYGAPG-IPG---G--S----
T.rubripes       NNQL------------ADKEEYQHQQKELEKVCNPIISNLYQG-G-MPG-G---------
X.tropicalis     NNQL------------AEKDEYAFQQKELERVCQPIITKLYQG-S-MPG-SMP--G----
                 ##                  #####################                   


                       1930      1940      1950      1960      1970      1980
                 =========+=========+=========+=========+=========+=========+
A.carolinensis   ----------GAA-G---------------------------------------------
A.gambiae        ----------GGAPG-----------------------------GMPGFPGGAP--GAGG
A.thaliana       -----G-MDEDVP-P---------------------------------------------
B.distachyon     QT---PEQQSSGA-S------------------------------------E--------
C.elegans        -----P-GSGGFH-----------------------------------------------
C.jacchus        -----G-FGAQGP-----------------------------------------------
C.neoformans     -----G-APGGFP-G------------------------------------A--------
P.tetraurelia    -----A-YNGNED-----------------------------------------------
D.rerio          ------GCGAQAR-----------------------------------------------
D.discoideum     -----G---GGMP-G------------------------------------G--------
D.melanogaster   -----ANCGQQAG-----------------------------------------------
E.siliculosus    KPAGGE-GDAAAA-AASGEKGGESAADASSAKAEGEAAAAEGGGAPKSGDSMDADTTPQ-
G.gallus         ----------GGA-G---------------------------------------------
G.gorilla        -----G-FGAQGP-----------------------------------------------
H.sapiens        -----G-FGAQGP-----------------------------------------------
I.tridecemlinea  -------CGTGYT-----------------------------------------------
M.mulatta        -----G-FGAQGP-----------------------------------------------
M.gallopavo      ----------GGA-G---------------------------------------------
M.musculus       -----G-FGAQAP-----------------------------------------------
O.sativa         -------MDEDAP-N------------------------------------G--------
O.latipes        -----G-TPGGFS-G---------------------------------------------
P.troglodytes    -----G-FGAQGP-----------------------------------------------
P.falciparum     -----G-MPGGMP-G-----------------------------G---------------
P.abelii         -----G-FGAQGP-----------------------------------------------
S.cerevisiae     -----G-APGGFP-G------------------------------------G--------
S.pombe          -----A-APGAAP-G------------------------------------A--------
S.purpuratus     -------GGGS-G-----------------------------------------------
S.scrofa         -----S-CGAQAR-----------------------------------------------
T.rubripes       ------NPGGQ-------------------------------------------------
X.tropicalis     -----SSCGAQA------------------------------------------------
                                                                             


                       1990      2000      2010      2020      2030      2040
                 =========+=========+=========+=========+=========+=========+
A.carolinensis   ------------------------------AGAPGGG--------PTIEEV---------
A.gambiae        AAGGA--A---------------------GGAGSGSG--------PTIEEV---------
A.thaliana       -------SAG------------------------GAG--------PKIEEV---------
B.distachyon     -------ADE--PA---------------NEGASQDQ--------PAAEQMETDRAEPSS
C.elegans        ------------------------------SSNYPQG--------PTVEEV---------
C.jacchus        ------------------------------KGGSGSG--------PTIEEV---------
C.neoformans     -------G---------------------GAPAQEEG--------PSVEEV---------
P.tetraurelia    ------------------------------GSVID----------TNYSET---------
D.rerio          -------G---------------------ASGASAQG--------PTIEEV---------
D.discoideum     -------MSN--------------------DSPKSSN--------NKVDEL---------
D.melanogaster   ------------------------------GFGGYSG--------PTVEEV---------
E.siliculosus    -EAAPPADGVPPS----------------KTTAATAEPVPTPMETDEVEQL---------
G.gallus         ------------------------------AGGSGG---------PTIEEV---------
G.gorilla        ------------------------------KGGSGSG--------PTIEEV---------
H.sapiens        ------------------------------KGGSGSG--------PTIEEV---------
I.tridecemlinea  ------------------------------PGRPATG--------PTIEEV---------
M.mulatta        ------------------------------KGGSGSG--------PTIEEV---------
M.gallopavo      ------------------------------AGGSGG---------PTIEEV---------
M.musculus       ------------------------------KGASGSG--------PTIEEV---------
O.sativa         -------SAG-------------------TGGGSGAG--------PKIEEV---------
O.latipes        ------------------------------GAGASSG--------PTIEEV---------
P.troglodytes    ------------------------------KGGSGSG--------PTIEEV---------
P.falciparum     -------MPSG-MPGGMNFPGGMPGAGMPGNAPAGSG--------PTVEEV---------
P.abelii         ------------------------------KGGSGSG--------PTIEEV---------
S.cerevisiae     -------A---------------------PPAPEAEG--------PTVEEV---------
S.pombe          -------APG-------------------AAPGGDNG--------PEVEEV---------
S.purpuratus     ------------------------------QQQPAGG--------PRVEEV---------
S.scrofa         ------------------------------QGAPSTG--------PVIEEV---------
T.rubripes       ------------------------------SQSSSQG--------PTIEEV---------
X.tropicalis     ------------------------------RQGGSSG--------PTIEEV---------
                                                                             


                       2050      2060      2070      2080      2090      2100
                 =========+=========+=========+=========+=========+=========+
A.carolinensis   D-----------------------------------------------------------
A.gambiae        D-----------------------------------------------------------
A.thaliana       D-----------------------------------------------------------
B.distachyon     A-----------------------------------------------------------
C.elegans        D-----------------------------------------------------------
C.jacchus        DMEEQGKPLPSKYWARQEDPATASRDKYLQEQFLLFGPSMDSPHAVHIEVTTMDESPDIQ
C.neoformans     D-----------------------------------------------------------
P.tetraurelia    N-----------------------------------------------------------
D.rerio          D-----------------------------------------------------------
D.discoideum     D-----------------------------------------------------------
D.melanogaster   DMAQP-------------------------------------------------------
E.siliculosus    E-----------------------------------------------------------
G.gallus         D-----------------------------------------------------------
G.gorilla        D-----------------------------------------------------------
H.sapiens        D-----------------------------------------------------------
I.tridecemlinea  DMEDHVEILKIVL-------------AWS-------------------------------
M.mulatta        D-----------------------------------------------------------
M.gallopavo      D-----------------------------------------------------------
M.musculus       D-----------------------------------------------------------
O.sativa         D-----------------------------------------------------------
O.latipes        D-----------------------------------------------------------
P.troglodytes    D-----------------------------------------------------------
P.falciparum     D-----------------------------------------------------------
P.abelii         D-----------------------------------------------------------
S.cerevisiae     D-----------------------------------------------------------
S.pombe          D-----------------------------------------------------------
S.purpuratus     D-----------------------------------------------------------
S.scrofa         D-----------------------------------------------------------
T.rubripes       D-----------------------------------------------------------
X.tropicalis     D-----------------------------------------------------------
                                                                             


                       2110      2120      2130      2140      2150      2160
                 =========+=========+=========+=========+=========+=========+
A.carolinensis   ------------------------------------------------------------
A.gambiae        ------------------------------------------------------------
A.thaliana       ------------------------------------------------------------
B.distachyon     ------------------------------------------------------------
C.elegans        ------------------------------------------------------------
C.jacchus        QAHSGDRSLPYKEAREWSRVASSSESSPWQQMQKPSPLMSVLCFAKELSLCPHNEGSRLG
C.neoformans     ------------------------------------------------------------
P.tetraurelia    ------------------------------------------------------------
D.rerio          ------------------------------------------------------------
D.discoideum     ------------------------------------------------------------
D.melanogaster   ------------------------------------------------------------
E.siliculosus    ------------------------------------------------------------
G.gallus         ------------------------------------------------------------
G.gorilla        ------------------------------------------------------------
H.sapiens        ------------------------------------------------------------
I.tridecemlinea  -------FICLFGAPDEDT-----------------------------------------
M.mulatta        ------------------------------------------------------------
M.gallopavo      ------------------------------------------------------------
M.musculus       ------------------------------------------------------------
O.sativa         ------------------------------------------------------------
O.latipes        ------------------------------------------------------------
P.troglodytes    ------------------------------------------------------------
P.falciparum     ------------------------------------------------------------
P.abelii         ------------------------------------------------------------
S.cerevisiae     ------------------------------------------------------------
S.pombe          ------------------------------------------------------------
S.purpuratus     ------------------------------------------------------------
S.scrofa         ------------------------------------------------------------
T.rubripes       ------------------------------------------------------------
X.tropicalis     ------------------------------------------------------------
                                                                             


                       2170      2180      2190      2200      2210      2220
                 =========+=========+=========+=========+=========+=========+
A.carolinensis   ------------------------------------------------------------
A.gambiae        ------------------------------------------------------------
A.thaliana       ------------------------------------------------------------
B.distachyon     ------------------------------------------------------------
C.elegans        ------------------------------------------------------------
C.jacchus        HAAKSSKVFGKQQFMELEAKTLAVCSEDKAHSMKPYTPRPQSFRYTVLCFLTFTTLLAPY
C.neoformans     ------------------------------------------------------------
P.tetraurelia    -----------------------------------MD-----------------------
D.rerio          ------------------------------------------------------------
D.discoideum     ------------------------------------------------------------
D.melanogaster   ------------------------------------------------------------
E.siliculosus    ------------------------------------------------------------
G.gallus         ------------------------------------------------------------
G.gorilla        ------------------------------------------------------------
H.sapiens        ------------------------------------------------------------
I.tridecemlinea  ------KLLDGLD-----------------------------------------------
M.mulatta        ------------------------------------------------------------
M.gallopavo      ------------------------------------------------------------
M.musculus       ------------------------------------------------------------
O.sativa         ------------------------------------------------------------
O.latipes        ------------------------------------------------------------
P.troglodytes    ------------------------------------------------------------
P.falciparum     ------------------------------------------------------------
P.abelii         ------------------------------------------------------------
S.cerevisiae     ------------------------------------------------------------
S.pombe          ------------------------------------------------------------
S.purpuratus     ------------------------------------------------------------
S.scrofa         ------------------------------------------------------------
T.rubripes       ------------------------------------------------------------
X.tropicalis     ------------------------------------------------------------
                                                                             


                       2230      2240      2250      2260      2270      2280
                 =========+=========+=========+=========+=========+=========+
A.carolinensis   ------------------------------------------------------------
A.gambiae        ------------------------------------------------------------
A.thaliana       ------------------------------------------------------------
B.distachyon     ------------------------------------------------------------
C.elegans        ------------------------------------------------------------
C.jacchus        GPEKKLAPTLLKAPEKEVPSSPASPKCHEQHNIHSLFPRPILTPGTACAAFSRARGAWKH
C.neoformans     ------------------------------------------------------------
P.tetraurelia    ------------------------------------------------------------
D.rerio          ------------------------------------------------------------
D.discoideum     ------------------------------------------------------------
D.melanogaster   ------------------------------------------------------------
E.siliculosus    ------------------------------------------------------------
G.gallus         ------------------------------------------------------------
G.gorilla        ------------------------------------------------------------
H.sapiens        ------------------------------------------------------------
I.tridecemlinea  ---------MLLAP----------------------------------------------
M.mulatta        ------------------------------------------------------------
M.gallopavo      ------------------------------------------------------------
M.musculus       ------------------------------------------------------------
O.sativa         ------------------------------------------------------------
O.latipes        ------------------------------------------------------------
P.troglodytes    ------------------------------------------------------------
P.falciparum     ------------------------------------------------------------
P.abelii         ------------------------------------------------------------
S.cerevisiae     ------------------------------------------------------------
S.pombe          ------------------------------------------------------------
S.purpuratus     ------------------------------------------------------------
S.scrofa         ------------------------------------------------------------
T.rubripes       ------------------------------------------------------------
X.tropicalis     ------------------------------------------------------------
                                                                             


                       2290      2300      2310      2320      2330      2340
                 =========+=========+=========+=========+=========+=========+
A.carolinensis   ------------------------------------------------------------
A.gambiae        ------------------------------------------------------------
A.thaliana       ------------------------------------------------------------
B.distachyon     ------------------------------------------------------------
C.elegans        ------------------------------------------------------------
C.jacchus        HFRGGPGGRGEAGGGGARQFRAGPLGPQPPNRVLDYIWSKLGASRARGRASGFGASAAAA
C.neoformans     ------------------------------------------------------------
P.tetraurelia    ----------I-----CAFERSFFF-----------------------------------
D.rerio          ------------------------------------------------------------
D.discoideum     ------------------------------------------------------------
D.melanogaster   ------------------------------------------------------------
E.siliculosus    ------------------------------------------------------------
G.gallus         ------------------------------------------------------------
G.gorilla        ------------------------------------------------------------
H.sapiens        ------------------------------------------------------------
I.tridecemlinea  -----------------------KIC------------------------------PRR-
M.mulatta        ------------------------------------------------------------
M.gallopavo      ------------------------------------------------------------
M.musculus       ------------------------------------------------------------
O.sativa         ------------------------------------------------------------
O.latipes        ------------------------------------------------------------
P.troglodytes    ------------------------------------------------------------
P.falciparum     ------------------------------------------------------------
P.abelii         ------------------------------------------------------------
S.cerevisiae     ------------------------------------------------------------
S.pombe          ------------------------------------------------------------
S.purpuratus     ------------------------------------------------------------
S.scrofa         ------------------------------------------------------------
T.rubripes       ------------------------------------------------------------
X.tropicalis     ------------------------------------------------------------
                                                                             


                       2350      2360      2370      2380      2390      2400
                 =========+=========+=========+=========+=========+=========+
A.carolinensis   ---------------------------------------------MSIEIESSDVI---R
A.gambiae        ---------------------------------------------MA-------------
A.thaliana       ------------------------------------------------------------
B.distachyon     ----------------------------------------------M-------------
C.elegans        --------------------------------------------MSE-------------
C.jacchus        RRAGASWPRSPASDRPLHARHARRRLPRAGSGGFRRARRGNKWARMP-------------
C.neoformans     ---------------------------------------------MS-------------
P.tetraurelia    ------------------------------------------------------------
D.rerio          ---------------------------------------------MS-------------
D.discoideum     ---------------------------------------------MS-------------
D.melanogaster   -------------------------------------------QPPA-------------
E.siliculosus    -------------------------------------------MSSS-------------
G.gallus         -------------------------------------------MRMP-------------
G.gorilla        ---------------------------------------------MP-------------
H.sapiens        ---------------------------------------------MP-------------
I.tridecemlinea  -------------------------Q-------------------KL-------------
M.mulatta        ------------------------------------------------------------
M.gallopavo      ------------------------------------------------------------
M.musculus       ---------------------------------------------MP-------------
O.sativa         ----------------------------------------------M-------------
O.latipes        ---------------------------------------------MP-------------
P.troglodytes    ---------------------------------------------MP-------------
P.falciparum     ---------------------------------------------MD---NIFINSPIIK
P.abelii         ---------------------------------------------MP-------------
S.cerevisiae     --------------------------------------------MSS-------------
S.pombe          ---------------------------------------------MS-------------
S.purpuratus     ------------------------------------------------------------
S.scrofa         ------------------------------------------------------------
T.rubripes       ---------------------------------------------MATEMEPTDVI---R
X.tropicalis     ---------------------------------------------MSIEIESSDVI---R
                                                                             


                       2410      2420      2430      2440      2450      2460
                 =========+=========+=========+=========+=========+=========+
A.carolinensis   LIMQYLKENSLHRAL-----------ATLQEETTVSLNTVDSIESFVADINSGHWDTVLQ
A.gambiae        -----YSNKFIYATL-----------PRTQRGQPIVLGGDPKGKNFLYT---NGHSVIIR
A.thaliana       -----MELSETYACV-----------PSTERGRGILISGNSKSDTILYT---NGRSVVTL
B.distachyon     -----AQLQETYACS-----------PATERGRGILLGGDPKTDTIAYC---AGRSVIIR
C.elegans        -----FSQTAVFPSL-----------PRAARGVPVILGSSPAGDKILYC---NGNSVFTI
C.jacchus        -----YEIKKVFASL-----------PQVERGVSKIIGGDPKGNNFLYT---NGKCVILR
C.neoformans     -----YKAGSVYPCN-----------PATSRSESTKLGVDPKGEKLVYT---NGRAVVIR
P.tetraurelia    -------------------------------GKIHKV-----LSFL----SG--------
D.rerio          -----YELKRVFASL-----------PHMERGVAKVLGGDPKGNNFLYT---NGKSVIIR
D.discoideum     -----VTLKNIIAPT-----------PATTRGKSVAINGDPKGENIVYA---SGSSIIIR
D.melanogaster   -----YENKNIYATL-----------PRTQRGQPIVLGADPKGKNFLYT---NGNSVIIR
E.siliculosus    -----YKRLTI-KRF-----------PRVQERET-------------------AEARYWR
G.gallus         -----YEIKKVFASL-----------PQVERGVSKIIGGDPKGNNFLYT---NGKCVVIR
G.gorilla        -----YEIKKVFASL-----------PQVERGVSKIIGGDPKGNNFLYT---NGKCVILR
H.sapiens        -----YEIKKVFASL-----------PQVERGVSKIIGGDPKGNNFLYT---NGKCVILR
I.tridecemlinea  -----QELEKVFASL-----------PQVERGVSKILGGDPKGNNFLYT---NGKCVILR
M.mulatta        ------------------------------------------------------------
M.gallopavo      -------MEKVFASL-----------PQVERGVSKIIGGDPKGNNFLYT---NGKCVVIR
M.musculus       -----YEIKKVFASL-----------PQVERGVSKILGGDPKGDHFLYT---NGKCVILR
O.sativa         -----AQLQETYACS-----------PATERGRGILLAGDPKTETIAYC---TGRSVIIR
O.latipes        -----YELKHVFASL-----------PQMERGVAKVIGGDPKGNNFLYT---NGKCVIIR
P.troglodytes    -----YEIKKVFASL-----------PQVERGVSKIIGGDPKGNNFLYT---NGKCVILR
P.falciparum     DY---YNIKDFFLHIDNEIHLTELPFPCDYNTYGHFCLSNEEINKIMCT---EENRFEEY
P.abelii         -----YEIKKVFASL-----------PQVERGVSKIIGGDPKGNNFLYT---NGKCVILR
S.cerevisiae     -----ISLKEIIPPQ-----------PSTQRNFTTHLSYDPTTNAIAYP---CGKSAFVR
S.pombe          -----SQLKSTWAPV-----------PSTKPSQPCKIGTDFKGERIVYP---ANKAIIIR
S.purpuratus     ---------------------------------------------MLYL---NGNNVIIR
S.scrofa         ------------------------------------------------------------
T.rubripes       LIMQYLKENNLYRTL-----------TTLQEETTLSLNTVESLDSFIADINCGHWDSVLM
X.tropicalis     LIMQYLKENSLHRTL-----------ATLQEETTVSLNTVDSIESFVADINSGHWDTVLQ
                                                                             


                       2470      2480      2490      2500      2510      2520
                 =========+=========+=========+=========+=========+=========+
A.carolinensis   AIQSLKLPDKT-----LIDLYEQVVLELIELRELG----------AARSLLRQTDPMIML
A.gambiae        NIDN-----PE-----IADIYTEHSCAVNVAKYSPS--GFYIASGDASGKIRIWD-----
A.thaliana       DLNN-----PL-----KVSIYGEHAYPATVARYSPN--GEWIASGDVSGTVRIWG-----
B.distachyon     RLDT-----PL-----DAWAYTDHAYPTTVARFSPN--GEWVASADASGCVRVWG-----
C.elegans        PIDS-----LN-----SADIYTEHAHQTTVAKISPT--GFYCASGDTQGNIRIWD-----
C.jacchus        NIDN-----PA-----LADIYTEHAHQVVVARYSPS--GFYIASGDVSGKLRIWD-----
C.neoformans     DLNH-----VG-----LSHIYTEHTQNTTVARISPS--GYYCASADVAGNVRVWD-----
P.tetraurelia    -------------LPNKY----L--CT--KCVCFIGNIPFDLYKKYFF-YSCVKSHLIFE
D.rerio          NIEN-----PA-----IADVYTEHPHQVIVAKYCPS--GFYIASGDVSGKVRIWD-----
D.discoideum     NVKN-----PM-----VADIYYEHPCQTTVAKYAPS--GNYIASGDVQGNLRIWD-----
D.melanogaster   NIEN-----PA-----IADVYTEHSCAVNVAKYSPS--GFYIASGDASGKIRIWD-----
E.siliculosus    NFTN-----PE-----V----QTHNAPVSCIHFSA-------------------------
G.gallus         NIDN-----PA-----IADIYTEHAHQVVVAKYAPS--GFYIASGDVSGKLRIWD-----
G.gorilla        NIDN-----PA-----LADIYTEHAHQVVVAKYAPS--GFYIASGDVSGKLRIWD-----
H.sapiens        NIDN-----PA-----LADIYTEHAHQVVVAKYAPS--GFYIASGDVSGKLRIWD-----
I.tridecemlinea  NIDN-----PA-----VADIYTEHAHQVVVAKYAPS--GFYIASGDVSGKLRIWD-----
M.mulatta        ------------------------------------------------------------
M.gallopavo      NIDN-----PA-----IADIYTEHAHQVVVAKYAPS--GFYIASGDVSGKLRIWD-----
M.musculus       NIDN-----PA-----IADIYTEHAHQVVVAKYAPS--GFYIASGDISGKLRIWD-----
O.sativa         RLDA-----PL-----DCWAYPDHAYPTTVARFSPN--GEWVASADASGCVRVWG-----
O.latipes        NIDN-----PA-----IADIYTEHAHQVGVAKYAPS--GFYIASGDASGKIRIWD-----
P.troglodytes    NIDN-----PA-----LADIYTEHAHQVVVAKYAPS--GFYIASGDVSGKLRIWD-----
P.falciparum     NIEK-----------ENEDKITNYIYQTRYSRYSK----YNIKMDEYT--LRRND-----
P.abelii         NIDN-----PA-----LADIYTEHAHQVVVAKYAPS--GFYIASGDVSGKLRIWD-----
S.cerevisiae     CLDD-----GDSKVPPVVQFTGHGSSVVTTVKFSPIKGSQYLCSGDESGKVIVWG-----
S.pombe          EIEK-----QE-----NCFQYNEHTAPTTVARFSPS--GYYVASGDNQGNVRIWD-----
S.purpuratus     DIED-----PT-----QVTIFNDHARATTSARWAPS--GYYVASAAECGNVRVWV-----
S.scrofa         ------------------------------------------------------------
T.rubripes       AIESLKLPDST-----LIDLYEQVVMELIEMREVG----------AARSLLRQTDPMIML
X.tropicalis     AIQSLKLPDKT-----LIDLYEQVVLELIELRELG----------AARSLLRQTDPMIML
                                                                             


                       2530      2540      2550      2560      2570      2580
                 =========+=========+=========+=========+=========+=========+
A.carolinensis   KQTQP------ERYIHLENLLARSYFDPREAYPDGSSKEKRRAAIAQA-LAGEVSVVPPS
A.gambiae        -TVNK------EHILKNEFQPIGGPIKDISWSPDSQRIVIVGEGR-ER-FGHVFMAETGT
A.thaliana       -AYN-------DHVLKNEFKVLAGRIDDLQWSADGMRIVASGDGK-GKSLVRAFMWDSGS
B.distachyon     -RYG-------DRALKAEFRPISGRVDDLRWSPDGLRIVVSGDGK-GKSLVRAFMWDSGS
C.elegans        -TTQS------THILKHTIPVFSGPVKDIAWDSESKRIAAVGEGK-ER-FGHVFLLDTTT
C.jacchus        -TTQK------EHLLKYEYQPFAGKIKDIAWTEDSKRIAVVGEGR-EK-FGAVFLWDSGS
C.neoformans     -VTQP------ENILKLATRPLGGKINDLAWDGESKRIIVGGEGK-DR-FGAAFFMDSGS
P.tetraurelia    -------KSDGFRQIFIYF------KQK----IYLFSRKCSLV--KIIKKNLSR------
D.rerio          -TTQR------EHLLKYEYQPFGGKIKDIAWTEDSKRLAVVGEGR-EK-FGAVFLWDTGS
D.discoideum     -TLQK------EHILKATYKVLNGAILDIAWTSDNQRLVVVGDGK-ER-FGAAILWDSGS
D.melanogaster   -TVNK------EHLLKNEFQPIAGPIKDISWSPDNQRIVAVGEGR-ER-FGHVFMSETGT
E.siliculosus    -------------TAPHDFAVTCSTHVSLHAAATGRQIRSVGR------FDHVAHSAQLR
G.gallus         -TTQK------EHLLKYEYQPFAGKIKDLAWTEDSKRIAVVGEGR-EK-FGAVFLWDSGS
G.gorilla        -TTQK------EHLLKYEYQPFAGKIKDIAWTEDSKRIAVVGEGR-EK-FGAVFLWDSGS
H.sapiens        -TTQK------EHLLKYEYQPFAGKIKDIAWTEDSKRIAVVGEGR-EK-FGAVFLWDSGS
I.tridecemlinea  -TTQK------EHLLKYEYQPFAGKIKDIAWTEDSKRIAVVGEGR-EK-FGAVFLWDSGS
M.mulatta        ------------------------------------------------------------
M.gallopavo      -TTQK------EHLLKYEYQPFAGKIKDLAWTEDSKRIAVVGEGR-EK-FGAVFLWDSGS
M.musculus       -TTQK------EHLLKYEYQPFAGKIKDIAWTEDSKRIAVVGEGR-EK-FGAVFLWDTGS
O.sativa         -RYG-------DRALKAEFRPLSGRVDDLRWSPDGLRIVVSGDGK-GKSFVRAFVWDSGS
O.latipes        -TTQK------EHMLKYEYTPISGKIKDIAWTEDSKRIAVVGDGR-EK-FGAVFLWDSGS
P.troglodytes    -TTQK------EHLLKYEYQPFAGKIKDIAWTEDSKRIAVVGEGR-EK-FGAVFLWDSGS
P.falciparum     -YVKD------NNVYYNPNHHKTNSITNYKNDDINKK-----SDN-NKY-----------
P.abelii         -TTQK------EHLLKYEYQPFAGKIKDIAWTEDSKRIAVVGEGR-EK-FGAVFLWDSGS
S.cerevisiae     -WTFDKESNSVEVNVKSEFQVLAGPISDISWDFEGRRLCVVGEGR-DN-FGVFISWDSGN
S.pombe          -CAGE------DKILKNQVTAISGRITDLDWDGDSQRIIAVGEGK-ER-YGHAFTADSGN
S.purpuratus     -IVNP------CTLLVFLDQ----------------RNLING--------GAALIVDTGT
S.scrofa         ----------------MATEEK----KPETEAARA-------------------------
T.rubripes       KQTQP------QRYLHLENLLTCSYFDPLEAYPKGSSKEKRRLAIAET-LVREVSVVPPS
X.tropicalis     KQNQP------ERYIHLENLLARSYFDPREAYPDGSSKEKRRAAIAQA-LAGEVSVVPPS
                                                                             


                       2590      2600      2610      2620      2630      2640
                 =========+=========+=========+=========+=========+=========+
A.carolinensis   RLMALLGQALKWQQHQGLLPP--------G--MTIDLFRGKAAVKDVEEEKFPTQLSRHI
A.gambiae        SVGEISGQSKPINSCDFRPAR--------P--FRIITGSEDNTIGVFEGPPFKF--KMTK
A.thaliana       NVGEFDGHSRRVLSCAIKPTR--------P--FRIVTCGEDFLVNFYEGPPFKF--KLSS
B.distachyon     TVGDFDGHSKRVLSCDFKPTR--------P--FRIVTCGEDFLANFYEGPPFKF--KHSI
C.elegans        SNGSLTGQSRTMNSVDFKPTR--------P--FRIISGSDDNTVAIFEGPPFKF--KHTF
C.jacchus        SVGEITGHNKVINSVDIKQSR--------P--YRLATGSDDNCAAFFEGPPFKF--KFTI
C.neoformans     SCGEIAGHAKPITALSIRQQR--------P--FRAISGSDDNSLIFHTAVPFKY--DKMI
P.tetraurelia    --------K--------NNFNSKIILDGELCFNL---V--S------ETYEFLIYDLISF
D.rerio          SVGEIVGHSKIINSVDIKQTR--------P--YRLVTGSDDNCTTFLEGPPFKF--KCTM
D.discoideum     SCGEITGHSKMILSCDIKSTR--------P--FRAATGSEDFAVNWFEGPPFKF--QKNI
D.melanogaster   SVGEISGQSKSINSADFRPAR--------P--FRIVTGSEDNTIAVFEGPPFKF--KMTK
E.siliculosus    SDGKLMGTGDHTGTVKVIDVK--------T--KGIL---------------------RAF
G.gallus         SVGEITGHNKVINSVDIKQTR--------P--YRLATGSDDNCAAFFEGPPFKF--KFTL
G.gorilla        SVGEITGHNKVINSVDIKQSR--------P--YRLATGSDDNCAAFFEGPPFKF--KFTI
H.sapiens        SVGEITGHNKVINSVDIKQSR--------P--YRLATGSDDNCAAFFEGPPFKF--KFTI
I.tridecemlinea  SVGEITGHNKVINSVDIKQSR--------P--YRLVTGSDDNCAAFFEGPPFKF--KFTI
M.mulatta        ------------------------------------------------------------
M.gallopavo      SVGEITGHNKVINSVDIKQTR--------P--YRLATGSDDNCAAFFEGPPFKF--KFTL
M.musculus       SVGEITGHNKVINSVDIKQTR--------P--YRLATGSDDNCAAFFEGPPFKF--KFTI
O.sativa         TVGEFDGHSKRVLSCDFKPTR--------P--FRIVTCGEDFLANYYEGPPFKF--KHSI
O.latipes        SVGELSGHSKLINSVDIRQKR--------P--YRLAAASDDTCGSFFEGPPFKF--KFTL
P.troglodytes    SVGEITGHNKVINSVDIKQSR--------P--YRLATGSDDNCAAFFEGPPFKF--KFTI
P.falciparum     -------FDDDIFIFNMYKNMEERNIRKNINRYNLIKDDNNMYRNILFEHSHKN----IM
P.abelii         SVGEITGHNKVVNSVDIKQSR--------P--YRLATGSDDNCAAFFEGPPFKF--KFTI
S.cerevisiae     SLGEVSGHSQRINACHLKQSR--------P--MRSMTVGDDGSVVFYQGPPFKF--SASD
S.pombe          SVGEIFGHSSVVNAVSLRKSR--------P--FRAATASDDNSINFFHGTPYRF--NRSL
S.purpuratus     SCGTISTLGR-TSTCDVRQKR--------P--YRAVIGADSYVVKLFEGPPFKY--KSQY
S.scrofa         ------------------QPA--------P--SAAATQSKP----TPVKPNYAL--KFTL
T.rubripes       RLMGLLEQSMKIQPHSDQLSP------------EMTIDVSANKNRHMEKETFPTQLYRHI
X.tropicalis     RLMALLGQALKWQQHQGLLPP--------G--MIIDLFRGKAAVKDVEEEKFPTQLSRHI
                                                                             


                       2650      2660      2670      2680      2690      2700
                 =========+=========+=========+=========+=========+=========+
A.carolinensis   KF-GQ---KSHVECARFSPD-GQYLVTGSVDGFIEVWNFTTGKIRKDLKYQAQ-DNFM--
A.gambiae        --QDH---TRFVQAVRYSPS-GHLFASAGFDGKVFIYDGTTSELVGEVG--S-----P--
A.thaliana       --REH---SNFVNCVRFAPD-GSKFITVSSDKKGIIYDGKTCEILGELS-SD-----D--
B.distachyon     --RDH---TNFVNCIRYAPD-GSKFITVSSDKKGLIYDGKTGDKIGELS-SE-----G--
C.elegans        --DHH---KKFVQSTRYNND-GSLFASTGSDGTVVLYNGVDGEKVGVLE-DA-----KGV
C.jacchus        --GDH---SRFVNCVRFSPD-GNRFATASADGQIYIYDGKTGEKVCALG-GS-----K--
C.neoformans     --NTH---TRFVRDVAFSPN-GDLFASVASDGKMFFYEGKTGEVKGEVD-RD--------
P.tetraurelia    YED-WRVLSWNLK-SKIRFFEITLKDIICSLKFNV-------------KKK--NEFRTNR
D.rerio          --TDH---SRFVNCVRFSPD-GSRYASAGADGQIFLYDGKTGEKLSSLG-GE-----K--
D.discoideum     AAGDF---TRFVNCVRFSPD-GNKLVTVGADKKAFVYDGKTGEKLIELN-PA-----Q--
D.melanogaster   --QDH---SRFVQAVRYSPD-GKFFASAGFDGKVFLYDGTSSELVGEFG--S-----P--
E.siliculosus    --REHK---APTRVVRWSCD-GLQLASGSDDKTLRLWDLPTSTAVQVRQ-----------
G.gallus         --SDH---TRFVNCVRFSPD-GNRFATASADGQIFIYDGKTGEKVCALG-GG-----K--
G.gorilla        --GDH---SRFVNCVRFSPD-GNRFATASADGQIYIYDGKNGEKVCALG-GS-----K--
H.sapiens        --GDH---SRFVNCVRFSPD-GNRFATASADGQIYIYDGKTGEKVCALG-GS-----K--
I.tridecemlinea  --GDH---SRFVNCVRFSPD-GNRFATASADGQIYIYDGKTGEKVCALG-EG-----K--
M.mulatta        ------------------------------------------------------------
M.gallopavo      --SDH---TRFVNCVRFSPD-GNRFATASADGQIFIYDGKTGEKVCALG-GG-----K--
M.musculus       --GDH---SRFVNCVRFSPD-GNRFATASADGQIFIYDGKTGEKVCALG-ES-----K--
O.sativa         --RDH---SNFVNCIRYAPD-GSKFISVSSDKKGLIYDGKTGDKIGELS-SE-----G--
O.latipes        --RDH---SQFVNCVRFSPD-GNRFATAGADGQIFLYDGTTGESVGTLG-GE-----K--
P.troglodytes    --GDH---SRFVNCVRFSPD-GNRFATASADGQIYIYDGKTGEKVCALG-GS-----K--
P.falciparum     --LRE---YDKTKNITFHPH-GTYFLTYPFYILNNN----KKEKRKIPNMPYRILSAP--
P.abelii         --GDH---SRFVNCVRFSPD-GNRFATASADGQIYIYDGKTGEKVCALG-GS-----K--
S.cerevisiae     --RTHHKQGSFVRDVEFSPDSGEFVITVGSDRKISCFDGKSGEFLKYIE-DD-----QE-
S.pombe          --RVH---SKFVYDVRYSPN-DERFASAGADGKVYVFDGKTGDQVYEID-----------
S.purpuratus     --QEH---NNFVQKVRYAPN-GEMFVSGGSDGKLFLRGGDQGELLKDLG-ETR-EKGK--
S.scrofa         --AGHT---KAVSSVKFSPN-GEWLASSSADKLIKIWGAYDGKFEKTIS-----------
T.rubripes       KF-GR---RSHVECARFSPD-GRYLITGSVDGFIEVWNFVTGKISKDLKYQAQ-ESFM--
X.tropicalis     KF-GQ---KSHVECARFSPD-GQYLVTGSVDGFIEVWNFTTGKIRKDLKYQAQ-DNFM--
                                                                             


                       2710      2720      2730      2740      2750      2760
                 =========+=========+=========+=========+=========+=========+
A.carolinensis   MMDDAVLCMC---FSRDTEMLATGAQDGKIKVWKI-Q---SGQ-----------------
A.gambiae        AHSGGVYGVA---WKPDGTQLLTCSGDKSCRLWDV-A---TRTLISEFPM---GS-TVDD
A.thaliana       GHKGSIYAVS---WSPDGKQVLTVSADKSAKIWDI-SDNGSGSLNTTLNCPGSSG-GVDD
B.distachyon     IHSGSIYAVS---WSPDSKQVLTVSADKTAKVWDI-MEDASGKLNRTLVCNG-TG-GVDD
C.elegans        AHTGSIFALA---WSPDQSRIATASADKSVKIWDV-S---ARKLERTIVM---GS-KIED
C.jacchus        AHDGGIYAVSMNTWSPDSTHLLSASGDKTSKIWDV-N---VNSVVSVFPM---GT-TVLD
C.neoformans     GSTASLMACS---WSPDSSRVTTAGTDGIVAIWDS-S---TLKTIQSYNV---GS-DVAA
P.tetraurelia    IQSLFNNLIKNTIFQNQLYLNH-------Q----V-KSDLICNENDGIVFTPSRLFFFFK
D.rerio          AHDGGIYAVS---WSPDSTQLISASGDRTVKLWDV-G---SGTSVSTFSL---GS-DVLD
D.discoideum     QHTGGIYGCS---WSADNNRVLTASADKSCKIWDT-T---TGQCINSFTF---GS-DVND
D.melanogaster   AHKGGVYALA---WKPDSTQLLTCSGDKTCRLWTV-E---SRELVSEFVM---GT-TVDD
E.siliculosus    GHADYVRALAASPSSPDT--WISGSYDHTVKMWDTRQ---PKGNVLELSH---GA-PVEA
G.gallus         AHDGGIYAIS---WSPDSSQLLSASGDKTAKIWDV-G---ANSVVSTFNM---GS-NVLD
G.gorilla        AHDGGIYAIS---WSPDSTHLLSASGDKTSKIWDV-S---VNSVVSTFPM---GS-TVLD
H.sapiens        AHDGGIYAIS---WSPDSTHLLSASGDKTSKIWDV-S---VNSVVSTFPM---GS-TVLD
I.tridecemlinea  AHEGGIYAIS---WSPDSTHLLSASGDKTSKIWDV-G---VNSVVSTFPM---GS-SVLD
M.mulatta        -------------------------------------------------M---GS-TVLD
M.gallopavo      AHDGGIYAIS---WSPDSCQLLSASGDKTAKIWDV-G---ANSVVSTFNM---GS-NVLD
M.musculus       AHDGGIYAIS---WSPDSTHLLSASGDKTSKIWDV-N---VNSVVSTFPM---GS-NVLD
O.sativa         SHTGSIYAVS---WSPDSKQVLTVSADKTAKVWDI-LEDASGKLNRTLACPG-TG-GVDD
O.latipes        AHGGGIYALS---WSPDSSQLISASGDKTVKLWDV-G---AGTAVTTFNL---GS-DVTD
P.troglodytes    AHDGGIYAIS---WSPDSTHLLSASGDKTSKIWDV-S---VNSVVSTSPM---GS-TVLD
P.falciparum     ELMDDFY-LNLLDWSK-KNIIATALCDK-LYLWNN----NTCTNQKLFVANK-IEKEIEQ
P.abelii         AHDGGIYAIS---WSPDSTHLLSASGDKTSKIWDV-N---VNSMVSTFPM---GS-TVLD
S.cerevisiae     PVQGGIFALS---W-LDSQKFATVGADATIRVWDV-T---TSKCVQKWTL---DKQQLGN
S.pombe          AHKGSIFSIS---WSPDSSQFVTSSAGYSCKIWDA-N---TGSLIREWL-----S-SDKK
S.purpuratus     AHIGSIYGIS---WSDDSKKILTSSADKTAKIWDV-E---TGQAVTEFTF---GT-DLEH
S.scrofa         GHKLGISDVA---WSSDSNLLVSASDDKTLKIWDV-S---SGK-----------------
T.rubripes       MMDDAVLCMC---FSQDAELLATGAQNGKIKVWTI-Q---SGL-----------------
X.tropicalis     MMDDAVLCMC---FSRDTEMLATGAQDGKIKVWKI-Q---SGQ-----------------
                                                                             


                       2770      2780      2790      2800      2810      2820
                 =========+=========+=========+=========+=========+=========+
A.carolinensis   ---------------------------------------------CLRRFERAHSKGVTC
A.gambiae        QQVS---CLW-Q-GD-----HILSVS---LSGFINYLDV--NNPTKPLRIVKGHNKPITV
A.thaliana       MLVG---CLW-Q-ND-----HIVTVS---LGGTISIFSA--SDLDKSPFQFSGHMKNVSS
B.distachyon     MLVG---CLW-Q-ND-----HLVTVS---LGGTFNIFSA--SNPDKEPVTFAGHLKTVSA
C.elegans        QQLG---IVW-T-KS-----ALITVS---VSGFLNFLNP--DDGSVE-KIRQGHNKAITA
C.jacchus        QQLG---CLW-Q-KD-----HLLSVS---LSGYINYLDR--NNPSKPLRVIKGHSKSIQC
C.neoformans     QQNG---VVY-ANTN-----TVVSAS---LSGTLNIFDI--REPTSKWRTLHGPTKAITA
P.tetraurelia    FFF-FTLKWKYENKNSSDFCVKNFS------------PEK--IDYCMLFQNL-VFR----
D.rerio          QQLG---CLW-V-NK-----HLLSVS---LSGYINYLDR--NNPNRPLRTIKGHTKSIQC
D.discoideum     QQLG---CLW-F-GD-----SLLSVN---LAGEISTLNL--DDVAKPSRVIKGHNKLVGT
D.melanogaster   QQVS---CLW-Q-GD-----NLITVS---LSGVITYLNV--ADPSKPLRVVKGHNKPITV
E.siliculosus    -------CLFLG-GG-----GLCVSA---GGNEVKVWDLLGGGGGRLLHTLANHQKTVTC
G.gallus         QQLG---CLW-Q-KD-----HLLSLS---LSGYINYLDK--NNPDKPLRVIKGHSKSIQC
G.gorilla        QQLG---CLW-Q-KD-----HLLSVS---LSGYINYLDR--NNPSKPLRVIKGHSKSIQC
H.sapiens        QQLG---CLW-Q-KD-----HLLSVS---LSGYINYLDR--NNPSKPLHVIKGHSKSIQC
I.tridecemlinea  QQLG---CLW-Q-KD-----HLLSIS---LSGYINYLDK--NNPSKPLRVIKGHSKSIQC
M.mulatta        QQLG---CLW-Q-KD-----HLLSVS---LSGYINYLDR--NNPSKPLRVIKGHSKSIQC
M.gallopavo      QQLG---CLW-Q-KD-----HLLSLS---LSGYINYLDK--NNPDKPLRVIKGHSKSIQC
M.musculus       QQLG---CLW-Q-KD-----HLLSIS---LSGYINYLDK--NNPSKPLRVIKGHSKSIQC
O.sativa         MLVG---CLW-Q-ND-----YLVTVS---LGGTFNVFSA--SNPDKEPVTFAGHLKTVSS
O.latipes        QQLG---CLW-Q-KD-----HLLSIS---LSGYINYLDK--NNPNRPIRTVKGHSKSIQC
P.troglodytes    QQLG---CLW-Q-KD-----HLLSVS---LSGYINYLDR--NNPSKPLRVIKGHSKSIQC
P.falciparum     KDKGDDKKTE-K-KDKDKKEKRKSNNDKYYNHKCESMEI--LKPSEKKK--NKPQKTISS
P.abelii         QQLG---CLW-Q-KD-----HLLSVS---LSGYINYLDR--NNPSKPLRVVKGHSKSIQC
S.cerevisiae     QQVG---VVA-TGNG-----RIISLS---LDGTLNFYEL--GHD-EVLKTISGHNKGITA
S.pombe          QLVG---TVW-PTKD-----LIIVVN---SKGNLTYLNP--SDC-KVIDTIYGHQRSITA
S.purpuratus     QQLG---CLW-Q-GD-----FLLSLS---LSGNINYLDP--NDPSKPCRIVQGHQKRIVS
S.scrofa         -------CL--------------------------------------KTL-KGHSNYVFC
T.rubripes       ---------------------------------------------CLRRFEHAHNKGVTC
X.tropicalis     ---------------------------------------------CLRRFERAHSKGVTC
                                                                             


                       2830      2840      2850      2860      2870      2880
                 =========+=========+=========+=========+=========+=========+
A.carolinensis   LSFSKDSS----QILSASFDQTIRIHGLK--SGKT----LK-EFRGH-SSFVNEATFTQD
A.gambiae        LTLS--DD--RSTIYTGSHDGAVTNWNSG--SGTN----DRVAGIGH-GNQINDIRAAG-
A.thaliana       LAVLK-GN--ADYILSGSYDGLICKWMLG--RGFC----GKLQRT-Q-NSQIKCFAAHE-
B.distachyon     LTFLPQSN--PRTMLTTSYDGVIIRWIQG--FGYG----GRLIRK-N-NTQIKCFIAAE-
C.elegans        LSKSSD----GKFLFSADAEGHITTWEIS--SGTS----SRKS--PH-TTMITGAQTSSN
C.jacchus        LTVHKNGG--KSYIYSGSHDGHIK-------TGEN----DSFAGKGH-TNQVSRMTVDES
C.neoformans     SAIFSNGNDKDTTFYAGSFDGTVKKFEIGEAYGEKEGTCDEIEGTGH-SARIAAISANGK
P.tetraurelia    -F---------KNRK-----TRK----------MNFKCTRRIGY---KSLVKEFES---K
D.rerio          VTVHKADG--RTSIYSGSHDGHINYWDAE--SGEN----EAFVGKGH-SNLVSRMMVDDA
D.discoideum     IAFDK--N--AGSLYSASYDASLLQWDLS--TGLA----TNFTGPAH-KNQITSIKING-
D.melanogaster   LGLS--DD--RSTIYTGSHDGVVTNWNSG--SGTN----DRITGTGH-GNQINGIAAWG-
E.siliculosus    LALDGTGS----RLLTGGLDRHVKIYNLE--NFKV----VHML-KYQ-APVLALALAPDN
G.gallus         LTVHKNGG--KSYIYSGSNDGHINYWDSD--TGEN----DGFSGKGH-TNQVSRMAVDEM
G.gorilla        LTVHKNGG--KSYIYSGSHDGHINYWDSE--TGEN----DSFAGKGH-TNQVSRMTVDES
H.sapiens        LTVHKNGG--KSYIYSGSHDGHINYWDSE--TGEN----DSFAGKGH-TNQVSRMTVDES
I.tridecemlinea  LTVHKNGG--KSYIYSGSHDGHINYWDSE--TGEN----DSFSGKGH-TNQVSRMTVDES
M.mulatta        LTVHKNGG--KSYIYSGSHDGHINYWDSE--TGEN----DSFAGKGH-TNQVSRMTVDES
M.gallopavo      LTVHKNGG--KSYIYSGSNDGHINYWDSD--TGEN----DGFSGKGH-TNQVSRMAVDEM
M.musculus       LTVHRNGG--KSYIYSGSHDGHINYWDSE--TGEN----DSFSGKGH-TNQVSRMTVNES
O.sativa         LAFFPQSN--PKTILSTSYDGVIMRWIKG--VGYG----GRLMRK-N-NTQIKCFTVAE-
O.latipes        LTVHKKDG--RSFVYSGSHDGHINYWDAE--TGQN----DCFSGKGH-SNQVSKMVTNAA
P.troglodytes    LTVHKNGG--KSYIYSGSHDGHINYWDSE--TGEN----DSFAGKGH-TNQVSRMTVDES
P.falciparum     LKWNI--N--GNFLATGLSNGVVEIWDIE--KCVR------IRKYKNHKSRVNTLCWNH-
P.abelii         LAVHKNGG--KSYIYSGSHDGHINYWDSE--TGEN----DSFAGKGH-TNQVSRMTVDES
S.cerevisiae     LTVN--------PLISGSYDGRIMEWSSSS--M----------HQDH-SNLIVSLDNSKA
S.pombe          ATLSPD----ATHFYTASYDGTVLSWDIGK------QKAFPLVGESH-TNQVMQMIMAD-
S.purpuratus     IAVNE--D--RSKVFAGSYDGVTTWWDSK--TGLA----KTVEGRKK-PTLITQIDYKE-
S.scrofa         CNFNPQSN----LIVSGSFDESVRIWDVK--TGKC----LKTL-PAH-SDPVSAVHFNRD
T.rubripes       LSFSKDNN----QILSASFNQSIRIHELR--SGKT----LK-ELTGH-LSFVNDAYFTQD
X.tropicalis     LSFSKDCS----QILSASFDQTIRVHGLK--SGKT----LK-EFRGH-SSFVNEATFTQD
                                                                             


                       2890      2900      2910      2920      2930      2940
                 =========+=========+=========+=========+=========+=========+
A.carolinensis   GHYII-SASSD---------------------------------G---TVKVWNMKT--S
A.gambiae        DFVYT-AGIDDSIKQISVEGNTYTAV-EAKLACQPRGMDILK--ESN-TVVVGCVKD--I
A.thaliana       EEIVT-SGYDNKISRISYKDDQCTNEESIDIGNQPKDLSLAPLSP-D-LLLVTFESG--V
B.distachyon     EELIT-SGYDNKVFRIPLNGDQCGDAESVDVGGQPNALCVAIQQP-E-LALITTDSA--I
C.elegans        GDLFT-VGWDDQLKITD----GSGDTKSFKLPSQPCGLEVS---D-D-LAIVACYKH--V
C.jacchus        GQLIS-CSMDDTVRYTNLTLRDYSGQAVVKLDVQPKCVAVGP--G-G-YAVVVCIGQ--I
C.neoformans     DKVWS-AGWDDKVT--AIEGSQFT-SNPIPTKAQPTSIAAT---P-D-SVYIATASG--V
P.tetraurelia    DNRIAEYVLN---K-NT---------SQ-------WIYCKDRKDKKNPNSIKTLIGILEI
D.rerio          ERLVT-CSMDDTVRYTDLKKKEYSASDMVKMDVQPKCVSVGP--G-G-LAVTVCIGQ--L
D.discoideum     DQLIT-CAMDDSVKISSISKKTYG--ESIGVDSPAQAVAFS---G-D-VVVAVSMKT--I
D.melanogaster   DFVYT-CGIDDSLRQFSVEGNSYTDY-VVKLNCQPRGLAILR--NEN-IIALACIKE--L
E.siliculosus    SSLV-AATTDRALTIRRRDLRHAAAL--AEREMAKSGRIGGP--V-G----S-DGGP--R
G.gallus         DQLVT-CSMDDTVRYTNLSKRDYSGQDAVKMDVQPKCLAVGP--G-G-YTVVLCIGQ--I
G.gorilla        GQLIS-CSMDDTVRYTSLMLRDYSGQGVVKLDVQPKCVAVGP--G-G-YAVVVCIGQ--I
H.sapiens        GQLIS-CSMDDTVRYTSLMLRDYSGQGVVKLDVQPKCVAVGP--G-G-YAVVVCIGQ--I
I.tridecemlinea  GQLVS-CSMDDTVRYTNLMLRDYSGQGVVKLDVQPKCVAVGP--G-G-YAVVVCIGQ--I
M.mulatta        GQLIS-CSMDDTVRYTSLTLRDYSGQGVVKLDVQPKCVAVGP--G-G-YAVVVCIGQ--I
M.gallopavo      DQLVT-CSMDDTVRYTNLSKRDYSGQDAVKMDVQPKCLAVGP--G-G-YTVVLCIGQ--I
M.musculus       EQLVS-CSMDDTVRYTNLTLRDYSGQGVVKLDVQPKCVAVGP--G-G-YTVVVCIGQ--I
O.sativa         EELVT-SGYDNKVFRIPLNGDQCGDAESVDVGGQPNALNLAIQKP-E-FALVTTDSG--I
O.latipes        XELVS-CSMDDTLRYTKLDKKEYSASDVVKMDFQPKSVSVA---G-G-ISLAVCIGQ--V
P.troglodytes    GQLIS-CSMDDTVRYTSLMLRDYSGQGVVKLDVQPKCVAVGP--G-G-YAVVVCIGQ--I
P.falciparum     NTLTT-GGRDNKIINSDIRSKEIYYIELTKHKSEICGLEWNAD-G-T-Y--LASGSNDNS
P.abelii         GQLIS-CSMDDTVRYTSLMLRDYSGQGVVKLDVQPKCVAVGP--G-G-YAVVVCIGQ--I
S.cerevisiae     QEYSS-ISWDDTLKVNGITKHEFGSQ--------PKVASANN--D-GFTAVLTNDDD--L
S.pombe          DHVIT-IGMDDTLRVIDIKQGCFAKDNVFPTGYQPIGVCSV---E-D-CLILVTVSD--I
S.purpuratus     GKLST-VGMDDTMRLIDDQTLEYMD-DSCGLDSQPQSVCRG--SG-G-LTVVGGIQK--V
S.scrofa         GSLIVSSSYDGLCRIWDTASGQ--------------------------------------
T.rubripes       GRHII-SASAD---------------------------------G---TVKIWNTKS--W
X.tropicalis     GHYII-SASSD---------------------------------G---TVKIWNMKT--T
                                                                             


                       2950      2960      2970      2980      2990      3000
                 =========+=========+=========+=========+=========+=========+
A.carolinensis   ECSNTFKSLGSTAGTDITVNSVILLPKNPEHFVVCNRSNTV-VIMNMQGQIVRSFSSGKR
A.gambiae        TVLQDSRKVSA-LPISYESSSVSINP-ETMDVAVGGDDSKV-HIYMLSGGQLTHKL-DLE
A.thaliana       VFLRDGKV-VSTINLGFIVTALAVTP-DGTEAVIGGQDGKL-HLYSINGDSLTEEAVLER
B.distachyon     VLLHKSNI-TSTTKVNYTITSSTVSP-DGTEAIVGAQDGKL-RIYSISGDTLTEEAVLEK
C.elegans        VVLSRGTP--TENPIEFHSTCVAFCA-EKSLVAVGGKDAKV-HVYKLNGDGRLEELKKIE
C.jacchus        VLLKDQRKCFSIDSPGYEPEVVAVHP-GGDTVAVGGADGSV-RLYSILGTTLKDEGKLLE
C.neoformans     EVNG-PSP--T-TLVTTPTSAVAAYP-GPNSDLVATASGKIVTLFSTSPQTVL--ATFDD
P.tetraurelia    TNENTREYELVDNLLKL------------------------------------------N
D.rerio          VLLKDKKKLFTLDSLDYEPEAVAVHP-GGGTVAVGGADGKV-HLYSVQGNTLKDDGKALE
D.discoideum     YVIKGGKI-VSQTAATWEPTSVAIN---DTEVSVGGKDNKI-HVFTLSGNNLTASHTLDN
D.melanogaster   TLVQDQKKIFS-LPIKYEASSIAVNA-DTSDVAVGGDDQKL-HIYTLKGGVLEPKV-ELD
E.siliculosus    SVRTGTARYFNRGKTEGAGEGDVKVA-ARRSRAIASYDESL-RKFRFREALDRGLATGSP
G.gallus         VLMKDKKKCFAIDDLGYEPEAVAVHP-GGGSVAVGGTDGNV-RLYSIQGTSLKSDDKTLE
G.gorilla        VLLKDQRKCFSIDNPGYEPEVVAVHP-GGDTVAVGGADGNI-RLYSILGTTLKDEGKLLE
H.sapiens        VLLKDQRKCFSIDNPGYEPEVVAVHP-GGDTVAIGGVDGNV-RLYSILGTTLKDEGKLLE
I.tridecemlinea  VLLKDQKKCFSIDSPGYEPEVVAVHP-AGDTVAVGGTDGNV-RVYSILGSTLKDEGKLLE
M.mulatta        VLLKDQRKCFSIDNPGYEPEVVAVHP-GGDTVAVGGADGNV-RLYSILGTTLKDEGKLLE
M.gallopavo      VLMKDKKKCFAIDDLGYEPEAVAVHP-GGGSVAVGGTDGNV-RLYSIQGTSLKSDDKTLE
M.musculus       VLLKDQKKCFSIDNPGYEPEVVAVHP-GGDTVAVGGTDGNV-RVYSILASTLKDEGKLLE
O.sativa         VLLHKSTV-ISTTKVNYTITSSAVSP-DGTEAIVGAQDGKL-RIYSINGDTVTEEALIEK
O.latipes        VLLKDKKKLFTLDNLGYEAEVGALHP-SGTTAAVGGTDGKI-RLYSIQGNTLKDEGKTLE
P.troglodytes    VLLKDQRKCFSIDNPGYEPEVVAVHP-GGDTVAIGGADGNV-RLYSILGTTLKDEGKLLE
P.falciparum     IYIWDKYT-----------------------------------------NKYL--FHFKK
P.abelii         VLLKDQRKCFSIDNPGYEPEVVAVHP-GGDTVAVGGADGNV-RLYSILGTTLKDEGKLLE
S.cerevisiae     LILQSFTG-DIIKSVRLNSPGSAVSL-SQNYVAVGLEEGNTIQVFKLSDLEVSFDLK-TP
S.pombe          QVLRSLTGVST-AKTIYQPSAVASHP-LKSEFCVGGEDCCV-YIHTLEKGELCEVAQCKD
S.purpuratus     TIVRGSNK-ASVLEVTYNGTAVDIHP-NQSEVAIGDEMGNI-HVYTLAGDTLTEKTSFKA
S.scrofa         --------------------------------------------------CLK--TLIDD
T.rubripes       ECIHTHKSV--SRASEVPVNNVIPLPQHPEHFVVCNHSNTV-VVLNMRGQTVKTFSSGRQ
X.tropicalis     ECSNTFKSLGSTAGTDITVNSVILLPKNPEHFVVCNRSNTV-VIMNMQGQIVRSFSSGKR
                                                                             


                       3010      3020      3030      3040      3050      3060
                 =========+=========+=========+=========+=========+=========+
A.carolinensis   EGGDFVCCALSPRGEWIYC---VGEDFVLYCFS---------------------------
A.gambiae        HLGPVTDVRYSPDNKLLVA---SDANRKVILYSVADYKP--PHNKEWGFHNARVNCVA--
A.thaliana       HRGAISVIRYSPDLSMFAS---ADLNREAVVWDRVSREMK--LK-NMLYHSARINCLA--
B.distachyon     HRGPITTIHYSPDVSMFAS---ADANREAVAWDRATREIK--LK-NMLFHTARINCLA--
C.elegans        HSAEITAVSFSDDGEYLAV---TDLARKVIPYSVSTDFSVTSPN-SWTFHTSKVLTVA--
C.jacchus        AKGPVTDVAYSHDGAFLAV---CDASKVVTVFSVADGYS--ENN-VFYGHHAKIVCLA--
C.neoformans     NKGDVLSLAFSPDGKYLAS---GDATGRIILIDVEKKETV-VSS-KWTFHTGRVVGLA--
P.tetraurelia    IRI-NRSKF---------------------------------------------------
D.rerio          VQGPVTDMSYSKDGAFLAV---TDEKKVVTVFTVADAYK--EKS-EFYGHHAKVVCLS--
D.discoideum     HRGAITDLSYSPCGKYLAS---GCSNREVIVWSGKE--AK--SK-GWVNHTARINAVA--
D.melanogaster   HLGAVTDVSYSPDLKYLVA---CDAHRKVVLYSVEEYKP--AHNKEWGFHSARVNTVA--
E.siliculosus    LVVAALLEALRERG----------------------------------------------
G.gallus         AKGPVTDLAYSHDGAFLAV---CDANKVVTVFSVPDGYV--EHN-VFYGHHAKVVCIA--
G.gorilla        AKGPVTDVAYSHDGAFLAV---CDASKVVTVFSVADGYS--ENN-VFYGHHAKIVCLA--
H.sapiens        AKGPVTDVAYSHDGAFLAV---CDASKVVTVFSVADGYS--ENN-VFYGHHAKIVCLA--
I.tridecemlinea  AKGPVTDVAYSHDGAFLAV---CDASKVVTVFSVADGYS--ENN-VFYGHHAKIVCLA--
M.mulatta        AKGPVTDVAYSHDGAFLAV---CDASKVVTVFSVADGYS--ENN-VFYGHHAKIVCLA--
M.gallopavo      AKGPVTDLAYSHDGAFLAV---CDANKVVTVFSVPDGYA--EHN-VFYGHHAKIVCIA--
M.musculus       AKGPVTDVAYSHDGAFLAV---CDASKVVTVFSVADGYS--ENN-VFYGHHAKIVCLA--
O.sativa         HRGAITCIHYSPDVSMFAS---ADANREAVVWDRATREIK--LK-NMLFHSARINCLA--
O.latipes        AKGPITDMAYSNDGAYLAV---IDEKKAITAYSVPDGYS--VKN-EFYGHHAKPVSLA--
P.troglodytes    AKGPVTDVAYSHDGAFLAV---CDASKVVTVFSVADGYS--ENN-VFYGHHAKIVCLA--
P.falciparum     HKAAVKAIAWCPYKNHILSSGGGSVDKKIFLWNIKTGKSI--NEIY---TKSQVSNII--
P.abelii         AKGPVTDVAYSHDGAFLAV---CDASKVVTVFSVADGYS--ENN-VFYGHHAKIVCLA--
S.cerevisiae     LRAKPSYISISPSETYIAA---GDVMGKILLYDLQSREV--KTS-RWAFHTSKINAIS--
S.pombe          STAPITCLAYSPDGKYLAC---GDASGKVVLYDANSREV--ITS-RWAFHTGRILGMS--
S.purpuratus     HCEAITRCRYSPDGEYLAV---ASASKAVEVYHHHCDYAQDLDR-KNYKHNAKITDLA--
S.scrofa         DNPPVSFVKFSPNGKYILA---ATLDNTLKLWDYSKGKC--LK--TYTGHKNEKYCIFAN
T.rubripes       EGAEFVCSTVSPRGEWIYC---VGEDLVLYCFN---------------------------
X.tropicalis     EGGDFVCCTLSPRGEWIYC---VGEDFVLYCFS---------------------------
                                                                             


                       3070      3080      3090      3100      3110      3120
                 =========+=========+=========+=========+=========+=========+
A.carolinensis   -------------------------------------TVTGKL-E--RTLTVHEKDVIGI
A.gambiae        FS-P-----NSE-----LV----ASGSLDT-TIIIWFVNSPAK-H-TIIKNAHPQSQITG
A.thaliana       WS-P-----NST-----MV----ATGSLDT-CVIVYEVDKPASSR-MTIKGAH-LGGVYG
B.distachyon     WS-P-----DSR-----LV----ATGSIDT-CAIVSEIDKPASSR-ITIKGAH-LGGVHG
C.elegans        WS-P-----DNQ-----RL----ATGSIDT-SVIIWDMKKPGE-HPVIIKGAHPMSSVNV
C.jacchus        WS-P-----DNE-----HF----ASGGMDM-MVYVWTLSDPET-R-VKIQDAHRLHHVSS
C.neoformans     WA-I-----DSK-----RL----ASAGLDE-NIYVWDTNKQLK-N-ISIKNAHP-GGVNG
P.tetraurelia    ------------------------------------------------------------
D.rerio          WS-P-----DNE-----HF----ATSGMDM-MVYVWTASDTEK-R-IKLPDCHRLHHVSG
D.discoideum     WS-N-----DSK-----FV----ASASLDS-QIYIWNVENPTASP-VQVKNSH-LGGVND
D.melanogaster   WS-P-----NSL-----LV----ASGSLDT-TIIIWSVANPAK-H-TIIKNAHPQSQITR
E.siliculosus    -----------A------LEKALSGRDAAG-LEPVVAYLAKHL-T--NP--RHTEQLIAV
G.gallus         WS-P-----DNE-----HF----ASGGMDM-MVYVWTVSDPET-R-IKIPDAHRLHHVSG
G.gorilla        WS-P-----DNE-----HF----ASGGMDM-MVYVWTLSDPET-R-VKIQDAHRLHHVSS
H.sapiens        WS-P-----DNE-----HF----ASGGMDM-MVYVWTLSDPET-R-VKIQDAHRLHHVSS
I.tridecemlinea  WS-P-----DNE-----HF----ASGGMDM-MVYVWTLSDPET-R-VKIQDAHRLHHVSS
M.mulatta        WS-P-----DNE-----HF----ASGGMDM-MVYVWTLSDPET-R-VKIQDAHRLHHVSS
M.gallopavo      WS-P-----DNE-----HF----ASGGMDM-MVYVWTVSDPET-R-VKIPDAHRLHHVSG
M.musculus       WS-P-----DNE-----HF----ASGGMDM-MVYVWTLSDPET-K-VKIQDAHRLHHVSS
O.sativa         WS-P-----DNR-----LV----ATGSLDT-CAIVYEIDKPASSR-ITVKGAH-LGGVHG
O.latipes        WS-P-----DNE-----HF----ATGGMDM-MVYVWTLSDPDR-R-IKLADAHRLHHVSS
P.troglodytes    WS-P-----DNE-----HF----ASGGMDM-VVYVWTLSDPET-R-VKIQDAHRLHHVSS
P.falciparum     WS-----INTSE-----LIS---TH-SHSLNQIILWNLPQL-KK--VTTLRGHKSRVLYA
P.abelii         WS-P-----DNE-----HF----ASGGMDM-MVYVWTLSDPET-R-VKIQDAHRLHHVSS
S.cerevisiae     WK-PAEKGANEEEIEEDLV----ATGSLDT-NIFIYSVKRPMK-I-IKALNAHK-DGVNN
S.pombe          WN-A-----KST-----HL----ATASLDT-NIHIYSVERPMK-Y-IAMKNAHS-LGATQ
S.purpuratus     WS-P-----DGK-----HY----ATTALDG-SVYVWGLEKPNP-I--QAMKVHGRAATNC
S.scrofa         FSVT-----GGK------W---IVSGSEDN-LVYIWNLQTKEI-V--QKLQGHTDVVIST
T.rubripes       -------------------------------------YKTGKL-Q--KTLTVHEKEVIGI
X.tropicalis     -------------------------------------TVTGKL-E--RTLTVHEKDVIGI
                                                                             


                       3130      3140      3150      3160      3170      3180
                 =========+=========+=========+=========+=========+=========+
A.carolinensis   AHHPHQN-LIATYSE--DGLLKL-WKP---------------------------------
A.gambiae        LVWLDNE-TLISTGQ--DCNTKV-WN---I-------------ENV--------------
A.thaliana       LGFADDS-HVVSSGE--DACIRV-WS---F-------------TP---------------
B.distachyon     LTFADND-TLVTAGE--DACVRV-WK---L-------------VQ---------------
C.elegans        VRWLDET-TVVSAGQ--DSNIKF-WN---V-------------P----------------
C.jacchus        LAWLDEH-TLVTTSH--DASVKE-WT---I-------------TY---------------
C.neoformans     VAWVDGNTRLVSAGA--DGCVRT-WT---V-------------PE---------------
P.tetraurelia    ------------------------------------------------------------
D.rerio          LAWLNPN-TLVTTSH--DACIKQ-WT---L-------------KI---------------
D.discoideum     VIYGSNN-EIFSAGN--EGAIKI-WY---V-------------S----------------
D.melanogaster   LVWLDNN-TVISTGQ--DCNTKV-WH---V-------------EN---------------
E.siliculosus    ----------TSS--VLDMYGDVVASSPKALGLFAKLRAQQTQG----------------
G.gallus         LAWLDEH-TLVTTSH--DASVKE-WS---I-------------SYN--------------
G.gorilla        LAWLDEH-TLVTTSH--DASVKE-WT---I-------------TY---------------
H.sapiens        LAWLDEH-TLVTTSH--DASVKE-WT---I-------------TY---------------
I.tridecemlinea  LAWLDEH-TLVTTSH--DASVKE-WT---I-------------TY---------------
M.mulatta        LAWLDEH-TLVTTSH--DASVKE-WT---I-------------TY---------------
M.gallopavo      LAWLDEH-TLVTTSH--DASVKE-WS---I-------------SYN--------------
M.musculus       LAWLDEH-TLVTTSH--DASVKE-WT---I-------------TY---------------
O.sativa         LTFVDND-SLVTAGE--DACIRV-WK---L-------------VP---------------
O.latipes        LAWIDEH-TLATTSH--DASIKL-WT---I-------------TY---------------
P.troglodytes    LAWLDEH-TLVTTSH--DASVKE-WT---I-------------TY---------------
P.falciparum     ALSPDGT-SIATGSP--DQTIRL-WNI----------------FPKCGDKSISLFFPLSN
P.abelii         LAWLDEH-TLVTTSH--DASVKE-WT---I-------------TY---------------
S.cerevisiae     LLWETPS-TLVSSGA--DACIKR-WN---V-------------VL---------------
S.pombe          VEWVSEN-ELLSTGS--DAAIKV-WS---V-------------T----------------
S.purpuratus     VVWLDNA-SFVTGGD--DACLRT-AS---V-------------T----------------
S.scrofa         ACHPTEN-IIASAALENDKTIKL-WK---S-------------D----------------
T.rubripes       THHPHEN-LIATYSE--DGLLRL-WKP---------------------------------
X.tropicalis     AHHPHQN-LIGTYSE--DGLLKL-WKP---------------------------------
                                                                             


                       3190      3200      3210      3220      3230      3240
                 =========+=========+=========+=========+=========+=========+
A.carolinensis   ------------------------------------------------------------
A.gambiae        -----A----------------------------MSRGPGPI------------------
A.thaliana       -----QMDLNAS--PQPEEDDEPFKRR------HEDRMESAVEIARREREERKKRMRFDR
B.distachyon     -----QMDLNAS--PLPEDDDQTYEGAGDADFSQDEHIESAVEIMRREREERRRKLKRDQ
C.elegans        -----LMATRGP---------------------TPDKARMGL------------------
C.jacchus        ------------------------------------------------------------
C.neoformans     -----L------------------------------------------------------
P.tetraurelia    ------------------------------------------------------------
D.rerio          ------------------------------------MSQTGA------------------
D.discoideum     -----NMK----RSN------------------NDSNGPNKK------------------
D.melanogaster   -----IMALTNR---------------------DRERGSGPL------------------
E.siliculosus    -----NMDLLSDLID------------------NAEKTHSSL------------------
G.gallus         ------------------------------------MSYNKI------------------
G.gorilla        ------------------------------------MAHNKI------------------
H.sapiens        ------------------------------------MAHNKI------------------
I.tridecemlinea  ------------------------------------------------------------
M.mulatta        ------------------------------------MAHNKI------------------
M.gallopavo      ------------------------------------------------------------
M.musculus       ------------------------------------MAYNKI------------------
O.sativa         -----QMDLNAS--PSPEEDDQSYEEH--ADFSQSEHAESAVEIMRREREERRRKLKREQ
O.latipes        ------------------------------------MSHTGP------------------
P.troglodytes    ------------------------------------MAHNKI------------------
P.falciparum     CYNTVR------------------------------------------------------
P.abelii         ------------------------------------------------------------
S.cerevisiae     -----E------------------------------------------------------
S.pombe          -----F------------------------------------------------------
S.purpuratus     -----F--------------------------------MLAV------------------
S.scrofa         -----C------------------------------------------------------
T.rubripes       ------------------------------------MSHTGP------------------
X.tropicalis     ------------------------------------MAHNKI------------------
                                                                             


                       3250      3260      3270      3280      3290      3300
                 =========+=========+=========+=========+=========+=========+
A.carolinensis   -------------------------------------------------------MLGPK
A.gambiae        -------------------------------PHRWLHCPRKSDSIIADRFIAFKTPLKRD
A.thaliana       PTRVS--Q--PAFRDQYRDTRVYD---QSKLPQGWLDCPGFG-LEI-GCIIPSKVPLSES
B.distachyon     PDDGPRPRPQQIRNEQINQNKIGGYRRIKETPQGWLDCPASG-QPI-DKIIPSKVPLDET
C.elegans        -------------------------------PDRWLHCPKTG-TLINNLFFPFKTPLCKM
C.jacchus        -------------------------------------------------------MLGPR
C.neoformans     ------------------------------------------------------------
P.tetraurelia    ------------------------------------------------------------
D.rerio          -------------------------------PPRWRNCPRRG-QPVAGKFLPMKTMLGPR
D.discoideum     ----------------------------SKQDDDPLSSILGDIDQSQHGKLQSTSIKSTF
D.melanogaster   -------------------------------PNRWLYCPRKSDTIIAERFLAFKTPLSNN
E.siliculosus    -------------------------------GKR--PAPSKPQPQGDDLFQALAHALSAL
G.gallus         -------------------------------PPRWLHCPRRG-QPVAGKFLPLKTMLGPR
G.gorilla        -------------------------------PPRWLNCPRRG-QPVAGRFLPLKTMLGPR
H.sapiens        -------------------------------PPRWLNCPRRG-QPVAGRFLPLKTMLGPR
I.tridecemlinea  ------------------------------------------------------------
M.mulatta        -------------------------------PPRWLNCPRRG-QPVAGRFLPLKTMLGPR
M.gallopavo      -----------------------------------------------GKFLPLKTMLGPR
M.musculus       -------------------------------PPRWLNCPRRG-QPVAGRFLPLKTMLGPR
O.sativa         HDDGPRLNRQPIRNDHMNQNKIIRHGRIKEPPQGWLDCPGSG-EPI-DRIVPSKVPLDET
O.latipes        -------------------------------PPRWLNCPRKG-HIIADKFLPMKTMLGPR
P.troglodytes    -------------------------------PPRWLNCPRRG-QPVAGRFLPLKTMLGPR
P.falciparum     -------------------------------------------------------MITST
P.abelii         ------------------------------------------------------------
S.cerevisiae     ------------------------------------------------------------
S.pombe          ------------------------------------------------------------
S.purpuratus     -------------------------------PERWLNCPRKG-TLIAGKFLPFKTPLGPK
S.scrofa         ------------------------------------------------------------
T.rubripes       -------------------------------PPRWRNCPRRG-QPVEGKFLPMKTMLGPR
X.tropicalis     -------------------------------PPRWLNCPRRG-QPVAAKFLPLKTMLGPK
                                                                             


                       3310      3320      3330      3340      3350      3360
                 =========+=========+=========+=========+=========+=========+
A.carolinensis   YDDQVAEAS-RFHPSMLSNFLKSLKVKMCLLIDLTNTTRFYDKS----------------
A.gambiae        FQSQMPVQC-SFAPSMLFDLMKRQKRRIGLWIDLTNTNRFYDKN----------------
A.thaliana       YNEHVPPGK-RYSFKQVVRNQRINGRKLGLVIDLTNTTRYYPTL----------------
B.distachyon     FNESVLPGK-RYSSKQVVNKQRKANREIGLVIDLTNTSRYYSPS----------------
C.elegans        YDNQIAERRYQFHPAEVFSHPHLHGKKIGLWIDLTNTDRYYFRE----------------
C.jacchus        YDSQVAEEN-RFHPSMLSNYLKSLKVKMGLLVDLTNTSRFYDRN----------------
C.neoformans     ------------------------------------------------------------
P.tetraurelia    ------------------------------------------------------------
D.rerio          YDDKVPEEN-RFHPSMLSNYLKSLKVKMGLLVDLTNTTRFYDRA----------------
D.discoideum     SLDSVMNRIIELSKQHGGIDSIEVEGRIGLFSNTSNGNTFKPGM----------------
D.melanogaster   FHDKMPIEC-TFQPEMLFEYCKTLKVKLGLWVDLTNTKRFYDRS----------------
E.siliculosus    HQRL------CHEPAEVRSN-LEIEVRVGLISQHDRLERATPGIPGSGAVQMDSDMMRQR
G.gallus         YDDQVAEEN-RFHPSMLSNYLKSLKVKMGLLVDLTNTNRFYDRN----------------
G.gorilla        YDSQVAEEN-RFHPSMLSNYLKSLKV----------------------------------
H.sapiens        YDSQVAEEN-RFHPSMLSNYLKSLKVKMGLLVDLTNTSRFYDRN----------------
I.tridecemlinea  ---------------MLSNYLKSLKVKMGLLVDLTNTSRFYDRN----------------
M.mulatta        YDSQVAEEN-RFHPSMLSNYLKSLKVKMGLLVDLTNTSRFYDRN----------------
M.gallopavo      YDDQVAEEN-RFHPSMLSNYLKSLKVKMGLLVDLTNTNRFYDRN----------------
M.musculus       YDSQVAEEN-RFHPSMLSNYLKSLKVKMSLLVDLTNTSRFYDRN----------------
O.sativa         FNESVPAGK-RYSSKQVVNKQRKAGRDIGLVIDLTNTTRYYSPT----------------
O.latipes        YDDQVEEAN-RFHPSMLFNHLKSLKVKMGLLVDLTNTNRFYDRN----------------
P.troglodytes    YDSQVAEEN-RFHPSMLSNYLKSLKVKMGLLVDLTNTSRFYDRN----------------
P.falciparum     YHPGEKIEN-EFLKEKI-------------------------RS----------------
P.abelii         ---------------------------MGLLVDLTNTSRFYDRN----------------
S.cerevisiae     ------------------------------------------------------------
S.pombe          ------------------------------------------------------------
S.purpuratus     YNDSIPEEN-RFDTSMLFAYMNSRQVKLGWVFDLTNTNRFYDKD----------------
S.scrofa         ------------------------------------------------------------
T.rubripes       YDDQVAAEN-RFHPSMLSNLLKSLKVKMGLLVDLTNTTRFYDRN----------------
X.tropicalis     YDDQVPEEN-RFHPSMLSNYLKSLKVKMGLLVDLTNTTRFYDRN----------------
                                                                             


                       3370      3380      3390      3400      3410      3420
                 =========+=========+=========+=========+=========+=========+
A.carolinensis   ---------------DIEKDGIV--YIKLQCKGHGECPTTENTEAFLRVCEYFSNKNPT-
A.gambiae        ---------------EIEDAGAT--YIKLKCRGHGETPSVEHVRSFIEIVEEFIQEHPL-
A.thaliana       ---------------DLKKDGIK--HVKIACRGRDAVPDNVSVNTFVNEVLQFVLNQKHA
B.distachyon     ---------------EWTKQGIK--HVKIACRGRDAVPENESVNTFVYEVLAFHERQKPS
C.elegans        ---------------EVTEHECI--YHKMKMAGRGVSPTQEDTDNFIKLVQEFHKKYPD-
C.jacchus        ---------------DIEKEGIK--YIKLQCKGHGECPTTENTETFIRLCERFNERNPP-
C.neoformans     ------------------------------------------------------------
P.tetraurelia    -------------------------------------------------MRRQYRFFNFI
D.rerio          ---------------DIEKEGIK--YVKLSCKGHGECPTAETTEMFIRLCEHFIEKTPT-
D.discoideum     ---------------VRDDWNDLYHHLKLKSEPVATTETDYIYSDSIRVAYDEHSKKCL-
D.melanogaster   ---------------AVEELGAK--YIKLQCRGHGETPSPEQTHSFIEIVDNFINERPF-
E.siliculosus    GLRFVSGVSAPAFDAVKEEVGRRYGVAELASKEVVYVYDSGQMRDQRVVADGVNPLRCE-
G.gallus         ---------------DIQKEGIK--YIKLQCKGHGECPTPENTETFIRVCEHFSEKNPT-
G.gorilla        ------------------------------------------------------------
H.sapiens        ---------------DIEKEGIK--YIKLQCKGHGECPTTENTETFIRLCERFNERNPP-
I.tridecemlinea  ---------------DIEKEGIK--YIKLQCKGHGECPTTENTETFIRLCERFNERNPP-
M.mulatta        ---------------DIEKEGIK--YIKLQCKGHGECPTTENTETFIRLCERFNERNPP-
M.gallopavo      ---------------DIQKEGIK--YIKLQCKGHGECPTPENTETFIRVCEHFSEKNPT-
M.musculus       ---------------DIEKEGIK--YIKLQCKGHGECPTTENTETFIRLCERFNERSPP-
O.sativa         ---------------EWTRQGTK--YVKIACKGRDAVPDNESVNTFVYEVMAFLDRQKQS
O.latipes        ---------------DIEKEGIK--YVKLACKGHGECPSKEITATFTRLCELFIQKNPS-
P.troglodytes    ---------------DIEKEGIK--YIKLQCKGHGECPTTENTETFIRLCERFNERNPP-
P.falciparum     ---------------KINEM------LKWKRRGFPGCNPVSLTN----------------
P.abelii         ---------------DIEKEGIK--YIKLQCKGHGECPTTENTETFIRLCERFNERNPP-
S.cerevisiae     ------------------------------------------------------------
S.pombe          ------------------------------------------------------------
S.purpuratus     ---------------EIEKNGAR--HIKLPCRGRGECPSKEQTSLFIQMCST-NCKNPD-
S.scrofa         ------------------------------------------------------------
T.rubripes       ---------------DIEKEGIK--YVKLHCKGHGECPSADTTAMFIRLCEHFIERNPT-
X.tropicalis     ---------------DIEKEGIK--YIKLQCKGHGECPSQENTDTFLRLCERFIDRNPT-
                                                                             


                       3430      3440      3450      3460      3470      3480
                 =========+=========+=========+=========+=========+=========+
A.carolinensis   ---DLIGVHCTHGFNRTGFLICAFLVEKLD-W-SIEAAVATFA-------QARPPGIYKG
A.gambiae        ---DVIGVHCTHGFNRTGFLIVSYMVERLD-C-AVDAAVMAFA-------QARPPGIYKG
A.thaliana       K--KYVLVHCTHGHNRTGFMIVHYLMRSMPTM-NVTQALKLFS-------DARPPGIYKP
B.distachyon     RNPKYVLVHCTHGHNRTGFMIVHYLMRTQLSS--VTEALNIFA-------QRRPPGIYKA
C.elegans        ---RVVGVHCTHGFNRTGFLIAAYLFQVEE-Y-GLDAAIGEFA-------ENRQKGIYKQ
C.jacchus        ---ELIGVHCTHGFNRTGFLICAFLVEKMD-W-SIEAAVATFA-------QARPPGIYKG
C.neoformans     ------------------------------------------------------------
P.tetraurelia    NYHFRSQSIIWQQIESFNFFLNVGLKKTLYK----FKCI---------NNLQENNLS-YN
D.rerio          ---ELIGVHCTHGFNRTGFLICAYLVEKMD-W-SIEAAVAAFA-------QARPPGIYKG
D.discoideum     ---RKDKKTDKTSFDQSTNLIYDFRIST-----SIEEKFPPPL---------SLPPGYII
D.melanogaster   ---DVIAVHCTHGFNRTGFLIVCYLVERLD-C-SVSAALAIFA-------SARPPGIYKQ
E.siliculosus    ---RK-EARQQVNFQLAAAQ-YDLRVQA-----SLEQPVPPEAVPGLQPGSSEPPQGWSG
G.gallus         ---ELIGVHCTHGFNRTGFLICAFLVEKLD-W-SIEAAVATFA-------QARPPGIYKG
G.gorilla        --------HCTHGFNRTGFLICAFLVEKMD-W-SIEAAVATFA-------QARPPGIYKG
H.sapiens        ---ELIGVHCTHGFNRTGFLICAFLVEKMD-W-SIEAAVATFA-------QARPPGIYKG
I.tridecemlinea  ---ELIGVHCTHGFNRTGFLICAFLVEKMD-W-SIEAAVATFA-------QARPPGIYKG
M.mulatta        ---ELIGVHCTHGFNRTGFLICAFLVEKMD-W-SIEAAVATFA-------QARPPGIYKG
M.gallopavo      ---ELIGVHCTHGFNRTGFLICAFLVEKLD-W-SIEAAVATFA-------QARPPGIYKG
M.musculus       ---ELIGVHCTHGFNRTGFLICAFLVEKMD-W-SIEAAVATFA-------QARPPGIYKG
O.sativa         RNPKYILVHCTHGHNRTGFMIVHYLMRTQLSS--VTEALNIFA-------QRRPPGIYKN
O.latipes        ---ELIGVHCTHGFNRTGFLICAYLVEKMD-W-STEAAVATFT-------LARAPGIYKG
P.troglodytes    ---ELIGVHCTHGFNRTGFLICAFLVEKMD-W-SIEAAVATFA-------QARPPGIYKG
P.falciparum     --------HNIKNLFTKEYLICE-KTDGVR-YFLFIASNTTFL-------IDRNYEIFKN
P.abelii         ---ELIGVHCTHGFNRTGFLICAFLVEKMD-W-SIEAAVATFA-------QARPPGIYKG
S.cerevisiae     ----------------------------------M-------------------------
S.pombe          ----------------------------------M-------------------------
S.purpuratus     ---QIIGVHCTHGYNRTGFLICAYLVETLD-W-SVDAAVSLFA-------QGRPPGIYKG
S.scrofa         ----------------------------MD-W-SIEAAVATFA-------QARPPGIYKG
T.rubripes       ---ELIGVHCTHGFNRTGFLICAYLVEKMD-W-SLEAAVAAFS-------QARTPGIYKG
X.tropicalis     ---ELIGVHCTHGFNRTGFLICAFLVEKMD-W-SIEAAVATFA-------QARPPGIYKA
                                                                             


                       3490      3500      3510      3520      3530      3540
                 =========+=========+=========+=========+=========+=========+
A.carolinensis   DYLKELFRRYGDVE--D-VP-APPPL-PEWCFDEDEEE-----------------DEEDN
A.gambiae        DYLKELFARYGDVE--D-AP-PPPEL-PAWCLEYDDGDQ----P-NNGA--HQLVEQDDD
A.thaliana       DYIDALYTFYHEIK-PE-SV-TCPPT-PEWKRSAELDLN----G-E---------AVQDD
B.distachyon     DYIQALYSFYHEI--PE-SI-ACPPT-PEWKRPSDLDLN----G-E---------AKQDD
C.elegans        DYIDDLFARYDPTE--DDKI-LAPEK-PDWEREMSIGMS----------------TQIDN
C.jacchus        DYLKELFRRYGDIE--E-AP-PPPLL-PDWCFEDDEDE-----------------DED--
C.neoformans     ------------------------------------------------------------
P.tetraurelia    FSNLKLGSTTSCNESTEYT--VTPNECRIRDISYMAPILVDVCYFFKKK-----------
D.rerio          DYLKELFRRYGDVE--D-AP-AAPPL-PEWCFDEDEEE-----------------DGEED
D.discoideum     RREKQRYTFTEDQWKID-LT-KVIVR-PDFNAT-----------------------VEQE
D.melanogaster   DYINELYKRYEDTN--A-AP-AAPEQ-PNWCLDYDDGNGDGFVQDNSSSTSQQAGEQDDD
E.siliculosus    RRTKRRFSFKSDANTPE-DE-RW-------------------------------------
G.gallus         DYLKELFRRYGDED--D-AP-SPPEL-PEWCFEDDEEE-----------------DDD-N
G.gorilla        DYLKELFRRYGDIE--E-AP-PPPLL-PDWCFEDDEDE-----------------DED--
H.sapiens        DYLKELFRRYGDIE--E-AP-PPPLL-PDWCFEDDEDE-----------------DED--
I.tridecemlinea  DYLKELFRRYGDIE--E-AP-PPPLL-PDWCFEDDEDE-----------------DED--
M.mulatta        DYLKELFRRYGDIE--E-AP-PPPLL-PDWCFEDDEDE-----------------DED--
M.gallopavo      DYLKELFRRYGDED--D-AP-SPPEL-PEWCFEDDEEE-----------------DDD-N
M.musculus       DYLKELFRRYGDIE--E-AP-PPPVL-PDWCFEDEDEE-----------------DED--
O.sativa         DYIQAIYSFYHEI--PE-NI-VCPPT-PEWKRPSDLDLN----G-E---------AKQDD
O.latipes        DYLKELFRRYGDEK--D-AP-PAPAL-PDWCFGEDDG------------------EVDDD
P.troglodytes    DYLKELFRRYGDIE--E-AP-PPPLL-PDWCFEDDEDE-----------------DED--
P.falciparum     DMHIPTIEDLSKKQ--Q-LTLLDGELVEDIIYNEK-------------------------
P.abelii         DYLKELFRRYGDIE--E-AP-PPPLL-PDWCFEDDEDE-----------------DED--
S.cerevisiae     ------------------------------------------------------------
S.pombe          ------------------------------------------------------------
S.purpuratus     DYIQELFDRYGDVS--E-AP-PPPDL-PDWCVGADDRD-----------------DDE--
S.scrofa         DYLKELFRRYGDIE--E-AP-PPPLL-PDWCFEDDEDE-----------------DEE--
T.rubripes       DYLRELFRRYGDEE--D-AP-SPPAL-PEWCFEDDDGG-----------------ELDDD
X.tropicalis     DYLKELFHRYGDIE--D-AP-KPPEL-PDWCFE---EE-----------------DVDDD
                                                                             


                       3550      3560      3570      3580      3590      3600
                 =========+=========+=========+=========+=========+=========+
A.carolinensis   C-KPGT---------PESEAGSST---------SFSGKRRKEHLKLGAVFL----EGIT-
A.gambiae        D-DGGGEESRPRGSKRSQDGEGAP---------QTPKRFKRASYNPNAVFM----ED---
A.thaliana       D-DDDS---------PP----------------DPVQEI----NQEN-VKM----SNDDT
B.distachyon     D-DD-N---------GD----------------LAPSPD----PADN-KAI----TNDDV
C.elegans        G-RPST--S-------QQIPATNGNN-------NQNGNQLSGGGDNSKLFM----DGL--
C.jacchus        --EDGK---------KESEPGSS----------ASFGKRRKERLKLGAIFL----EGVT-
C.neoformans     -----------------------------------------MPAHTPI---------PD-
P.tetraurelia    ----FFSR----MNLTLCILPIMIRSTGCIFFNKSENFFVKFKECPLDGGGYFIIKGIER
D.rerio          G-SASA---------PASEPSSSH---------TGQSKKKKERLKLGAVFL----EGVS-
D.discoideum     L-YEVE---------IELFPEAIQACQEKTSLTELLNDFLNAIKGLTNIVKNGGETSFP-
D.melanogaster   AEEVEGEDAG---G-DCDASTSDG---------QPRKKRRREMIIKNATFM----AG---
E.siliculosus    --------------------------------------------------------IWR-
G.gallus         G-KMGG---------QESEPGSSS---------SSFGKRRKEHLKLGAVFL----EGVT-
G.gorilla        --EDGK---------KESEPGSS----------ASFGKRRKERLKLGAIFL----EGVT-
H.sapiens        --EDGK---------KESEPGSS----------ASFGKRRKERLKLGAIFL----EGVT-
I.tridecemlinea  --EDGK---------KESEPGSS----------ASFGKRRKERLKLGAIFL----EGVT-
M.mulatta        --EDGK---------KESEPGSS----------ASFGKRRKERLKLGAIFL----EGVT-
M.gallopavo      G-KTGG---------QESEPGSSS---------SSFGKRRKEHLKLGAVFL----EGVT-
M.musculus       --EDGK---------KDSEPGSS----------ASFSKRRKERLKLGAIFL----EGIT-
O.sativa         D-DDGN---------VD----------------RAPSPN----HEDD-KVI----TNDDI
O.latipes        G-NAVG---------QELGPSSSG---------SGPGKRKKEKLKLGAVFL----EGIT-
P.troglodytes    --EDGK---------KESEPGSS----------ASFGKRRKERLKLGAIFL----EGVT-
P.falciparum     ---------------------------------TGVEEKKIVYLIYDGLYI----QRKD-
P.abelii         --EDGK---------KESEPGSS----------ASFGKRRKERLKLGAIFL----EGVT-
S.cerevisiae     ---------------------------------------VLAMESRVA---------PE-
S.pombe          ---------------------------------------APSEKDIEE---------VS-
S.purpuratus     --IAAS---------SKKQGGG-------------GGRNKNQQDKQ---FV----EG---
S.scrofa         --EDGK---------KESEPGSS----------ASFGKRRKERLKLGAIFL----EGVT-
T.rubripes       G-NAVG---------QESGPSSSG---------SAPGRRKKEKLKLGAVFL----EGIS-
X.tropicalis     G-NKVF---------QEAEAGSSG---------AAYNRRKKERLKLGAIFL----EGVT-
                                                                             


                       3610      3620      3630      3640      3650      3660
                 =========+=========+=========+=========+=========+=========+
A.carolinensis   VKGVTQITAQPKLGNIQQKCQQF----C--GW--KGTGFPGAQPVSMDEQNI-KFLEQKP
A.gambiae        VPGVTLVRDEALIAKLQERVREM----C--GS--KLQGFAGAQPVSMDMHNI-RYLKEMP
A.thaliana       LGDEIPHYQEEAYRQFCYKMLMM----N--VGGRGFMQFPGSHPVSLDRESL-QLLRQRY
B.distachyon     LGDAVPYDQQDILRGICFKLLDF----V--PNGRANAQFPGSHPVSLNSENL-QLLRQRY
C.elegans        IRGVKVCEDEGKKSMLQAKIKNL----C--KY--NKQGFPGLQPVSLSRGNI-NLLEQES
C.jacchus        VKGVTQVTTQPKLGEVQQKCHQF----C--GW--EGSGFPGAQPVSMDKQNI-KLLDLKP
C.neoformans     IPGELLT-DPTLQYFLAERVSNL----CGI----GGGKFPGSQPVSFSSSSL-DLLEKED
P.tetraurelia    VVSIRERLTFNKINIEKDQDG-N-L--CACVISCL--------------AERRVKNSIIL
D.rerio          VKGVSQVTTQPKLGEIQRKCQQF----S--EW--DRSGFPGAQPVSMDRKNI-RMLEQNG
D.discoideum     EISLDKVGNVSEFYRLRDLVFKYIPSAP--QR--KNDTFPGSMPVNFGKKYF-IHVQNNE
D.melanogaster   VPGVRQVSDQPRLGDLQRKVQDW----C--DW--KKNGFPGAQPVSMDRENI-KRLSEIP
E.siliculosus    ADLTLVEEDDGIRKAVAAACAQLKR--P--TG--KGSSFPGAQPVNMCKRNV-PDVQRGS
G.gallus         VKYVTQVTTQPKLGGIQQKCQQF----C--GW--EGSGFPGAQPVSMDKQNI-KFLEQKP
G.gorilla        VKGVTQVTTQPKLGEVQQKCHQF----C--GW--EGSGFPGAQPVSMDKQNI-KLLDLKP
H.sapiens        VKGVTQVTTQPKLGEVQQKCHQF----C--GW--EGSGFPGAQPVSMDKQNI-KLLDLKP
I.tridecemlinea  VRGVTQVTTQPKLGEVQQKCHQF----C--GW--EGSGFPGAQPVSMDKQNI-KLLEQKP
M.mulatta        VKGVTQVTTQPKLGEVQQKCHQF----C--GW--EGSGFPGAQPVSMDKQNI-KLLDLKP
M.gallopavo      VKYVNQVTTQPKLGGIQQKCQQF----C--GW--EGSGFPGAQPVSMDKQNI-KFLEQKP
M.musculus       VKGVTQVTTQPKLGEVQQKCHQF----C--GW--EGSGFPGAQPVSMDKQNI-RLLEQKP
O.sativa         LGDAVPYDQQDFLRSICFRLLEL----V--PSGRANAQFPGSHPVSLNSENL-QILRQRY
O.latipes        VKGVTQVTSQPKLGEIQRMCQKM----A--EW--DKSGFPGAQPVSMDRQNL-NFLSQNP
P.troglodytes    VKGVTQVTTQPKLGEVQQKCHQF----C--GW--EGSGFPGAQPVSMDKQNI-KLLDLKP
P.falciparum     IT-------N--LSYFERLTNVY----NYVIT--PLKKYKKSQKNKKNKNNLQTNHENES
P.abelii         VKGVTQVTTQPKLGEVQQKCHQF----C--GW--EGSGFPGAQPVSMDKQNI-KLLDLKP
S.cerevisiae     IPGLIQP-GNVTQ-DLKMMVCKL----LNSPK--PTKTFPGSQPVSFQHSDVEEKLLAHD
S.pombe          VPGVLAP-RDDVR-VLKTRIAKL----LGT----SPDTFPGSQPVSFSKKHL-QALKEKN
S.purpuratus     VKGVTVVTDFQKANHLRRKVEQM----V--GW--KRQEFPGSQPVSMDRNNI-NFLKKNY
S.scrofa         VKGVTQVTTQPKLGEVQQKCHQF----C--GW--EGSGFPGAQPVSMDKQNI-KLLEQKP
T.rubripes       VKGVTQLAVQPKLGEIQRKCQEM----S--EW--DRSGFPGAQPVSMDRQNI-TFLEHSP
X.tropicalis     VKHVNQITTEPKLGEIQRKCQQF----C--SW--RGSGFPGAQPVSMDRSNM-KFLEQKA
                                                                        #####


                       3670      3680      3690      3700      3710      3720
                 =========+=========+=========+=========+=========+=========+
A.carolinensis   FKVSW-------KADGTRYMMLID------GKN---EVYMIDRDNSVF--HVSNLEFPF-
A.gambiae        YRVSW-------KADGTRYMMLIH------REG---EIYFLDRDNSVF--KAEGIRFPT-
A.thaliana       YYATW-------KADGTRYMMLLT------I-D---GCYLIDRSFKFR--RVQ-MRFPC-
B.distachyon     YYATW-------KADGTRYMMLIM------R-D---GCFLIDRNFCFR--RVQ-MRFPI-
C.elegans        YMVSW-------KADGMRYIIYIN-------DG---DVYAFDRDNEVF--EIENLDFVT-
C.jacchus        YKVSW-------KADGTRYMMLID------GTN---EVFMIDRDNSVF--HVSNLEFPF-
C.neoformans     FWVCE-------KSDGVRVLVFIVVNQS-TEQQ---EVWLIDRKQRFF--KIQGLYFAH-
P.tetraurelia    KNKKFYFRSNIFLEDILVLILYKAINITDEKDFIGLVG-----FQFENILNFTVDEMKIM
D.rerio          YKVSW-------KADGTRYMMLID------GRN---EVYMIDRDNSVF--HIENLEFPF-
D.discoideum     YFVSD-------KTDGIRYMLLID------HTG----CYLVDRKFDFY--QIQGFDILV-
D.melanogaster   YRVSW-------KADGTRYMMLID------GRD---EVYFFDRNHSCF--QVENVTFVE-
E.siliculosus    YLVAE-------KTDGVRYLMMAV------GTERGATCVLVDRSMNVF--RVTGGGFLA-
G.gallus         YKVSW-------KADGTRYMMLID------GKN---EVYMIDRDNSIF--HVSNLEFPF-
G.gorilla        YKVSW-------KADGTRYMMLID------GTN---EVFMIDRDNSVF--HVSNLEFPF-
H.sapiens        YKVSW-------KADGTRYMMLID------GTN---EVFMIDRDNSVF--HVSNLEFPF-
I.tridecemlinea  YKVSW-------KADGT-------------------------------------------
M.mulatta        YKVSW-------KADGTRYMMLID------GTN---EVFMIDRDNSVF--HVSNLEFPF-
M.gallopavo      YKVSW-------KADGTRYMMLID------GKN---EVYMIDRDNSIF--HVANLEFPF-
M.musculus       YKVSW-------KADGTRYMMLID------GTN---EVFMIDRDNSVF--HVSNLEFPF-
O.sativa         YYATW-------KADGTRYMMLIT------R-D---GCFLIDRNFCFR--RVQ-MRFPL-
O.latipes        YKVSW-------KADGTRILSLVI------QTE---RLYELNRDNNNILEKAGNCSFFF-
P.troglodytes    YKVSW-------KADGTRYMMLID------GTN---EVFMIDRDNSVF--HVSNLEFPF-
P.falciparum     LYIELDEKDNIKKRKSNLNNMLTE------EEN----VLISHKKNDHP--HINNKNMNAV
P.abelii         YKVSW-------KADGTRYMMLID------GTN---EVFMIDRDNSVF--HVSNLEFPF-
S.cerevisiae     YYVCE-------KTDGLRVLMFIVINPV-TGEQ---GCFMIDRENNYY--LVNGFRFPR-
S.pombe          YFVCE-------KSDGIRCLLYMTEHPRYENRP---SVYLFDRKMNFY--HVEKIFYPV-
S.purpuratus     YWVSW-------KADGIRYMMLID------GPG---EVYLFDRDHVVF--SAPHLSFPN-
S.scrofa         YKVSW-------KADGTRYMMLID------GTN---EVFMIDRDNSVF--HVSNLEFPF-
T.rubripes       YKVSW-------KADGTRYMMLIN------GKN---EVFMIDRDNSVF--HIANLEFPF-
X.tropicalis     YKVSW-------KADGTRYMMIID------GKN---EVYMIDRDNSVF--HVTNLEFPF-
                 #####                                                       


                       3730      3740      3750      3760      3770      3780
                 =========+=========+=========+=========+=========+=========+
A.carolinensis   -R-----------KD------PRAHLENTLLDGEMIIDK---V--N--D--QVVPRYLIY
A.gambiae        -L-----------KDT------SVHITDTLVDGEMVLDN--YG--E--G--KKIPRYLVY
A.thaliana       -K-----------HSREG--ISDKVHHYTLLDGEMVIDTPTGE--Q--G--EARRRYLVY
B.distachyon     -R-----------NFNDG--F----HNFTLIDGEMVVDTIP----D--G--GLKRRYLAY
C.elegans        -K-----------NG--------APLMETLVDTEVIIDK---VEIN--GAMCDQPRMLIY
C.jacchus        -R-----------KD------LRMHLSNTLLDGEMIIDR---V--N--G--QAVPRYLIY
C.neoformans     -W-----------ENRS------AFLGETLLDGELVIDI---D--PISG--AQTLMYYAF
P.tetraurelia    GIFTQKQALLYIYQRLDLKV-W-K-Y------KQ--FFKKNAGNTYEFIKKIVFNVFSKY
D.rerio          -R-----------KD------LRIHLSNTLLDGEMIIDK---V--N--G--QPVPRYLIY
D.discoideum     -T-----------L----------FGEGTLLDGEMVRNL---Q--------TKRANFLIF
D.melanogaster   -S-----------RNL------NEHLDGTLVDGEMVLDK--IG--E-----TVTPRYLIY
E.siliculosus    -G-----------I----------VGVGTILDGELVHNR---T--------MKKAIFVAF
G.gallus         -R-----------KD------LRMHLTNTLLDGEMIVDK---V--N--G--QVVPRYLIY
G.gorilla        -R-----------KD------LRMHLSNTLLDGEMIIDR---V--N--G--QAVPRYLIY
H.sapiens        -R-----------KD------LRMHLSNTLLDGEMIIDR---V--N--G--QAVPRYLIY
I.tridecemlinea  ------------------------------------------------------------
M.mulatta        -R-----------KD------LRMHLSNTLLDGEMIIDR---V--N--G--QAVPRYLIY
M.gallopavo      -R-----------KD------LRMHLTNTLLDGEMIVDK---V--N--G--QVVPRYLIY
M.musculus       -R-----------KD------LRMHLSNTLLDGEMIIDK---V--N--G--QAVPRYLIY
O.sativa         -R-----------NSNEG--F----HHYTLIDGEMIVDTVP----D--L--GLKRRYLAY
O.latipes        -L-----------KDS----IKAQIHNNSRLDGLRIDNDAWKT--N--S--NLNPTYFIS
P.troglodytes    -R-----------KD------LRMHLSNTLLDGEMIIDR---V--N--G--QAVPRYLIY
P.falciparum     NVNGVDVNGVNINQDFN----NHNENNNLLMNQGILIDE---N--N--N--GIQ------
P.abelii         -R-----------KD------LRMHLSNTLLDGEMIIDR---V--N--G--QAVPRYLIY
S.cerevisiae     -L-----------PQKKKEELLETLQDGTLLDGELVIQT---N--PMTK--LQELRYLMF
S.pombe          -E-----------NDKSGK----KYHVDTLLDGELVLDI---Y--PG-G--KKQLRYLVF
S.purpuratus     -R-----------KG---------PHRDTLVDGEMIIDT---V--D--G--KSVARYLIY
S.scrofa         -R-----------KD------LRIHLSNTLLDGG-FLSF---V--A--G--CPV------
T.rubripes       -R-----------KD------PSTHLANTLLDGEMIIDK---V--N--G--QPVPRYLIY
X.tropicalis     -R-----------KD------LQHHLANTLLDGEMIIDK---V--N--G--QVVPRYLIY
                                                                             


                       3790      3800      3810      3820      3830      3840
                 =========+=========+=========+=========+=========+=========+
A.carolinensis   DIIKFNGQ-PVGDCD-FN-IRLACIE---KEIIFPRHDKIK--------NGQIDKA-KEP
A.gambiae        DVIYLHNR-EVRKQR-FFPDRLGLID---REIIQPRTNAIR--------QGRLDRE-SEP
A.thaliana       DMVAINGE-SVVERT-FC-ERWNMFV---REVIGPRAAEKL-------RSHCYRYD-LEP
B.distachyon     DLMALNFS-SKVKLP-FS-DRWKLLE---DEIIRPRIHERKQFETGLKGNPSYRYD-LEL
C.elegans        DIMRFNSV-NVMKEP-FY-KRFEIIK---TEIIDMRTAAFK--------TGRLKHE-NQI
C.jacchus        DIIKFNGQ-PVGDCD-FN-VRLQCIE---REIINPRHEKMK--------TGLIDKT-QEP
C.neoformans     DCMVLHGE-NIMEKP-LL-KRYARLH---DWVIKPFATALS--------ANPDMRR-TIP
P.tetraurelia    ILIHVISQKKTRDNFYGKIMFISIMIKKILASIDQTICIDNKDYYGNKRFELSGQIMIK-
D.rerio          DIIKFSGQ-PVGQCD-FN-RRLLCIE---KEIISPRFEKMK--------LGQIDKA-KEP
D.discoideum     DVLSVKNE-LHHQKL-LK-DRLTEIG---NVVSTLRSNLKV----------------DTP
D.melanogaster   DIVRLSNR-DVRDEP-FYPNRLDYIK---TEVIGPRILGMK--------HGIINQR-LQA
E.siliculosus    DILRNRER-NLVPCG-FL-DRLSVLQ---KEIIPAYVDRVR--------EGGAEAAPDGH
G.gallus         DIIKFNGQ-PVGDCD-FN-VRLSCIE---KEIIFPRHEKMK--------TGHIDKA-QEP
G.gorilla        DIIKFNSQ-PVGDCD-FN-VRLQCIE---REIISPRHEKMK--------TGLIDKT-QEP
H.sapiens        DIIKFNSQ-PVGDCD-FN-VRLQCIE---REIISPRHEKMK--------TGLIDKT-QEP
I.tridecemlinea  ------------------------------------------------------------
M.mulatta        DIIKFNSQ-PVGDCD-FN-VRLQCIE---REIINPRHEKMK--------TGLIDKT-QEP
M.gallopavo      DIIKFNGQ-PVGDCD-FN-VRLSCIE---KEIIFPRHEKMK--------TGHIDKA-QEP
M.musculus       DIIKFNAQ-PVGDCD-FN-IRLQCIE---REIISPRHEKMK--------TGLIDKT-QEP
O.sativa         DLMSINSQ-SVVKLP-FS-ERWKLLD---DEIIRPRYHDKG-------RSPSYKYD-MEL
O.latipes        AVLHTHRK-MKRDCT-LL-QKLNCTQ---KQLTTVKYNLSA--------QGCFSLR-QNP
P.troglodytes    DIIKFNSQ-PVGDCD-FN-VRLQCIE---REIISPRHEKMK--------TGLIDKT-QEP
P.falciparum     NIGTNDNINSLNNCNLLL-YKREEHR---EEKEYEEEEDER-SYSSDDTASTIHEE-EIP
P.abelii         DIIKFNSQ-PVGDCD-FN-VRLQCIE---REIISPRHEKMK--------TGLIDKT-QEP
S.cerevisiae     DCLAINGR-CLTQSP-TS-SRLAHLG---KEFFKPYFDLRA--------AYPNRCT-TFP
S.pombe          DCLACDGI-VYMSRL-LD-KRLGIFA---KSIQKPLDEYTK--------THMRETA-IFP
S.purpuratus     DIIKYWGK-PVGGCD-FG-WRRRCIQ---DEIIAPREVELQ--------KGSIDRM-REP
S.scrofa         ------------------------------------------------------------
T.rubripes       DIIKFNGQ-PVGQCN-FN-IRLLCIE---KEIITPRMEKMK--------SGQIDKT-KEP
X.tropicalis     DIIKFNGQ-PVGDCD-FN-IRLSCIE---KEIISPRHEKMK--------TGLIDKA-KEP
                                                                             


                       3850      3860      3870      3880      3890      3900
                 =========+=========+=========+=========+=========+=========+
A.carolinensis   FSVRHK-P------FFDIHVAKKLLEG-------N------FAKEVSHEVDGLIFQPT-G
A.gambiae        FGVRLK-Q------FWDIMQSRALLGP-------K------FTKGLGHEPDGLIFQPSID
A.thaliana       FAVRMK-G------FWLLSTVEKLLKN--------------TIPSLSHEADGLIFQGWDD
B.distachyon     FSVRRK-D------FWLLSTVKKLLKE--------------FIPALSHESDGLIFQGWDD
C.elegans        MSVRRK-D------FYDLEATAKLFGP-------K------FVQHVGHEIDGLIFQPKKT
C.jacchus        FSVRNK-P------FFDICISRKLLEG-------N------FAKEVSHEMDGLIFQPT-G
C.neoformans     FMMVAK-R------EELSYHLRFVMEE--------------HIPKLKHGHDGLIFTCVHT
P.tetraurelia    LFENVFKRSVFQMEKKYFSLSKK-IKGRIECDVFSFFHS---------------N--FIS
D.rerio          FSVRNK-P------FFDIHAARKLLEG-------S------FTSQVSHEVDGLIFQPI-G
D.discoideum     FDILGK-S------FQLKSKIVNLFKNIKEYP---NGERVYSDGKRCHNTDGIIFTPN-I
D.melanogaster   FSVRGK-D------FWDIWMSARLLGE-------K------FSRTLAHEPDGLIFQPSKQ
E.siliculosus    LMLVPK-R------FFPRQKIMDLFRQV--LVEGQHRIFRDEERSLHHKTDGIIFQPD-A
G.gallus         FSVRNK-P------FFDIYASRKLLEG-------S------FAREVSHEVDGLIFQPT-G
G.gorilla        FSVRNK-P------FFDICTSRKLLEG-------N------FAKEVSHEMDGLIFQPT-G
H.sapiens        FSVRNK-P------FFDICTSRKLLEG-------N------FAKEVSHEMDGLIFQPT-G
I.tridecemlinea  ------------------------------------------------------------
M.mulatta        FSVRNK-P------FFDICTSRKLLEG-------N------FAKEVSHEMDGLIFQPT-G
M.gallopavo      FSVRNK-P------FFDIYASRKLLEG-------S------FAREVSHEVDGLIFQPT-G
M.musculus       FSVRPK-Q------FFDINISRKLLEG-------N------FAKEVSHEMDGLIFQPI-G
O.sativa         FSVRRK-D------FWQLSAVNKILKE--------------FIPKLCHESDGLILQGWDD
O.latipes        FVSLYC-S------IFLSSGSAELLEG-------S------FTSQVSHEVDGLIFQPC-G
P.troglodytes    FSVRNK-P------FFDICTSRKLLEG-------N------FAKEVSHEMDGLIFQPT-G
P.falciparum     FEIYLK-D------FYPIEKICELIK---------------IMKKLPHYSDGIIFTPLHS
P.abelii         FSVRNK-P------FFDICTSRKLLEG-------N------FAKEVSHEMDGLIFQPT-G
S.cerevisiae     FKISMK-H------MDFSYQLVKVAKS---------------LDKLPHLSDGLIFTPVKA
S.pombe          FLTSLK-K------MELGHGILKLFNE--------------VIPRLRHGNDGLIFTCTET
S.purpuratus     FGIREK-P------FWDITSSKKILDG-------S------FSQELMHETDGLIFQPHKD
S.scrofa         ------------------------------------------------------------
T.rubripes       FSIRHK-S------FFDIHASRKLLEG-------S------FTSQVSHEVDGLIFQPC-G
X.tropicalis     FSVRNK-P------FFDIHAARKLLEG-------S------FAREVSHEVDGLIFQPI-G
                                                                             


                       3910      3920      3930      3940      3950      3960
                 =========+=========+=========+=========+=========+=========+
A.carolinensis   KYKPGRCD-DILKWKPPSLNSVDFRLKITR------------VGG----EGLLPQNV-GL
A.gambiae        PYESGVCP-RVLKWKPHHMNSIDFRLVIKE------------ETG----QGMLPTKK-GL
A.thaliana       PYVPRTHK-GLLKWKYAEMNSVDFLYEMGE------------EEG----RGFL------F
B.distachyon     PYVNRTHE-GLLKWKYPEMNSVDFLFETGS------------EN-----RQLI------F
C.elegans        KYETGRCD-KVLKWKPPSHNSVDFLLKVEK------------KCK----EGMLPEWI-GY
C.jacchus        KYKPGRCD-DILKWKPPSLNSVDFRLKITR------------MGG----EGLLPQNV-GL
C.neoformans     PYVAGTD-ENILKWKPPSENSIDFKVELRFPPLADSDEP---------DYRAKPEFL-LN
P.tetraurelia    SSFEYSLSSGNWILSKFSQEKQGMTQILSRLSFISSISSVN-------------------
D.rerio          KYKPGRCD-DILKWKPPSHNSVDFRLKITK------------VGG----EGLIPQTV-GL
D.discoideum     AYSNYTVH-TLFKWKYCDKWTIDFKVRDRGQ------------KG----WYLSCVA-NDN
D.melanogaster   PYTAGTCS-DVFKWKPHELNSVDFRLKIIT------------ERG----EGLLTKKV-GF
E.siliculosus    PYKVGTDT-ALLKWKWVDLASVDLRAYPATA------------AV----GGGGGVRLCSE
G.gallus         KYKPGRCD-DILKWKPPSLNSVDFRLKITR------------IGG----EGLLTQNV-GL
G.gorilla        KYKPGRCD-DILKWKPPSLNSVDFRLKITR------------MGG----EGLLPQNV-GL
H.sapiens        KYKPGRCD-DILKWKPPSLNSVDFRLKITR------------MGG----EGLLPQNV-GL
I.tridecemlinea  ------------------------------------------------------------
M.mulatta        KYKPGRCD-DILKWKPPSLNSVDFRLKITR------------MGG----EGLLPQNV-GL
M.gallopavo      KYKPGRCD-DILKWKPPSLNSVDFRLKITR------------IGG----EGLLTQNV-GL
M.musculus       KYKPGRCD-DILKWKPPSLNSVDFRLKITR------------MGG----EGLLPQNV-GL
O.sativa         PYVTRTHE-GLLKWKYPEMNSVDFLFEIGS------------EN-----RQFI------F
O.latipes        RYKAGRCD-DILKWKPPNLNSVDFRLKITK------------VGG----EGLLTQTV-GL
P.troglodytes    KYKPGRCD-DILKWKPPSLNSVDFRLKITR------------MGG----EGLLPQNV-GL
P.falciparum     PYITGNFY-QLLKWKPLNLNTVDFGIETIYD------------E-----YNIPSKFE-LF
P.abelii         KYKPGRCD-DILKWKPPSLNSVDFRLKITR------------MGG----EGLLPQNV-GL
S.cerevisiae     PYTAGGKDSLLLKWKPEQENTVDFKLILDIPMVEDPSLPKDDRNRWYYNYDVKPVFS-LY
S.pombe          PYVSGTD-QSLLKWKPKEMNTIDFMLKLEFAQPEEGDI----------DYSAMPEFQ-LG
S.purpuratus     PYIPGRCD-LILKWKPPSLNSVDFRIKVTV------------VKR----EGCIPETL-GL
S.scrofa         ------------------------------------------------------------
T.rubripes       PYKPGRCD-DILKWKPPNLNSVDFRLKITK------------VTG----EGLLPKTY-GL
X.tropicalis     KYKAGRCD-DILKWKPPNLNSVDFLLKITK------------VGG----EGLLTRNV-GL
                                                                             


                       3970      3980      3990      4000      4010      4020
                 =========+=========+=========+=========+=========+=========+
A.carolinensis   -----LY---------VGSL-----DRP---F--------------AQI----KVTKDLK
A.gambiae        -----LY---------VGGM-----EHS---Y--------------GEI----KLTKELR
A.thaliana       -----LH---------ERGK-----KKL---M---E--------GYSVE---FRDDSDPS
B.distachyon     -----LY---------ERGK-----KKL---M---D--------GTRVV---FSDEVDPS
C.elegans        -----LF---------VQNL-----SDP---F--------------GTM----KATATLK
C.jacchus        -----LY---------VGGY-----ERP---F--------------AQI----KVTKELK
C.neoformans     -----TWLGG--------D-RYEF--------------FDFMAMTDDEW---QRFKESEE
P.tetraurelia    ------------------------------------------------------------
D.rerio          -----LY---------VGNY-----DMP---F--------------AQM----KITKDLK
D.discoideum     -----IE---------VDCR-----E---------------VNFSNDDL---QKLRREFQ
D.melanogaster   -----LY---------VGGH-----DAP---Y--------------GRM---QRLTKETR
E.siliculosus    -----AG---------NHGE-----EVD---------LSRTVHLSEHDQ---ARLVADMQ
G.gallus         -----LY---------VGNF-----DRP---F--------------AQI----KVTKELK
G.gorilla        -----LY---------VGGY-----ERP---F--------------AQI----KVTKELK
H.sapiens        -----LY---------VGGY-----ERP---F--------------AQI----KVTKELK
I.tridecemlinea  ------------------------------------------------------------
M.mulatta        -----LY---------VGGY-----ERP---F--------------AQI----KVCL---
M.gallopavo      -----LY---------VGNF-----DRP---F--------------AQI----KVTKELK
M.musculus       -----LY---------VGGY-----ERP---F--------------AQI----KVTKELK
O.sativa         -----LY---------ERGR-----KKL---M---D--------GARVV---FPDEVDPP
O.latipes        -----LY---------VGNY-----DMP---F--------------AKM----KATKELK
P.troglodytes    -----LY---------VGGY-----ERP---F--------------AQI----KVTKELK
P.falciparum     ISINGVR---------TS-Y-----KCY---L-------AE----YGDVYKELLQLAISN
P.abelii         -----LY---------VGGY-----ERP---F--------------AQI----KVTKELK
S.cerevisiae     -----VWQGGADVNSRLKHFDQPFDRKEFEILERTYRKFAELSVSDEEW---QNLKNLEQ
S.pombe          -----VWEGR--------N-MYSF--------------FAFMYVDEKEW---EKLKSFNV
S.purpuratus     -----LF---------VGGQ-----QQP---F--------------GQM----KITKDLK
S.scrofa         ------------------------------------------------------------
T.rubripes       -----LY---------VGSY-----NQP---F--------------AEI----KVTKELT
X.tropicalis     -----LY---------VGKY-----DCP---F--------------SEI----KVTKDLK
                                                                             


                       4030      4040      4050      4060      4070      4080
                 =========+=========+=========+=========+=========+=========+
A.carolinensis   Q---YDNKIIECKFE---N------------NS-------WVFMRQRIDKSFPNAYSTAM
A.gambiae        K---LNNKIIECKFE---N-------------G-------WVLMRERTDKSFPNSYETAK
A.thaliana       S---YNGKIVECAWD---K-DK---------KV-------WFSMRIRVDKTTPNDINTAR
B.distachyon     L---ISGKIVECSWN---K-QE---------DC-------WSCMRIRADKSTPNDINTYR
C.elegans        K---YHNKIIECTLLVDNQGRP---------KE-------WKFMRERTDKSLPNGLRTAE
C.jacchus        Q---YDNKIIECKFE---N------------NS-------WVFMRQRTDKSFPNAYNTAM
C.neoformans     Q---LDERIVEVCWD---S-QI---------QA-------WKMLRMRDDKPHGNHKSIVD
P.tetraurelia    ------------------------------------------------------------
D.rerio          Q---YDNKIIECTFV---N------------NT-------WVFMRQRVDKSFPNAYDTAM
D.discoideum     RARDTSTVVAECSFQ---P-KW---------GT-------WKFHQVRHDKKKGNYISIVM
D.melanogaster   E---LDNRIVECTMN---Q--F---------GN-------WDFMRERTDKKNPNSYNTAR
E.siliculosus    G---SRSVIAEMALD---P-GS---------GL-------WVYMGLRPDKDRPNFITTVI
G.gallus         Q---YDNKIIECKFE---N------------NS-------WVFMRQRIDKSFPNAYSTAM
G.gorilla        Q---YDNKIIECKFE---N------------NS-------WVFMRQRTDKSFPNAYNTAM
H.sapiens        Q---YDNKIIECKFE---N------------NS-------WVFMRQRTDKSFPNAYNTAM
I.tridecemlinea  ------------------------------------------------------------
M.mulatta        -------FIFRVLFL---D-----------------------VLLSRIHQKLANNHQHIK
M.gallopavo      Q---YDNKIIECKFE---N------------NS-------WVFMRQRIDKSFPNAYSTAM
M.musculus       Q---YDNKIIECKFE---N------------NS-------WVFMRQRIDKSFPNAYNTAM
O.sativa         S---ISGKIVECSWN---K-EE---------DC-------WSCMRIRTDKSTPNDINTYR
O.latipes        Q---YDNKIIECTFA---N------------NT-------WVFMRQRVDKSFPNSYDTAM
P.troglodytes    Q---YDNKIIECKFE---N------------NS-------WVFMRQRTDKSFPNAYNTAM
P.falciparum     K---ISHYIIECYYV---S-KNIFSICKGENGREQKVEGGWIAQKIRFDKNIPNDISTLN
P.abelii         Q---YDNKIIECKFE---N------------NS-------WVFMRQRTDKSFPNAYNTAM
S.cerevisiae     P---LNGRIVECAKN---Q-ET---------GA-------WEMLRFRDDKLNGNHTSVVQ
S.pombe          P---LSERIVECYL----D-DE---------NR-------WRFLRFRDDKRDANHISTVK
S.purpuratus     Q---YDNKIIECSYN---N--K---------KG-------WQFMRERTDKSFPNGYKTAV
S.scrofa         ------------------------------------------------------------
T.rubripes       Q---YDNKIIECTFA---N------------NS-------WVFMRQRVDKSFPNSYDTAM
X.tropicalis     Q---YENKIIECKFE---N------------NR-------WVFMRQRVDKSFPNSYDTAL
                                                                             


                       4090      4100      4110      4120      4130      4140
                 =========+=========+=========+=========+=========+=========+
A.carolinensis   AVCNSIRYPVTKEILFEFIDKCMTGATPAA--------TPEQ--------------TRKH
A.gambiae        NVWESIRNPVTEDRLLTLIAKDG-------------------------------------
A.thaliana       KVIKSINDNITEEVLLQEIREII-----RLP--------MYA--------------DRIR
B.distachyon     KVMRSITDNITEDKLLAEMNEIS-----SLP--------MYA--------------DRKA
C.elegans        NVVETMVNPVTETYLIEYVNHAL-----RVL---KRAAAAHR--------------H---
C.jacchus        AVCNSISNPVTKEMLFEFIDRCA-----AA--------SQGQ--------------KRKH
C.neoformans     KILVSINDGVEIEELYARADTIK-----AAWKERAK----------------------QR
P.tetraurelia    ------------------------------------------------------------
D.rerio          AVCNSIQHPVTKEILLEFLERCA----------------QVQ--------------SRKN
D.discoideum     DTMESIAENLSSDELKYRIPLL-P----HD--------DNWEEEMSRIRSQL--IN-NIK
D.melanogaster   SVVDSIKHPVTKEYLLNFIASSG-------------------------------------
E.siliculosus    STMVEVAEGLSEEELKYRMLAESP----AS--------DDWARQEMTMRKRAVQWQYKRK
G.gallus         AVCNSIQNPVTKEILFEFIDRCM-----AA--------SQGQ--------------TRKH
G.gorilla        AVCNSISNPVTKEMLFEFIDRCT-----AA--------SQGQ--------------KRKH
H.sapiens        AVCNSISNPVTKEMLFEFIDRCT-----AA--------SQGQ--------------KRKH
I.tridecemlinea  ------------------------------------------------------------
M.mulatta        ILCPSTS--------------------------------GGQ--------------DRWI
M.gallopavo      AVCNSIQNPVTKEILFEFIDRCM-----AA--------SQGQ--------------TRKH
M.musculus       AVCNSISNPVTKEMLFEFIDRCA-----AA--------AQGQ--------------KRKY
O.sativa         KVMRSITDNITEDKLLEEIYEIM-----NLP--------MYA--------------DRKA
O.latipes        AVCKSIQEPVTKAILLEYVDRCA-----QG----------AQ--------------NHKR
P.troglodytes    AVCNSISNPVTKEMLFEFIDRCT-----AA--------SQGQ--------------KRKH
P.falciparum     KVIQSILDNITIDSLIKEISRN-----------------------------------RKA
P.abelii         AVCNSISNPVTKEMLFEFIDRCT-----AA--------SQGQ--------------KRKH
S.cerevisiae     KVLESINDSVSLEDLEEIVGDIK-----RCWDERRANMAGGS--------------GRPL
S.pombe          SVLQSIEDGVSKEDLLKEMPIIR-----EAYYNRKK-P--SV---------------TKR
S.purpuratus     AVCQSIQNPVTKDFLMNFIDKEA-----IK--------PSSN--------------KRPA
S.scrofa         ------------------------------------------------------------
T.rubripes       AVCKSIKHPVTKEYLLQYVDHCI----------------QAQ--------------NRKR
X.tropicalis     AVCNSIRNPVTKEILFELIDRCT-----AG--------IQRQ--------------SRKN
                                                                             


                       4150      4160      4170      4180      4190      4200
                 =========+=========+=========+=========+=========+=========+
A.carolinensis   HMDPDTEL-MPP-PPPK----------RPR------------------------------
A.gambiae        -FRSDAEL-MPP-PR-------------------------P-------------------
A.thaliana       -------N-DSQ---AA----------RR-------------------------------
B.distachyon     HADRKAHA-EKM-AQQH----------RR-------------------------------
C.elegans        HQIHQQQL-HEG-EPEA----------RRQK-----------------------------
C.jacchus        HLDPDTEL-MPP-PPPK----------RPRPS----------------------------
C.neoformans     S----------QQ-QPPQN-QKPPPQAYGQDLYTPGHGYAHTGYNPDHSH-PPPQQGIMS
P.tetraurelia    ------------------------------------------------------------
D.rerio          P--ADSDL-MPP-PPPK----------RSANSI---------------------------
D.discoideum     PSKSSTST-YQP--FP--------------------------------------------
D.melanogaster   -YRNDHVM-MPP-PINS----------HNQNHNH-NHFYPHGHGQHQRRPCVNP-TLG--
E.siliculosus    STQPQQLQ-QPP-PPPP-------------------------------------------
G.gallus         HLDPDTEL-MPP-PPPK----------RPRTI----------------------------
G.gorilla        HLDPDTEL-MPP-PPPK----------RPRPL----------------------------
H.sapiens        HLDPDTEL-MPP-PPPK----------RPRPL----------------------------
I.tridecemlinea  ------------------------------------------------------------
M.mulatta        ------------------------------------------------------------
M.gallopavo      HLDPDTEL-MPP-PPPK----------RPRTI----------------------------
M.musculus       PLDPDTEL-MPP-PPPK----------RLHRP----------------------------
O.sativa         K----AHA-RSM-AQQR----------R--------------------------------
O.latipes        PSDPDSQL-MPP-PPPK----------RSNRDI---------------------------
P.troglodytes    HLDPDTEL-MPP-PPPK----------RPRPL----------------------------
P.falciparum     K-----------------------------------------------------------
P.abelii         HLDPDTEL-MPP-PPPK----------RPRPL----------------------------
S.cerevisiae     PSQ-SQNATL-STSKPV-H-SQPP---------SN-DKE---------PK-YVDEDD---
S.pombe          KLD-ETSNDD-APAIKK-VAKESE------------------------------KE----
S.purpuratus     DNSSSSSS-SPR-PPPA----------KQQRTDS-HPPSSLASGPGRPSSSLMP-PPPAP
S.scrofa         ------------------------------------------------------------
T.rubripes       PPDPDSEL-MPP-PPPK----------RATRPI---------------------------
X.tropicalis     PLDPDAEL-MPP-PPPK----------IP-------------------------------
                                                                             


                       4210      4220      4230      4240      4250      4260
                 =========+=========+=========+=========+=========+=========+
A.carolinensis   ---P------------------------------------------------M-------
A.gambiae        ---H--------------------------------------------------------
A.thaliana       ---R--------------------------------------------------------
B.distachyon     ---R------------------------------------------------M-------
C.elegans        ---L------------------------------------------------M-------
C.jacchus        ---S------------------------------------------------M-------
C.neoformans     GLRRMPPKAPVKRVAASSSTTAPRKAPRAGPSTTTTTAKRPATTSSSVAKGKA-------
P.tetraurelia    ------------------------------------------------------------
D.rerio          --PQ--------------------------------------------------------
D.discoideum     ---Q-----------------------------------MDPESVGYCNNPDF-------
D.melanogaster   ---N------------------------------------------------M-------
E.siliculosus    ---RMSYEDEGDDVFGDAVPEGEAGVEEDDYGDGGGAAGGGGG---------YDNMDEEE
G.gallus         ---T------------------------------------------------M-------
G.gorilla        ---T------------------------------------------------M-------
H.sapiens        ---T------------------------------------------------M-------
I.tridecemlinea  ----------------------------------------------------M-------
M.mulatta        ---T------------------------------------------------M-------
M.gallopavo      ---T--------------------------------------------------------
M.musculus       ---T------------------------------------------------M-------
O.sativa         ---R------------------------------------------------M-------
O.latipes        --P-------------------------------------------------M-------
P.troglodytes    ---T------------------------------------------------M-------
P.falciparum     ----------------------------------------------------M-------
P.abelii         ---T------------------------------------------------M-------
S.cerevisiae     -WSD--------------------------------MVAATKR--RKTHIHKH-------
S.pombe          ---I----------------------------MGVNTAGDPQKSQPKINKGGI-------
S.purpuratus     -LHH--------------------------------MSSRAKSKEKNVKDEEK-------
S.scrofa         ---P--------------------------------------------------------
T.rubripes       ---P------------------------------------------------M-------
X.tropicalis     ---R------------------------------------------------M-------
                                                                             


                       4270      4280      4290      4300      4310      4320
                 =========+=========+=========+=========+=========+=========+
A.carolinensis   -------------------------------------D--D--------LV---------
A.gambiae        -----------------------------------------VKQEQQDEDA---------
A.thaliana       --------------------------------MGLDQEDLD---------L---------
B.distachyon     ------------------------------PPVMPGPD--A--------VA---------
C.elegans        -------------------------------------T--I--------GQ---------
C.jacchus        -------------------------------------D--V--------LA---------
C.neoformans     --------------PARPSASTQVDVKPRIEDTRG-EE--E--------WA---------
P.tetraurelia    ------------------------------------------------------------
D.rerio          ----------------------------------------M--------LQ---------
D.discoideum     -------------------------------------L--H--------KG---------
D.melanogaster   -------------------------------------V--ELKMGDHNVEA---------
E.siliculosus    EEEDERGYENGAAGGGGGEGLGLGE-----GEEEE-YD--P--------DA---------
G.gallus         -------------------------------------E--A--------LA---------
G.gorilla        -------------------------------------D--V--------LA---------
H.sapiens        -------------------------------------D--V--------LA---------
I.tridecemlinea  -------------------------------------E--V--------LA---------
M.mulatta        -------------------------------------D--V--------LA---------
M.gallopavo      -------------------------------------------------MH---------
M.musculus       -------------------------------------D--V--------LA---------
O.sativa         ------------------------------ERTEE-EE--A--------PA---------
O.latipes        -------------------------------------E--V--------LG---------
P.troglodytes    -------------------------------------D--V--------LA---------
P.falciparum     ---------------------------------K------D--------IIKIKAKRKNV
P.abelii         -------------------------------------D--V--------LA---------
S.cerevisiae     ---------------------------------VK-DE--A--------FD---------
S.pombe          ---------------------------------GK-DE--S--------FG---------
S.purpuratus     -------------------------------------D--F--------EN---------
S.scrofa         ------------------------------------------------------------
T.rubripes       -------------------------------------E--I--------LG---------
X.tropicalis     -------------------------------------E--V--------LD---------
                                                                             


                       4330      4340      4350      4360      4370      4380
                 =========+=========+=========+=========+=========+=========+
A.carolinensis   -------------------------------AELGAS-----TPEQ-----LA-------
A.gambiae        -------------------------------PPVEK------QKPD-----FG-------
A.thaliana       -------------------------------TND-DHFIDK---EK-----LS-------
B.distachyon     -------------------------------AEGDPDALPVPTYRS-----LA-------
C.elegans        --------------------------VNGRARIKSEC-----GKDQ-----KC-------
C.jacchus        -------------------------------EEFGNL-----TPEQ-----LA-------
C.neoformans     -------------------------------DLMKQQ-----YGDKKGADWYS-------
P.tetraurelia    ------------------------------------------------------------
D.rerio          -------------------------------EEFGEM-----SPQQ-----LA-------
D.discoideum     -------------------------------SMDELY-----NISK-----LT-------
D.melanogaster   -------------------------------TTWDPG-----DSKD-----WS-------
E.siliculosus    -------------------------------AYGAGG---LMDDED-----EL-------
G.gallus         -------------------------------AELGAV-----TAER-----AA-------
G.gorilla        -------------------------------EEFGNL-----TPEQ-----LA-------
H.sapiens        -------------------------------EEFGNL-----TPEQ-----LA-------
I.tridecemlinea  -------------------------------EEFGNL-----TPEQ-----LA-------
M.mulatta        -------------------------------EEFGNL-----TPEQ-----LA-------
M.gallopavo      -------------------------------TNHTLR-----NKEA-----QQ-------
M.musculus       -------------------------------EEFGSL-----TPEQ-----LT-------
O.sativa         -------------------------------AKDPNASLPSPTYRS-----LA-------
O.latipes        -------------------------------EEFGDM-----TPQE-----LA-------
P.troglodytes    -------------------------------EEFGNL-----TPEQ-----LA-------
P.falciparum     NKINEENEEEIYQLKENNRAQNDDDDDDYNNNEKNKI-----NKII-----LKDENINSK
P.abelii         -------------------------------EEFGNL-----TPEQ-----LA-------
S.cerevisiae     -------------------------------DLLKPV-----YKGKK----LT-------
S.pombe          -------------------------------ALFKPV-----YKGKK----LA-------
S.purpuratus     -------------------------------EEAMEI-----DKEL-----LS-------
S.scrofa         ------------------------------------------------------------
T.rubripes       -------------------------------AEFCDM-----SPQE-----LA-------
X.tropicalis     -------------------------------VEFGHL-----SQEE-----LI-------
                                                                             


                       4390      4400      4410      4420      4430      4440
                 =========+=========+=========+=========+=========+=========+
A.carolinensis   -----------------------------A---P--------------------------
A.gambiae        -----------------------------L---S--------------------------
A.thaliana       -----------------------------A---P--------------------------
B.distachyon     -----------------------------A---P--------------------------
C.elegans        -----------------------------M---P--------------------------
C.jacchus        -----------------------------A---P--------------------------
C.neoformans     -----------------------------K---G--------------------------
P.tetraurelia    ------------------------------KINSTYNKSTKILTLRSLYPSQWGVVCPSD
D.rerio          -----------------------------A---P--------------------------
D.discoideum     -----------------------------D---D--------------------------
D.melanogaster   -----------------------------V---P--------------------------
E.siliculosus    -----------------------------DPLEE--------------------------
G.gallus         -----------------------------A---P--------------------------
G.gorilla        -----------------------------A---P--------------------------
H.sapiens        -----------------------------A---P--------------------------
I.tridecemlinea  -----------------------------A---P--------------------------
M.mulatta        -----------------------------A---P--------------------------
M.gallopavo      -----------------------------A---E--------------------------
M.musculus       -----------------------------A---P--------------------------
O.sativa         -----------------------------A---P--------------------------
O.latipes        -----------------------------A---P--------------------------
P.troglodytes    -----------------------------A---P--------------------------
P.falciparum     NDYNNMMNPYNVIGKSGNEEDTHIKIEEEK---NKDNLYECKNDDNKFYESKINFIKKQD
P.abelii         -----------------------------A---P--------------------------
S.cerevisiae     -----------------------------D---E--------------------------
S.pombe          -----------------------------D---P--------------------------
S.purpuratus     -----------------------------A---P--------------------------
S.scrofa         ------------------------------------------------------------
T.rubripes       -----------------------------A---P--------------------------
X.tropicalis     -----------------------------A---P--------------------------
                                                                             


                       4450      4460      4470      4480      4490      4500
                 =========+=========+=========+=========+=========+=========+
A.carolinensis   ------------------------------------------------------------
A.gambiae        ------------------------------------------------------------
A.thaliana       ------------------------------------------------------------
B.distachyon     ------------------------------------------------------------
C.elegans        ------------------------------------------------------------
C.jacchus        ------------------------------------------------------------
C.neoformans     ------------------------------------------------------------
P.tetraurelia    TPEGESCGIIKNLSILTYISLDEKNK-YIKNLCFDLGVEFL---SFSKVKTVPMDMVFLN
D.rerio          ------------------------------------------------------------
D.discoideum     ------------------------------------------------------------
D.melanogaster   ------------------------------------------------------------
E.siliculosus    ------------------------------------------------------------
G.gallus         ------------------------------------------------------------
G.gorilla        ------------------------------------------------------------
H.sapiens        ------------------------------------------------------------
I.tridecemlinea  ------------------------------------------------------------
M.mulatta        ------------------------------------------------------------
M.gallopavo      ------------------------------------------------------------
M.musculus       ------------------------------------------------------------
O.sativa         ------------------------------------------------------------
O.latipes        ------------------------------------------------------------
P.troglodytes    ------------------------------------------------------------
P.falciparum     EANNEDNNNNNNNNNNNCYYNNYENKHLNIFNNKNEVNNKNLSSNKILKDEYYDKNFNED
P.abelii         ------------------------------------------------------------
S.cerevisiae     ------------------------------------------------------------
S.pombe          ------------------------------------------------------------
S.purpuratus     ------------------------------------------------------------
S.scrofa         ------------------------------------------------------------
T.rubripes       ------------------------------------------------------------
X.tropicalis     ------------------------------------------------------------
                                                                             


                       4510      4520      4530      4540      4550      4560
                 =========+=========+=========+=========+=========+=========+
A.carolinensis   ------------------------------------------------------VNTVEE
A.gambiae        ------------------------------------------------------GKLTEE
A.thaliana       ------------------------------------------------------IKSTAD
B.distachyon     ------------------------------------------------------VRRPVD
C.elegans        ---------------------------------------------------------LED
C.jacchus        ------------------------------------------------------IPTVEE
C.neoformans     ------------------------------------------------------VKTVED
P.tetraurelia    NNYIGLHANTPRFLFVFRKMRRDTIISQNTSIFWDTVNRFVFIWTDRGRACRPFFIIKLN
D.rerio          ------------------------------------------------------VDTVEE
D.discoideum     ------------------------------------------------------IKPVEE
D.melanogaster   ------------------------------------------------------IKPLTE
E.siliculosus    ------------------------------------------------------DISQED
G.gallus         ------------------------------------------------------VSAVEE
G.gorilla        ------------------------------------------------------IPTVEE
H.sapiens        ------------------------------------------------------IPTVEE
I.tridecemlinea  ------------------------------------------------------IPTVEE
M.mulatta        ------------------------------------------------------IPTVEE
M.gallopavo      ------------------------------------------------------WGYIKE
M.musculus       ------------------------------------------------------IPTVEE
O.sativa         ------------------------------------------------------VTKPVD
O.latipes        ------------------------------------------------------VNTVAD
P.troglodytes    ------------------------------------------------------IPTVEE
P.falciparum     NINNLKEFIHNENKKPKTGKLYNNMNILNDEENFSGLVIKNKEHNKKIYENSKCVNTLNE
P.abelii         ------------------------------------------------------IPTVEE
S.cerevisiae     ------------------------------------------------------INTAQD
S.pombe          ------------------------------------------------------VPTIED
S.purpuratus     ------------------------------------------------------IKAVKD
S.scrofa         ------------------------------------------------------------
T.rubripes       ------------------------------------------------------VNTVAE
X.tropicalis     ------------------------------------------------------VDTVQE
                                                                             


                       4570      4580      4590      4600      4610      4620
                 =========+=========+=========+=========+=========+=========+
A.carolinensis   KWRLLPAFLKVKGLVKQHIDSFNYFINVEIKKIMKA--N-EKIT---------SDADPMW
A.gambiae        TNKVN----------GQHIDSFNYFINVDIKKIVQA--N-QEVK---------SDADPCF
A.thaliana       KFQLVPEFLKVRGLVKQHLDSFNYFINVGIHKIVKA--N-SRIT---------STVDPSI
B.distachyon     KFELLPAFLKVRGLVKEHIDSFNYFITKGIKNIVQA--N-NRIE---------ARSDPSI
C.elegans        KWLLVPAFLKVRGLVKQHLVSFDHFVQEEIRSIMLS--N-QKIT---------SDANPNF
C.jacchus        KWRLLPAFLKVKGLVKQHIDSFNYFINVEIKKIMKA--N-EKVT---------SDADPMW
C.neoformans     KWQLLPAFLKVKGLVKQHLDSFNYFVNVDIKAILAA--N-SLVI---------SDINPKY
P.tetraurelia    ESI--DKFKKRNTAKIFSWKNLQRENFVEFLDTYEQNNALIANESKKIN-----------
D.rerio          KWKLLPAFLKVKGLVKQHIDSFNYFINVEIKKIMKA--N-EKIT---------SDADPMW
D.discoideum     KWKLVPAFMKCRGLVKQHIDSFNFFINVEMKKIVKA--N-ERLT---------AENDPSY
D.melanogaster   KWKLVPAFLQVKGLVKQHIDSFNHFINVDIKKIVKA--N-ELVT---------SGADPLF
E.siliculosus    AWVVISAYFSEKGLVRQQLDSFDEFLQSTMHELVSSAGE-IKITPELQYMPGQDTVRR-T
G.gallus         KWRLLPAFLKVKGLVKQHIDSFNYFINVEIKKIMKA--N-EKVT---------SDADPMW
G.gorilla        KWRLLPAFLKLC---------------LEIQGIIR-------------------------
H.sapiens        KWRLLPAFLKVKGLVKQHIDSFNYFINVEIKKIMKA--N-EKVT---------SDADPMW
I.tridecemlinea  KWRLLPAFLKVKGLVKQHIDSFNYFINVEIKKIMKA--N-EKVT---------SDADPMW
M.mulatta        KWRLLPAFLKVKGLVKQHIDSFNYFINVEIKKIMKA--N-EKVT---------SDADPMW
M.gallopavo      KWRLLPAFLKVKGLVKQHIDSFNYFINVEIKKIMKA--N-EKVT---------SDADPMW
M.musculus       KWRLLPAFLKVKGLVKQHIDSFNYFINVEIKKIMKA--N-EKVT---------SDADPMW
O.sativa         KFALLPAFLKVRGLVKEHIDSFNYFITKGIRNIVKA--N-NRIE---------ARNNPSI
O.latipes        KWKLLPAFLKVKGLVKQHIDSFNYFINVEIKKIMKA--N-EKIT---------SDADPMW
P.troglodytes    KWRLLPAFLKVKGLVKQHIDSFNYFINVEIKKIMKA--N-EKVT---------SDADPMW
P.falciparum     KWKLLPAYLKVKGLVKQHIESYNYFIKREIKTIMNAT-TNKIIK---------SDIDEHF
P.abelii         KWRLLPAFLKVKGLVKQHIDSFNYFINVEIKKIMKA--N-EKVT---------SDADPMW
S.cerevisiae     KWHLLPAFLKVKGLVKQHLDSFNYFVDTDLKKIIKA--N-QLIL---------SDVDPEF
S.pombe          KWQLLPAFLKVKGLVKQHLDSYNYFVDVDLKKIVQA--N-EKVT---------SDVEPWF
S.purpuratus     KWKLLPAFLKVRGLVRQHIDSFNYFINVEVXHLISL--N-LSIY---------NKXTLLH
S.scrofa         ---------------------------------MKA--N-EKVT---------SDADPMW
T.rubripes       KWKLLPAFLKVKGLVKQHIDSFNYFINVEIKKIMKA--N-EKIT---------SDADPMW
X.tropicalis     KWKLLPAFLKVKGLVKQHIDSFNYFINDEIKKIMKA--N-EKVT---------SDADPMW
                                                                             


                       4630      4640      4650      4660      4670      4680
                 =========+=========+=========+=========+=========+=========+
A.carolinensis   -Y-LKYLNIYVGTPDVEESFNVTR-PVSPHECRLRDMTYSAPITVDIEYTRGS-------
A.gambiae        -Y-LKYLNVHVGKPDVEEGFNNTK-STTPHECRLRDMTYSAPITVDIEYTRGT-------
A.thaliana       -Y-LRFKKVRVGEPSIINVNTVEN--INPHMCRLADMTYAAPIFVNIEYVHGS-------
B.distachyon     -Y-LRYKDIRVGEPSVQVDFRVET--ITPHFCRLTDRTYSAPVIVDIEYTVGK-------
C.elegans        -Y-LKYLDIRIGKPSSEEGLNMTHDKITPQECRLRDMTYSAPISVDIEYTRGN-------
C.jacchus        -Y-LKYLNIYVGLPDVEESFNVTR-PVSPHECRLRDMTYSAPITVDIEYTRGS-------
C.neoformans     -Y-IRYTDIRVGRPARHDANQVAS-ALSPMECRLTDSTYSAPIYVDVEYMGEE-------
P.tetraurelia    SKTTHLEISPEIILGICANNIPFPNHNQSPRNTYQCAMGKQSIGFIGLNQIYRADTILSL
D.rerio          -Y-LKYLNIYVGMPDVEESFNVTR-PVSPHECRLRDMTYSAPITVDIEYTRGS-------
D.discoideum     -F-VRFTDINVGSPTSTEDNLDSV-QLTPQRCRLRDMTYSAPIFVNIEYTRNK-------
D.melanogaster   -Y-LKYLDVRVGKPDIDDGFNITK-ATTPHECRLRDTTYSAPITVDIEYTRGT-------
E.siliculosus    FQ-INFGQVYLAKPTAREKDGSLT-SMFPHEARLRNLTYNSPLYCDISCKTYEADVGDRS
G.gallus         -Y-LKYLNIYVGTPDVEESFNVTR-PVSPHECRLRDMTYSAPITVDIEYTRGS-------
G.gorilla        -----YLNIYVGLPDVEESFNVTR-PVSPHECRLRDMTYSAPITVDIEYTRGS-------
H.sapiens        -Y-LKYLNIYVGLPDVEESFNVTR-PVSPHECRLRDMTYSAPITVDIEYTRGS-------
I.tridecemlinea  -Y-LKYLNIYVGLPDVEESFNVTR-PVSPHECRLRDMTYSAPITVDIEYTRGS-------
M.mulatta        -Y-LKYLNIYVGLPDVEESFNVTR-PVSPHECRLRDMTYSAPITVDIEYTRGS-------
M.gallopavo      -Y-LKYLNIYVGTPDVEESFNVTR-PVSPHECRLRDMTYSAPITVDIEYTRGS-------
M.musculus       -Y-LKYLNIYVGLPDVEESFNVTR-PVSPHECRLRDMTYSAPITVDIEYTRGS-------
O.sativa         -F-LRYNSVRVGVPSVQVQYIAEK--ITPHFCRLTDRTYSAPVLADIEYTVGK-------
O.latipes        -Y-LKYLNIYVGMPDVEESFNVTR-PVSPHECRLRDMTYSAPITVDIEYTRGS-------
P.troglodytes    -Y-LKYLNIYVGLPDVEESFNVTR-PVSPHECRLRDMTYSAPITVDIEYTRGS-------
P.falciparum     -Y-VEFLDITVGTPSVEENMIETK--LTPQICRQRDLTYSAPIYVDVEYVKGN-------
P.abelii         -Y-LKYLNIYVGLPDVEESFNVTR-PVSPHECRLRDMTYSAPITVDIEYTRGS-------
S.cerevisiae     -Y-LKYVDIRVGK--KSSSSTKDY-LTPPHECRLRDMTYSAPIYVDIEYTRGR-------
S.pombe          -Y-LKYLDIRVGAPVRTDADAIQA-SISPHECRLRDLTYGANIYVDIEYTRGK-------
S.purpuratus     -LPLRYLNIYVGSPDVEESFNITK-PTSPHECRLRDMTYSAPITVDIEYTRGQ-------
S.scrofa         -Y-LKYLNIYVGLPDVEESFNVTR-PVSPHECRLRDMTYSAPITVDIEYTRGS-------
T.rubripes       -Y-LKYLNIYVGMPDVEESFNVTR-PVSPHECRLRDMTYSAPITVDIEYTRGS-------
X.tropicalis     -Y-LKYLNIYVGLPDVEESFNVTR-PVSPHECRLRDMTYSAPITVDIEYTRGS-------
                                           ##########################        


                       4690      4700      4710      4720      4730      4740
                 =========+=========+=========+=========+=========+=========+
A.carolinensis   --------QRIIR----NALPVGRMPIMLRSSNCVLTGKTPAEFAKLNECPLDPGGYFIV
A.gambiae        --------QRVVR----NGLLIGRMPIMLRSSNCVLTGKSDYQLSKVNECPMDPGGYFII
A.thaliana       --------HGNKAKSAKDNVIIGRMPIMLRSCRCVLHGKDEEELARLGECPLDPGGYFII
B.distachyon     --------THVVHR--KPNFIIGYMPIMLRSYACVLNGKDEAELARYGECPLDPGGYFVV
C.elegans        --------QRVFK----KDLIIGRMPIMLRSSKCILRDLAEEELARVQECPYDPGGYFVV
C.jacchus        --------QRIIR----NALPIGRMPIMLRSSNCVLTGKTPAEFAKLNECPLDPGGYFIV
C.neoformans     --------KRSKQ----RGVQIGMLPVMLRSDLCNLKGKNEAELARMGECPMDPGGYFVV
P.tetraurelia    LVYPQKPIVGTKITELSGNNRLTGGINACICVMSYRGHDIEDAMTLNRSSIQRGFMKSVL
D.rerio          --------QRIIR----NALPIGRMPIMLRSSNCVLTGKTPMEFSKLNECPLDPGGYFIV
D.discoideum     --------QIISK----RDVHIGNIPIMLRSSNCVLSKKTPEQMAALGECPMDPGGYFIV
D.melanogaster   --------QRIKR----NNLLIGRMPLMLRCSNCALTGKSEFELSKLNECPLDPGGYFVV
E.siliculosus    QEDGEGLEAEEERE--NPKEFLGWVPIMLRSSFCVLVNRTDKELTELGECIYDQGGYFVI
G.gallus         --------QRIIR----NALPIGRMPIMLRSSNCVLTGKTPAEFAKLNECPLDPGGYFIV
G.gorilla        --------QRIIR----NALPIGRMPIMLRSSNCVLTGKTPAEFAKLNECPLDPGGYFIV
H.sapiens        --------QRIIR----NALPIGRMPIMLRSSNCVLTGKTPAEFAKLNECPLDPGGYFIV
I.tridecemlinea  --------QRIIR----NALPIGRMPIMLRSSNCVLTGKTPAEFAKLNECPLDPGGYFIV
M.mulatta        --------QRIIR----NALPIGRMPIMLRSSNCVLTGKTPAEFAKLNECPLDPGGYFIV
M.gallopavo      --------QRIIR----NALPIGRMPIMLRSSNCVLTGKTPAEFAKLNECPLDPGGYFIV
M.musculus       --------QRIIR----NALPIGRMPIMLRSSNCVLTGKTPAEFAKLNECPLDPGGYFIV
O.sativa         --------QYELKR--KPNFIIGYLPIMLRSHACVLNGKDEAELARYGECPLDPGGYFIV
O.latipes        --------QRIIR----NALPIGRMPIMLRSSNCVLTGKTPMEFSKLNECPLDPGGYFIV
P.troglodytes    --------QRIIR----NALPIGRMPIMLRSSNCVLTGKTPAEFAKLNECPLDPGGYFIV
P.falciparum     --------SIITK----NNVEIGRLPVMLRSDICVLNNKSEEELMKLGECPYDPGGYFIV
P.abelii         --------QRIIR----NALPIGRMPIMLRSSNCVLTGKTPAEFAKLNECPLDPGGYFIV
S.cerevisiae     --------NIIMH----KDVEIGRMPIMLRSNKCILYDADESKMAKLNECPLDPGGYFIV
S.pombe          --------QVVRR----RNVPIGRMPVMLRSNKCVLSGKNEMEMAALNECPLDPGGYFIV
S.purpuratus     --------QRVIR----NNLPIGRMPIMLRSSNCVLTGKSPAELAKLNECPLDPGGYFIV
S.scrofa         --------QRIIR----NALPIGRMPIMLRSSNCVLTGKTPAEFAKLNECPLDPGGYFIV
T.rubripes       --------QRIIR----NALPIGRMPIMLRSSNCVLTGKTPMEFAKLNECPLDPGGYFIV
X.tropicalis     --------QRIIR----NALPIGRMPIMLRSSNCVLTGKTPAEFAKLNECPLDPGGYFIV
                                      #######################################


                       4750      4760      4770      4780      4790      4800
                 =========+=========+=========+=========+=========+=========+
A.carolinensis   KGVEKVILIQEQLSKNRIIVEADRKG-T--V-GASVTSST--------------HEKKSR
A.gambiae        RGTEKVILIQEQLSWNKMITE-DYNG-V--I-QCQVTSST--------------HEKKSR
A.thaliana       KGTEKVLLIQEQLSKNRIIIDSDKKG-N--I-NASVTSST--------------EMTKSK
B.distachyon     KGNEKVILIQEQLSKNRIIIDTDNKK-R--V-IASVTSST--------------HEVKSK
C.elegans        KGSEKVILIQEQLSKNRIMVGRNSSK-E--L-QCEVLSST--------------SERKSK
C.jacchus        KGVEKVILIQEQLSKNRIIVEADRKG-A--V-GASVPSST--------------HEKKSR
C.neoformans     KGTEKVILVQEQLSKNRILVMKD-KKDE--V-MAEVTSST--------------HDRVVK
P.tetraurelia    LKKSKIILKDK---------KSL-------------------------------------
D.rerio          KGQEKVILIQEQLSKNRIIVDQDRKG-T--V-GASVTSST--------------HEKKSR
D.discoideum     RGQEKVILNHEQLSKNRIIIEMDSKG-L--P-SASVTSST--------------HERKSR
D.melanogaster   RGQEKVILIQEQLSWNKMLTE-DFNG-V--V-QCQVTSST--------------HEKKSR
E.siliculosus    NGSEKVLIANERMSTNHVYCFKKRQP-SKFTWTSEIRSFVDNSGRPPSSMFLQMYAKGTQ
G.gallus         KGVEKVILIQEQLSKNRIIVEADRKG-T--V-GASVTSST--------------HEKKSR
G.gorilla        KGVEKVILIQEQLSKNRIIVEADRKG-A--V-GASVTSST--------------HEKKSR
H.sapiens        KGVEKVILIQEQLSKNRIIVEADRKG-A--V-GASVTSST--------------HEKKSR
I.tridecemlinea  KGVEKVILIQEQLSKNRIIVEADRKG-A--V-GASVTSST--------------HEKKSR
M.mulatta        KGVEKVILIQEQLSKNRIIVEADRKG-A--V-GASVTSST--------------HEKKSR
M.gallopavo      KGVEKVILIQEQLSKNRIIVEADRKG-T--V-GASVTSST--------------HEKKSR
M.musculus       KGVEKVILIQEQLSKNRIIVEADRKG-A--V-GASVTSST--------------HEKKSR
O.sativa         KGTEKVILIQEQLSKNRIIIDTDSKG-R--V-IASVTSST--------------HEIKSK
O.latipes        KGQEKVILIQEQLSKNRIIVDQDRKG-T--V-GASVTSST--------------HEKKSR
P.troglodytes    KGVEKVILIQEQLSKNRIIVEADRKG-A--V-GASVTSST--------------HEKKSR
P.falciparum     KGTERVLLMQEQLSKNRIIVEMDIKH-N--I-CATITSTT--------------AESKSR
P.abelii         KGVEKVILIQEQLSKNRIIVEADRKG-A--V-GASVTSST--------------HEKKSR
S.cerevisiae     NGTEKVILVQEQLSKNRIIVEADEKKGI--V-QASVTSST--------------HERKSK
S.pombe          KGTEKVILVQEQLSKNRIIVEAEPKKGL--W-QASVTSST--------------HERKSK
S.purpuratus     RGTEKVILIQEQLSKNRMIVEVDRKG-I--V-SCNVTSST--------------HERKSR
S.scrofa         KGVEKVILIQEQLSKNRIIVEADRKG-T--V-GASVTSST--------------HEKKSR
T.rubripes       KGQEKVILIQEQLSKNRIIVDQDRKG-A--V-GASVTSST--------------HEKKSR
X.tropicalis     KGVEKVILIQEQLSKNRIIVEADRKG-T--V-GASVTSST--------------HEKKSR
                 ###########                                                 


                       4810      4820      4830      4840      4850      4860
                 =========+=========+=========+=========+=========+=========+
A.carolinensis   TNITVKQGRFYLKHNTLSEDIPIAIIFKAMGVESDQEIVQMIG-T--EEHVMAAFAPSLE
A.gambiae        TILLTKHGRYYLKHNSMTEEIPVAIILKAMGIASDQEIMQLVG-I--DPETQKRFAPSLL
A.thaliana       TVIQMEKEKIYLFLHRFVKKIPIIIVLKAMGMESDQEIVQMVG-R--DPRFSASLLPSIE
B.distachyon     TVIVMDKEKIYLQLNQFTKPIPIIVVMKAMGIQSDQEIVQMVG-R--DPRYGDLLYLSIQ
C.elegans        TYVTMKKGKYSVRHNQLTDDVPVSIIFKAMGVESDFDIVSTIG-H--EEKYVSAFAQTLE
C.jacchus        TNMAVKQGRFYLRHNTLSEDIPIVIIFKAMGVESDQEIVQMIG-T--EEHVMAAFGPSLE
C.neoformans     TYVVSKANRLYLRHNSFKEFIPIVIALKAMGLTADKEILQLICGS--DERYQEAFGVSLE
P.tetraurelia    ------------------------------------------------------------
D.rerio          TNMIVKQGRFYLKHNTLSEDAPIAIIFKAMGVESDQEIVQMIG-T--EEHVMAAFAPSLE
D.discoideum     TGVTLKNEKLYLKHNTFGEDIPVAIVLKGMGVETDQEMAQLVG-S--DDVFLNAITPSLE
D.melanogaster   TLVLSKHGKYYLKHNSMTDDIPIVVIFKALGVVSDQEIQSLIG-I--DSKSQNRFGASLI
E.siliculosus    HS-KVNGGHIRAQLPYIRTDVPVVLVFRALGYTNDKAILEHIVYDFSDTDMMEKFRPSLE
G.gallus         TNMVVKQGRFYLRHNTLSEDIPIAIIFKAMGVESDQEIVQMIG-T--EEHVMAAFAPSLE
G.gorilla        TNMAVKQGRFYLRHNTLSEDIPIVIIFKAL------------------------------
H.sapiens        TNMAVKQGRFYLRHNTLSEDIPIVIIFKAMGVESDQEIVQMIG-T--EEHVMAAFGPSLE
I.tridecemlinea  TNMAVKQGRFYLRHNTLSEDIPIVIIFKAMGVESDQEIVQMIG-T--EEHVMAAFGPSLE
M.mulatta        TNMAVKQGRFYLRHNTLSEDIPIVIIFKAMGVESDQEIVQMIG-T--EEHVMAAFGPSLE
M.gallopavo      TNLVVKQGRFYLRHNTLSEDIPIAIIFKAMGVESDQEIVQMIG-T--EEHVMAAFAPSLE
M.musculus       TNMAVKQGRFYLRHNTLSEDIPIVIIFKAMGVESDQEIVQMIG-T--EEHVMAAFGPSLE
O.sativa         TVIFMEKEKIYLQLNQFTKPIPIIVVMKAMGMESDQEVAQMVG-R--DPRYGDLLYPSIQ
O.latipes        TNIIVKQGRFYLRHNTLSEDAPIAIIFKGMGVESDQEIVQMIG-T--EEHVMANFAPSLE
P.troglodytes    TNMAVKQGRFYLRHNTLSEDIPIVIIFKAMGVESDQEIVQMIG-T--EEHVMAAFGPSLE
P.falciparum     CAIVYKNNKLYLKHNSFIEDIGVCIILRAMGYESDQEIFQMIG-S--HKNYVNGILLSLY
P.abelii         TNMAVKQGRFYLRHNTLSEDIPIVIIFKAMGVESDQEIVQMIG-T--EEHVMAAFGPSLE
S.cerevisiae     TYVITKNGKIYLKHNSIAEEIPIAIVLKACGILSDLEIMQLVCGN--DSSYQDIFAVNLE
S.pombe          TYVITKNGKLYLKHNSVADDIPIVVVLKAMGLQSDQEIFELVAGA--EASYQDLFAPSIE
S.purpuratus     TVVIVKKGKHYLKHNTFTEDIPIVVIFKAMGMESDQEIVQTIG-T--EESTMVSFASNLE
S.scrofa         TNMAVKQGRFYLRHNTLSEDIPIVIIFKAMGVESDQEIVQMIG-T--EEHVMAAFGPSLE
T.rubripes       TNMIVKQGRFYLRHNTLSEDAPIAIIFKGMGVESDQEIVQMIG-T--EDYVMASFAPSLE
X.tropicalis     TNLVVKQGRFYLKHNTLTEDIPIAIIFKAMGVESDQEIVQMIG-T--EEHVIAAFAPSLE
                                                                             


                       4870      4880      4890      4900      4910      4920
                 =========+=========+=========+=========+=========+=========+
A.carolinensis   ECQKAQIFTQMQALKYLGNKVRRQRMW-G---------G----------PKKTKMEEARE
A.gambiae        EAANHKVFTQQRALEYMGSKLIAKRFT-T-A-------A---------TKYKTTADEARD
A.thaliana       ECVSEGVNTQKQALDYLEAKVKKISYG-T-P--------------------PEKDGRALS
B.distachyon     ECASERIYTQQQALQYMDDKVMFPGPG-N-----------------------VKEGRSKT
C.elegans        ESINAGVYTQQQALAYVTSKVKARKFT-PFGSLPGTSVS---------VLTPPKEHEAVD
C.jacchus        ECQKAQIFTQMQALKYIGNKVRRQRMW-G-G-------G----------PKKTKIEEARE
C.neoformans     EAAKEKTFTRRQALEWIGARVSPNQAKDD-S-------GS--------NQKLTPSDIAQQ
P.tetraurelia    ------STLYKKSQEFFFSIKKNFFSIKKKK-NFKNSN------------EKMPYIISPR
D.rerio          ECQKAQIFTQTQALRYIGNKVRRQRMW-G-G------------------PKKTKMEEARE
D.discoideum     ECQKCGVHTAAQALDYLGSRIKVFRRP-Y-G-------V---------QNKKTKSEEARD
D.melanogaster   DAYNLKVFTQQRALEYMGSKLVVKRFQ-S-A-------T---------T--KTPSEEARE
E.siliculosus    EADV--IQNQVVAQDFIGKRGSAVNV--G---------------------RNERINYAKG
G.gallus         ECQKAQIFTQMQALKYIGNKVRRQRMW-G-G------------------PKKTKMEEARE
G.gorilla        --------------KYIGNKVRRQRMW-G-G-------G----------PKKTKIEEARE
H.sapiens        ECQKAQIFTQMQALKYIGNKVRRQRMW-G-G-------G----------PKKTKIEEARE
I.tridecemlinea  ECQKAQIFTQMQALKYIGNKVRRQRMW-G-G-------G----------PKKTKIEEARE
M.mulatta        ECQKAQIFTQMQALKYIGNKVRRQRMW-G-G-------G----------PKKTKIEEARE
M.gallopavo      ECQKAQIFTQMQALKYIGNKVRRQRMW-G-G------------------PKKTKMEEARE
M.musculus       ECQKAQIFTQMQALKYIGNKVRRQRMW-G-G-------G----------PKKTKIEEARE
O.sativa         ECAFERIYTQKQALQYMDDKVMYPGAG-N-----------------------QKEGRSKS
O.latipes        ECQKAQIFTQTQ------------------------------------------------
P.troglodytes    ECQKAQIFTQMQALKYIGNKVRRQRMW-G-G-------G----------PKKTKIEEARE
P.falciparum     ELYNENIKTNLDALLYIGKKIRPRLLA-K-G-------FFSSMKEKQVKNEKDIIEEGLD
P.abelii         ECQKAQIFTQMQALKYIGNKVRRQRMW-G-G-------G----------PKKTKIEEARE
S.cerevisiae     ESSKLDIYTQQQALEYIGAKVKTMRRQ-----------------------KLTILQEGIE
S.pombe          ECAKLNIYTAQQALEYIGARVKVNRRAG----------A----------NRLPPHEEALE
S.purpuratus     ECHKIQVYTQLQALKYMGMKVRQRRMW-G-G-------G----------SKKSKVDEARE
S.scrofa         ECQKAQIFTQMQALKYIGNKVRRQRMW-G-G-------G----------PKKTKIEEARE
T.rubripes       ECQKAQIFTQTQALRYLGNKVRRQRMW-G-G------------------PKKTKMEEARE
X.tropicalis     ECQKAQIFTQMQALKFIGNKVRRQRMW-G-SS------G----------PKKSKIDEARE
                                                                             


                       4930      4940      4950      4960      4970      4980
                 =========+=========+=========+=========+=========+=========+
A.carolinensis   LLATTILAHV--P-VKEFNFRSKCIYTAVMVRRVILAQGDN-KVDDRDYYGNKRLELAGQ
A.gambiae        LLATTILAHV--P-VPSFNFQVKAIYVALMIRRVMAAELDRSAVDDRDYYGNKRLELAGS
A.thaliana       ILRDLFLAHV--P-VPDNNFRQKCFYVGVMLRRMIEAMLNKDAMDDKDYVGNKRLELSGQ
B.distachyon     LLRDVFVAHV--P-VENGNFREKCIYTAVMLRRMLDAILNSDTFDDKDYVGNKRLELSGQ
C.elegans        FLSNSMITHI--A-CPDGNFKMKAIYLGLMTRRLIQTELGENGLDDRDFYGNKRLELAGS
C.jacchus        LLASTILTHV--P-VKEFNFRAKCIYTAVMVRRVILAQGDN-KVDDRDYYGNKRLELAGQ
C.neoformans     ALAAMVLGHV--P-VRNMNFRPKCIYLATMSRRVLMAMIDDHMVDDRDYVGNKRLELAGQ
P.tetraurelia    R--TENQKMYNSILTHNNHDFVFVKFVFRETRTPEVGDKFSSRHGQKGVCGIVNFYEDFP
D.rerio          LLASTILTHV--P-VKEFNFRAKCIYLAVMVRRVILAQGDN-KVDDRDYYGNKRLELAGQ
D.discoideum     ILAGVVLNHV--P-VRRYNFRLKVIYLSLMIRRIIMASKDKSCLDDKDYYGNKRIELSGQ
D.melanogaster   LLLTTILAHV--P-VDNFNLQMKAIYVSMMVRRVMAAELDKTLFDDRDYYGNKRLELAGS
E.siliculosus    LLQREFLPHV--G-IGAGTEAKKVFFLGYMVHKLLMCSLGRLEEDDRDHYGKKRLDLAGA
G.gallus         LLASTILTHV--L-VKEFNFRAKCIYTAVMVRRVILAQGEN-KVDDRDYYGNKRLELAGQ
G.gorilla        LLASTILTHV--P-VKEFNFRAKCIYTAVMVRRVILAQGDN-KVDDRDYYGNKRLELAGQ
H.sapiens        LLASTILTHV--P-VKEFNFRAKCIYTAVMVRRVILAQGDN-KVDDRDYYGNKRLELAGQ
I.tridecemlinea  LLASTILTHV--P-VKEFNFRAKCIYTAVMVRRVILAQGDN-KVDDRDYYGNKRLELAGQ
M.mulatta        LLASTILTHV--P-VKEFNFRAKCIYTAVMVRRVILAQGDN-KVDDRDYYGNKRLELAGQ
M.gallopavo      LLASTILTHV--L-VKEFNFRAKCIYTAVMVRRVILAQGEN-KVDDRDYYGNKRLELAGQ
M.musculus       LLASTILTHV--P-VKEFNFRAKCIYTAVMVRRVILAQGDN-KVDDRDYYGNKRLELAGQ
O.sativa         ILRDVFVAHV--P-VESGNFRPKCIYTAVMLRRMMDAILNADTFDDKDYVGNKRLELSGQ
O.latipes        --------------VKEFNFRAKCIYLAVMVRRVILAQGEN-KVDDRDYYGNKRLELAGQ
P.troglodytes    LLASTILTHV--P-VKEFNFRAKCIYTAVMVRRVILAQGDN-KVDDRDYYGNKRLELAGQ
P.falciparum     FLSRVLLSHI--QQKSKYDFRNKARCICLMIRRVLDSANNKNEIDDKDYYGNKRLELAGQ
P.abelii         LLASTILTHV--P-VKEFNFRAKCIYTAVMVRRVILAQGDN-KVDDRDYYGNKRLELAGQ
S.cerevisiae     AIATTVIAHL--T-VEALDFREKALYIAMMTRRVVMAMYNPKMIDDRDYVGNKRLELAGQ
S.pombe          VLAAVVLAHI--N-VFNLEFRPKAVYIGIMARRVLMAMVDPLQVDDRDYVGNKRLELAGQ
S.purpuratus     LLATTILAHI--P-VVQFNFRAKSIYLALMVRRVIEAQGDAVKVDDRDYYGNKRLELAGQ
S.scrofa         LLASTILTHV--P-VKEFNFRAKCIYTAVMVRRVILAQGDN-KVDDRDYYGNKRLELAGQ
T.rubripes       LLASLILTHV--P-VKEFNFRAKCIYLAVMVRRVILAQGGN-KVDDRDYYGNKRLELAGQ
X.tropicalis     LLASTILTHV--P-VKEFHFRTKCIYIAVMVRRVILAQGAN-KVDDRDYYGNKRLELAGQ
                               ########################### ##################


                       4990      5000      5010      5020      5030      5040
                 =========+=========+=========+=========+=========+=========+
A.carolinensis   LLSL-LFEDLFKKFNSELKKIADQVIPK-Q-RAAQFDVVKHMRQ---------------D
A.gambiae        LLSL-MFEDLFKRFNWELKMIADKNIPK-I-KAAQFDVGKHMRE---------------A
A.thaliana       LISL-LFEDLFKTMLSEAIKNVDHILNKPI-RASRFDFSQCLNK-D-------------S
B.distachyon     LVSL-LFEDLFKTMNSFAVDRMNKNS-DMA-RSSPLDFSQLIMQ-Q-------------D
C.elegans        LLSL-LFEDVFKRFNSELKRIADNALMK-T-MAAPLDIVKHMRQ---------------D
C.jacchus        LLSL-LFEDLFKKFNSEMKKIADQVIPK-Q-RAAQFDVVKHMRQ---------------D
C.neoformans     LLSL-LFEDSFKTFNSELKKRMDKILEK-PNRAGPFDAGTLIRQGG-------------D
P.tetraurelia    FSNEGSVPDIIMNPHGFPSRMTIGKIVELLGSKLSCLSGKFFEGTPFKKF-----NRIVF
D.rerio          LLSL-LFEDLFKKFNSELKKIADQIIPK-Q-RAAQFDVVKHMRQ---------------D
D.discoideum     LISL-LFEDCFKKFQSELKKSVDQAIAK-ANRAENLDLPKLIRT---------------D
D.melanogaster   LLSM-MFEDLFKRMNWELKTIADKNIPK-V-KAAQFDVVKHMRA---------------A
E.siliculosus    LLAG-LFRQLFRKLTQNVRKYLQLCLDK-G---TQFVVGTAIKS---------------Q
G.gallus         LLSL-LFEDLFKKFNSELKKIADQVIPK-Q-RAAQFDVVKHMRQ---------------D
G.gorilla        LINDILFQDCDWVRNSLMSFIS--------------------------------------
H.sapiens        LLSL-LFEDLFKKFNSEMKKIADQVIPK-Q-RAAQFDVVKHMRQ---------------D
I.tridecemlinea  LLSL-LFEDLFKKFNSEMKKIADQVIPK-Q-RAAQFDVVKHMRQ---------------D
M.mulatta        LLSL-LFEDLFKKFNSEMKKIADQVIPK-Q-RAAQFDVVKHMRQ---------------D
M.gallopavo      LLSL-LFEDLFKKFNSELKKIADQVIPK-Q-RAAQFDVVKHMRQ---------------D
M.musculus       LLSL-LFEDLFKKFNSEMKKIADQVIPK-Q-RAAQFDVVKHMRQ---------------D
O.sativa         LISL-LFEDLFKTMNSNAVELMNKTS-EKI-HSSPLDLS-LHIK-E-------------N
O.latipes        LLSL-LFEDLFKKFNSELKKIADQIIPK-Q-RAAQFDVVKHMRQ---------------D
P.troglodytes    LLSL-LFEDLFKKFNSEMKKIADQVIPK-Q-RAAQFDVVKHMRQ---------------D
P.falciparum     LISL-LFEDLYKRFYFTLKKQIDQTLSK-YMQSNYNSKLRSTGNNMNDNYPDVFRNLPKD
P.abelii         LLSL-LFEDLFKKFNSEMKKIADQVIPK-Q-RAAQFDVVKHMRQ---------------D
S.cerevisiae     LISL-LFEDLFKKFNNDFKLSIDKVLKK-PNRAMEYDALLSINVHS-------------N
S.pombe          LLAL-LFEDLFKKFNSDLKLNIDKVLKK-PHRTQEFDAYNQLTVHS-------------D
S.purpuratus     LIAL-LFEDLFKKFNSELKKYADQNIPR-P-RAAQFDIVKYMRQ---------------D
S.scrofa         LLSL-LFEDLFKKFNSEMKKIADQVIPK-Q-RAAQFDVVKHMRQ---------------D
T.rubripes       LLSL-LFEDLFKKFNSELKKIADQIIPK-Q-RAAQFDVVKHMRQ---------------D
X.tropicalis     LLSL-LFEDLFKKFNSEMKKIADQVIPK-Q-RAAQFDVVKHMRQ---------------D
                 #### ##########                                             


                       5050      5060      5070      5080      5090      5100
                 =========+=========+=========+=========+=========+=========+
A.carolinensis   --QITNGMVNAISTGNWSLKRFKMDRQGVTQVLSRLSYISALGMMT--------------
A.gambiae        --LITAGLETAISTGNWTIKRFKMERAGVTQVLSRLSYISALGMMT--------------
A.thaliana       RYSISLGLERTLSTGNFDIKRFRMHRKGMTQVLTRLSFIGSMGFIT--------------
B.distachyon     --LITTGLERAISTGNWDIKRFKMHRKGVSQVLSRLSYMASLGYMT--------------
C.elegans        --MITNTIVNAMSTGNWIIKRFRMERLGVTQVLSRLSYISALGMMT--------------
C.jacchus        --QITNGMVNAISTGNWSLKRFKMDRQGVTQVLSRLSYISALGMMT--------------
C.neoformans     --PITQAFVRSISTGNWSLKRFHVERAGVTHVLSRLSFIAALGMMT--------------
P.tetraurelia    EKKILKLGYNKNCKDIFFSGTSGQPLYAFVFSGPVFYQKLKHMVADKIYSRSRGIKSKLT
D.rerio          --QITNGMVNAISTGNWSLKRFKMDRQGVTQVLSRLSFISALGMMT--------------
D.discoideum     --TITNGFTHAISSGQWNLKRFRMERSGVSQVLSRLSYISCMGMMTRIQSQFEKTRKVAG
D.melanogaster   --QITAGLESAISSGNWTIKRFKMERAGVTQVLSRLSYISALGMMT--------------
E.siliculosus    --FITDGLKYSLATGNWGDKK-TATKAGVSQVLNRLTYASALSHLR--------------
G.gallus         --QITNGMVNAISTGNWSLKRFKMDRQGVTQVLSRLSYISALGMMT--------------
G.gorilla        ------------LQGNWSLKRFKMDRQGVTQVLSRLSYISALGMMT--------------
H.sapiens        --QITNGMVNAISTGNWSLKRFKMDRQGVTQVLSRLSYISALGMMT--------------
I.tridecemlinea  --QITNGMVNAISTGNWSLKRFKMDRQGVTQVLSRLSYISALGMMT--------------
M.mulatta        --QITNGMVNAISTGNWSLKRFKMDRQGVTQVLSRLSYISALGMMT--------------
M.gallopavo      --QITNGMVNAISTGNWSLKRFKMDRQGVTQVLSRLSYISALGMMT--------------
M.musculus       --QITNGMVNAISTGNWSLKRFKMDRQGVTQVLSRLSYISALGMMT--------------
O.sativa         --IITHGLERAISTGNWDIKRFRMHRKGVSQVLSRLSYMASLGYMT--------------
O.latipes        --QITNGMVNAISTGNWSLKRFKMDRQGVTQVLSRLSYISALGMMT--------------
P.troglodytes    --QITNGMVNAISTGNWSLKRFKMDRQGVTQVLSRLSYISALGMMT--------------
P.falciparum     --IITRGMQTAISTGNWNIKRFKMEKSGVSQVLSRLSFIACIGMMT--------------
P.abelii         --QITNGMVNAISTGNWSLKRFKMDRQGVTQVLSRLSYISALGMMT--------------
S.cerevisiae     --NITSGLNRAISTGNWSLKRFKMERAGVTHVLSRLSYISALGMMT--------------
S.pombe          --HITQGMVRALSTGNWSLKRFKMERAGVTHVLSRLSYISALGMMT--------------
S.purpuratus     --QITNGLITAISTGNWSVKRFKMERAGVTQVLSRLSYISCLGMMT--------------
S.scrofa         --QITNGMVNAISTGNWSLKRFKMDRQGVTQVLSRLSYISALGMMT--------------
T.rubripes       --QITNGMVNAISTGNWSLKRFKMDRQGVTQVLSRLSFISALGMMT--------------
X.tropicalis     --QITNGMVNAISSGNWSLKRFKMDRQGVTQVLSRLSYIAALGMMT--------------
                                       ########################              


                       5110      5120      5130      5140      5150      5160
                 =========+=========+=========+=========+=========+=========+
A.carolinensis   ------------------------------------------------------------
A.gambiae        ------------------------------------------------------------
A.thaliana       ------------------------------------------------------------
B.distachyon     ------------------------------------------------------------
C.elegans        ------------------------------------------------------------
C.jacchus        ------------------------------------------------------------
C.neoformans     ------------------------------------------------------------
P.tetraurelia    RQPVEGRNKGGGLRFGEMERDCLVSYGASELTLERLMISSDIYLGSFDTKTGTITH----
D.rerio          ------------------------------------------------------------
D.discoideum     PRSLQPSQWGMLCPSDTPEGEACLHPDTIITMSNGQQKPIRQLKDGDSIITLDPITMEAH
D.melanogaster   ------------------------------------------------------------
E.siliculosus    ------------------------------------------------------------
G.gallus         ------------------------------------------------------------
G.gorilla        ------------------------------------------------------------
H.sapiens        ------------------------------------------------------------
I.tridecemlinea  ------------------------------------------------------------
M.mulatta        ------------------------------------------------------------
M.gallopavo      ------------------------------------------------------------
M.musculus       ------------------------------------------------------------
O.sativa         ------------------------------------------------------------
O.latipes        ------------------------------------------------------------
P.troglodytes    ------------------------------------------------------------
P.falciparum     ------------------------------------------------------------
P.abelii         ------------------------------------------------------------
S.cerevisiae     ------------------------------------------------------------
S.pombe          ------------------------------------------------------------
S.purpuratus     ------------------------------------------------------------
S.scrofa         ------------------------------------------------------------
T.rubripes       ------------------------------------------------------------
X.tropicalis     ------------------------------------------------------------
                                                                             


                       5170      5180      5190      5200      5210      5220
                 =========+=========+=========+=========+=========+=========+
A.carolinensis   ------------------------------------------------------------
A.gambiae        ------------------------------------------------------------
A.thaliana       ------------------------------------------------------------
B.distachyon     ------------------------------------------------------------
C.elegans        ------------------------------------------------------------
C.jacchus        ------------------------------------------------------------
C.neoformans     ------------------------------------------------------------
P.tetraurelia    ------VTNSNKHSITLIKFPYACKLLFQELYSMNIIPRIYF--GFDNYNKFT-V-----
D.rerio          ------------------------------------------------------------
D.discoideum     STRIYSHFIKSSSQYGKQLLKITTITGKEIICTNDHRFLTSNGNWKQSKDLLLNDKLFLI
D.melanogaster   ------------------------------------------------------------
E.siliculosus    ------------------------------------------------------------
G.gallus         ------------------------------------------------------------
G.gorilla        ------------------------------------------------------------
H.sapiens        ------------------------------------------------------------
I.tridecemlinea  ------------------------------------------------------------
M.mulatta        ------------------------------------------------------------
M.gallopavo      ------------------------------------------------------------
M.musculus       ------------------------------------------------------------
O.sativa         ------------------------------------------------------------
O.latipes        ------------------------------------------------------------
P.troglodytes    ------------------------------------------------------------
P.falciparum     ------------------------------------------------------------
P.abelii         ------------------------------------------------------------
S.cerevisiae     ------------------------------------------------------------
S.pombe          ------------------------------------------------------------
S.purpuratus     ------------------------------------------------------------
S.scrofa         ------------------------------------------------------------
T.rubripes       ------------------------------------------------------------
X.tropicalis     ------------------------------------------------------------
                                                                             


                       5230      5240      5250      5260      5270      5280
                 =========+=========+=========+=========+=========+=========+
A.carolinensis   ------------------------------------------------------------
A.gambiae        ------------------------------------------------------------
A.thaliana       ------------------------------------------------------------
B.distachyon     ------------------------------------------------------------
C.elegans        ------------------------------------------------------------
C.jacchus        ------------------------------------------------------------
C.neoformans     ------------------------------------------------------------
P.tetraurelia    ------------------------------------------------------------
D.rerio          ------------------------------------------------------------
D.discoideum     SSSNQLEFNNNNNENNNENNNDIIEILNENQLINQGVVPIKIIQELKSIELLPLLNNNEK
D.melanogaster   ------------------------------------------------------------
E.siliculosus    ------------------------------------------------------------
G.gallus         ------------------------------------------------------------
G.gorilla        ------------------------------------------------------------
H.sapiens        ------------------------------------------------------------
I.tridecemlinea  ------------------------------------------------------------
M.mulatta        ------------------------------------------------------------
M.gallopavo      ------------------------------------------------------------
M.musculus       ------------------------------------------------------------
O.sativa         ------------------------------------------------------------
O.latipes        ------------------------------------------------------------
P.troglodytes    ------------------------------------------------------------
P.falciparum     ------------------------------------------------------------
P.abelii         ------------------------------------------------------------
S.cerevisiae     ------------------------------------------------------------
S.pombe          ------------------------------------------------------------
S.purpuratus     ------------------------------------------------------------
S.scrofa         ------------------------------------------------------------
T.rubripes       ------------------------------------------------------------
X.tropicalis     ------------------------------------------------------------
                                                                             


                       5290      5300      5310      5320      5330      5340
                 =========+=========+=========+=========+=========+=========+
A.carolinensis   ------------------------------------------------------------
A.gambiae        ------------------------------------------------------------
A.thaliana       ------------------------------------------------------------
B.distachyon     ------------------------------------------------------------
C.elegans        ------------------------------------------------------------
C.jacchus        ------------------------------------------------------------
C.neoformans     ------------------------------------------------------------
P.tetraurelia    ------------------------------------------------------------
D.rerio          ------------------------------------------------------------
D.discoideum     LITISRIIGSIDKIGSNKQNEPIIQYQFNLISDFDQFLKDLQYLGFINPIYKLNEEQQQQ
D.melanogaster   ------------------------------------------------------------
E.siliculosus    ------------------------------------------------------------
G.gallus         ------------------------------------------------------------
G.gorilla        ------------------------------------------------------------
H.sapiens        ------------------------------------------------------------
I.tridecemlinea  ------------------------------------------------------------
M.mulatta        ------------------------------------------------------------
M.gallopavo      ------------------------------------------------------------
M.musculus       ------------------------------------------------------------
O.sativa         ------------------------------------------------------------
O.latipes        ------------------------------------------------------------
P.troglodytes    ------------------------------------------------------------
P.falciparum     ------------------------------------------------------------
P.abelii         ------------------------------------------------------------
S.cerevisiae     ------------------------------------------------------------
S.pombe          ------------------------------------------------------------
S.purpuratus     ------------------------------------------------------------
S.scrofa         ------------------------------------------------------------
T.rubripes       ------------------------------------------------------------
X.tropicalis     ------------------------------------------------------------
                                                                             


                       5350      5360      5370      5380      5390      5400
                 =========+=========+=========+=========+=========+=========+
A.carolinensis   ------------------------------------------------------------
A.gambiae        ------------------------------------------------------------
A.thaliana       ------------------------------------------------------------
B.distachyon     ------------------------------------------------------------
C.elegans        ------------------------------------------------------------
C.jacchus        ------------------------------------------------------------
C.neoformans     ------------------------------------------------------------
P.tetraurelia    ------------------------------------------------------------
D.rerio          ------------------------------------------------------------
D.discoideum     QQQKIDHQQQQQQQVEQQQKSIIIDFIGSSFGYFIQSLLNEKNWIEKSNNQFVKKEFLSS
D.melanogaster   ------------------------------------------------------------
E.siliculosus    ------------------------------------------------------------
G.gallus         ------------------------------------------------------------
G.gorilla        ------------------------------------------------------------
H.sapiens        ------------------------------------------------------------
I.tridecemlinea  ------------------------------------------------------------
M.mulatta        ------------------------------------------------------------
M.gallopavo      ------------------------------------------------------------
M.musculus       ------------------------------------------------------------
O.sativa         ------------------------------------------------------------
O.latipes        ------------------------------------------------------------
P.troglodytes    ------------------------------------------------------------
P.falciparum     ------------------------------------------------------------
P.abelii         ------------------------------------------------------------
S.cerevisiae     ------------------------------------------------------------
S.pombe          ------------------------------------------------------------
S.purpuratus     ------------------------------------------------------------
S.scrofa         ------------------------------------------------------------
T.rubripes       ------------------------------------------------------------
X.tropicalis     ------------------------------------------------------------
                                                                             


                       5410      5420      5430      5440      5450      5460
                 =========+=========+=========+=========+=========+=========+
A.carolinensis   ------------------------------------------------------------
A.gambiae        ------------------------------------------------------------
A.thaliana       ------------------------------------------------------------
B.distachyon     ------------------------------------------------------------
C.elegans        ------------------------------------------------------------
C.jacchus        ------------------------------------------------------------
C.neoformans     ------------------------------------------------------------
P.tetraurelia    ------------------------------------------------------------
D.rerio          ------------------------------------------------------------
D.discoideum     FISNVNRIQFNIIEEINQQNNDSNFKILLNYKQQKQQQNEKEERAVVDHDNEIFNIKSLQ
D.melanogaster   ------------------------------------------------------------
E.siliculosus    ------------------------------------------------------------
G.gallus         ------------------------------------------------------------
G.gorilla        ------------------------------------------------------------
H.sapiens        ------------------------------------------------------------
I.tridecemlinea  ------------------------------------------------------------
M.mulatta        ------------------------------------------------------------
M.gallopavo      ------------------------------------------------------------
M.musculus       ------------------------------------------------------------
O.sativa         ------------------------------------------------------------
O.latipes        ------------------------------------------------------------
P.troglodytes    ------------------------------------------------------------
P.falciparum     ------------------------------------------------------------
P.abelii         ------------------------------------------------------------
S.cerevisiae     ------------------------------------------------------------
S.pombe          ------------------------------------------------------------
S.purpuratus     ------------------------------------------------------------
S.scrofa         ------------------------------------------------------------
T.rubripes       ------------------------------------------------------------
X.tropicalis     ------------------------------------------------------------
                                                                             


                       5470      5480      5490      5500      5510      5520
                 =========+=========+=========+=========+=========+=========+
A.carolinensis   ------------------------------------------------------------
A.gambiae        ------------------------------------------------------------
A.thaliana       ------------------------------------------------------------
B.distachyon     ------------------------------------------------------------
C.elegans        ------------------------------------------------------------
C.jacchus        ------------------------------------------------------------
C.neoformans     ------------------------------------------------------------
P.tetraurelia    ------------------------------------------------------------
D.rerio          ------------------------------------------------------------
D.discoideum     ILLNQFNVISSIDFEESNLIIINSSLKKFIDLINIKFNQKLNNQIIQIREYLNYINYNNN
D.melanogaster   ------------------------------------------------------------
E.siliculosus    ------------------------------------------------------------
G.gallus         ------------------------------------------------------------
G.gorilla        ------------------------------------------------------------
H.sapiens        ------------------------------------------------------------
I.tridecemlinea  ------------------------------------------------------------
M.mulatta        ------------------------------------------------------------
M.gallopavo      ------------------------------------------------------------
M.musculus       ------------------------------------------------------------
O.sativa         ------------------------------------------------------------
O.latipes        ------------------------------------------------------------
P.troglodytes    ------------------------------------------------------------
P.falciparum     ------------------------------------------------------------
P.abelii         ------------------------------------------------------------
S.cerevisiae     ------------------------------------------------------------
S.pombe          ------------------------------------------------------------
S.purpuratus     ------------------------------------------------------------
S.scrofa         ------------------------------------------------------------
T.rubripes       ------------------------------------------------------------
X.tropicalis     ------------------------------------------------------------
                                                                             


                       5530      5540      5550      5560      5570      5580
                 =========+=========+=========+=========+=========+=========+
A.carolinensis   -----------------------------------RISSQFEKTRKVSGPRSLQPSQWGM
A.gambiae        -----------------------------------RVNSQFEKTRKVSGPRSLQPSQWGM
A.thaliana       -----------------------------------KISPQFEKSRKVSGPRSLQPSQWGM
B.distachyon     -----------------------------------RITPQFEKTRKTSGPRALQPSQWGM
C.elegans        -----------------------------------RINSTFEKTRKVSGPRSLQPSQWGM
C.jacchus        -----------------------------------RISSQFEKTRKVSGPRSLQPSQWGM
C.neoformans     -----------------------------------RISSQFEKTRKVSGPRALQPSQWGM
P.tetraurelia    ------------------------------------------------------------
D.rerio          -----------------------------------RISSQFEKTRKVSGPRSLQPSQWGM
D.discoideum     NNNNEEINIKKKDFGYFKKLKIKRNSFEIEIEKIEQLNYQDCPEISDFTTESDYHS---M
D.melanogaster   -----------------------------------RVNSQFEKTRKVSGPRSLQPSQWGM
E.siliculosus    -----------------------------------RLNTPLGREGKQAKPRQLHNTHWGF
G.gallus         -----------------------------------RISSQFEKTRKVSGPRSLQPSQWGM
G.gorilla        -----------------------------------RISSQFEKTRKVSGPRSLQPSQWGM
H.sapiens        -----------------------------------RISSQFEKTRKVSGPRSLQPSQWGM
I.tridecemlinea  -----------------------------------RISSQFEKTRKVSGPRSLQPSQWGM
M.mulatta        -----------------------------------RISSQFEKTRKVSGPRSLQPSQWGM
M.gallopavo      -----------------------------------RISSQFEKTRKVSGPRSLQPSQWGM
M.musculus       -----------------------------------RISSQFEKTRKVSGPRSLQPSQWGM
O.sativa         -----------------------------------RITPQFEKTRKTSGPRALQPSQWGM
O.latipes        -----------------------------------RISSQFEKTRKVSGPRSLQPSQWGM
P.troglodytes    -----------------------------------RISSQFEKTRKVSGPRSLQPSQWGM
P.falciparum     -----------------------------------RLNSQFEKGRKVSGPRALQPSQWGV
P.abelii         -----------------------------------RISSQFEKTRKVSGPRSLQPSQWGM
S.cerevisiae     -----------------------------------RISSQFEKSRKVSGPRALQPSQFGM
S.pombe          -----------------------------------RITSQFEKTRKVSGPRSLQASQFGM
S.purpuratus     -----------------------------------RISSQFEKTRKVSGPRSLQPSQWGM
S.scrofa         -----------------------------------RISSQFEKTRKVSGPRSLQPSQWGM
T.rubripes       -----------------------------------RISSQFEKTRKVSGPRSLQPSQWGM
X.tropicalis     -----------------------------------RISSQFEKTRKVSGPRSLQPSQWGM
                                                                             


                       5590      5600      5610      5620      5630      5640
                 =========+=========+=========+=========+=========+=========+
A.carolinensis   LCPSDTPEGEACGLVKNLALMTHITTDMEDG-PIIKLAGNLGVEDVNLLCGEELSYPNVF
A.gambiae        LCPSDTPEGEACGLVKNLALMTHITTEVDEE-PVIRLAYNAGVEDIRLLGGETINNPKVF
A.thaliana       LCPCDTPEGESCGLVKNLALMTHVTTDEEEG-PLVAMCYKLGVTDLEVLSAEELHTPDSF
B.distachyon     LCPCDTPEGEACGLTKNLALLTHVTTDQEEG-PLMNLCYSLGVEKLSLLSGEEIHAPGSF
C.elegans        LCPSDTPEGEACGLVKNLALISHITTDSDEK-PVLRLLLNSGVEDLHNVHFSHVNKPENT
C.jacchus        LCPSDTPEGEACGLVKNLALMTHITTDMEDG-PIVKLASNLGVEDVNLLCGEELSYPNVF
C.neoformans     LCPSDTPEGEACGLVKNLALMTHITTDVPEA-PLVKLAFMLGVEDISLVTGNELYRPGVL
P.tetraurelia    ------------------------------------------------------------
D.rerio          LCPSDTPEGEACGLVKNLALMTHITTDMEDG-PIIKLAFNLGVEDVNLLCGEELSYPTVF
D.discoideum     I--SNGFVSHNCGLVKNFALMSHVTTDDSEG-PLLRLAYNLGVQDILLVTGEELNSRNAY
D.melanogaster   LCPSDTPEGEACGLVKNLALMTHITTEVEER-PVMIVAFNAGVEDIREVSGNPINNPNVF
E.siliculosus    ICPAETPEGQAVGLVKNLALMAYISVGCSLS-PILEFLEEWAMENLDEIAPHMIAQTNCT
G.gallus         LCPSDTPEGEACGLVKNLALMTHITTDMEDG-PIIKLASNLGVEDVNLLCGEELSYPNVF
G.gorilla        LCPSDTPEGEACGLVKNLALMTHITTDMEDG-PIVKLASNLGVEDVNLLCGEELSYPNVF
H.sapiens        LCPSDTPEGEACGLVKNLALMTHITTDMEDG-PIVKLASNLGVEDVNLLCGEELSYPNVF
I.tridecemlinea  LCPSDTPEGEACGLVKNLALMTHITTDMEDG-PIVKLASNLGVEDVNLLCGEELSYPNVF
M.mulatta        LCPSDTPEGEACGLVKNLALMTHITTDMEDG-PIVKLASNLGVEDVNLLCGEELSYPNVF
M.gallopavo      LCPSDTPEGEACGLVKNLALMTHITTDMEDG-PIIKLASNLGVEDVNLLCGEELSYPNVF
M.musculus       LCPSDTPEGEACGLVKNLALMTHITTDMEDG-PIIKLAGNLGVEDVNLLCGEELSYPNVF
O.sativa         LCPCDTPEGEACGLTKNLALLTHVTTDQEEG-PLMNLCYSLGVEDLSLLSGEEIHASGSF
O.latipes        LCPSDTPEGEACGLVKNLALMTHITTDMEDG-PIIKLALNLGVEDVNLLCGEELSYPSVF
P.troglodytes    LCPSDTPEGEACGLVKNLALMTHITTDMEDG-PIVKLASNLGVEDVNLLCGEELSYPNVF
P.falciparum     LCPCDTPEGESCGLVKNLALMTHVTNDNENNENLIEILYTLGVEDSDSLTGEEIYKEGVF
P.abelii         LCPSDTPEGEACGLVKNLALMTHITTDMEDG-PIVKLASNLGVEDVNLLCGEELSYPNVF
S.cerevisiae     LCTADTPEGEACGLVKNLALMTHITTDDEEE-PIKKLCYVLGVEDITLIDSASLHLN--Y
S.pombe          LCTSDTPEGEACGLVKNLALMTHITTDEEEE-PIIKLAYAFGIEDIHVISGRELHSHGTY
S.purpuratus     LCPSDTPEGEGCGLIKNLALMTHITTDMEEA-PIVKLAFNLGVEPVHLMSGEELSMKDVY
S.scrofa         LCPSDTPEGEACGLVKNLALMTHITTDMEDG-PIVKLASNLGVEDVNLLCGEELSYPNVF
T.rubripes       LCPSDTPEGEACGLVKNLALMTHITTDMEDG-PIIKLAFNLGVEDVNLLCGEELSYPSVF
X.tropicalis     LCPSDTPEGEACGLVKNLALMTHITTDMEDG-PIIKLAANLGVEDVNLLCGEELSYANVF
                                                                             


                       5650      5660      5670      5680      5690      5700
                 =========+=========+=========+=========+=========+=========+
A.carolinensis   LVFLNGNILGVIRDHQKLVYTFRLMRRAGYINEFVSISTNLTDRCVYISSDGGRLCRPYI
A.gambiae        MVFINGNILGVTIAYRRLVEVFRMMRRRGLIGAFVSIHTSFTQRCVYIHTDGGRLCRPYI
A.thaliana       LVILNGLILGKHSRPQYFANSLRRLRRAGKIGEFVSVFTNEKQHCVYVASDVGRVCRPLV
B.distachyon     LVMFNGLILGKHRQPQRFAKSMRTFRQSGKIGEFVSIFVNEKQHCIHIASDGGRVCRPLI
C.elegans        LIFLNGVLIGTAVDPERVVKAVRDLRRSGLLSEFVSVSRSLTNRSVFISSDGGRLCRPYI
C.jacchus        LVFLNGNILGVIRDHKKLVNTFRLMRRAGYINEFVSISTNLTDRCVYISSDGGRLCRPYI
C.neoformans     MVQVNGTLIGVTNMAKRFVRQFRKLRRAGRMSEFVSIFINHHHKIIYIASDGGRICRPMI
P.tetraurelia    ------------------------------------------------------------
D.rerio          LVFLNGNILGVIRDHQKLVYTFRLMRRAGFINEFVSISTNLTDRCVYISSDGGRLCRPYI
D.discoideum     LVLLNGQIIGIHNSPDYFVTTLRKMRRAGRIREFVSICKNKAQQTISVACDGGRLCRPVI
D.melanogaster   LVFINGNVLGLTLNHKHLVRNLRYMRRKGRMGSYVSVHTSYTQRCIYIHTDGGRLCRPYV
E.siliculosus    KVFVNGNWVGVHRDPNRLVQTLVHQRRALDIDVEVSVVRDIKGRELRLYTDAGRVCRPLF
G.gallus         LVFLNGNILGVIRDHQKLVNTFRIMRRAGYINEFVSISTNLSDRCVYISSDGGRLCRPYI
G.gorilla        LVFLNGNILGVIRDHKKLVNTFRLMRRAGYINEFVSISTNLTDRCVYISSDGGRLCRPYI
H.sapiens        LVFLNGNILGVIRDHKKLVNTFRLMRRAGYINEFVSISTNLTDRCVYISSDGGRLCRPYI
I.tridecemlinea  LVFLNGNILGVIRDHKKLVNTFRLMRRAGYINEFVSISTNLTDRCVYISSDGGRLCRPYI
M.mulatta        LVFLNGNILGVIRDHKKLVNTFRLMRRAGYINEFVSISTNLTDRCVYISSDGGRLCRPYI
M.gallopavo      LVFLNGNILGVIRDHQKLVNTFRIMRRAGYINEFVSISTNLSDRCVYISSDGGRLCRPYI
M.musculus       LVFLNGNILGVIRDHKKLVSTFRLMRRAGYINEFVSISTNLTDRCVYISSDGGRLCRPYI
O.sativa         LVMFNGLILGKHRQPQRFANAMRKLRRSGIIGEFVSIFVNEKQHCIHIASDGGRVCRPLI
O.latipes        LVFLNGNILGVIRNHHKLVSTFRLMRRAGFINEFVSISTNLTDRCVYISSDGGRLCRPYI
P.troglodytes    LVFLNGNILGVIRDHKKLVNTFRLMRRAGYINEFVSISTNLTDRCVYISSDGGRLCRPYI
P.falciparum     FVILNGILLGVHKRPQKFMQRIRYLRRYGKIGQFVSIYDNFLHNAIYISTDGGRLCRPLI
P.abelii         LVFLNGNILGVIRDHKKLVNTFRLMRRAGYINEFVSISTNLTDRCVYISSDGGRLCRPYI
S.cerevisiae     GVYLNGTLIGSIRFPTKFVTQFRHLRRTGKVSEFISIYSNSHQMAVHIATDGGRICRPLI
S.pombe          LVYLNGAILGISRYPSLFVASFRKLRRSGKISPFIGIFINTHQRAVFISTDGGRICRPLI
S.purpuratus     VVFLNGNILGVVREHKRLMRTFRLVRRAGYISEFVSICPNHAQRCVTIASDGGRVCSI--
S.scrofa         LVFLNGNILGVIRDHKKLVNTFRLMRRAGYINEFVSISTNLTDRCVYISSDGGRLCRPYI
T.rubripes       IVFLNGNILGVIRDHQKLVGTFRLMRRAGFINEFVSISTNLTDRCVYISSDGGRLCRPYI
X.tropicalis     LVFLNGNILGVIRDHQKLVNTFRLMRRAGYINEFVSISTNISDRCVYISSDGGRLCRPYI
                                                                             


                       5710      5720      5730      5740      5750      5760
                 =========+=========+=========+=========+=========+=========+
A.carolinensis   IVKKQTPAVTEKHMEELAQGYRNFEDFLHESLVEYLDVNEENDCNIALYEQTIN------
A.gambiae        IVQAGRPL--------------------------YLDVNEENDSFIAYQERDIDP-----
A.thaliana       IADKGISRVKQHHMKELQDGVRTFDDFIRDGLIEYLDVNEENNALIALYESDGTTELDEG
B.distachyon     IADKGRSRVKEHHMKELRDGVRSFDDFLRDGLIEYLDVNEENNALIALYEHLDQDD----
C.elegans        IVKNGTPMLTQAHVQELKEGKMIFEDFVDDGIVEYLDVNEMNDALIAVYGREIGP-----
C.jacchus        IVKKQKPAVTNKHMEELAQGYRNFEDFLHESLVEYLDVNEENDCNIALYEHTIN------
C.neoformans     IVEKGRSRVTTEHVRLLKEGKVTFDHFLRAGLVEYLDVNEENDSFIACYENEIE------
P.tetraurelia    ------------------------------------------------------------
D.rerio          IVKNGKPAVKNKHIEELSQGYRTFEDFLHESLVEYLDVNEENDCNIALYEHMIS------
D.discoideum     IVDDQRPRLTQEHIEDLKDGLRTFDDFIREGIIEYLDVNEENDSFLAWREAAIQP-----
D.melanogaster   IVENRRPLVKQHHLDELNRGIRKFDDFLLDGLIEYLDVNEENDSFIAWNEDQIED-----
E.siliculosus    VVENNRLRIRKKHINDLHDPNYGWTNLMQQGVVEYIDTEEEETTMVAMEPKDLEES----
G.gallus         IVKKQKPAVTNKHMEELAQGYRNFEDFLHEGLVEYLDVNEENDCNIALYEHTIN------
G.gorilla        IVKKQKPAVTNKHMEELAQGYRNFEDFLHESLVEYLDVNEENDCNIALYEHTIN------
H.sapiens        IVKKQKPAVTNKHMEELAQGYRNFEDFLHESLVEYLDVNEENDCNIALYEHTIN------
I.tridecemlinea  IVKKQKPAVTNKHMEELAQGYRNFEDFLHESLVEYLDVNEENDCNIALYEHTIN------
M.mulatta        IVKKQKPAVTNKHMEELAQGYRNFEDFLHESLVEYLDVNEENDCNIALYEHTIN------
M.gallopavo      IVKKQKPAVTNKHMEELAQGYRNFEDFLHEGLVEYLDVNEENDCNIALYEHTIN------
M.musculus       IVKKQKPAVTNKHMEELAQGYRNFEDFLHESLVEYLDVNEENDCNIALYEHTIN------
O.sativa         IADKGIPRVKEHHMKQLRDGIRSFDDFLRDGLIEYLDVNEENNALIALYEHEDQDD----
O.latipes        IVKKGQPMVKSKHIEDLSQGYRNFEDFLHEGLVEYLDVNEENDCQIALYEHMIN------
P.troglodytes    IVKKQKPAVTNKHMEELAQGYRNFEDFLHESLVEYLDVNEENDCNIALYEHTIN------
P.falciparum     IIENGKSKLLPQHIKALENGTINFFDLLKSSVIEWIDVNEQNNLLIALNESDIS------
P.abelii         IVKKQKPAVTNKHMEELAQGYRNFEDFLHESLVEYLDVNEENDCNIALYEHTIN------
S.cerevisiae     IVSDGQSRVKDIHLRKLLDGELDFDDFLKLGLVEYLDVNEENDSYIALYEKDIV------
S.pombe          IVQNGLPKVESKHIRLLKEGKWGFEDFLKQGLVEYVDVNEENDSLISVYERDIT------
S.purpuratus     ------------------------------------------------------------
S.scrofa         IVKKQKPAVTNKHMEELAQGYRNFEDFLHESLVEYLDVNEENDCNIALYEHTIN------
T.rubripes       IVKKGQPMVKNKHIEDLSQGYRNFEDFLHDGLVEYLDVNEENDCQIALYEHMIH------
X.tropicalis     IVKNQRSAVTNKHIQELSQGYRSFEDFLHESLVEYLDVNEENDCNVALYEHTIT------
                                                                             


                       5770      5780      5790      5800      5810      5820
                 =========+=========+=========+=========+=========+=========+
A.carolinensis   -----KDTTHLEIEPFTLLGVCAGLIPYPHHNQSPRNTYQCAMGKQAMGTIGYNQRNRID
A.gambiae        -----EKTTHLEIEPFTLLGVCAGLVPYPHHNQSPRNTYQCAMGKQAMGIIGYNQKNRID
A.thaliana       AEAAKADTTHIEIEPFTILGVVAGLIPYPHHNQSPRNTYQCAMGKQAMGNIAYNQLNRMD
B.distachyon     --VQRSSITHIEIEPMTILGVVAGLIPYPHHNQSPRNTYQCAMGKQAMGNIAYNQLFRAD
C.elegans        ------ETTHLEIEPFTLLGVCAGLIPYPHHNQSPRNTYQCAMGKQAMGTIAYNQQKRID
C.jacchus        -----KDTTHLEIEPFTLLGVCAGLIPYPHHNQSPRNTYQCAMGKQAMGTIGYNQRNRID
C.neoformans     -----EGTTHLEIEPFTILGAVAGLIPYPHHNQSPRNTYQCAMGKQAIGAIAYNQLNRID
P.tetraurelia    ------------------------------------------------------------
D.rerio          -----KDTTHLEIEPFTLLGVCAGLIPYPHHNQSPRNTYQCAMGKQAMGTIGYNQRNRID
D.discoideum     ------WTTHLEIEPFTMLGCVAGLIPYPHHNQSPRNTYQCAMGKQAIGAIAYNQLTRID
D.melanogaster   ------RTTHLEIEPFTLLGVCAGLVPYPHHNQSPRNTYQCAMGKQAMGMIGYNQKNRID
E.siliculosus    -GGYSSTYTHCEIHPSMILGVCASIIPFPDHNQSPRNTYQSAMGKQAMGIYASNYQLRMD
G.gallus         -----KDTTHLEIEPFTLLGVCAGLIPYPHHNQSPRNTYQCAMGKQAMGTIGYNQRNRID
G.gorilla        -----KDTTHLEIEPFTLLGVCAGLIPYPHHNQSPRNTYQCAMGKQAMGTIGYNQRNRID
H.sapiens        -----KDTTHLEIEPFTLLGVCAGLIPYPHHNQSPRNTYQCAMGKQAMGTIGYNQRNRID
I.tridecemlinea  -----KDTTHLEIEPFTLLGVCAGLIPYPHHNQSPRNTYQCAMGKQAMGTIGYNQRNRID
M.mulatta        -----KDTTHLEIEPFTLLGVCAGLIPYPHHNQSPRNTYQCAMGKQAMGTIGYNQRNRID
M.gallopavo      -----KDTTHLEIEPFTLLGVCAGLIPYPHHNQSPRNTYQCAMGKQAMGTIGYNQRNRID
M.musculus       -----KDTTHLEIEPFTLLGVCAGLIPYPHHNQSPRNTYQCAMGKQAMGTIGYNQRNRID
O.sativa         --VQRSSITHIEIEPLTILGVVAGLIPYPHHNQSPRNTYQCAMGKQAMGNIAYNQLFRAD
O.latipes        -----KETTHLEIEPFTLLGVCAGLIPYPHHNQSPRNTYQCAMGKQAMGTIGYNQRNRID
P.troglodytes    -----KDTTHLEIEPFTLLGVCAGLIPYPHHNQSPRNTYQCAMGKQAMGTIGYNQRNRID
P.falciparum     -----LSTTHLEIDPLTILGVVAGLIPYPNHNQSPRNTYQCAMGKQAIGAIGYNQFVRCD
P.abelii         -----KDTTHLEIEPFTLLGVCAGLIPYPHHNQSPRNTYQCAMGKQAMGTIGYNQRNRID
S.cerevisiae     -----PSMTHLEIEPFTILGAVAGLIPYPHHNQSPRNTYQCAMGKQAIGAIAYNQFKRID
S.pombe          -----PDTTHLEIEPFTILGAVAGLIPYPHHNQSPRNTYQCAMGKQAIGAIAYNQLQRID
S.purpuratus     ------------------------------------------------------------
S.scrofa         -----KDTTHLEIEPFTLLGVCAGLIPYPHHNQSPRNTYQCAMGKQAMGTIGYNQRNRID
T.rubripes       -----KDTTHLEIEPFTLLGVCAGLIPYPHHNQSPRNTYQCAMGKQAMGTIGYNQRNRID
X.tropicalis     -----KHTTHLEIEPFTLLGVCAGLIPYPHHNQSPRNTYQCAMGKQAMGTIGYNQRNRID
                                                                             


                       5830      5840      5850      5860      5870      5880
                 =========+=========+=========+=========+=========+=========+
A.carolinensis   TLMYLLAYPQRPMVKTKTIELIEFEKLPAGQNATVAVMSYSGYDIEDALVLNKASLDRGF
A.gambiae        TLMYNIVYPQSPMVRSRTIELTNFDKLPAGQNATVAVMSYSGYDIEDALILNKASIDRGY
A.thaliana       TLLYLLVYPQRPLLTTRTIELVGYDKLGAGQNATVAVMSFSGYDIEDAIVMNKSSLDRGF
B.distachyon     SLLYLLVYAQRPLLTTKTIELVGYDKLGAGQNATVAVMSYSGYDIEDAIVMNKSSLDRGF
C.elegans        SIMYLLCYPQRPLVKSKTIELTNFEKLPAGANGIIAVMSYSGYDIEDALVLNKASLDRGY
C.jacchus        TLMYLLAYPQKPMVKTKTIELIEFEKLPAGQNATVAVMSYSGYDIEDALVLNKASLDRGF
C.neoformans     TLLYLMTYPQQPMVKTKTIELIGYNKLPAGQNATVAVMSYSGYDIEDALILNRASVDRGF
P.tetraurelia    ------------------------------------------------------------
D.rerio          TLMYLLAYPQRPMVKTKTIELIDFEKLPAGQNATVAVMSYSGYDIEDALVLNKASLDRGF
D.discoideum     TLLYLLVHTQRPLCQTRTIDLLNWYKLPAGHNATVAVMSYSGYDIEDALVMNKASLDRGF
D.melanogaster   SLMYNLVYPHAPMVKSKTIELTNFDKLPAGQNATVAVMSYSGYDIEDALILNKASIDRGY
E.siliculosus    TLAHVLHYPEKPLVTTRAMEHLHFRELPSGFNVIVGIMVYSGYNQEDSLIMNQSAVDRGL
G.gallus         TLMYLLAYPQKPMVKTKTIELIDFEKLPAGQNATVAVMSYSGYDIEDALVLNKASLDRGF
G.gorilla        TLMYLLAYPQKPMVKTKTIELIEFEKLPAGQNATVAVMSYSGYDIEDALVLNKASLDRGF
H.sapiens        TLMYLLAYPQKPMVKTKTIELIEFEKLPAGQNATVAVMSYSGYDIEDALVLNKASLDRGF
I.tridecemlinea  TLMYLLAYPQKPMVKTKTIELIEFEKLPAGQNATVAVMSYSGYDIEDALVLNKASLDRGF
M.mulatta        TLMYLLAYPQKPMVKTKTIELIEFEKLPAGQNATVAVMSYSGYDIEDALVLNKASLDRGF
M.gallopavo      TLMYLLAYPQKPMVKTKTIELIDFEKLPAGQNATVAVMSYSGYDIEDALVLNKASLDRGF
M.musculus       TLMYLLAYPQKPMVKTKTIELIDFEKLPAGQNATVAVMSYSGYDIEDALVLNKASLDRGF
O.sativa         SLLYLLVYAQRPLLTTKTIELVGYDKLGAGQNATVAVMSYSGYDIEDAIVMNKSSLDRGF
O.latipes        TLMYLLAYPQRPMVKTKTIELIDFEKLPAGQNATVAVMSYSGYDIEDALVLNKASLDRGF
P.troglodytes    TLMYLLAYPQKPMVKTKTIELIEFEKLPAGQNATVAVMSYSGYDIEDALVLNKASLDRGF
P.falciparum     TLLYLLVYPQKPLVKSKTIEFINFEKLPAGQNAIVAVMSFCGYDIEDAIVMNKSSIDRGF
P.abelii         TLMYLLAYPQKPMVKTKTIELIEFEKLPAGQNATVAVMSYSGYDIEDALVLNKASLDRGF
S.cerevisiae     TLLYLMTYPQQPMVKTKTIELIDYDKLPAGQNATVAVMSYSGYDIEDALVLNKSSIDRGF
S.pombe          TLLYLMVYPQQPMVKTKTIELIGYDKLPAGQNATVAIMSYSGYDIEDALVLNKSSIDRGF
S.purpuratus     ------------------------------------------------------------
S.scrofa         TLMYLLAYPQKPMVKTKTIELIEFEKLPAGQNATVAVMSYSGYDIEDALVLNKASLDRGF
T.rubripes       TLMYLLAYPQRPMVKTKTIEIIEFEKLPAGQNATVAVMSYSGYDIEDALILNKASLDRGF
X.tropicalis     TLMYLLAYPQRPMVKTKTIELIEFEKLPAGQNATVAVMSYSGYDIEDALVLNKASLDRGF
                                                                             


                       5890      5900      5910      5920      5930      5940
                 =========+=========+=========+=========+=========+=========+
A.carolinensis   GRCLVYKNAKCTLKRY---------TNQT-FDK---------------VMGPMLD-----
A.gambiae        GRCLVYKNSKCTIKRY---------SNQT-FDR---------------IMGPMKD-----
A.thaliana       GRCIVMKKIVAMSQKY---------DNCT-ADR---------------ILIPQRT-----
B.distachyon     GRCIAMKKYTVTTEKY---------ANGTISDR---------------IAKPQRD-----
C.elegans        GRCLVYKHVKGTAKKY---------PNQT-FDR---------------LLGPALD-----
C.jacchus        GRCLVYKNAKCTLKRY---------TNQT-FDK---------------VMGPMLD-----
C.neoformans     GRCHVLKKVTTPMRTF---------HNGS-HER---------------TAYPDPP-----
P.tetraurelia    ------------------------------------------------------------
D.rerio          GRCLVYKNAKCTLRRY---------TNQT-FDK---------------VMGPMLD-----
D.discoideum     GRCIVLKKQVTSIKKH---------GNDT-SDR---------------IFPPTPN-----
D.melanogaster   GRCLVYKNSKCTVKRY---------ANQT-FDR---------------IMGPMKD-----
E.siliculosus    FRSSFFRTLNDQEKAGRGMGDMAVLSAES-FER------------------PARE-----
G.gallus         GRCLVYKNAKCTLKRY---------TNQT-FDK---------------VMGPMLD-----
G.gorilla        GRCLVYKNAKCTLKRY---------TNQT-FDK---------------VMGPMLD-----
H.sapiens        GRCLVYKNAKCTLKRY---------TNQT-FDK---------------VMGPMLD-----
I.tridecemlinea  GRCLVYKNAKCTLKRY---------TNQT-FDK---------------VMGPMLD-----
M.mulatta        GRCLVYKNAKCTLKRY---------TNQT-FDK---------------VMGPMLD-----
M.gallopavo      GRCLVYKNAKCTLKRY---------TNQT-FDK---------------VMGPMLD-----
M.musculus       GRCLVYKNAKCTLKRY---------TNQT-FDK---------------VMGPMLD-----
O.sativa         GRCIAMKKYTVTKEKY---------EGGY-SDR---------------IVKPQRD-----
O.latipes        GRCLVYKNTKCTLRRY---------TNQT-FDK---------------VMGPMLD-----
P.troglodytes    GRCLVYKNAKCTLKRY---------TNQT-FDK---------------VMGPMLD-----
P.falciparum     GRCMSLRKHSVELKKY---------FNGS-NDIVLPSPLVINKLQQQRKEREIKSEIKNE
P.abelii         GRCLVYKNAKCTLKRY---------TNQT-FDK---------------VMGPMLD-----
S.cerevisiae     GRCETRRKTTTVLKRY---------ANHT-QDI---------------IGGMRVD-----
S.pombe          GRCQVFHKHSVIVRKY---------PNGT-HDR---------------IGDPQRD-----
S.purpuratus     ----VWKY----------------------------------------------------
S.scrofa         GRCLVYKNAKCTLKRY---------TNQT-FDK---------------VMGPVLD-----
T.rubripes       GRCLVYKNSKCTLRRY---------TNQT-FDK---------------VMGPMLD-----
X.tropicalis     GRCLVYKNAKCTLKRY---------SNQT-FDK---------------VMGPMLD-----
                                                                             


                       5950      5960      5970      5980      5990      6000
                 =========+=========+=========+=========+=========+=========+
A.carolinensis   ----AA-------------------------------------------------THKPI
A.gambiae        ----GV-------------------------------------------------TGKII
A.thaliana       ----GP-------------------------------------------------DA---
B.distachyon     ----KD-------------------------------------------------GALIK
C.elegans        ----PN-------------------------------------------------TRKPI
C.jacchus        ----AA-------------------------------------------------TRKPI
C.neoformans     ----PR-------------------------------------------------PDAYT
P.tetraurelia    ------------------------------------------------------------
D.rerio          ----AE-------------------------------------------------TRKPI
D.discoideum     ----DL-------------------------------------------------RQP--
D.melanogaster   ----AL-------------------------------------------------TNKVI
E.siliculosus    ----NT-------------------------------------------------AGMKH
G.gallus         ----AA-------------------------------------------------TRKPI
G.gorilla        ----AA-------------------------------------------------TRKPI
H.sapiens        ----AA-------------------------------------------------TRKPI
I.tridecemlinea  ----AA-------------------------------------------------TRKPI
M.mulatta        ----AA-------------------------------------------------TRKPI
M.gallopavo      ----AA-------------------------------------------------TRKPI
M.musculus       ----AA-------------------------------------------------TRKPI
O.sativa         ----KD-------------------------------------------------GALLK
O.latipes        ----AA-------------------------------------------------TRKPL
P.troglodytes    ----AA-------------------------------------------------TRKPI
P.falciparum     KEKDGIKQEHHHNNIDSKNNKSSYNNNVSKIDNVKKDSYGHKNISNESKDDIKKMANKDI
P.abelii         ----AA-------------------------------------------------TRKPI
S.cerevisiae     -----E-------------------------------------------------NGDPI
S.pombe          ----PE-------------------------------------------------TGEVV
S.purpuratus     ------------------------------------------------------------
S.scrofa         ----AA-------------------------------------------------TRKPI
T.rubripes       ----AA-------------------------------------------------TRKPI
X.tropicalis     ----AN-------------------------------------------------TRKQI
                                                                             


                       6010      6020      6030      6040      6050      6060
                 =========+=========+=========+=========+=========+=========+
A.carolinensis   WRHEILDADGICSPGEKVENKQVLVNKSMPTVTQTP-LEGSSV------PQ--QPQYKDV
A.gambiae        AKHECLDTDGIVSPGERLTCKQTMVNKEMPAVKSNN-P-IE------------QKESGQQ
A.thaliana       EKMQILDDDGLATPGEIIRPNDIYINKQVPVDTVTK-FTSALS------DS----QYRPA
B.distachyon     QNMRALDEDGFVAPGQIIRNHDIYVNKQTPKAIPKT-PGTTLT------DR----DYKDS
C.elegans        FKHKNLDQEGIVFAGARIMPKQTIINKHMPVVSGES-GPGASASANTIGIAGRQVDYKDV
C.jacchus        WRHEILDADGICSPGEKVENKQVLVNKSMPTVTQIP-LEGSNV------PQ--QPQYKDV
C.neoformans     FVD---KADGMTAPGATINQYDVMIHRETPVDTRGG-GADT-------------QLYKPM
P.tetraurelia    ------------------------------------------------------------
D.rerio          WRHSILDADGICSPGEKVENKQVLVNKSMPTVTQTP-LEGSAQ------PG--QPQYREV
D.discoideum     -KYGLLDSDGIAKPGELAQKGQILVNKYSPLNTIDA-TPNPELI------P--DSAYKSS
D.melanogaster   FKHDVLDTDGIVAPGEQVQNKQIMINKEMPAVTSMN-P-LQGQ------SA--QVPYTAV
E.siliculosus    GNYDKLEEDGLVAPGTRVSGTDVLIGKTTPLGGGVAGVTGGPG----------HRSKKDS
G.gallus         WRHEILDADGICSPGEKVENKQVLVNKSMPTVTQTP-LEGSSV------PQ--QPQYKDV
G.gorilla        WRHEILDADGICSPGEKVENKQVLVNKSMPTVTQIP-LEGSNV------PQ--QPQYKDV
H.sapiens        WRHEILDADGICSPGEKVENKQVLVNKSMPTVTQIP-LEGSNV------PQ--QPQYKDV
I.tridecemlinea  WRHEILDADGICSPGEKVENKQVLVNKSMPTVTQIP-LEGSNV------PQ--QPQYKDV
M.mulatta        WRHEILDADGICSPGEKVENKQVLVNKSMPTVTQIP-LEGSNV------PQ--QPQYKDV
M.gallopavo      WRHEILDADGICSPGEKVENKQVLVNKSMPTVTQTP-LEGSSV------PQ--QPQYKDV
M.musculus       WRHEILDADGICSPGEKVENKQVLVNKSMPTVTQIP-LEGSNV------PQ--QPQYKDV
O.sativa         QNMRALDEDGFAAPGLIIRNHDIYVNKQTPRNTKRD-SGAHLT------DR----DYKDS
O.latipes        WRHNILDADGICCPGEKVENKQILVNKSMPTVTQTP-LEGSAQ------PG--QPQYKEV
P.troglodytes    WRHEILDADGICSPGEKVENKQVLVNKSMPTVTQIP-LEGSNV------PQ--QPQYKDV
P.falciparum     RKYHSLDMDGVASIGYLIKEGQLYVNKFSPKNIKDH-VKDIGKV-----D---INDFKIN
P.abelii         WRHEILDADGICSPGEKVENKQVLVNKSMPTVTQIP-LEGSNV------PQ--QPQYKDV
S.cerevisiae     WQHQSLGPDGLGEVGMKVQSGQIYINKSVPTNSADA-PNPN--------NVNVQTQYREA
S.pombe          WKHGVVEDDGLAGVGCRVQPGQIYVNKQTPTNALDN-SITLGH------TQTVESGYKAT
S.purpuratus     ------------------------------------------------------------
S.scrofa         WRHEILDADGICSPGEKVENKQVLVNKSMPTVTQIP-LEGSNV------PQ--QPQYKDV
T.rubripes       WRHNILDADGICSPGERVENKQVLVNKSMPTVTQTP-LEGSTQ------TG--QPQYRDV
X.tropicalis     WRHEILDADGICSPGEKVENKQVLVNKYMPTVTQTP-LEGSSV------PQ--QPQYKDV
                                                                             


                       6070      6080      6090      6100      6110      6120
                 =========+=========+=========+=========+=========+=========+
A.carolinensis   PVTYKG--ATDSYIEKVMISSNAEDAFLIKILLRQTRRPEIGDKFSSRHGQKGVCGLIVP
A.gambiae        PIAYS---------ARVLVSTNNEEEFLVKILLRQTRRPEIGDKFSSRHGQKGVTGLIVE
A.thaliana       REYFKGPEGETQVVDRVALCSDKKGQLCIKYIIRHTRRPELGDKFSSRHGQKGVCGIIIQ
B.distachyon     PAVYKGVDGETTVVDRVMLCSDTNDKLTIKCIIRHTRRPEVGDKFSSRHGQKGVCGTIVQ
C.elegans        SITYKT--PTPSYAERVLLTYNEDEAHLFKVLLRQTRRPELGDKFSSRHGQKGVCGLIAQ
C.jacchus        PITYKG--ATDSYIEKVMISSNAEDAFLIKMLLRQTRRPEIGDKFSSRHGQKGVCGLIVP
C.neoformans     PVTHKT--PEPILVDKVMLTEGE-DGALIKILTRQTRRPELGDKFSSRHGQKGVCGLIVP
P.tetraurelia    ------------------------------------------------------------
D.rerio          PVSYKG--ATDSYIEKVMISSNAEDAFLVKILLRQTRRPEIGDKFSSRHGQKGVCGLIVP
D.discoideum     YMGYKY--DNPAFIDKVLLTSGDDEQLLIKMLMRSTRRPELGDKFSSRHGQKGVCGIIVK
D.melanogaster   PISYKG--PEPSYIERVMVSANAEEDFLIKILLRQTRIPEIGDKFSSRHGQKGVTGLIVE
E.siliculosus    STVMRS--HEDGIVDRVMLTTNQEGFKFCKVRIRNVRVPQIGDKFASRHGQKGTIGMTYR
G.gallus         PVTYKG--ATDSYIEKVMISSNAEDAFLIKMLLRQTRRPEIGDKFSSRHGQKGVCGLIVP
G.gorilla        PITYKG--ATDSYIEKVMISSNAEDAFLIKMLLRQTRRPEIGDKFSSRHGQKGVCGLIVP
H.sapiens        PITYKG--ATDSYIEKVMISSNAEDAFLIKMLLRQTRRPEIGDKFSSRHGQKGVCGLIVP
I.tridecemlinea  PITYKG--ATDSYIEKVMISSNAEDAFLIKMLLRQTRRPEIGDKFSSRHGQKGVCGLIVP
M.mulatta        PITYKG--ATDSYIEKVMISSNAEDAFLIKMLLRQTRRPEIGDKFSSRHGQKGVCGLIVP
M.gallopavo      PVTYKG--ATDSYIEKVMISSNAEDAFLIKMLLRQTRRPEIGDKFSSRHGQKGVCGLIVP
M.musculus       PITYKG--ATDSYIEKVMISSNAEDAFLIKMLLRQTRRPEIGDKFSSRHGQKGVCGLIVP
O.sativa         PAVYKGVDGETTVVDRVMLCSDTDEKLIIKCIIRHTRRPEVGDKFSSRHGQKGVCGTIVQ
O.latipes        PISYKG--STDSYIEKVMISSNAEDAFLIKILLRQTRRPEIGDKFSSRHGQKGVCGLIVP
P.troglodytes    PITYKG--ATDSYIEKVMISSNAEDAFLIKMLLRQTRRPEIGDKFSSRHGQKGVCGLIVP
P.falciparum     EIKYKS--VYPSYIDKIIFTENSEGLKLYKIIMRQTRLPELGDKFSSRHGQKGVVGLLVN
P.abelii         PITYKG--ATDSYIEKVMISSNAEDAFLIKMLLRQTRRPEIGDKFSSRHGQKGVCGLIVP
S.cerevisiae     PVIYRG--PEPSHIDQVMMSVSDNDQALIKVLLRQNRRPELGDKFSSRHGQKGVCGIIVK
S.pombe          PMTYKA--PEPGYIDKVMLTTTDSDQTLIKVLMRQTRRPELGDKFSSRHGQKGVCGVIVQ
S.purpuratus     -----------------------------------------------------FCHFSVP
S.scrofa         PITYKG--ATDSYIEKVMISSNAEDAFLIKMLLRQTRRPEIGDKFSSRHGQKGVCGLIVP
T.rubripes       PICYKG--STDSYIEKVMISSNAEDAFLIKILLRQTRRPEIGDKFSSRHGQKGVCGLIVP
X.tropicalis     PITYKG--PTDSYIEKVMISSNAEDAFLIKILLRQTRRPEIGDKFSSRHGQKGVCGLIVP
                                                                             


                       6130      6140      6150      6160      6170      6180
                 =========+=========+=========+=========+=========+=========+
A.carolinensis   QEDMPFCDSGICPDIIMNPHGYPSRMTVGKLIELLAGKAGVLDGRFHYGTAFGGS-----
A.gambiae        QEDLPFNDYGMSPDMVMNPHGFPSRMTVGKTLELLGSKAGVLEGKFHYGTAFGGS-----
A.thaliana       QEDFPFSELGICPDLIMNPHGFPSRMTVGKMIELLGSKAGVSCGRFHYGSAFGERSGHAD
B.distachyon     QEDFPFSERGICPDLIMNPHGFPSRMTIGKMIELLGGKAGVSCGRFHYGSAFGESSGNAD
C.elegans        QEDMPFNDLGMVPDMIMNPHGYPSRMTVGKLMELLSGKAGVVNGTYHYGTAFGGD-----
C.jacchus        QEDMPFCDSGICPDIIMNPHGFPSRMTVGKLIELLAGKAGVLDGRFHYGTAFGGS-----
C.neoformans     QADMPFNDQGIVPDIIMNPHGFPSRMTVGKMIELLSGKAGVVAGKLQYGTAFGGS-----
P.tetraurelia    ------------------------------------------------------------
D.rerio          QEDMPFCDSGICPDIIMNPHGYPSRMTVGKLIELLAGKAGVLDGRFHYGTAFGGS-----
D.discoideum     QEDMPFSDLGICPDIIMNPHGFPSRMTIGKMIELLAGKAGVLSGKFGFGTCFGGD-----
D.melanogaster   QEDMPFNDFGICPDMIMNPHGFPSRMTVGKTLELLGGKAGLLEGKFHYGTAFGGS-----
E.siliculosus    QEDMPWSIDGIVPDIIVNPHAIPSRMTIAQLIECLLGKVGVLTGTEGDATPFTDV-----
G.gallus         QEDMPFCDTGICPDIIMNPHGFPSRMTVGKLIELLAGKAGVLDGRFHYGTAFGGS-----
G.gorilla        QEDMPFCDSGICPDIIMNPHGFPSRMTVGKLIELLAGKAGVLDGRFHYGTAFGGS-----
H.sapiens        QEDMPFCDSGICPDIIMNPHGFPSRMTVGKLIELLAGKAGVLDGRFHYGTAFGGS-----
I.tridecemlinea  QEDMPFCDSGICPDIIMNPHGFPSRMTVGKLIELLAGKAGVLDGRFHYGTAFGGS-----
M.mulatta        QEDMPFCDSGICPDIIMNPHGFPSRMTVGKLIELLAGKAGVLDGRFHYGTAFGGS-----
M.gallopavo      QEDMPFCDTGICPDIIMNPHGFPSRMTVGKLIELLAGKAGVLDGRFHYGTAFGGS-----
M.musculus       QEDMPFCDSGICPDIIMNPHGFPSRMTVGKLIELLAGKAGVLDGRFHYGTAFGGS-----
O.sativa         QEDFPFSERGICPDLIMNPHGFPSRMTIGKMIELLGGKAGVSCGQFHYGSAFGEPSGNAD
O.latipes        QEDMPFCDSGVCPDIIMNPHGYPSRMTVGKLIELLAGKAGVLDGRFHYGTAFGGS-----
P.troglodytes    QEDMPFCDSGICPDIIMNPHGFPSRMTVGKLIELLAGKAGVLDGRFHYGTAFGGS-----
P.falciparum     QEDMPFTESGICPDLIMNPHGFPSRMTVGKLLELVASKAAVMDGEYKYGSIFSGT-----
P.abelii         QEDMPFCDSGICPDIIMNPHGFPSRMTVGKLIELLAGKAGVLDGRFHYGTAFGGS-----
S.cerevisiae     QEDMPFNDQGIVPDIIMNPHGFPSRMTVGKMIELISGKAGVLNGTLEYGTCFGGS-----
S.pombe          QEDMPFNDQGICPDIIMNPHGFPSRMTVGKMIELLSGKVGVLRGTLEYGTCFGGT-----
S.purpuratus     Q--------------IMNPHGFPSRMTVGKLIEFLAGKAGVLEGKFHYGTAFGGD-----
S.scrofa         QEDMPFCDSGICPDIIMNPHGFPSRMTVGKLIELLAGKAGVLDGRFHYGTAFGGS-----
T.rubripes       QEDMPFCDTGICPDIIMNPHGYPSRMTVGKLIELLAGKAGVLDGRFHYGTAFGGS-----
X.tropicalis     QEDMPFCETGICPDIIMNPHGYPSRMTVGKLIELLAGKAGVLDGRFHYGTAFGGS-----
                                                                             


                       6190      6200      6210      6220      6230      6240
                 =========+=========+=========+=========+=========+=========+
A.carolinensis   KVKDVCEDLIRYGYNYLGKDYVTSGITGEPLEAYIYFGPVYYQKLKHMVLDKMHARARGP
A.gambiae        KCSDLQDELFNHGFNYLGKDVFYSGITGEPLEVYIYSGPVYYQKLKHMVQDKMHARARGP
A.thaliana       KVETISATLVEKGFSYSGKDLLYSGISGEPVEAYIFMGPIYYQKLKHMVLDKMHARGSGP
B.distachyon     HVDDISHTLVKHGFSYNGKDFLYSGILGHPLEAYIFMGPIYYQKLKHMVLDKMHARASGP
C.elegans        QVKDVCEELAACGYNYMGKDMLTSGITGQPLSAYIYFGPIYYQKLKHMVLDKMHARARGP
C.jacchus        RVKDVCEDLVRHGYNYLGKDYVTSGITGEPLEAYIYFGPVYYQKLKHMVLDKMHARARGP
C.neoformans     KVVDMSQILIDNGFSYGGKDMLTSGITGQPMEAYVYFGPIYYQKLKHMVMDKMHARPTGP
P.tetraurelia    ------------------------------------------------------------
D.rerio          KVKDVCEDLIRYGYNYQGKDYVTSGITGEPLEAYIYFGPVYYQKLKHMVLDKMHARARGP
D.discoideum     RVENISKVLISKGFSYGGKDYVTSGVTGEPLACFIFFGPIFYQKLKHMVMDKMHARARGP
D.melanogaster   KVEDIQAELERHGFNYVGKDFFYSGITGTPLEAYIYSGPVYYQKLKHMVQDKMHARARGP
E.siliculosus    TVANVSSTLHKIGFQKHGNEVMYSGHTGRMLNTKVFLGPTFYQRLKHLVDDKIHSRSRGP
G.gallus         KVKDVCEDLIRHGYNYLGKDYVTSGITGEPLEAYIYFGPVYYQKLKHMVLDKMHARARGP
G.gorilla        KVKDVCEDLVRHGYNYLGKDYVTSGITGEPLEAYIYFGPVYYQKLKHMVLDKMHARARGP
H.sapiens        KVKDVCEDLVRHGYNYLGKDYVTSGITGEPLEAYIYFGPVYYQKLKHMVLDKMHARARGP
I.tridecemlinea  KVKDVCEDLVRHGYNYLGKDYVTSGITGEPLEAYIYFGPVYYQKLKHMVLDKMHARARGP
M.mulatta        KVKDVCEDLVRHGYNYLGKDYVTSGITGEPLEAYIYFGPVYYQKLKHMVLDKMHARARGP
M.gallopavo      KVKDVCEDLIRHGYNYLGKDYVTSGITGEPLEAYIYFGPVYYQKLKHMVLDKMHARARGP
M.musculus       KVKDVCEDLVRHGYNYLGKDYVTSGITGEPLEAYIYFGPVYYQKLKHMVLDKMHARARGP
O.sativa         KVEDISRTLVKHGFSYNGKDLLYSGILGHPCQAYVFMGPIYYQKLKHMVLDKMHARASGP
O.latipes        KVKDVCEDLIRYGYNYQGKDYVTSGITGEPLEAYIYFGPVYYQKLKHMVLDKMHARARGP
P.troglodytes    KVKDVCEDLVRHGYNYLGKDYVTSGITGEPLEAYIYFGPVYYQKLKHMVLDKMHARARGP
P.falciparum     PFEEIAEILFRYGFNCSSKELLYSGLTGEPLETYIFMGPIYYQKLKHMVQDKIHARARGP
P.abelii         KVKDVCEDLVRHGYNYLGKDYVTSGITGEPLEAYIYFGPVYYQKLKHMVLDKMHARARGP
S.cerevisiae     KLEDMSKILVDQGFNYSGKDMLYSGITGECLQAYIFFGPIYYQKLKHMVLDKMHARARGP
S.pombe          KVEDASRILVEHGYNYSGKDMLTSGITGETLEAYIFMGPIYYQKLKHMVMDKMHARARGP
S.purpuratus     KVVDICEDLRKHGFNYLGKDFLTSGITGEPLTAYIYFGPVYYQKLKHMVLDKMHARAKGP
S.scrofa         KVKDVCEDLVRHGYNYLGKDYVTSGITGEPLEAYIYFGPVYYQKLKHMVLDKMHARARGP
T.rubripes       KVKDVCEDLIRHGYNYQGKDYVTSGITGEPLEAYIYFGPVYYQKLKHMVLDKMHARARGP
X.tropicalis     KVKDVCEDLIRYGYNYLGKDYVTSGITGEPLEAYIYFGPVYYQKLKHMVLDKMHARARGP
                                                                             


                       6250      6260      6270      6280      6290      6300
                 =========+=========+=========+=========+=========+=========+
A.carolinensis   RAVLTRQPTEGRSRDGGLRLGEMERDCLIGYGASMLLLERLMISSDAFEVDVCGQCGLLG
A.gambiae        RAVLTRQPTQGRSREGGLRLGEMERDCLISYGASMLIMERLMISSDAFDVDVCNVCGRLA
A.thaliana       RVMMTRQPTEGKSKNGGLRVGEMERDCLIAYGASMLIYERLMISSDPFEVQVCRACGLLG
B.distachyon     RVLLTRQPTEGRSRDGGLRLGEMERDCLIAYGASMLIFERLLLSSDPYQVQVCRKCGLLG
C.elegans        RAALTRQPTEGRSREGGLRLGEMERDCLIAYGASMLLIERLMVSSDEFKVDVCTGCGVIG
C.jacchus        RAVLTRQPTEGRSRDGGLRLGEMERDCLIGYGASMLLLERLMISSDAFEVDVCGKCGLLG
C.neoformans     RANLTRQPTEGRSKDGGLRLGEMERDCLIGYGATQLLLERLMISSDAFETQVCESCGMLG
P.tetraurelia    ------------------------------------------------------------
D.rerio          RAVLTRQPTEGRSRDGGLRLGEMERDCLIGYGASMLLLERLMISSDAFEVDVCGQCGLLG
D.discoideum     TVTLTRQPTEGRARGGGLRLGEMERDCLIGYGASALIMERLMISSDRFTVYACKNCGFLG
D.melanogaster   KAVLTRQPTQGRSREGGLRLGEMERDCLISYGASMLIMERLMISSDAFEVDVCRTCGRMA
E.siliculosus    VMNLTRQPMEGRGRDGGLRMGEMERDCLISHGCANFLRDRLFINSDAYRVHVCDKCGLIA
G.gallus         RAVLTRQPTEGRSRDGGLRLGEMERDCLIGYGASMLLLERLMISSDAFEVDVCGQCGLLG
G.gorilla        RAVLTRQPTEGRSRDGGLRLGEMERDCLIGYGASMLLLERLMISSDAFEVDVCGQCGLLG
H.sapiens        RAVLTRQPTEGRSRDGGLRLGEMERDCLIGYGASMLLLERLMISSDAFEVDVCGQCGLLG
I.tridecemlinea  RAVLTRQPTEGRSRDGGLRLGEMERDCLIGYGASMLLLERLMISSDAFEVDVCGQCGLLG
M.mulatta        RAVLTRQPTEGRSRDGGLRLGEMERDCLIGYGASMLLLERLMISSDAFEVDVCGQCGLLG
M.gallopavo      RAVLTRQPTEGRSRDGGLRLGEMERDCLIGYGASMLLLERLMISSDAFEVDVCGQCGLLG
M.musculus       RAVLTRQPTEGRSRDGGLRLGEMERDCLIGYGASMLLLERLMISSDAFEVDVCGQCGLLG
O.sativa         RVLLTRQPTEGRSRDGGLRLGEMERDCLIAYGASMLIFERLLISSDPYQVQVCRKCGLLG
O.latipes        RAVLTRQPTEGRSRDGGLRLGEMERDCLIGYGASMLLLERLMISSDAFEVDVCGQCGLLG
P.troglodytes    RAVLTRQPTEGRSRDGGLRLGEMERDCLIGYGASMLLLERLMISSDAFEVDVCGQCGLLG
P.falciparum     RQLLTRQPTEGRSKEGGLRLGEMERDCLIAYGVSNLLLERLMLSSDVCNVYICEDCGMMG
P.abelii         RAVLTRQPTEGRSRDGGLRLGEMERDCLIGYGASMLLLERLMISSDAFEVDVCGQCGLLG
S.cerevisiae     RAVLTRQPTEGRSRDGGLRLGEMERDCVIAYGASQLLLERLMISSDAFEVDVCDKCGLMG
S.pombe          RAVLTRQPTEGRSRDGGLRLGEMERDCLIAYGASQLLLERLMISSDACDVDVCGQCGLLG
S.purpuratus     RAVLTRQPTEGRARDGGLRLGEMERDCLIGYGASMLLLERLMISSDQFQVDVCGDCGLIG
S.scrofa         RAVLTRQPTEGRSRDGGLRLGEMERDCLIGYGASMLLLERLMISSDAFEVDVCGQCGLLG
T.rubripes       RAVLTRQPTEGRSRDGGLRLGEMERDCLIGYGASMLLLERLMISSDAFEVDVCGQCGLLG
X.tropicalis     RAVLTRQPTEGRSRDGGLRLGEMERDCLIGYGASMLLLERLMISSDAFEVDVCGKCGLLG
                                                                             


                       6310      6320      6330      6340      6350      6360
                 =========+=========+=========+=========+=========+=========+
A.carolinensis   YSGW------CHYCKSSCHVSSLRIPYACKLLFQELQSMNIIPRLKL--SK----YN-E-
A.gambiae        YSSW------CHNCRSSASVSTISMPYACKLLFQELTSMNIVPRLKL--QN-------Y-
A.thaliana       YYNYKLKKAVCTTCKNGDNIATMKLPYACKLLFQELQSMNVVPRLKL--TE-------A-
B.distachyon     YYNYKLKTSFCSMCKNGENMAKMRMPYACKLLFQELQSMNVVPRLKL--TE-------G-
C.elegans        SKGW------CQKCRSSKSMANIKIPYACKLLFQELQSMNIVPRLDL--AR----YT-E-
C.jacchus        YSGW------CHFCKSSRQVSSLRIPYACKLLFQELQSMNIIPRLKL--SK----YN-EM
C.neoformans     YNNW------CPKCKSGKGVVGLTIPYAAKLLIQELMGMNIMPKLCL--ED----T--V-
P.tetraurelia    ------------------------------------------------------------
D.rerio          YSGW------CHYCKSSCHVSSLRIPYACKLLFQELQSMNIIPRLKL--AR----YN-E-
D.discoideum     YEGY------CQYCKSSVDISTIQIPYACKLLFQELQAMNIVPRLKL--VD-------S-
D.melanogaster   YCSW------CHFCQSSANVSKISMPYACKLLFQELTSMNVVPKMIL--EN-------Y-
E.siliculosus    VANLRKMSFECRGCKGKSDVSQIHVPYACKLLFQELMAMCIAPRMFVRADAGAVQVQ-Y-
G.gallus         YSGW------CHYCKSSCHVSSLRIPYACKLLFQELQSMNIIPRLKL--AK----YN-E-
G.gorilla        YSGW------CHYCKSSCHVSSLRIPYACKLLFQELQSMNIIPRLKL--SK----YN-EM
H.sapiens        YSGW------CHYCKSSCHVSSLRIPYACKLLFQELQSMNIIPRLKL--SK----YN-EM
I.tridecemlinea  YSGW------CHYCKSSCHVSSLRIPYACKLLFQELQSMNIIPRLKL--SK----YN-E-
M.mulatta        YSGW------CHYCKSSCHVSSLRIPYACKLLFQELQSMNIIPRLKL--SK----YN-EM
M.gallopavo      YSGW------CHYCKSSCHVSSLRIPYACKLLFQELQSMNIIPRLKL--AK----YN-E-
M.musculus       YSGW------CHYCKSSCHVSSLRIPYACKLLFQELQSMNIIPRLKL--AK----YN-E-
O.sativa         YYNHKLKASYCSMCKNGENMAKMRMPYACKLLFQELQAMNVVPRLKL--TE-------G-
O.latipes        YSGW------CHYCKSSCHVSSLKIPYACKLLFQELQSMNIIPRLKL--SR----YN-E-
P.troglodytes    YSGW------CHYCKSSCHVSSLRIPYACKLLFQELQSMNIIPRLKL--SK----YN-EM
P.falciparum     YDLY------CTFCKKCDKNIVIQMPYACKLLFQELQTMNVFPKIIV--KE-------V-
P.abelii         YSGW------CHYCKSSCHVSSLRIPYACKLLFQELQSMNIIPRLKL--SK----YN-EM
S.cerevisiae     YSGW------CTTCKSAENIIKMTIPYAAKLLFQELLSMNIAPRLRL--ED----IFQQ-
S.pombe          YKGW------CNSCQSTREVVKMTIPYAAKLLFQELLSMNIVPRLAL--ED----EFKY-
S.purpuratus     HSGW------CPYCHCSQNISSLRIPYACKLLFQELQSMNIIPRLRL--KK----YT-EM
S.scrofa         YSG---------------------------------------------------------
T.rubripes       YSGW------CHYCKSSCHVSSLRIPYACKLLFQELQSMNIIPRLKL--SR----YN-E-
X.tropicalis     YSGW------CHFCKSSCHVSSLRIPYACKLLFQELQSMNIIPRLKL--AK----YN-E-
                                                                             


                       6370      6380      6390      6400      6410      6420
                 =========+=========+=========+=========+=========+=========+
A.carolinensis   --MGSASSKDDSAPVK-T---PEA-----------------------SVPFSLT-DVFLF
A.gambiae        --MDAQTETEV---PAGS---VSD-----------------------EPFLGPL-DIVLL
A.thaliana       ------------------MTSA------L-YASDLFKQLKS--IMGT-DSL--SDDVVLV
B.distachyon     --MALE------------AARSWAASVLPPELAAAAGG--DP--------LVAL-AATAA
C.elegans        --MLAWIVSG----------------------------------------LDTS-DLVVL
C.jacchus        INMGDSNVDTSSTVSE-A--AAEE-----------------------VSLFSMT-DMILI
C.neoformans     -------------------------------------------------MLSAI-DIVII
P.tetraurelia    ------------------------------------------------------------
D.rerio          --MEDTEPQ-PVSD---Q--DQTP-----------------------DPLLSSL-DIFLF
D.discoideum     --M----------------EI------LES-I--------D--FIEV-LILDNL-GA---
D.melanogaster   --MASEQTIDGAAAIPSG---GGD-----------------------EPFLGLL-DVALL
E.siliculosus    --M----------------AS---SGASAW------------------PY------IGLL
G.gallus         --MGDAGMESTVSPPE-G--TAQD------------------------SFLSMT-DVFLI
G.gorilla        INMGDSHMDTSSTVSE-A--VAEE-----------------------VSLFSMT-DMILF
H.sapiens        INMGDSHVDTSSTVSE-A--VAEE-----------------------VSLFSMT-DMILF
I.tridecemlinea  ------------------------------------------------------------
M.mulatta        INMGDSHVDTSSTVSE-A--VAEE-----------------------VSLFSMT-DVILF
M.gallopavo      --MGGAGVESAVSPPE-S--TAQD------------------------SFFSMT-DVFLI
M.musculus       --MGDSHEDTSATVPE-A--VAEE-----------------------VSLFSTT-DIVLF
O.sativa         --MDSGG-GGGGGALR-PSALDLVAALLTGRGRPEEEG--WPPSLAENRHLIVL-LTTSL
O.latipes        -----MEPE-THTE---P--VGDE-----------------------EPLFSNL-DLFLF
P.troglodytes    INMGDSHVDTSSTVSE-A--VAEE-----------------------VSLFSMT-DMILF
P.falciparum     ------------------------------------------------MFMRWN-KISRY
P.abelii         GCM-----------YS-S--PPEE-----------------------PAL----------
S.cerevisiae     -----------------------M-----------------------PFGIDNT-DFTVL
S.pombe          --------------------------------------------------MKTY-EYVLL
S.purpuratus     --FPEAEASESIEATS-T--EAMG-----------------------GPAIGTL-DIFLL
S.scrofa         --MGDSNVDTGTTTSE-M--VAEE-----------------------VSLFSAT-DMVLF
T.rubripes       --M--LRPP-LRAH---P--WEEV-----------------------EPLFSNL-DIFLF
X.tropicalis     --MGESCAEQDMCTSE-QGNSSPE-----------------------EAFFSMA-DMFLL
                                                                             


                       6430      6440      6450      6460      6470      6480
                 =========+=========+=========+=========+=========+=========+
A.carolinensis   SII-IGLLTY-WFF-FRKKKEEVPD-------FA-KIQTP-SAAQ--STND-S-SFIEKM
A.gambiae        VSL-LAGTAW-YLL-KGKKKESQASQF---K-SY-SIQPTTVNTM--TMVE-N-SFIKKL
A.thaliana       IATTSLALVAG-FVVLLW-KK-TT--ADRSGELKPLMI---PKS-LMAKDE-D-DD-LDL
B.distachyon     ALV-AGLLVLA-VW-FRSGGGAPSK-PA--AT-P-LR-----P--PPVKVD-A-D--ADA
C.elegans        TLL-AGGAII-FLF-MKVFNQQPSSSR---YSPT-VASVT-TSAA--ASKSNQ-SFIDRM
C.jacchus        SLI-VGLVTY-LFL-FRKKKEEVPE-------FT-KIQ---TSTS--SVKE-S-SFVEKM
C.neoformans     TLT-IALPLL-FFF-RESLPFIGGKTR--AA-AP-HAAIA-NKASVDEGDP-S-DFVGKM
P.tetraurelia    ------------------------------------------------------------
D.rerio          SLI-AGLLIY-WFF-FRKKVEPIPE-------MK-PFT---LVTA--PIRE-T-SFIEKM
D.discoideum     III-VAVIVGTYLY-MNKPPPPPPV-FN--K--P-NNK---INKEAQKPKK-T-I--TKN
D.melanogaster   AVL-IGGAAF-YFL-RSRKKEEEPT-----R-SY-SIQPTTVCTT--SASD-N-SFIKKL
E.siliculosus    ALV-PGLL----FL-RPKRKPDEAD-----------------------IE--E----EEE
G.gallus         SLI-TGLFTY-WFF-FRKKKEEIPD-------LP-KIQ---TVSS--PARD-S-SFIEKM
G.gorilla        SLI-VGLLTY-WFL-FRKKKEEVPE-------FT-KIQ---TLTS--SVRE-S-SFVEKM
H.sapiens        SLI-VGLLTY-WFL-FRKKKEEVPE-------FT-KIQ---TLTS--SVRE-S-SFVEKM
I.tridecemlinea  -----------------------------------------------------------M
M.mulatta        SVI-VGLLTY-WFL-FRKKKEEVPE-------FT-KIQ---TLTS--SVRE-S-SFVEKM
M.gallopavo      SLI-AGLFTY-WFF-FRKKKEEIPD-------LP-KIQ---TVSS--PARD-S-SFIEKM
M.musculus       SLI-VGVLTY-WFI-FKKKKEEIPE-------FS-KIQ---TTAP--PVKE-S-SFVEKM
O.sativa         AVL-VGCGVAL-LV-RRSSISAPAV-RA--QE-P-QPRAP-AP--AKRKQE-A-E--PDP
O.latipes        SLI-IGLVVY-YFM-SRKKPEPIPE-------FK-KID---TPQV--SQRE-T-SFIEKM
P.troglodytes    SLI-VGLLTY-WFL-FRKKKEEVPE-------FT-KIQ---TLTS--SVRE-S-SFVEKM
P.falciparum     TLL-SGMVVSFWLF-YRSENFNLLR-------RL-ISKLR-SLFP--LFIK-N-NFLNN-
P.abelii         -----------------------------------------RRTS--SVRE-S-SFVEKM
S.cerevisiae     AGL-VLAVLL-YVK-RNSIK-----EL--LM-SD-DGDIT-AVSS--G-N--R-DIAQVV
S.pombe          VII-LILSLC-YFI-YNNFL-----NK--PK-AP-ERRVV-ATD----------SIVELM
S.purpuratus     SLS-VGVGVY-WFF-FRKKAENKTE-G---L-VN-PITSS-SMTM--SSNS-GSGFITKM
S.scrofa         SLI-VGLLTY-WFI-FRKKKDEVPE-------FT-KIE---TTTS--SVKD-S-SFVEKM
T.rubripes       SLI-VGIVIY-WFM-FRKKPEPVPE-------FK-KIE---TQAP--STRE-T-SFIEKM
X.tropicalis     SLI-VGLLTY-WFF-FRKKKEETIE-------FT-KIQ-P-TVSS--SVRE-S-SFIEKM
                                                                             


                       6490      6500      6510      6520      6530      6540
                 =========+=========+=========+=========+=========+=========+
A.carolinensis   KKDGKNIVVFYGSQTGTAEEFANRLAKDAH-RYGMRGM-SADPEEYNLSDLNRLPE-IDK
A.gambiae        QSSGRRLVVFYGSQTGTAEEFAGRLAKEGI-RYQMKGM-VADPEECNMEELLMLKD-IDK
A.thaliana       GSGKTRVSIFFGTQTGTAEGFAKALSEEIKARYEKAAVKVIDLDDYAADDDQYEEKLKKE
B.distachyon     DDGRKRVTIFFGTQTGTAEGFAKAMAEEAKVRYEKTVFKVVDLDDYAAEDDEYEEKLKKE
C.elegans        KNENRQVLIMYGSQTGTAEEMSGRLAKDLT-RYTKKAV-VVDPEDIECEDLNRLSE-VED
C.jacchus        KKTGRNVIVFYGSQTGTAEEFANRLSKDAH-RYGMRGM-TADPEEYDLADLSSLPE-IEN
C.neoformans     TRANKRCVIFYGSQTGTAEEYAIRLAKEAKSRYGLSSL-VCDPEEYDMNMLDQVP---ED
P.tetraurelia    ------------------------------------------------------------
D.rerio          KKTNRSVVVFYGSQTGTGEEFANRLSKDAH-RYGMKGM-AADPEEYDMSELSRLKE-IPK
D.discoideum     EDGKKVMKIFFGTQTRTAEDFSRIIEKECK-KIGIPCE-VVDLESYEHEQ-----ELHSE
D.melanogaster   KASGRSLVVFYGSQTGTGEEFAGRLAKEGI-RYRLKGM-VADPEECDMEELLQLKD-IDN
E.siliculosus    AAPNDTINVYFGSQTGTAESFAQTIAAEGR-RHGFHID-VVDLEEFSASE------LLEK
G.gallus         KKTGRNIVVFYGSQTGTAEEFANRLSKDAH-RYGLRGM-AADPEEYDLSDLSRLSE-IDK
G.gorilla        KKTGRNIIVFYGSQTGTAEEFANRLSKDAH-RYGMRGM-SADPEEYDLADLSSLPE-IEN
H.sapiens        KKTGRNIIVFYGSQTGTAEEFANRLSKDAH-RYGMRGM-SADPEEYDLADLSSLPE-IDN
I.tridecemlinea  KKTGRNIIVFYGSQTGTAEEFANRLSKDAH-RYGMRGM-AADPEEYDLADLSSLPE-IHN
M.mulatta        KKTGRNIIVFYGSQTGTAEEFANRLSKDAH-RYGMRGM-SADPEEYDLADLSSLPE-IDN
M.gallopavo      KKTGRNIVVFYGSQTGTAEEFANRLSKDAH-RYGLRGM-AADPEEYDLSDLSRLSE-IDK
M.musculus       KKTGRNIIVFYGSQTGTAEEFANRLSKDAH-RYGMRGM-SADPEEYDLADLSSLPE-IDK
O.sativa         DDGRQRVAVFFGTQTGTAEGFAKALAEEAKSRYDKAVFKVLDLDEYAADDEEYEQKLKKE
O.latipes        KKTGKNIIVFYGSQTGTAEEFANRLSKDAQ-RYGMKGM-AADPEEYTMGELARMSE-IKN
P.troglodytes    KKTGRNIIVFYGSQTGTAEEFANRLSKDAH-RYGMRGM-SADPEEYDLADLSSLPE-IDN
P.falciparum     -EIKNSVKIYFGSQSGTAEEFAKELKANLNDLFHIQAN-IIDLEYFNKEEIKSF------
P.abelii         KKTGRNIIVFYGSQTGTAEEFANRLSKDAH-RYGMRGM-SADPEEYDLADLSSLPE-IDN
S.cerevisiae     TENNKNYLVLYASQTGTAEDYAKKFSKELVAKFNLNVM-CADVENYDFESLNDVP---V-
S.pombe          EAEKLTAAVFFGSQTGTAEDFAYRFSTEAKANFNLTNM-VFDLENYDLTDLDNFD---RS
S.purpuratus     KSSSRNVVVFYGSQTGTGEEFSVRLAKEAQ-RYGLKGM-VADPEENEMEDLSQLAD-IEN
S.scrofa         KKTGRNIIVFYGSQTGTAEEFANRLSKDAH-RYGMRGM-AADPEEYDLSDLSSLPE-IEN
T.rubripes       KKMGKNIIVFYGSQTGTGEEFANRLSKDAQ-RYGMKGM-AADPEEYDMAELSRLSE-IEN
X.tropicalis     KKTGKNIVVFYGSQTGTGEEFANRLAKDAH-RYGMRGM-AADPEEFEMTDLSRLTE-IEN
                                                                             


                       6550      6560      6570      6580      6590      6600
                 =========+=========+=========+=========+=========+=========+
A.carolinensis   SLAVFCMATYGEGDPTDNAQDFYDWLQETD--------A---DLSGLKFAVFGLGNKTYE
A.gambiae        SLAVFCLATYGEGDPTDNCMEFYDWIQNND--------L---DMTGLNYAVFGLGNKTYE
A.thaliana       TLAFFCVATYGDGEPTDNAARFYKWFTEENE-----RDI---KLQQLAYGVFALGNRQYE
B.distachyon     TLVLFFLATYGDGEPTDNAARFYKWFTEGKE-----KEV---WLKDFKYAVFGLGNRQYE
C.elegans        ALLVLCIATYGEGDPTDNAVTLVEYLNAGD--------C---DLSGVRFAVFGLGNKTYE
C.jacchus        SLVVFCMATYGEGDPTDNAQDFYDWLQETD--------M---DLSGVKFSVFGLGNKTYE
C.neoformans     ACVIFVMATYGEGEPTDNANAMMELLQEPEPEFS--Q--GGSTLENLNYVIFGLGNRTYE
P.tetraurelia    ------------------------------------------------------------
D.rerio          SMAVFCMATYGEGDPTDNAQEFYDWLQGTD--------D---DLEGVNFAVFGLGNKTYE
D.discoideum     SFVMFLVATHGEGDPTDNAKEFYLWLTNDER-----PTD---LLNGVPFTVFGLGNKTYE
D.melanogaster   SLAVFCLATYGEGDPTDNAMEFYEWITSGD--------V---DLSGLNYAVFGLGNKTYE
E.siliculosus    GKAIFLMATYGDGEPTDNASEFTTWLKNESGEL---ESD---YLATVEFTVFGLGNTQYE
G.gallus         SLAVFCMATYGEGDPTDNAQDFYDWLQEAD--------T---DLSGLRFAVFGLGNKTYE
G.gorilla        ALVVFCMATYGEGDPTDNAQDFYDWLQETD--------V---DLSGVKFAVFGLGNKTYE
H.sapiens        ALVVFCMATYGEGDPTDNAQDFYDWLQETD--------V---DLSGVKFAVFGLGNKTYE
I.tridecemlinea  SLVVFCMATYGEGDPTDNAQDFYDWLQEAD--------V---DLSGVKYAVFGLGNKTYE
M.mulatta        ALVVFCMATYGEGDPTDNAQDFYDWLQETD--------V---DLSGVKFAVFGLGNKTYE
M.gallopavo      SLAVFCMATYGEGDPTDNAQDFYDWLQEAD--------A---DLSGLRFAVFGLGNKTYE
M.musculus       SLVVFCMATYGEGDPTDNAQDFYDWLQETD--------V---DLTGVKFAVFGLGNKTYE
O.sativa         IIALFFVATYGDGEPTDNAARFYKWFGEGNE-----RGE---WLSNLRFGVFGLGNRQYE
O.latipes        SLAIFCMATYGEGDPTDNAQDFYDWLQEND--------D--EDLSGLNYTVFALGNKTYE
P.troglodytes    ALVVFCMATYGEGDPTDNAQDFYDWLQETD--------V---DLSGVKFAVFGLGNKTYE
P.falciparum     GIRIFIVATYGDGEPTDNAVEFFKWLKSLNND-----ND---YFRNTKYSIMGLGSKQYK
P.abelii         ALVVFCMATYGEGDPTDNAQDFYDWLQXTD--------V---DLSGVS------------
S.cerevisiae     -IVSIFISTYGEGDFPDGAVNFEDFICNAE----------AGALSNLRYNMFGLGNSTYE
S.pombe          KLLVFFLATYGEGEPTDNAEAFLQLLEGDDTVFSSGKGIEDTPFEGIRYAIFGLGNHTYE
S.purpuratus     SLAIFCVATYGEGDPTDNAQEFYDWLQDGN--------G---DLSGVKYTVFGLGNKTYE
S.scrofa         ALAVFCMATYGEGDPTDNAQDFYDWLQEAD--------V---DLSGVKYAVFGLGNKTYE
T.rubripes       SLAIFCMATYGEGDPTDNAQDFYDWLQEND--------D--EELSGLNYTVFALGNKTYE
X.tropicalis     ALAVFCMATYGEGDPTDNAQDFYDWLQETD--------I---DLAGLKYAVFGLGNKTYE
                                                                             


                       6610      6620      6630      6640      6650      6660
                 =========+=========+=========+=========+=========+=========+
A.carolinensis   HFNAMGKYVDKRMEELGAQRIFELGLGDDDG-KRTGSFL---------------------
A.gambiae        HYNKVGIYVDKRLEELGANR----------------------------------------
A.thaliana       HFNKIGIVLDEELCKKGAKRLIEVGLGDDDQ-SIEDDFNAWKESLWSELDKLLKDEDDKS
B.distachyon     HFNKVAKVVDELLEEQGGKRLVPCGLGDDDQ-CIEDDFTAWKEQVWPELDQLLRDDDDTT
C.elegans        HFNEIGIQMDKQLEKLGAKRIFHLGLGDDDA-NLEEDFMIWREAFLPKVAEEFGWELNT-
C.jacchus        HFNAMGKYVDKRLEQLGAQRIFDLGLGDDDG-NLEEDFITWREQFWPAVCEHFGVEATG-
C.neoformans     FYNEVAKKLDKRLTELGAKRIGERGEGDDDK-SMEEDYLAWKDPMWTAFAERMGVEEGG-
P.tetraurelia    ------------------------------------------------------------
D.rerio          HYNAMGKYTDKRLAELGGKRVFDLGLGDDDS-NLEEDFISWKEQFWPAVCEYFGVEATG-
D.discoideum     HYNAVARVIDRRMEELGGKRVFERGEGDDDA-TLEEDFNRWKKDMWPVVCKFLGYELKST
D.melanogaster   HYNKVAIYVDKRLEELGANRVFELGLGDDDA-NIEDDFITWKDRFWPAVCDHFGIEGGG-
E.siliculosus    HYNMVGKSTNEGMEKLGAQRMFEYGEGDDDN-QLEEDFEAWREKMWMALVMRFGGMGGVD
G.gallus         HFNAMGKYVDKRLEELGAQRIFELGLGDDDG-NLEEDFITWREQFWPAVCEHFGVEATG-
G.gorilla        HFNAMGKYVDKRLEQLGAQRIFELGLGDDDG-NLEEDFITWREQFWPAVCEHFGVEATG-
H.sapiens        HFNAMGKYVDKRLEQLGAQRIFELGLGDDDG-NLEEDFITWREQFWPAVCEHFGVEATG-
I.tridecemlinea  HFNAMGKYVDQRLEQLGAQRIFELGLGDDDG-NLEEDFITWREQFWPAVCEFFGVEATG-
M.mulatta        HFNAMGKYVDKRLEQLGAQRIFELGLGDDDG-NLEEDFITWREQFWPAVCEHFGVEATG-
M.gallopavo      HFNAMGKYVDKRLEELGAQRIFELGLGDDDG-NLEEDFITWREQFWPAVCEHFGVEATG-
M.musculus       HFNAMGKYVDQRLEQLGAQRIFELGLGDDDG-NLEEDFITWREQFWPAVCEFFGVEATG-
O.sativa         HFNKVGKVVDQLLAEQGGKRIVPLGLGDDDQ-CIEDDFNAWKELLWPELDKLLRVEDDKS
O.latipes        HYNAMGKYVDKRLEELGAKRIFDLGLGDDDS-NLEEDFVSWREQFWPAVCEHFGVEASA-
P.troglodytes    HFNAMGKYVDKRLEQLGAQRIFELGLGDDDG-NLEEDFITWREQFWPAVCEHFGVEATG-
P.falciparum     HFNKIAKKLDTFLLNFKAHQISETIYGDDDD-NIYHDFEVWKNKFFMQLPKLLNMKNIPI
P.abelii         ------------------------------S-RLEEDFITWREQFWPAVCEHFGVEATG-
S.cerevisiae     FFNGAAKKAEKHLSAAGAIRLGKLGEADDGAGTTDEDYMAWKDSILEVLKDELHLDEQE-
S.pombe          YYNAMAKKVDAAMTRLGATRVGNLGLGDDAAGMLEEDYLQWKDDTLPEIGKLFHLQEVH-
S.purpuratus     HYNAMGKYLDKRLEELGGERIFELGLGDDDQ-NIEEDFVTWKDRFWPAVCEYYGLEATG-
S.scrofa         HFNAMGKYVDKRLEQLGAQRIFDLGLGDDDG-NLEEDFITWREQFWPAVCEHFGVEATG-
T.rubripes       HYNAMGKYVDKRLEELGAKRIFDLGLGDDDG-NLEEDFVSWREQFWPAVCEHFGVEPLG-
X.tropicalis     HFNAMGKYVDKRLEELGAERIFELGMGDDDG-NLEEDFITWREQFWPAVCEHFGVEATG-
                                                                             


                       6670      6680      6690      6700      6710      6720
                 =========+=========+=========+=========+=========+=========+
A.carolinensis   ------------------------------------------------------------
A.gambiae        ------------------------------------------------------------
A.thaliana       VA---TPYT-------------AVIPEYRVVTHD-PR---------------F--T-TQK
B.distachyon     GA--STPYT-------------AAIPEYRVVFID-KS----DL---------V--VEDKS
C.elegans        ----E---A-------------ETMRQYQLEPVEE-----G--KA------LFKGEF---
C.jacchus        ----E---E-------------SSIRQYELVVHSD---IDA--AK------VYMGEM---
C.neoformans     ----A---G--------------DVPDFVVKELHD-H--SP--EK------VYHGEL---
P.tetraurelia    ------------------------------------------------------------
D.rerio          ----E---D-------------SSIRQFELVVHND---INM--NQ------VYTGEM---
D.discoideum     ED------D-------------KFVPRFRMVTLN-QD--SKDINDPFIK--IVS-TPLKP
D.melanogaster   ----E---E-------------VLIRQYRLLEQPD---VQP--DR------IYTGEI---
E.siliculosus    GV----DK--------------AVDLPFSVKMLTAAE----AA---------C--HAGV-
G.gallus         ----E---E-------------SSIRQYELVVHTD---VNM--NK------VYTGEM---
G.gorilla        ----E---E-------------SSIRQYELVVHTD---IDA--AK------VYMGEM---
H.sapiens        ----E---E-------------SSIRQYELVVHTD---IDA--AK------VYMGEM---
I.tridecemlinea  ----E---E-------------SSIRQYELVVHTD---MDM--AK------VYVGEM---
M.mulatta        ----E---E-------------SSIRQYELVVHTD---IDA--AK------VYVGEM---
M.gallopavo      ----E---E-------------SSIRQYELVVHTD---VNM--NK------VYTGEM---
M.musculus       ----E---E-------------SSIRQYELVVHED---MDT--AK------VYTGEM---
O.sativa         AA--PTPYT-------------AAIPEYRVVLVK-PE----EA---------M--HINKS
O.latipes        ----D---E-------------LSIRQYELKLHND---VNM--NK------VFTGEI---
P.troglodytes    ----E---E-------------SSIRQYELVVHTD---IDA--AK------VYMGEM---
P.falciparum     YVPKE--DIIELTSWRDMAEIKLDIQYYDHLIEED---NKK--EKNVVTENIINESV---
P.abelii         ----E---E-------------SSIRQYELVVHTD---IDA--AK------VYMGEM---
S.cerevisiae     ----A---K--------------FTSQFQYTVLNE-I--TD---S------MSLGEP---
S.pombe          ----K---E--------------YNPMFEVIEKPE-ISNTS--ST------VFLGEP---
S.purpuratus     ----D---E-------------SNIRQYAVTEHTE---DVP--EK------VFSGEV---
S.scrofa         ----E---E-------------SSIRQYELVVHTD---MDT--AV------VYTGEM---
T.rubripes       ----D---E-------------SSIRQYELKVHTD---INM--NK------VYSGEI---
X.tropicalis     ----E---D-------------SSIRQYELVVHTD---ENM--NK------VYTGEM---
                                                                             


                       6730      6740      6750      6760      6770      6780
                 =========+=========+=========+=========+=========+=========+
A.carolinensis   ------------------------------------------------------------
A.gambiae        ------------------------------------------------------LHK-A-
A.thaliana       S-M--------ESNVANGN---TTIDIHHPCRVD--------------VAVQKELHTHE-
B.distachyon     W-T--------LANG---N---GVIDIHHPCRSN--------------VAVRKELHKPA-
C.elegans        G-R--------LGAYERPR---PPFDVKNPYLAT--------------VAINDELHTEH-
C.jacchus        G-R--------LKSYENQK---PEEGLGLPP-----------------------------
C.neoformans     S-SR-----ALLASASGTNTPVGAYGVKNPYPAP--------------VLSSKELFSVG-
P.tetraurelia    ------------------------------------------------------------
D.rerio          G-R--------LKSFQTQK---PPFDAKNPFLAS--------------VAVNRKLNK-G-
D.discoideum     K-L--------STD---NK---VIYDMKNPYYAE--------------VLENRELHSNE-
D.melanogaster   A-R--------LHSIQNQR---PPFDAKNPFLAP--------------IKVNRELHK-G-
E.siliculosus    S-E--------TAAA--SS---SKF-YWHGCDAP--------------VVVNRELRAQAP
G.gallus         G-R--------LKSYENQK---PPFDAKNPFLAV--------------VTENRKLNE-G-
G.gorilla        G-R--------LKSYENQK---PPFDAKNPFLAA--------------VTTNRKLNQ-G-
H.sapiens        G-R--------LKSYENQK---PPFDAKNPFLAA--------------VTTNRKLNQ-G-
I.tridecemlinea  G-R--------LKSYENQK---PPFDAKNPFLAA--------------VTTNRKLNQ-G-
M.mulatta        G-R--------LKSYENQK---PPFDAKNPFLAA--------------VTTNRKLNQ-G-
M.gallopavo      G-R--------LKSYENQK---PPFDAKNPFLAV--------------VTENRKLNE-G-
M.musculus       G-R--------LKSYENQK---PPFDAKNPFLAA--------------VTTNRKLNQ-G-
O.sativa         F-S--------LSNG---H---AVYDIQHPCRAN--------------VAVRRELHTPA-
O.latipes        G-R--------LKSFEVQK---PPFDAKNPFLAP--------------VTVNRRLNK-A-
P.troglodytes    G-R--------LKSYENQK---PPFDAKNPFLAA--------------VTTNRKLNQ-G-
P.falciparum     TNN------QQLLNHNQNN---LSINNKSNYISTDIIGKFYFNHLTGKVISNT-------
P.abelii         G-R--------LKSYENQK---PPFDAKNPFLAA--------------VTTNRKLNQ-G-
S.cerevisiae     S-AHYLPSHQLNRNADGIQL--GPFDLSQPYIAP--------------IVKSRELFS-S-
S.pombe          S-R------QQLKGNVASK---APRSQANPFFSS--------------PVRSLELFK-S-
S.purpuratus     A-R--------LNAFKNQK---PPYDAKNPYLSA--------------ITVNRELHQ-G-
S.scrofa         G-R--------LKSYENQK---PPFDAKNPFLAV--------------VTTNRKLNQ-G-
T.rubripes       G-R--------LKSFEVQK---PPFDSKNPFLAP--------------VTVNRRLNK-G-
X.tropicalis     G-R--------LKSYETQK---PPFDAKNPFLAN--------------ATVNRKLNE-G-
                                                                             


                       6790      6800      6810      6820      6830      6840
                 =========+=========+=========+=========+=========+=========+
A.carolinensis   -------------------------------PRG-WCRLQPFLEPP--E-----------
A.gambiae        GGRSCM------------HVEFDIEGSKMRYEAGDHLAMYPVNDRDLVE-----------
A.thaliana       SDRSCI------------HLEFDISRTGITYETGDHVGVYAENHVEIVE-----------
B.distachyon     SDRSCI------------HLEFDISGTGLVYETGDHVGVYSENSVDTVE-----------
C.elegans        SDRSCR------------HIEFSVEGSRIRYEAGDHLAVFPTNDPVLVD-----------
C.jacchus        ---------------------------------------LPQGRSQESA-----------
C.neoformans     GDRNCI------------HIEFDITGTGMTYQHGDHVGIWPSNSDVEVD-----------
P.tetraurelia    ------------------------------------------------------------
D.rerio          GNRHLM------------HIELDITESKIRYDSGDHVAVYPTNDSAMVN-----------
D.discoideum     SDRSCR------------HIEFKLG-DEVSYTTGDHLGVFPINDSKLVE-----------
D.melanogaster   GGRSCM------------HIELSIEGSKMRYDAGDHVAMFPVNDKSLVE-----------
E.siliculosus    GVGSTR------------HVEIDLQGTSVGYHTADNLAILPLNDATTAE-----------
G.gallus         GERHLM------------HLELDISNSKIRYESGDHVAVYPANDASLVN-----------
G.gorilla        TERHLM------------HLELDISDSKIRYESGDHVAVYPANDSALVN-----------
H.sapiens        TERHLM------------HLELDISDSKIRYESGDHVAVYPANDSALVN-----------
I.tridecemlinea  TERHLM------------HLELDISDSKIRYESGDHVAVYPANDCTLVN-----------
M.mulatta        TERHLM------------HLELDISDSKIRYESGDHVAVYPANDSALVN-----------
M.gallopavo      GERHLM------------HLELDISNSKIRYESGDHVAVYPANDASLVN-----------
M.musculus       TERHLM------------HLELDISDSKIRYESGDHVAVYPANDSTLVN-----------
O.sativa         SYRSCI------------HLEFDISGTGLTYETGDHVGVYAENCTETVE-----------
O.latipes        GDRHLM------------HLELDITGSKLRYESGDHVAVFPTNDSALVN-----------
P.troglodytes    TERHLM------------HLELDISDSKIRYESGDHVAVYPANDSALVN-----------
P.falciparum     --KLLKNVDLSNNGDKVNHINISIED-NIIYKAADNLSILTKNTKEVITWWLKRLNIDEK
P.abelii         TERHLM------------HLELDISDSKIRYESGDHVAVYPANDSALVN-----------
S.cerevisiae     NDRNCI------------HSEFDLSGSNIKYSTGDHLAVWPSNPLEKVE-----------
S.pombe          GSRNCL------------HLELDIADSGMRYQTGDYASICPMNPSQAVD-----------
S.purpuratus     GDRSCM------------HIEFDISGSRIRYESGDHVAVYPTNDPELVA-----------
S.scrofa         TERHLM------------HLELDISDSKIRYESGDHVAVYPANDSALVN-----------
T.rubripes       GERHLM------------HLELDITGSKIRYESGDHVAVFPTNDSALVN-----------
X.tropicalis     GDRHFM------------HLELDITGSKIRYESGDHVAVYPANDAALVN-----------
                                                                             


                       6850      6860      6870      6880      6890      6900
                 =========+=========+=========+=========+=========+=========+
A.carolinensis   ---------QTGFS------------------------ANG---------MSSV--A---
A.gambiae        ---------RLGRL------------------------CNAEL----DTVFSLI--N---
A.thaliana       ---------EAGKL------------------------LGHSL----DLVFSIH--A---
B.distachyon     ---------QAERL------------------------LGLSP----DTVFSIH--A---
C.elegans        ---------RLINM------------------------LQFDP----DHAFRLV--N---
C.jacchus        ---------ALAAL------------------------PGPQR----C-SVPRG--S---
C.neoformans     ---------RMLAV------------------------LGLAASGRRQAIVDIE--S---
P.tetraurelia    ------------------------------------------------------------
D.rerio          ---------RIGER------------------------LGVDL----DAVISLK--N---
D.discoideum     ---------QLIKR------------------------LGVNG----DDMIALV--P---
D.melanogaster   ---------KLGQL------------------------CNADL----DTVFSLI--N---
E.siliculosus    ---------KLCAQ------------------------LGYDP----DSFFILE--H---
G.gallus         ---------QLGEI------------------------LGTDL----DTVMSLN--N---
G.gorilla        ---------QLGKI------------------------LGADL----DVVMSLN--N---
H.sapiens        ---------QLGKI------------------------LGADL----DVVMSLN--N---
I.tridecemlinea  ---------QLGEI------------------------LGADL----DVVMSLN--N---
M.mulatta        ---------QLGKI------------------------LGADL----DVVMSLN--N---
M.gallopavo      ---------QFGEI------------------------LGTDL----DTVMSLN--N---
M.musculus       ---------QIGEI------------------------LGADL----DVIMSLN--N---
O.sativa         ---------EVENL------------------------LGYSP----DTLFSIH--A---
O.latipes        ---------RLGEI------------------------LGVDL----DVVISLN--N---
P.troglodytes    ---------QLGKI------------------------LGADL----DIVMSLN--N---
P.falciparum     EKTKKFTFVKRNKLIDNSFTMNDPKDDVKNETFNNDVNKGN---NKTNIDYNSNNNGNNN
P.abelii         ---------QLGKI------------------------LGADL----DVIMXLN--N---
S.cerevisiae     ---------QFLSI------------------------FNLDP----ETIFDLK--P---
S.pombe          ---------DLLEV------------------------LGLKEKR--DTVIIVK--P---
S.purpuratus     ---------AIGKI------------------------LDADL----DTVFTLT--N---
S.scrofa         ---------QLGEI------------------------LGTDL----DIVMSLN--N---
T.rubripes       ---------KLGQV------------------------LGVDL----DVVISLN--N---
X.tropicalis     ---------KLGEI------------------------LGADL----ETVISLN--N---
                                                                             


                       6910      6920      6930      6940      6950      6960
                 =========+=========+=========+=========+=========+=========+
A.carolinensis   HGKE---------SNKKHPFPCPTTYRTALTHYLDITNPPRTNVLYELAQYASAASEQEH
A.gambiae        TDTD---------SSKKHPFPCPTTYRTALTHYLEITALPRTHILKELAEYCGEEKDKEF
A.thaliana       DKEDGSPL---E-SAVPPPFPGPCTLGTGLARYADLLNPPRKSALVALAAYATEPSEAEK
B.distachyon     DAEDGSPR---KGGSLAPPFPSPCTLRTALLRYADLLNSPKKAALVALASHASDPTEAER
C.elegans        VDED---------ASKRHPFPCPTTFRTALSHYVDICAPVKSHVLKAISEYCTDDTEKEF
C.jacchus        SHEE---------SNKKHPFPCPTSYRTALTYYLDITNPPRTNVLYELAQYASEPSEQEL
C.neoformans     LDP----------ALAKVPFPTPATYDAIFRHYLDISAVASRQTIAFLARYAPSEAAREK
P.tetraurelia    ------------------------------------------------------------
D.rerio          LDEE---------SNKKHPFPCPTTYRTALTHYLDINNMPRTNVLYELAQYASDPQEQEN
D.discoideum     IDQEGS--------VIKAS-FGPMTIRRAFSEHLDITNPVRKSVLRALAESTTNEEEKKR
D.melanogaster   TDTD---------SSKKHPFPCPTTYRTALTHYLEITAIPRTHILKELAEYCTDEKEKEL
E.siliculosus    DD------------NHKPVFPTPCTVRDAFLRFMDIMAIPRRSLLEQLTPYVEDDAEREA
G.gallus         LDEE---------SNKKHPFPCPTSYRTALTYYLDITNPPRTNVLYELAQYATDTGEQEQ
G.gorilla        LDEE---------SNKKHPFPCPTSYRTALTYYLDITNPPRTNVLYELAQYASETSEQEL
H.sapiens        LDEE---------SNKKHPFPCPTSYRTALTYYLDITNPPRTNVLYELAQYASEPSEQEL
I.tridecemlinea  LDEE---------SNKKHPFPCPTSYRTALTYYLDITNPPRTNVLYELAQYASEPAEQEH
M.mulatta        LDEE---------SNKKHPFPCPTSYRTALTYYLDITNPPRTNVLYELAQYASEPSEQEL
M.gallopavo      LDGE---------YGS--------------------------------------------
M.musculus       LDEE---------SNKKHPFPCPTTYRTALTYYLDITNPPRTNVLYELAQYASEPSEQEH
O.sativa         DQEDGTPL---FGGSLPPPFPSPCTVGTALARYADLLSFPKKSALIALASHASDPKDAER
O.latipes        LDEE---------SNKKHPFPCPTTYRTALTHYLDIMNPPRTNVLYELAQYASEPKDQEN
P.troglodytes    LDEE---------SNKKHPFPCPTSYRTALTYYLDITNPPRTNVLYELAQYASEPSEQEL
P.falciparum     NNN---NYNEYDDNHIYVPFPTPCSVEDALSYYCDLTTIPRLNILKKFKCFIKDIEELKM
P.abelii         LDEE---------SNKKHPFPCPTSYRTALTYYLDITNPPRTNVLYELAQYASEPSEQEL
S.cerevisiae     LDP-----------TVKVPFPTPTTIGAAIKHYLEITGPVSRQLFSSLIQFAPNADVKEK
S.pombe          IDT-----------LDKAPVLSPTTYDTVFRYYYEICGIVSRQLLSFIAPFAPTPESKQE
S.purpuratus     VDEE---------ASKKHPFPCPTSYQTAFSHYLDITSCPRANVLKEISEYATDPADKEK
S.scrofa         LDEE---------SNKRHPFPCPTTYRTALTYYLDITNPPRTNVLYELAQYASEPSEQEQ
T.rubripes       LDEE---------SNKKHPFPCPTTYRTALTHYLDITQPPRTNVLYELAQYATDGKDQEN
X.tropicalis     LDEE---------SNKKHPFPCPTTYRTALTYYLDITNPPRTNVLYELAQYATDSKEQEN
                                                                             


                       6970      6980      6990      7000      7010      7020
                 =========+=========+=========+=========+=========+=========+
A.carolinensis   LRKMASSSAEGKALYLSWVVEARRNILAI-LQD------------TP-SLRPPIDH-LCE
A.gambiae        LRFISSTAPDGKAKYQEWVQDSCRNIVHV-LED------------IP-SCHPPIDH-VCE
A.thaliana       LKHLTSP--DGKDEYSQWIVASQRSLLEV-MAA------------FP-SAKPPLGVFFAA
B.distachyon     LRFLASP--AGKDEYSQWIVASQRSLLEV-MAA------------FP-SAKPPLGVFFAA
C.elegans        LNKLSTANEEGLKEYARYIVKERRSIVDV-LTD------------QK-TCKPPIEY-LLE
C.jacchus        LRKMASSSGEGKELYLSWVVEARRHILAI-LQD------------CP-SLRPPIDH-LCE
C.neoformans     LTRWGT----DKEAYANEIDGPALKLAEV-LQSASSDSVEPPFESQT-VWPIPFDR-IVS
P.tetraurelia    ------------------------------------------------------------
D.rerio          MRKMASASPEGKALYQSWVLDSERNILAI-LED------------LP-SLNPPIDH-LCE
D.discoideum     LLYLATE--EANEEYNKYIKNDFRGVVDL-LES------------FP-GLQPLIA-HFLE
D.melanogaster   LRSMASISPEGKEKYQSWIQDACRNIVHI-LED------------IK-SCRPPIDH-VCE
E.siliculosus    MHLLSSK--EGKEKYHREVEEPGWTLADL-ILE------------RFSSLSMTLD-HFLH
G.gallus         LRKMASSSAEGKALYLSWVVEARRNILAI-LQD------------MP-SLRPPIDH-LCE
G.gorilla        LRKMASSSGEGKELYLSWVVEARRHILAI-LQD------------CP-SLRPPIDH-LCE
H.sapiens        LRKMASSSGEGKELYLSWVVEARRHILAI-LQD------------CP-SLRPPIDH-LCE
I.tridecemlinea  LRKMASSSGEGKELYLSWVVEARRHILAI-LQD------------YP-SLRPPIDH-LCE
M.mulatta        LRKMASSSGEGKELYLSWVVEARRHILAI-LQD------------CP-SLRPPIDH-LCE
M.gallopavo      --PMHISSSSSAALYLSWVVEARRNILAI-LQD------------MP-SLHPPIDH-LCE
M.musculus       LHKMASSSGEGKELYLSWVVEARRHILAI-LQD------------YP-SLRPPIDH-LCE
O.sativa         LRHLASP--AGKKEYSQWIVSSQRSLLEV-MTE------------FP-SAKPPLGVFFAA
O.latipes        MRKMASSSPEGKALYQTWVLDSCRNILAI-LED------------MP-SLKPPVDH-LCE
P.troglodytes    LRKMASSSGEGKELYLSWVVEARRHILAI-LQD------------CP-SLRPPIDH-LCE
P.falciparum     FNFILS--NNQRNTFFNICKECDMTFIEFVDMF------------MQ-SAVFELSP-FLQ
P.abelii         LRKMASSSGEGKELYLSWVVEARRHILAI-LQD------------CP-SLRPPIDH-LCE
S.cerevisiae     LTLLSK----DKDQFAVEITSKYFNIADA-LKYLSDGA--------K-WDTVPMQF-LVE
S.pombe          LEKLGN----DYDYFKKNVVDLHLNLAQV-LRRVSPDA--------P-FTKLPFSM-LLE
S.purpuratus     LLLMSSATPEGKKEYSDWVTKCHRNIVAI-LED------------LP-SVKVPLDH-LCE
S.scrofa         LRKMASSSGEGKELYLSWVVEARRHILAI-LQD------------YP-SLRPPIDH-LCE
T.rubripes       LRKMASSSPEGKSLYQNWVLDACRNILAI-LED------------MP-SLRPPIDH-LCE
X.tropicalis     LRKMASSAQDGKALYLSWVVESRRNILAI-LED------------IP-SLRPPLDH-LCE
                                                                             


                       7030      7040      7050      7060      7070      7080
                 =========+=========+=========+=========+=========+=========+
A.carolinensis   LLPRLQARYYSIASSSKVHSNSIHICAVVVEYTTKT------G-RVNKGVATNWL--K-S
A.gambiae        LLPRLQPRYYSISSSSKLHPTTVHVTAVLVKYETKT------G-RLNKGVATTFL--A-E
A.thaliana       IAPRLQPRYYSISSSPRLAPSRVHVTSALVYGPTPT------G-RIHKGVCSTWM--K-N
B.distachyon     VAPRLQPRYYSISSSPKMAPSRIHVTCALVYGPTPT------E-RIHQGVCSTWM--K-N
C.elegans        LLPRLQARYYSIASSPRLNEEKIAICAVVTKYSIGD------R-DIN-GVCTRYL--T-T
C.jacchus        LLPRLQARYYSIASSSKVHPNSVHICAVVVEYESKA------G-RINKGVATNWL--R-A
C.neoformans     SVPRLQPRYYSISSSSKLHPNAIHVTAVVLKYQPTVSPPHHHEPRWVFGLSTNFI--L-N
P.tetraurelia    ------------------------------------------------------------
D.rerio          LLPRLQARYYSIASSSKVHPHCIHICAVVIEYNTKT------G-RVFKGVATNWL--K-G
D.discoideum     FTPRLPARMYSISSSPHNKNGVVSITSVVVNFTTGN------Q-RAHNGVASTWL--S-H
D.melanogaster   LLPRLQPRYYSISSSAKLHPTDVHVTAVLVEYKTPT------G-RINKGVATTYL--K-N
E.siliculosus    VVPHLHPRYYTISSSSSVSPSRVHITVAVLEQDRSQ------G-RLYRGICSSFL--S-S
G.gallus         LLPRLQARYYSIASSSKVHPNSIHICAVTVEYETKT------G-RLNKGVATNWL--K-D
G.gorilla        LLPRLQARYYSIASSSKVHPNSVHICAVVVEYETKA------G-RINKGVATNWL--R-A
H.sapiens        LLPRLQARYYSIASSSKVHPNSVHICAVVVEYETKA------G-RINKGVATNWL--R-A
I.tridecemlinea  LLPRLQARYYSIASSSKVHPNSVHICAVAVEYETKS------G-RVNKGVATSWL--R-A
M.mulatta        LLPRLQARYYSIASSSKVHPNSVHICAVVVEYETKA------G-RINKGVATNWL--R-A
M.gallopavo      LLPRLQARYYSIASSSKVHPNSIHICAVTVEYETKT------G-RLNKGVATNWL--K-N
M.musculus       LLPRLQARYYSIASSSKVHPNSVHICAVAVEYEAKS------G-RVNKGVATSWL--R-T
O.sativa         IAPRLQPRYYSISSSPRMTPTRIHVTCALVYGQTPT------G-RIHKGVCSTWM--K-N
O.latipes        LLPRLQARYYSIASSSKVHPNSIHICAVVVEYSTKT------G-RVNKGVATNWL--K-N
P.troglodytes    LLPRLQARYYSIASSSKVHPNSVHICAVVVEYETKA------G-RINKGVATNWL--R-A
P.falciparum     LIPRNTPKSYTISSSPKESKDILSLTVKKKQYCIHS------LRRALKNLKTNDMFPKLN
P.abelii         LLPRLQARYYSIASSSKVHPNSVHICAVVVEYETKA------G-RINKGVATNWL--R-A
S.cerevisiae     SVPQMTPRYYSISSSSLSEKQTVHVTSIVENFPNPELP---DAP-PVVGVTTNLL--R-N
S.pombe          NMAHMKPRYYSISSSSVVHPDKVHVTAVVDKKEWT------DKNHIFYGLTTNYL--L-A
S.purpuratus     LLPRLHARYYSISSSPKVSPDRISITAVLIRYTTPT------G-RIGKGVATNWL--K-D
S.scrofa         LLPRLQARYYSIASSSKVHPNSVHICAVVVEYETKS------G-RVNKGVATSWL--R-A
T.rubripes       LLPRLQARYYSIASSSKVHPNSIHICAVVVEYQTKT------G-RLNKGVATNWL--K-N
X.tropicalis     LLPRLQARYYSIASSSKVHPNSIHVCAVLVEYETKT------G-RENKGVATNWL--K-N
                                                                             


                       7090      7100      7110      7120      7130      7140
                 =========+=========+=========+=========+=========+=========+
A.carolinensis   -----------------------KQPM-D---------NG---------H-K--------
A.gambiae        -----------------------KHPN-D---------GE---------P-A--------
A.thaliana       -----------------------AVPAEK---------SH----------EC--------
B.distachyon     -----------------------TLPLEY---------SE----------EC--------
C.elegans        -----------------------KDA-------------------------G--------
C.jacchus        -----------------------KEPARE---------NG---------G-R--------
C.neoformans     -----------------------VKMAHSGENTPVE--GDVSHVSMKKVP-SYKLSGPRG
P.tetraurelia    ------------------------------------------------------------
D.rerio          ------------------------KHVTD---------NG---------H-K--------
D.discoideum     -----------------------LKVGD--------------------------------
D.melanogaster   -----------------------KQPQ-G---------SE-----------E--------
E.siliculosus    -----------------------LEPHDGA-TVEGATIDG----------SG--------
G.gallus         -----------------------KVPN-E---------NG---------R-N--------
G.gorilla        -----------------------KEPAGE---------NG---------G-R--------
H.sapiens        -----------------------KEPAGE---------NG---------G-R--------
I.tridecemlinea  -----------------------KEPAGE---------NG---------R-R--------
M.mulatta        -----------------------KEPAGE---------NG---------G-R--------
M.gallopavo      -----------------------KVPN-E---------NG---------R-N--------
M.musculus       -----------------------KEPAGE---------NG---------R-R--------
O.sativa         -----------------------SIPLEE---------SQ----------EC--------
O.latipes        ------------------------KLV-----------NG---------H-K--------
P.troglodytes    -----------------------KEPAGE---------NG---------G-R--------
P.falciparum     EQKLRELCSRRWFKGSSSYYLTEELNVND-------------------------------
P.abelii         -----------------------KEPAGE---------NG---------G-R--------
S.cerevisiae     -----------------------IQLAQNNVNIAET-----------NLPVHYDLNGPRK
S.pombe          -----------------------HCRHMHGEKIPHP-----------NGL-EYTLEGPRK
S.purpuratus     -----------------------KIPNGP---------ET---------T-P--------
S.scrofa         -----------------------KEPAGE---------NG---------R-R--------
T.rubripes       ------------------------KLISD---------NG---------H-K--------
X.tropicalis     -----------------------KQPS-D---------NG---------H-K--------
                                                                             


                       7150      7160      7170      7180      7190      7200
                 =========+=========+=========+=========+=========+=========+
A.carolinensis   -------SVVPMYVRKSQFRLPFKS-S--TPVLMIGPGTGVAPFIGFIQERGLH-K----
A.gambiae        -------PRVPIFIRKSQFRLPPKP-E--TPVIMVGPGTGLAPFRGFIQERDHC-K----
A.thaliana       -------SGAPIFIRASNFKLPSNP-ST--PIVMVGPGTGLAPFRGFLQERMAL-K----
B.distachyon     -------SWAPIFVRQSNFKLPADP-ST--PVIMIGPGTGLAPFRGFLQERLAL-K----
C.elegans        -------SKSPVFVRKSTMRLPHRT-T--TQVIMIGPGTGFAPFRGFLQDRQFH-K----
C.jacchus        -------ALVPMFVRKSQFRLPFKA-T--TPVIMVGPGTGVAPFMGFIQERAWL-Q----
C.neoformans     HYVKENVYKVPIHVRRSTFRLPTSP-K--VPIIMIGPGTGVAPFRGFVQERIALARKAID
P.tetraurelia    ------------------------------------------------------------
D.rerio          -------PTVPMYVRRSQFRLPFKP-S--NPVIMIGPGTGIAPFMGFIQERAWR-K----
D.discoideum     --------KVPLFVRESHFKLPSAA-TEQKPVIMVGPGTGLAPFRGFLQELQHR-N----
D.melanogaster   -------VKVPVFIRKSQFRLPTKP-E--TPIIMVGPGTGLAPFRGFIQERQFL-R----
E.siliculosus    -------SKCRVFVRESTFRLPADS-SI--PIIMIGPGTGVAPMRALLQERAWQ-K----
G.gallus         -------SLVPMYVRKSQFRLPFKP-S--TPVIMIGPGTGIAPFIGFIQERAWL-K----
G.gorilla        -------ALVPMFVRKSQFRLPFKA-T--TPVIMVGPGTGVAPFIGFIQERAWL-R----
H.sapiens        -------ALVPMFVRKSQFRLPFKA-T--TPVIMVGPGTGVAPFIGFIQERAWL-R----
I.tridecemlinea  -------ALVPMFVRKSQFRLPFKP-T--TPVIMVGPGTGVAPFIGFIQERAWL-R----
M.mulatta        -------ALVPMFVRKSQFRLPFKA-T--TPVIMVGPGTGVAPFIGFIQERAWL-R----
M.gallopavo      -------SLVPMYVRKSQFRLPFKP-S--TPVIMIGPGTGIAPFIGFIQERAWL-K----
M.musculus       -------ALVPMFVRKSQFRLPFKP-T--TPVIMVGPGTGVAPFMGFIQERAWL-R----
O.sativa         -------SWAPIFVRQSNFKLPTDP-TV--PIIMIGPGTGLAPFRGFLQERLAL-K----
O.latipes        -------STVPMFIRKSQFRLPFKA-T--NPVIMIGPGTGIAPFMGFIQERGWL-K----
P.troglodytes    -------ALVPMFVRKSQFRLPFKA-T--TPVIMVGPGTGVAPFIGFIQERAWL-R----
P.falciparum     --------IVKFNIKPSKFVLPENIQS--SHIIMIATGAGIAPFKAFLSEFIYYDQQIVK
P.abelii         -------ALVPMFVRKSQFRLPFKA-T--TPVIMVGPGTGVAPFIGFIQERAWL-R----
S.cerevisiae     LF---ANYKLPVHVRRSNFRLPSNP-S--TPVIMIGPGTGVAPFRGFIRERVAFLESQKK
S.pombe          NW---T-GKIPMFVKKSTFRLAP-P-D--VPIIMVGPGTGVAPFRGFVMERANLASK---
S.purpuratus     -------FRVPIYVRKSQFRLPFKT-T--TPVIMVGPGTGLAPFRGFIQERDFYRK----
S.scrofa         -------ALLPMFVRKSQFRLPFKA-T--TPVIMVGPGTGVAPFIGFIQERAWL-Q----
T.rubripes       -------SMVPMYIRKSQFRLPFKA-T--NPVLMVGPGTGIAPFVGFIQERGWL-K----
X.tropicalis     -------SSVPMFVRKSQFRLPFKP-S--TPVIMIGPGTGIAPFMGFIQEREWL-K----
                                                                             


                       7210      7220      7230      7240      7250      7260
                 =========+=========+=========+=========+=========+=========+
A.carolinensis   KQ-G---KEVGETVLYYGCRHENEDYLYKEELANFLKEG--ALTQLHVAFSRDQ----AQ
A.gambiae        QE-G---KEIGQTTLYFGCRKRSEDYIYEDELEDYSKRG--IIN-LRVAFSRDQ----EK
A.thaliana       ED-G---EELGSSLLFFGCRNRQMDFIYEDELNNFVDQG--VISELIMAFSREG----AQ
B.distachyon     ES-G---VGLGTSILFFGCRNRNMDYIYEDELQNFLQEG--ALSELVVAYSREG----PT
C.elegans        NA-G---KEIGAMHLYYGCRHPDHDYIYKDELAKFQEDE--VLTHLVCAFSRAQ----EH
C.jacchus        QQ-G---KEVGETLLYYGCRRSDEDYLYREELAQFHKDG--TLTQLNVAFSREQ----AH
C.neoformans     KNGPDALKDWAPMYLFYGCRRADEDFLYREEWPKYEQELKGVF-RMKVAFSREMKKPDGG
P.tetraurelia    ------------------------------------------------------------
D.rerio          EQ-G---KDVGETILYFGCRHSNEDFLYQQELEEFERAG--VLTQLNVAFSRDQ----EQ
D.discoideum     HS-----Q-QQQSLLFFGCRSDTVDYIYREELEQYHQSS--VLGDLVVAFSRKT----SQ
D.melanogaster   DE-G---KTVGESILYFGCRKRSEDYIYESELEEWVKKG--TLN-LKAAFSRDQ----GK
E.siliculosus    EQ-G---LSVGRNVLYFGCRCRDQDYIYRDELEAYQADG--TLDSLRLAFSREG----SS
G.gallus         EQ-G---KEVGETVLYYGCRREREDYLYRQELARFKQEG--VLTQLNVAFSRDQ----AE
G.gorilla        QQ-G---KEVGETLLYYGCRRSDEDYLYREELVQFHRDG--ALTQLNVAFSREQ----SH
H.sapiens        QQ-G---KEVGETLLYYGCRRSDEDYLYREELAQFHRDG--ALTQLNVAFSREQ----SH
I.tridecemlinea  QQ-G---KEVGETLLYYGCRRADEDYLYREELAQFHKDG--TLTQLNVAFSREQ----AH
M.mulatta        QQ-G---KEVGETLLYYGCRRSDEDYLYREELAQFHRDG--ALTQLNVAFSREQ----SH
M.gallopavo      EQ-G---KEVGETVLYYGCRREQEDYLYRQELARFKQEG--VLTQLNVAFSRDQ----AE
M.musculus       EQ-G---KEVGETLLYYGCRRSDEDYLYREELARFHKDG--ALTQLNVAFSREQ----AH
O.sativa         ET-G---VELGHAVLFFGCRNRKMDFIYEDELNNFVETG--ALSELIVAFSREG----PS
O.latipes        EQ-G---KEVGETVLYFGCRHKNEDYIYQEELEDAEKSG--VLTQLNVAFSRDQ----DH
P.troglodytes    QQ-G---KEVGETLLYYGCRRSDEDYLYREELAQFHRDG--ALTQLNVAFSREQ----SH
P.falciparum     DN-F---VRKGKRILFYGCRKREVDFLYEMEIMDALDKK--HIDETYFAFSRDQ----ES
P.abelii         QQ-G---KEVGETLLYYGCRRSDEDYLYREELAQFHRDG--ALTQLNVAFSREQ----SH
S.cerevisiae     -GG-N-NVSLGKHILFYGSRNTD-DFLYQDEWPEYAKKLDGSF-EMVVAHSRL---PNTK
S.pombe          --G----VKVAKTLLFYGCQYSDKDFLYKEEWQQYKDVLKDSF-ELITAFSREQ----DH
S.purpuratus     DE-T---KKVGDSILFFGCRKSTEDYIYKDELDEYLKNG--TLTNVHVAFSRET----EE
S.scrofa         EQ-G---KEVGETLLYYGCRRSDEDYLYREELAQFHAKG--ALTRLSVAFSREQ----PQ
T.rubripes       EQ-G---KEVGETVMYFGCRHKNEDYLYQEELEEAEKNA--ALTQLNVAFSRDQ----EQ
X.tropicalis     QQ-G---KDVGETVLYYGCRHEHEDFLYINELKRYHKEG--VLTQLNVAFSRDQ----AH
                                                                             


                       7270      7280      7290      7300      7310      7320
                 =========+=========+=========+=========+=========+=========+
A.carolinensis   KVYVQHLLKKNKESVWKLI-HEDNGHIYVCGDARNMARDVQNTFYEIVAEFG-KMDQPQA
A.gambiae        KVYVTHLLEQDSDLIWSVI-GENKGHFYICGDAKNMATDVRNILLKVIRSKG-GLSETEA
A.thaliana       KEYVQHKMMEKAAQVWDLI-KE-EGYLYVCGDAKGMARDVHRTLHTIVQEQE-GVSSSEA
B.distachyon     KEYVQHKMVEKATEIWNII-SQ-GGYVYVCGDAKGMARDVHRVLHTIVQEQG-SLDNSKT
C.elegans        KIYVQDRLWETRDRIWDAI-NV-GAHVYICGDARNMARDVQATLQKIFREIG-GKSETEA
C.jacchus        KVYVQHLLKRDREHLWKLI-EG-GAHIYVCGDARNMARDVQNTFCDIVAELG-AMEHAQA
C.neoformans     KVYVQDLIHDLASELAPLILEK-RAYIYICGDAKTMSKAVEERLMEMLGAAKGGSAAVEG
P.tetraurelia    ------------------------------------------------------------
D.rerio          KVYVQHLLKKNKQQLWKLI-HTDNAHIYVCGDARNMARDVHAAFSEIAEQEG-RLTHTQA
D.discoideum     KVYVQNKLLEHKEKVWELL-NK-GAYFYVCGDGRNMSKAVQQALLSIIKEFG-SKDDNSA
D.melanogaster   KVYVQHLLEQDADLIWNVI-GENKGHFYICGDAKNMAVDVRNILVKILSTKG-NMSEADA
E.siliculosus    KVYVQHLLREDAAEMWGLL-EG-GAYVYVCGGTK-MGTDVHSEFNHIAQSCG-LMGVEES
G.gallus         KVYVQHLLKKNKEHIWKLV-NDGNAHIYVCGDARNMARDVQNTFYEIVSEYG-NMNQSQA
G.gorilla        KVYVQHLLKRDREHLWKLI-EG-GAHIYVCGDARNMARDVQNTFYDIVAELG-AMEHTQA
H.sapiens        KVYVQHLLKQDREHLWKLI-EG-GAHIYVCGDARNMARDVQNTFYDIVAELG-AMEHAQA
I.tridecemlinea  KVYVQHLLKRDAEHLWELI-HNGGAHIYVCGDARNMARDVQNTFCDIVAELG-AMEHAQA
M.mulatta        KVYVQHLLKRDREHLWKLI-EG-GAHIYVCGDARNMARDVQNTFYDIVAELG-AMEHAQA
M.gallopavo      KVYVQHLIKKNKEHIWKLV-NDGNAHIYVCGDARNMARDVQNTFYEIVAEYG-NMNQSQA
M.musculus       KVYVQHLLKRDKEHLWKLI-HEGGAHIYVCGDARNMAKDVQNTFYDIVAEFG-PMEHTQA
O.sativa         KEYVQHKMAEKAPEIWSII-SQ-GGYIYVCGDAKGMARDVHRTLHTIVQEQG-SLDNSNT
O.latipes        KVYVQHLLKKNKEHVWKLI-HTDNAHLYVCG-----------------------------
P.troglodytes    KVYVQHLLKRDREHLWKLI-EG-GAHIYVCGDARNMARDVQNTFYDIVAELG-AMEHAQA
P.falciparum     KIYVQDLILQKKELVWNLL-QK-GAYIYVCGNS-NMSKDVNKTINSLPLHFK-QNDK---
P.abelii         KVYVQHLLKRDREHLWKLI-EG-GAHIYVCGDARNMARDVQNTFYDIVAELG-AMEHAQA
S.cerevisiae     KVYVQDKLKDYEDQVFEMI-NN-GAFIYVCGDAKGMAKGVSTALVGILSRGK-SITTDEA
S.pombe          KIYVQHRLLEHSDTIAKLV-EE-GAAFYICGDADHMAKDVVNALASILTTV-----DVDG
S.purpuratus     KLYVQHLMKRCQKQIWGML-EK-GSHIYVCGDARFMAPDVQRTIREIICQEG-GKTQTEA
S.scrofa         KVYVQHLLKRDKEHLWKLI-HDGGAHIYICGDARNMARDVQNTFCDIVAEQG-PMEHAQA
T.rubripes       KVYVQHLLKANKEHVWKLI-HSENAHIYICGDARNMAKDVQLAFYEIAEEQG-EMTRSQA
X.tropicalis     KVYVQHLLKNNKEMVWKLI-HEDNAHIYVCGDARNMARDVQNIFYDIVEEYG-KLDHAQA
                                                                             


                       7330      7340      7350      7360      7370      7380
                 =========+=========+=========+=========+=========+=========+
A.carolinensis   VDYIKKLMTKGRYSLDIWS------M-R------------------WL------------
A.gambiae        QQYIKKMEAQKRYSADVWS-----------------------------------------
A.thaliana       EAIVKKLQTEGRYLRDVW--------MSLL----------------LR------------
B.distachyon     ESYVKSLQMEGRYLRDVW-------MASS-----------------FL------------
C.elegans        VAYFKDMEKTKRYQADVWS-----------------------------------------
C.jacchus        VDYIKKLMTKGRYSLDVWS------M-Y------------------FP------------
C.neoformans     AKELKMLKERNRLMSDVWS------MA-LR----------------AS------------
P.tetraurelia    ------------------------------------------------------------
D.rerio          LDYFKKLMTKGRYSQDVWSMQITMAN-K------------------CR------------
D.discoideum     QQFIDDMSSHGRYLQDVWF-------------------------MNRV------------
D.melanogaster   VQYIKKMEAQKRYSADVWS------M-SGRYRGGGGGRFGGRGGGGYA------------
E.siliculosus    KEYMKGLHDAGRFVQELWS------M-M------------------CSTSAPPADVEVSA
G.gallus         VDYVKKLMTKGRYSLDVWS------M-A------------------PG------------
G.gorilla        VDYIKKLMTKGRYSLDVWS------M-Y------------------FP------------
H.sapiens        VDYIKKLMTKGRYSLDVWS------M-Y------------------FP------------
I.tridecemlinea  VDYIKKLMTKGRYSLDVWS------M-Y------------------FL------------
M.mulatta        VDYVKKLMTKGRYSLDVWS------M-T------------------FP------------
M.gallopavo      VDYVKKLMTKGRYSLDVWS------M-D------------------S-------------
M.musculus       VDYVKKLMTKGRYSLDVWS------M-Y------------------LG------------
O.sativa         ESYVKSLQMEGRYLRDVW------------------------------------------
O.latipes        -----------------------MAT-R------------------CR------------
P.troglodytes    VDYIKKLMTKGRYSLDVWS------M-Y------------------FP------------
P.falciparum     -KFTKKLKKSGRYIYEIW-------M-I------------------NCLLQLNGRLNYLK
P.abelii         VDYIKKLMTKGRYSLDVWS------M-Y------------------FP------------
S.cerevisiae     TELIKMLKTSGRYQEDVW--------------------------MLAR------------
S.pombe          MKAVKALRDDNRFFEDTW------------------------------------------
S.purpuratus     EDYIKKMQSKGRYSCDVWS-----------------------------------------
S.scrofa         VDYVKKLMTKGRYSLDVWS---------------------------MF------------
T.rubripes       TDYIKKLMTKGRYSQDVWS----MTS-S------------------SK------------
X.tropicalis     VDYIKKLMTKGRYSQDVWS---MALR-S------------------NL------------
                                                                             


                       7390      7400      7410      7420      7430      7440
                 =========+=========+=========+=========+=========+=========+
A.carolinensis   ---------------------------------------------R-------------C
A.gambiae        ------------------------------------------------------------
A.thaliana       ---------------------------------------------T-LPLRPTRFLSAT-
B.distachyon     ---------------------------------------------RASPLSLLS-RLKPV
C.elegans        ------------------------------------------------------------
C.jacchus        ---------------------------------------------S-------------W
C.neoformans     ---------------------------------------------R-T-------LTVL-
P.tetraurelia    ------------------------------------------------------------
D.rerio          ---------------------------------------------L-------------F
D.discoideum     ---------------------------------------------I-------------L
D.melanogaster   ------DGGGYNDFDNYRGGGGPGFNDNFGPGPGPNPFNMGGPNIS-------------G
E.siliculosus    EGLEIGASIKAKGDAI------RDLKAGGVSKDELKPHIEE------------------L
G.gallus         -----------------------------------------------------------W
G.gorilla        ---------------------------------------------S-------------W
H.sapiens        ---------------------------------------------S-------------W
I.tridecemlinea  ---------------------------------------------S-------------W
M.mulatta        ---------------------------------------------S-------------W
M.gallopavo      ------------------------------------------------------------
M.musculus       ---------------------------------------------F-------------W
O.sativa         ------------------------------------------------------------
O.latipes        ---------------------------------------------L-------------L
P.troglodytes    ---------------------------------------------S-------------W
P.falciparum     KYLLLINLFNLKNKDINFILVRKGLFVCGLRKME--------------------------
P.abelii         ---------------------------------------------S-------------W
S.cerevisiae     ---------------------------------------------S--------------
S.pombe          ------------------------------------------------------------
S.purpuratus     -----------------------------------------------------------M
S.scrofa         ---------------------------------------------C-------------W
T.rubripes       ---------------------------------------------F-------------L
X.tropicalis     ---------------------------------------------K-------------I
                                                                             


                       7450      7460      7470      7480      7490      7500
                 =========+=========+=========+=========+=========+=========+
A.carolinensis   --L--C------------G----------P------------------------------
A.gambiae        ------------------------------------------------------------
A.thaliana       ---------------AISI----------S------------------------------
B.distachyon     --N--RRPPASHLRRLLLL----------S------------------------------
C.elegans        ------------------------------------------------------------
C.jacchus        --L--S------------Q----------L------------------------------
C.neoformans     ---------------SASS----------R------------------------------
P.tetraurelia    ------------------------------------------------------------
D.rerio          --F--V------------R----------S------------------------------
D.discoideum     --K-DS------------K----------IFLNVLNK-----------------------
D.melanogaster   --NELM------------QLMSAMASNFNM------NSQPPQPMRQSCGELRNHHCGLFV
E.siliculosus    LEL--K------------Q----------L-YVK--ATGTAFGPPPSDKKAKKGKSKAPA
G.gallus         --A--A------------G----------R------------------------------
G.gorilla        --L--S------------Q----------L------------------------------
H.sapiens        --L--S------------Q----------L------------------------------
I.tridecemlinea  --L--S------------Q----------L------------------------------
M.mulatta        --L--S------------Q----------L------------------------------
M.gallopavo      --S--A------------G----------S------------------------------
M.musculus       --L--S------------R----------L------------------------------
O.sativa         ------------------------------------------------------------
O.latipes        --L--R------------R----------L------------------------------
P.troglodytes    --L--S------------Q----------L------------------------------
P.falciparum     -----K------------D----------D-VVSSTPAATEEAVMNDKKKEKKAKKLAEK
P.abelii         --L--S------------Q----------L------------------------------
S.cerevisiae     ------------------R----------VCLQTITR-----------------------
S.pombe          ------------------------------------------------------------
S.purpuratus     --L--R------------S----------L------------------------------
S.scrofa         --L--S------------R----------L------------------------------
T.rubripes       --L--H------------R----------L------------------------------
X.tropicalis     --L--T------------T----------C------------------------------
                                                                             


                       7510      7520      7530      7540      7550      7560
                 =========+=========+=========+=========+=========+=========+
A.carolinensis   -----------------------------------WLRRG--------------------
A.gambiae        ------------------------------------------------------------
A.thaliana       ------------------------------------NATN--FFV--V---PKRT-NP--
B.distachyon     ------------------------------------GSTS-------V---PSCS-PP--
C.elegans        ------------------------------------------------------------
C.jacchus        -----------------------------------YRGLS--RPI--R------------
C.neoformans     ------------------------------------STTT---RT------IPRVWGA--
P.tetraurelia    ------------------------------------------------------------
D.rerio          -----------------------------------AQQML--RCV--Y---SRRSVPSIN
D.discoideum     ----------------------------------PIIKNK--NCL--S---LLNITTSST
D.melanogaster   ELSGRLIKKRVNRFAELRDRNGGACQLVVLEDKHPRVARR----M--NNMPENTTLTIVG
E.siliculosus    S----GGGGGGGQPKQMDTRKADKAKKA-------EQARLAAEARDLE---ILKGLEP--
G.gallus         -----------------------------------SLTFP--NML--R---LV-------
G.gorilla        -----------------------------------YRGLS--RPI--R------------
H.sapiens        -----------------------------------YRGLS--RPI--R------------
I.tridecemlinea  -----------------------------------CRGLS--RPI--G------------
M.mulatta        -----------------------------------CRGLS--RPI--R------------
M.gallopavo      -----------------------------------FLCF---------------------
M.musculus       -----------------------------------CRGLS--RPI--G------------
O.sativa         ------------------------------------------------------------
O.latipes        -----------------------------------FGA----RSV--W---YHSSCGG--
P.troglodytes    -----------------------------------YRGLS--RPI--R------------
P.falciparum     E----LKLAKKLERENLKNEAAKVLDYV-------CEDINKDNYGYIK---VSTLQKY--
P.abelii         -----------------------------------YRGLS--RPI--R------------
S.cerevisiae     ----------------------------------R-------------------------
S.pombe          ------------------------------------------------------------
S.purpuratus     -----------------------------------LKRRP--------------------
S.scrofa         -----------------------------------FRGLS--RPT--G------------
T.rubripes       -----------------------------------VVACY--RNL--S---SHLRCSARP
X.tropicalis     -----------------------------------MLAVR--RPV--V---LE-------
                                                                             


                       7570      7580      7590      7600      7610      7620
                 =========+=========+=========+=========+=========+=========+
A.carolinensis   ----------------LCAA--L---------------------------KAQHRPLSGS
A.gambiae        ------------------------------------------------------------
A.thaliana       LPGTRRT-FS----S-SP-V--A---------------------------AASGDVVVKP
B.distachyon     TLRTLATDAA----T----APEE-----------------------------AALPAAEA
C.elegans        ----------------------M---------------------------ISARNVTAIC
C.jacchus        ---------R----TTQPIW--G---------------------------SLSRSLVLSS
C.neoformans     AASRVLV-RGNATATLTP---------------------------------REIKPFQEQ
P.tetraurelia    ------------------------------------------------------------
D.rerio          --HIYSS-RP----LISPNL--L---------------------------ECAYSISRSI
D.discoideum     TSIIKNQ-QI----NQFNKR-----------------------------NFTNTINNNKN
D.melanogaster   --L-VM-----------RRP-HNSCNQTMPTGEIEVEVQDILNIHFPAGGTKRAGDKRTY
E.siliculosus    VLYPSKE-Q-----LEAGVGE-----------------------------PSQRIGDYSL
G.gallus         ---------R----RVLFQR--G---------------------------TERARLSSGW
G.gorilla        ---------R----TTQPIW--G---------------------------SLYRSLLQSS
H.sapiens        ---------R----TTQPIW--G---------------------------SLYRSLLQSS
I.tridecemlinea  ---------R----TAQPTW--G---------------------------SFSRSLALSS
M.mulatta        ---------R----TTQPIW--G---------------------------SLYRSLVQSS
M.gallopavo      ------------------------------------------------------------
M.musculus       ---------K----TMRPIW--G---------------------------SLSRNLALSS
O.sativa         ------------------------------------------------------------
O.latipes        ---LLLW-RP----QLR--S--S---------------------------SCSTTRLYTP
P.troglodytes    ---------R----TTQPIW--G---------------------------SVYRSLLQSS
P.falciparum     ADSIMEL-Y-----NLEDIYN-----------------------------FFVKSEDCEN
P.abelii         ---------R----TTQPIW--G---------------------------SLYRSLLQSS
S.cerevisiae     --------------------------------------------------------LADF
S.pombe          ------------------------------------------------------------
S.purpuratus     -------------------L--A---------------------------AVTKTYCNVR
S.scrofa         ---------K----TTQPFW--G---------------------------FLSRSLILTS
T.rubripes       CPEIQPC-TA----QLR--L--L---------------------------SSSP-SLCMV
X.tropicalis     ---------N----SCSTHR--K---------------------------YIRTYTPNLT
                                                                             


                       7630      7640      7650      7660      7670      7680
                 =========+=========+=========+=========+=========+=========+
A.carolinensis   ------------------------------------------------------------
A.gambiae        ------------------------------------------------------------
A.thaliana       ------------------------------------------------------------
B.distachyon     ------------------------------------------------------------
C.elegans        ------------------------------------------------------------
C.jacchus        ------------------------------------------------------------
C.neoformans     ------------------------------------------------------------
P.tetraurelia    ------------------------------------------------------------
D.rerio          ------------------------------------------------------------
D.discoideum     ------------------------------------------------------------
D.melanogaster   STMVQQQSNLGITSTEYKIAKNENILKYFENRDLTCNDLRRDDVGKTVTLVGWIPSTKNN
E.siliculosus    ------------------------------------------------------------
G.gallus         ------------------------------------------------------------
G.gorilla        ------------------------------------------------------------
H.sapiens        ------------------------------------------------------------
I.tridecemlinea  ------------------------------------------------------------
M.mulatta        ------------------------------------------------------------
M.gallopavo      ------------------------------------------------------------
M.musculus       ------------------------------------------------------------
O.sativa         ------------------------------------------------------------
O.latipes        ------------------------------------------------------------
P.troglodytes    ------------------------------------------------------------
P.falciparum     ------------------------------------------------------------
P.abelii         ------------------------------------------------------------
S.cerevisiae     ------------------------------------------------------------
S.pombe          ------------------------------------------------------------
S.purpuratus     ------------------------------------------------------------
S.scrofa         ------------------------------------------------------------
T.rubripes       ------------------------------------------------------------
X.tropicalis     ------------------------------------------------------------
                                                                             


                       7690      7700      7710      7720      7730      7740
                 =========+=========+=========+=========+=========+=========+
A.carolinensis   ------------------------------------------------------------
A.gambiae        ------------------------------------------------------------
A.thaliana       ------------------------------------------------------------
B.distachyon     ------------------------------------------------------------
C.elegans        ------------------------------------------------------------
C.jacchus        ------------------------------------------------------------
C.neoformans     ------------------------------------------------------------
P.tetraurelia    ------------------------------------------------------------
D.rerio          ------------------------------------------------------------
D.discoideum     ------------------------------------------------------------
D.melanogaster   KFLQLKDGYGQTQLMIEDQSLSDTFLSTPEQTVIQIVGKVLGRPKANVNLKYDTGEVEVS
E.siliculosus    ------------------------------------------------------------
G.gallus         ------------------------------------------------------------
G.gorilla        ------------------------------------------------------------
H.sapiens        ------------------------------------------------------------
I.tridecemlinea  ------------------------------------------------------------
M.mulatta        ------------------------------------------------------------
M.gallopavo      ------------------------------------------------------------
M.musculus       ------------------------------------------------------------
O.sativa         ------------------------------------------------------------
O.latipes        ------------------------------------------------------------
P.troglodytes    ------------------------------------------------------------
P.falciparum     ------------------------------------------------------------
P.abelii         ------------------------------------------------------------
S.cerevisiae     ------------------------------------------------------------
S.pombe          ------------------------------------------------------------
S.purpuratus     ------------------------------------------------------------
S.scrofa         ------------------------------------------------------------
T.rubripes       ------------------------------------------------------------
X.tropicalis     ------------------------------------------------------------
                                                                             


                       7750      7760      7770      7780      7790      7800
                 =========+=========+=========+=========+=========+=========+
A.carolinensis   ------------------------------------------------------------
A.gambiae        ------------------------------------------------------------
A.thaliana       ------------------------------------------------------------
B.distachyon     ------------------------------------------------------------
C.elegans        ------------------------------------------------------------
C.jacchus        ------------------------------------------------------------
C.neoformans     ------------------------------------------------------------
P.tetraurelia    ------------------------------------------------------------
D.rerio          ------------------------------------------------------------
D.discoideum     ------------------------------------------------------------
D.melanogaster   VTSVKVLNPDDPYDGPIKAKEKKQKFSIDDLEAEEAAAEASANLTSNNGESTEDSAEAST
E.siliculosus    ------------------------------------------------------------
G.gallus         ------------------------------------------------------------
G.gorilla        ------------------------------------------------------------
H.sapiens        ------------------------------------------------------------
I.tridecemlinea  ------------------------------------------------------------
M.mulatta        ------------------------------------------------------------
M.gallopavo      ------------------------------------------------------------
M.musculus       ------------------------------------------------------------
O.sativa         ------------------------------------------------------------
O.latipes        ------------------------------------------------------------
P.troglodytes    ------------------------------------------------------------
P.falciparum     ------------------------------------------------------------
P.abelii         ------------------------------------------------------------
S.cerevisiae     ------------------------------------------------------------
S.pombe          ------------------------------------------------------------
S.purpuratus     ------------------------------------------------------------
S.scrofa         ------------------------------------------------------------
T.rubripes       ------------------------------------------------------------
X.tropicalis     ------------------------------------------------------------
                                                                             


                       7810      7820      7830      7840      7850      7860
                 =========+=========+=========+=========+=========+=========+
A.carolinensis   -V---------ALEADVNSFVSRTK--TCGELRSVHIGQEVTLCGWIQY--QRQ-G---Q
A.gambiae        --------APQKSSSSTNQFTTRSH--NCGELRLSHVGQKVTLCGWLEF--SRM-N---K
A.thaliana       -V---------PSPPSVLRWVSRTE--LCGELSVNDVGKRVHLCGWVAL--HRV-HGGLT
B.distachyon     -K---------LERMQPLQWPRRDA--LCGDLGAGDAGRRVRLCGWVAL--RRT-HAGLT
C.elegans        ----------CRRILSTSSYTVRTH--ICDELSTSNKNEKVSVMGWLSH--KRM-D---R
C.jacchus        -Q---------RRNPEFSSFVVRTN--TCGELRSSHLGQEVTLCGWIQY--RRQ-N---T
C.neoformans     -V---------TLKAHYGPRPKITH--DIADLSPLLVDQKVVIAGWLFS--QRRASDSLH
P.tetraurelia    ------------------------------------------------------------
D.rerio          -S---------THTSGQSSFSQRSH--TCGELCSSHIDKEVTLCGWVQY--LRQ-D---L
D.discoideum     ----------ENINNKILNIIERSHSC--GEITSKDIGKEVIIYGWINS--LRN-LGDNV
D.melanogaster   TANASLIAEQRAKVADTNKFADRTH--NCGELTSNDINEKVVICGWLEF--QRM-N---K
E.siliculosus    ----------TRSEHETGRVFSSVK--DLGAPGGVPVGREVWIRGRVHQ--VRG-KGGSA
G.gallus         -V---------HRAPDVNSFAARTN--TCGELRAAHVGQEVTLCGWIQY--QRQ-G---L
G.gorilla        -Q---------RRIPEFSSFVVRTN--TCGELRSSHLGQEVTLCGWIQY--RRQ-N---T
H.sapiens        -Q---------RRIPEFSSFVVRTN--TCGELRSSHLGQEVTLCGWIQY--RRQ-N---T
I.tridecemlinea  -Q---------RRIPEFNSFVARTN--TCGELRSSHLGQEVTLCGWIQY--RRQ-N---I
M.mulatta        -Q---------RRNPEFSSFAVRTN--TCGELRSSHLGQEVTLCGWIQY--RRQ-N---T
M.gallopavo      -----------FLIPDVNSFAARTN--TCGELRAAHIGQKVTLCGWIQY--QRQ-G---L
M.musculus       -Q---------R-IPEFSSFVARTN--TCGELRSSHLGQEVTLCGWIQY--RRQ-N---T
O.sativa         ------------------------------------------------------------
O.latipes        -P---------PASTGPSSLSLRSH--TCGELNVDHVGKRVTLCGWVQY--LRQ-D---L
P.troglodytes    -Q---------RRIPEFSSFVVRTN--TCGELRSSHLGQEVTLCGWIQY--RRQ-N---T
P.falciparum     ----------EKREICVDKNVGDVK--DAYNEENLLGKKKIWVRGRIHD--IRS-KGSIA
P.abelii         -Q---------RRIPEFSSFVVRTN--ACGELRSSHLGQEVTLCGWIQY--RRQ-N---T
S.cerevisiae     ----------PEANAIKKKFLFRKDTSTIKQLKGLSSGQKIVLNGWIEQKPKRV-GKNLI
S.pombe          ------------------MVLSRLPAC----L-LPLVGTKVSIQGWLVATSRQV-SKSIS
S.purpuratus     -L---------SSTSGNTSLTERSH--NCSELRCDHVGEKVTLCGWLQH--RRF-D---F
S.scrofa         -Q---------RSIPELSSFVARTN--TCGELRSSHLGQEVTLCGWIQY--RRQ-N---I
T.rubripes       -H---------PQNTGPNSLSFRSH--SCGELRSDHVGEKVTLCGWVQY--LRQ-E---L
X.tropicalis     -V---------QRTPDLNSFVARTH--TCGELCSAHVGQEVALYGWLQY--QRH-D---L
                                                                             


                       7870      7880      7890      7900      7910      7920
                 =========+=========+=========+=========+=========+=========+
A.carolinensis   FVILR-DFQG-LTQIVIPQ-D----A---------------AAS-HVKKTLCDVPVESVV
A.gambiae        FFTLR-DGYG-TVQALLPE-A----V---------------S----EGFSLDGLAFESIL
A.thaliana       FLNLR-DHTG-IVQVRTLP-D------E-------------F-P-EAHGLINDMRLEYVV
B.distachyon     FLTLR-DSSG-MVQVATLP--------E-------------Y-P-EVYAVVNKLRVESVV
C.elegans        FFVLR-DAYG-SVQAKISA-S----------------------S-KLQSLLKDIPYESVV
C.jacchus        FLVLR-DFHG-LVQVIIPQ-D----E---------------SAA-SVRKILCEAPMESVV
C.neoformans     FFTLRSPSSS-SAVQLVSR-D----------------------K-DVSKDMMEYPLESVV
P.tetraurelia    ------------------------------------------------------------
D.rerio          FVILR-DFSG-LVQILIPQ-D----E---------------SKS-ELKKSLLALTVESVI
D.discoideum     FLVIR-DGHG-KVQCYVDL-K----Q-QCILKSSVPNIDINERN-SIEENIKLFKLESIV
D.melanogaster   FFILR-DAYG-QTQVLLSP-K----T---------------YGL--EEYAETGVPIESIV
E.siliculosus    FLVVR-QDTASTVQAVHFK-DKENPE---------------DSR-RMIKFVGTLPLESIV
G.gallus         FLVLR-DFQG-LTQIIIPQ-D----E---------------AHS-HVRKLLSNAPVESVV
G.gorilla        FLVLR-DFDG-LVQVIIPQ-D----E---------------SAA-SVKKILCEAPVESVV
H.sapiens        FLVLR-DFDG-LVQVIIPQ-D----E---------------SAA-SVKKILCEAPVESVV
I.tridecemlinea  FLVLR-DCHG-LVQVVIPQ-D----E---------------SAA-SVKKILCEAPLESVV
M.mulatta        FLVLR-DFHG-LVQVVIPQ-D----E---------------SAA-SVRKILCEAPVESVV
M.gallopavo      FLVLR-DFQG-LTQIIIPQ-D----E---------------AHS-HVRKLLCNAPVESVV
M.musculus       FLVLR-DCHG-LVQILIPQ-D----E---------------SAA-SVRRILCEAPVESVV
O.sativa         ------------------------------------------------------------
O.latipes        FVILR-DFSG-LTQILVPQ-E----E---------------SAS-KVKADLCDLTVESVI
P.troglodytes    FLVLR-DFDG-LVQVIIPQ-D----E---------------SAA-SVKKILCEAPVESVV
P.falciparum     FIILR-HKLYSL-QCILDIKNNNND------------------K-NMMKWVSNLSLECIV
P.abelii         FLVLR-DFDG-LVQVIIPQ-D----E---------------SAA-SVKKILCDAPVESVV
S.cerevisiae     FGLLR-DSNG-DIIQLVDN-K---------------------------SLLKGFTLEDVV
S.pombe          FHQLR-DTHG-TILQLLST-DKIILQQKREPLVSSTDFSQQKST-SVMRTLSSIPPESVV
S.purpuratus     MFTLR-DSFG-TVQIVIPK-E----E---------------LGGKELVDTLTAAYQESVI
S.scrofa         FLVLR-DFHG-LVQVVIPQ-D----E---------------SAA-SVRRILCEAPVESVV
T.rubripes       FVILR-DFSG-MTQVVIPQ-K----E---------------SAW-HLKSTLCDLTVESVI
X.tropicalis     FVVLR-DFHG-LTQVILPQ-D----E---------------KCS-QLRKMLSEASLESVV
                                                                             


                       7930      7940      7950      7960      7970      7980
                 =========+=========+=========+=========+=========+=========+
A.carolinensis   KVTGTVVSRPPGQTNPN-MPTGDIEVKVETAEVLNSCKK-LPFEMK----DFT-------
A.gambiae        RVTGTVQPRPEGQANRT-NPTGEIEIHVSGLEVLNEAKKRLPINVK----DYN-------
A.thaliana       LVEGTVRSRPNESVNKK-MKTGFVEVVAEHVEILNPVRTKLPFLVTTADENKD-------
B.distachyon     SVEGVVRPRPTEAINTE-MKTGAIEVVADHILVLNSVTRSLPFPVTTADTVKE-------
C.elegans        RVDGIVVDRGDNR-NSK-MKTGDIEIDVEELTVLNKATS--NIPML----PDA-------
C.jacchus        QVSGTVISRPAGQENPK-MPTGEIEIKVKTAELLNACKK-LPFEIK----DFM-------
C.neoformans     LVQGTVKARKQKAKAIS-SAVDEIELEVESVTLLNPADQTLPFYPNR----PE-------
P.tetraurelia    ------------------------------------------------------------
D.rerio          MVKGRVIRRPEGQENKN-ISTGEIEVCAESIEVLNTSRK-LPFEIK----EFV-------
D.discoideum     SIKGKVIARPERMVNKN-MSTGEIEISVDQLQLLNNCVD-LPFTVE---HDST-------
D.melanogaster   RVEGTVIPRPAATINPK-MQTGHVEVEADKVVVLNPAKKNLPFEIR----KFN-------
E.siliculosus    DVKGLLVE-AN---VKS-CTQNNVEIKIDSVYAVSRASLFLPFSVE----DAARSEEEVE
G.gallus         RVTGIVSSRPPGQENPK-MPTGDIEVKAETAEVLNFCKK-LPFEIK----DFI-------
G.gorilla        QVSGTVISRPAGQENPK-MPTGEIEIKVKTAELLNACKK-LPFEIK----DFV-------
H.sapiens        QVSGTVISRPAGQENPK-MPTGEIEIKVKTAELLNACKK-LPFEIK----NFV-------
I.tridecemlinea  QVSGTVISRPPGQENPK-MPTGEIEIKVKTAELLNSCKK-LPFEIK----DFV-------
M.mulatta        QVSGTVISRPAGQENPK-MPTGEIEIKVKTAELLNACKK-LPFEIK----DFM-------
M.gallopavo      RVTGIVSSRPPGQENLK-MPTGDIEVKVESAEVLNFCKK-LPFEIK----DFI-------
M.musculus       RVSGTVISRPPGQENPK-MPTGEIEIKVKTAELLNACKK-LPFEIK----DFV-------
O.sativa         ------------------------------------------------------------
O.latipes        MVTGTVRKRPAGQENKE-MPTGEIEVLAENVEVFNVSKK-LPFEIK----EFV-------
P.troglodytes    QVSGTVISRPAGQENPK-MPTGEIEIKVKTAELLNACKK-LPFEIK----DFV-------
P.falciparum     DIYGEIKK-PE---IPIDSTNIKYEIHINKIFCLSKTMKELPFLLK----DANMKETNDE
P.abelii         QVSGTVISRPAGQENPK-MPTGEIEIKVKTAELLNACKK-LPFEIK----DFV-------
S.cerevisiae     QAVGILSLKRK---LSN-EDADEYEVQLEDITVLNASNK-KPAQMQ----DFK-------
S.pombe          QVTGKLQRRPE---HDR-RPGNEFELHVEDVKLLNVAKN-LQLFPG-----DE-------
S.purpuratus     QVKGHVQLRPDGLKNKE-MGTGDIEVVADSVELLNPCKEQLPVQLG----DFS-------
S.scrofa         QVSGTVISRPPGQENPK-MPTGEIEIKVKTAKLLNSCKK-LPFEIK----DFV-------
T.rubripes       KVTGTVRCRPEGQENKL-MPTGEIEILAESVEVYNLCQK-LPFEIK----DFV-------
X.tropicalis     VVKGTVIRRPPGQDNPR-MSTGEIEVLAKEAHILNTCKK-LPFEIK----DAV-------
                                                                             


                       7990      8000      8010      8020      8030      8040
                 =========+=========+=========+=========+=========+=========+
A.carolinensis   ----------KKN---EALRMQYRYLDMRSAQMQYNLRLRSQMVMKMREYLCNYYGFVDV
A.gambiae        ----------RAK---ENLRLEHRYIDLRNRDLQHNLRLRSQIIMRMRECMINDYGFVEV
A.thaliana       ----------LIK---EEIRLRFRCLDLRRQQMKNNIVLRHNVVKLIRRYLEDRHGFIEI
B.distachyon     ----------KFP---EEVRLRFRVLDLRRPQMQSNLRLRHKVIKHVRRYLEDRHDFVEI
C.elegans        ----------NAN---ERTRLKYRYIDLRSDKLQKSLRLRSEFVHNVRRFLVEKSGFVDV
C.jacchus        ----------KKT---EALRLQYRYLDLRSFQMQYNLRLRSQMVMKMREYLCNLHGFVDI
C.neoformans     ----------VAN---EDLRAQHRYLDLRRQDLANNLKTRSKVAHIIRNYLHD-QGFTEI
P.tetraurelia    ------------------------------------------------------------
D.rerio          ----------KKS---EALRMQYRYLDLRSATMQNNLRLRSQMVMKMREYLCNLHGFVDV
D.discoideum     ----------AVS---EELRLKYRYVDLRRDKVQSNIRLRSKVAMAARNYL-INQQFIEV
D.melanogaster   ----------RAG---ERLRLTHRYLDLRFNDMQHNLRLRSAVIMKMREYLINYLGFVEV
E.siliculosus    ASQETGRPFPRLG---QDLRLDNRWLDLRTPANNAIMRLQSGVCQLFRESLYA-QGFMEI
G.gallus         ----------KKS---EALRMQYRYLDLRSSQLQYNLRLRSQVVMRMREYLCNHHGFVDV
G.gorilla        ----------KKT---EALRLQYRYLDLRSFQMQYNLRLRSQMVMKMREYLCNLHGFVDI
H.sapiens        ----------KKT---EALRLQYRYLDLRSFQMQYNLRLRSQMVMKMREYLCNLHGFVDI
I.tridecemlinea  ----------KKT---EALRLQYRYLDLRSFQMQYNLRLRSQVVMKMREYLCNLHGFVDI
M.mulatta        ----------KKT---EALRLQYRYLDLRSFQMQYNLRLRSQMVMKMREYLCNLHASG--
M.gallopavo      ----------KKS---EALRMQYRYLDLRSSQLQYNLRLRSQVVMRMREYLCNHHGFVDV
M.musculus       ----------KKT---EALRLQYRYLDLRSFQMQYNLRLRSQMVMKMREYLCNLHGFVDI
O.sativa         ------------------------------------------------------------
O.latipes        ----------KKS---ESLRMQYRYLDLRSSRMQKNLRLRSQLVMKMREFLCNEHGFVDV
P.troglodytes    ----------KKT---EALRLQYRYLDLRSFQMQYNLRLRSQMVMKMREYLCNLHGFVDI
P.falciparum     ITI-------KVN---QDNRLNNRCFDLRTYANYSIFSLQSVICHIFRTFLLQ-HNFVEI
P.abelii         ----------KKT---EALRL---------------------------------------
S.cerevisiae     ----------LSAIYPPE----FRYLQLRNPKYQDFLKKRSSISKEIRNSF-NNFDFTEV
S.pombe          ----------KPG---MRIQLANRHIQLRAPKYNSYLRQRSRLAYQVHSFF-NDREFCEV
S.purpuratus     ----------KVS---EAVRMQFRYIDLRSKRMQRNLRLRSDVVMKMREFLWKKHGFVEV
S.scrofa         ----------KKT---ETLRLQYRYLDLRGAQMQYNLRLRSQMVMKMREYLCNLHGFVDI
T.rubripes       ----------KKS---EALRMQYRYLDLRSSQMQRNLRLRSQVVMKMREFLCNMHGFVDI
X.tropicalis     ----------KKT---EALRMQYRYLDIRSGQLQSNLRLRSKIVMKMREYLCNLHGFVDV
                                                                             


                       8050      8060      8070      8080      8090      8100
                 =========+=========+=========+=========+=========+=========+
A.carolinensis   ETPTLFKKT-PGGAKEFVVPSRE-A--------GKFYSLPQSPQQFKQLLMVGG-LDRYF
A.gambiae        ETPTLFRRT-PGGAQEFVVPSRK-P--------GHFYSLVQSPQQFKQMLMVGA-IDRYF
A.thaliana       ETPILSRST-PEGARDYLVPSRIQS--------GTFYALPQSPQLFKQMLMVSG-FDKYY
B.distachyon     ETPILSKST-PEGARDYLVPSRVQP--------GTCFALPQSPQLFKQMLMASG-FEKYF
C.elegans        ETPTLFRRT-PGGAAEFVVPAPS-PNQ------GLAYSLPQSPQQFKQLLMVGA-IDRYF
C.jacchus        ETPTLFKRT-PGGAKEFLVPSRE-P--------GKFYSLPQSPQQFKQLLMVGG-LDRYF
C.neoformans     ETPILLNSS-PEGAREFLVPTRS-PTFPRGVGQPTFYALPQSPQQPKQLLISSGAIPKYY
P.tetraurelia    ------------------------------------------------------------
D.rerio          ETPTLFKRT-PGGAKEFVVPSGD-P--------GKFYSLPQSPQQFKQLLMVAG-IDRYF
D.discoideum     ETPTLFRPT-PEGAREYLVPTR-HQ------GQ--FYSLPQSPQQYKQLLMVGG-IDRYF
D.melanogaster   ETPTLFRRT-PGGAQEFVVPTRK-A--------GHFYSLVQSPQQFKQMLMSGG-IDRYF
E.siliculosus    HTPKIIPGESEGGAGVFTTDYFG-----------KQACLAQSPQLYKQMAISAD-MGRVF
G.gallus         ETPTLFKRT-PGGAKEFLVPSRE-A--------GKFYSLPQSPQQFKQLLMIGG-LDRYF
G.gorilla        ETPTLFKRT-PG------------------------------------------------
H.sapiens        ETPTLFKRT-PGGAKEFLVPSRE-P--------GKFYSLPQSPQQFKQLLMVGG-LDRYF
I.tridecemlinea  ETPTLFKRT-PGGAKEFLVPSRE-P--------GKFYSLPQSPQQFKQLLMVGG-LDRYF
M.mulatta        ------QML-PEGAKEFLVPSRE-P--------GKFYSLPQSPQQFKQLLMVGG-LDRYF
M.gallopavo      ETPTLFKRT-PGGAKEFLVPSRE-P--------GKFYSLPQSPQQFKQLLMIGG-LDRYF
M.musculus       ETPTLFKRT-PGGAKEFLVPSRE-P--------GKFYSLPQSPQQFKQLLMVGG-LDRYF
O.sativa         ----------------------------------------------KQMLMVSG-FEKYY
O.latipes        ETPTLFKRT-PGGAKEFVVPSRE-P--------GLFYSLPQSPQQFKQLLMVAG-VDRYF
P.troglodytes    ETPTLFKRT-PGGAKEFLVPSRE-P--------GKFYSLPQSPQQFKQLLMVGG-LDRYF
P.falciparum     HTPKLLGESSEGGANAFKINYFN-----------QNGYLAQSPQLYKQMCINSG-FDKVF
P.abelii         ------------------------------------------------------------
S.cerevisiae     ETPMLFKAT-PEGAREFLVPTRTKRSD----GKPSFYALDQSPQQYKQLLMASG-VNKYY
S.pombe          ETPLLFKST-PEGAREFVVPSRLNP------GK--FYALPQSPQQYKQILMASG-IGNYY
S.purpuratus     ETPTLFRKT-PGGAQEFPVPCKQ-A--------GKFYSLPQSPQQFKQLLMVGG-IDRYF
S.scrofa         ETPTLFKRT-PGGAKEFVVPSRE-P--------GKFYSLPQSPQQFKQLLMVGG-LDRYF
T.rubripes       ETPTLFKRT-PGGAKEFVVPSRE-P--------GRFYCLPQSPQQFKQLLMVAG-IDRYF
X.tropicalis     ETPTLFKRT-PGGAKEFVVPTRE-P--------GKFYSLPQSPQQFKQLLIIGG-LDRYF
                                                                             


                       8110      8120      8130      8140      8150      8160
                 =========+=========+=========+=========+=========+=========+
A.carolinensis   QVARCYRDEGSKPDRQ-PEFTQVDIEMSFVDQ-PGIQALIEG----LLQYS---------
A.gambiae        QIARCYRDESTRPDRQ-PEFTQLDIELSFTDR-ESVIQLIEQ----VLTKS---------
A.thaliana       QIARCFRDEDLRADRQ-PEFTQLDMEMAFMPME-DMLKLNED----LIRKV---------
B.distachyon     QIARCFRDEDLRADRQ-PEFTQLDMEIAFTSME-DMLKLNEE----LMIHI---------
C.elegans        QIARCYRDEGSKGDRQ-PEFTQVDVEMSFTTQ-NGVMQLIED----MIISA---------
C.jacchus        QVARCYRDEGSRPDRQ-PEFTQIDIEMSFVDQ-TGIQSLIEG----LLQYS---------
C.neoformans     QIAKCFRDEDGRRDRQ-PEFTQIDLEIGFVSGA-AEQPRGEGEMRSTWAIGGQEVREVVE
P.tetraurelia    ------------------------------------------------------------
D.rerio          QLARCYRDEGSKPDRQ-PEFTQVDIEMSFVDQ-AGVMSLIEG----LVQFS---------
D.discoideum     QLARCYRDEDLRSDRQ-PEFTQIDMELSFVNTQ-MIYRIIEG----LVKTL---------
D.melanogaster   QVARCYRDEATRPDRQ-PEFTQLDIELSFTSR-DDIMQLIEE----TLRYS---------
E.siliculosus    EVGPVFRAENSNTRRHLTEFNGLDLEMSITDHYNEVIQVLHN----TFKSI---------
G.gallus         QVARCYRDEGSRPDRQ-PEFTQIDIEMSFVDQ-AGIQRLIEG----LLQYS---------
G.gorilla        ----------------------IDIEMSFVDQ-TGIQSLIEG----LLQYS---------
H.sapiens        QVARCYRDEGSRPDRQ-PEFTQIDIEMSFVDQ-TGIQSLIEG----LLQYS---------
I.tridecemlinea  QVARCYRDEGSRPDRQ-PEFTQIDIEMSFVDQ-TGIQSLIEG----LLQYS---------
M.mulatta        QVARCYRDEGSRPDRQ-PEFTQIDIEMSFVDQ-TGIQSLIEG----LLQYS---------
M.gallopavo      QVARCYRDEGSRPDRQ-PEFTQIDIEMSFVDQ-AGIQRLIEG----LLQYS---------
M.musculus       QVARCYRDEGSRPDRQ-PEFTQIDIEMSFVEQ-TGIQRLVEG----LLQYS---------
O.sativa         QIARCFRDEDLRADRQ-PEFTQLDMEIAFTSME-DMLKLNEE----LMRHI---------
O.latipes        QIARCYRDEGSKPDRQ-PEFTQVDIEMSFVDQ-EGIRTLVEG----MLQHS---------
P.troglodytes    QVARCYRDEGSRPDRQ-PEFTQIDIEMSFVDQ-TGIQSLIEG----LLQYS---------
P.falciparum     EVGPVFRAENSNTYRHLCEYVSLDIEMTYKFDYMENVHFYDS----MFKHI---------
P.abelii         ------------------------------------------------------------
S.cerevisiae     QMARCFRDEDLRADRQ-PEFTQVDMEMAFANSE-DVMKIIEK----TVSGV---------
S.pombe          QIARCFRDEDLRFDRQ-PEFTQIDLEMSFVDKPHEIMEVVED----LLVRL---------
S.purpuratus     QIARCYRDEGSRPDRQ-PEFTQVDLEMSFISK-EGIYHLIEE----MILFS---------
S.scrofa         QIARCYRDEGSRPDRQ-PEFTQIDIEMSFVDQ-TGIQSLIEG----LLQYS---------
T.rubripes       QIARCYRDEGSKPDRQ-PEFTQVDIEMSFVEQ-TGIMSLVEG----LLQHS---------
X.tropicalis     QIARCYRDEGSKPDRQ-PEFTQVDIEMSFVDQ-AGIQSLVEG----MLRFS---------
                                                                             


                       8170      8180      8190      8200      8210      8220
                 =========+=========+=========+=========+=========+=========+
A.carolinensis   ------WPL---EKGPIN--T----------------P--FPSISYNEALS---------
A.gambiae        ------WPY---SENPIR--A----------------P--FPRMTLAEAME---------
A.thaliana       ------FSE---IKG-IQ--L--------------PDP--FPRLTYADAMD---------
B.distachyon     ------FQE---VGD-IK--L--------------PKC--FPRLTYAEAMN---------
C.elegans        ------WPE---SLNHIKPKS----------------P--FPRIPYSDAMR---------
C.jacchus        ------WPN---DKNPVV--V----------------P--FPSMTFAEALA---------
C.neoformans     GMIKKIWKE---VKG-VD--L--------------EGW--FRVMPYEVAMD---------
P.tetraurelia    ------------------------------------------------------------
D.rerio          ------WPE---DKGQIN--V----------------P--FPTLTYEEAMR---------
D.discoideum     ------WK---------------------EAGFNIDY--EFPFYTYEQVLS---------
D.melanogaster   ------WPK---DFPRLQ--T----------------P--FRRITYEEAME---------
E.siliculosus    ------FSG---LEE-------------------RYKD--QL--------D---------
G.gallus         ------WPE---ERGPIM--T----------------P--FPTMTYDEALA---------
G.gorilla        ------WPN---DKDPVV--V----------------P--FPTMTFAEALA---------
H.sapiens        ------WPN---DKDPVV--V----------------P--FPTMTFAEVLA---------
I.tridecemlinea  ------WPS---DKDPLV--V----------------P--FPSMTFAEALA---------
M.mulatta        ------WPN---DKDPVV--V----------------P--FPTVTFTEALA---------
M.gallopavo      ------WPE---ERGPIT--T----------------P--FPSMTYEEALA---------
M.musculus       ------WPG---DKDPLV--T----------------P--FPSMTFAEALA---------
O.sativa         ------FQE---VGD-IK--L--------------PNP--FPRLTYTEAMN---------
O.latipes        ------WPA---EKGSIQ--V----------------P--FQTMTYEEAMR---------
P.troglodytes    ------WPN---DKDPVV--V----------------P--FPTMTFAEALA---------
P.falciparum     ------FKE---LTN-NE----------------KNKT--FI--------K---------
P.abelii         ------------------------------------------------------------
S.cerevisiae     ------WSKFSKKRGLLT--LDSKGTLVPAKKENGTV--SIFRMTYEQAMT---------
S.pombe          ------VS---------------------FAKG-ITLAKPFQHITYQHAID---------
S.purpuratus     ------WPD---SKPAPT--L----------------P--FPRMTYQDAMN---------
S.scrofa         ------WPS---DKDPLV--V----------------LS-FYALSVHTELRVRVKGGAVS
T.rubripes       ------WPA---ELGPIH--L----------------P--FNTMTFEEAMR---------
X.tropicalis     ------WPE---EKGPLQ--A----------------P--FPVMSYADAMC---------
                                                                             


                       8230      8240      8250      8260      8270      8280
                 =========+=========+=========+=========+=========+=========+
A.carolinensis   ---VYGTDKPDTRFA------MKITDVSDVLRN--VQM-EFLENHLSEPHGTIRAICIPQ
A.gambiae        ---RFGSDKPDTRFG------YELQNVTKTLGA--G-LTMPAGVRQQDFRVYGVVVRAEE
A.thaliana       ---RYGSDRPDTRFD------LELKDVSNVFTES--SF-RVFTEALE-SGGIIKVLCVPL
B.distachyon     ---CYGTDRPDLRFD------WELKDASDVFLGS--SF-KVFADTLE-NGGVIKALSVPG
C.elegans        ---LYGIDKPDMRIP------WQIDDVDND------IF-EFLQKDIDDDTWRSRILVCRG
C.jacchus        ---TYGTDKPDTRFG------MKIIDISDVFRN--TEI-GFLQDALSKPHGTVKAICIPE
C.neoformans     ---VYGSDKPDTRFD------MYTLPIGYYPTLS--DN-SLDKILLDQNPYTVEWMVTPA
P.tetraurelia    ------------------------------------------------------------
D.rerio          ---DYGVDKPDTRFG------MKLVDVSTAFQG--AQI-EFIKDALLEPEGCVQAICVPE
D.discoideum     ---TYGIDKPDTRY------DMKLVDITDCFNKDETNI-NLFKNALSQASNNFKESKPVI
D.melanogaster   ---KYGNDKPDTRFG------FLLNNVSEIIEK----S-DEFKEKYDDLGAYAIVVRASE
E.siliculosus    ---AVRMQYPST--------AVRVTEEPLVVHW---------------------------
G.gallus         ---DYGTDKPDTRFG------MKIVDISDTLRR--LDI-GFVQNVLTYPHGTAKAICIPQ
G.gorilla        ---TYGTDKPDTRFG------MKIIDISDVFRN--TEI-GFLQDALSKPHGTVKAICIPE
H.sapiens        ---TYGTDKPDTRFG------MKIIDISDVFRN--TEI-GFLQDALSKPHGTVKAICIPE
I.tridecemlinea  ---TYGTDKPDTRFG------MKIVDISNVFRH--TEV-RFLQDALSKPQGAVKAMCVRE
M.mulatta        ---TYGTDKPDTRFG------MKIIDISDVFRN--TEI-GFLQDALSKPHGTVKAICIPE
M.gallopavo      ---DYGTDKPDTRFG------MKIVDISDTLRR--VDI-GFVQNVLSYPHSTAKAICIPQ
M.musculus       ---TYGTDKPDTRFG------MKIVDVSDVFRN--TEL-RFLQDALAKPQGTVKAICVHD
O.sativa         ---RYGTDRPDLRFD------WQLKDVSDAFLGS--SF-KIFADTLE-NGGVIKALCVPG
O.latipes        ---DYGVDKPDTRFN------MKLTDISDIFSS--TQV-EFLRSALDQPEGSIQAILVPC
P.troglodytes    ---TYGTDKPDTRFG------MKIIDISDVFRN--TEI-GFLQDALSKPHGTVKAICIPE
P.falciparum     ---TIKNQYPSD--------DFVWLDKTPIFTY---------------------------
P.abelii         ------------------------------------------------------------
S.cerevisiae     ---SYGIDKPDLRAP-----DLKIINLGEFNA---------F-SHLNKKFPVFEVI----
S.pombe          ---KYGSDKPDIRF------ELPLKNITSLLP---------K-Q---DPLISTEIL----
S.purpuratus     ---QYGVDKPDTRFD------MKIQDVTDILQG--SGV-AMFDSSS--PSFRVKLINCKG
S.scrofa         LSKTLIISKRATALHRCNAGDISIVDISDMFRR--TEI-GYLQDALSKPQGTIKAMCVPE
T.rubripes       ---DYGVDKPDTRFA------MKLIDLSDIFMS--TDV-PFLRSALSQPGGYVQAICVPS
X.tropicalis     ---SYGVDKPDTRFE------MKIQDVTSQFRE--VQL-GYVQETLRKPHGCVKAICIPG
                                                                             


                       8290      8300      8310      8320      8330      8340
                 =========+=========+=========+=========+=========+=========+
A.carolinensis   G-----A---------------KYLHDKDLSRLN---EM-TKFQFNQV------LLPLI-
A.gambiae        A-----RKLP------------TGLKKSLEEIAK---QS-NFCKFTQS------------
A.thaliana       G-----A---------------KKYSNSALKKGD---IYNEAMKSGAK---G--LPFLK-
B.distachyon     G-----A---------------KVFSNTDLKKGT---VYAEASKAGAK---G--LPFLK-
C.elegans        A-----G-K-------------TTISNSMKNEWK---RL-IQMNENGK------------
C.jacchus        G-----A---------------KYFKRKDIESIR---KF-SADHFDQE------ILPVF-
C.neoformans     A-----Q-------------------AAGLDIPS---IAG--SNSF--------IDYVK-
P.tetraurelia    ------------------------------------------------------------
D.rerio          G-----T---------------KHLKGKDFEFLK---QA-AKTQYGQE------VSVVP-
D.discoideum     KCIKLDQVL-------------PTLKSKHLDQIT---TE-----SN------S-IITVQ-
D.melanogaster   A-----FW-N------------GAARKHYESLGK---EF-KGTLFVRK------------
E.siliculosus    ----------------------------------------------------------E-
G.gallus         G-----A---------------RYLKHKDLESLK---EA-AKSRFNQE------IMDVI-
G.gorilla        G-----A---------------KYLKRKDIESIR---KF-AADHFNQE------ILPVF-
H.sapiens        G-----A---------------KYLKRKDIESIR---NF-AADHFNQE------ILPVF-
I.tridecemlinea  G-----A---------------NYLKKKDIESIR---KF-AADHFNEE------VLPVF-
M.mulatta        G-----A---------------KYLKRKDIESIR---KF-AADHFNQE------ILPVF-
M.gallopavo      G-----V---------------RYLKNKDLESLK---EA-AKSQFNQE------IMDVI-
M.musculus       G-----A---------------KYLRKEDIEFIR---KF-AVHHFSQE------VLPIF-
O.sativa         G-----A---------------KEFSNTDLKKGT---VYTEASKAGAK---G--LPFLK-
O.latipes        G-----A---------------NLFTGRELETIK---QT-ANTQFGQE------LSLVL-
P.troglodytes    G-----A---------------KYLKRKDIESIR---KF-AADHFNQE------ILPVF-
P.falciparum     ----------------------------------------------------------E-
P.abelii         ------------------------------------------------------------
S.cerevisiae     -ILR------------------SAFSNMEEYKERWSFLT-NNSNYNYR---VPIVLPIEN
S.pombe          -VYNDLS---------------HSLSNAESRKLC--------EAVGEN---V-VVTSIRE
S.purpuratus     A-----A---------------NHLKRKHIDHLE---AE-GKKKATQLGMEKGGVIGVR-
S.scrofa         G-----AHICFENSLIRGFPKINTLKGKNLESLR---KF-AAEHFNQE------VLPVF-
T.rubripes       G-----V---------------KHLSGNHLNKLK---EM-AMTQFGQE------LSLVQ-
X.tropicalis     G-----A---------------KFIKRKELDSMQ---EL-VKQQYNQE------VVPVI-
                                                                             


                       8350      8360      8370      8380      8390      8400
                 =========+=========+=========+=========+=========+=========+
A.carolinensis   -LR--PD-VRLKSRLAKFLN-ESKKLEL---LKAV--QANVDDVVVLAAGEQE--KVC--
A.gambiae        --K--VTPEWSSGPIQRLLG-KESADAL---SEQL--SLQPDDLLLLGWGKQT--YVQ--
A.thaliana       -VLDNGE-IEGIAALVSSLD-SAGKINF---VKQC--GAAPGDLILFGVGPVT--SVN--
B.distachyon     -VMDNGE-LEGIGPLVSTLK-PEKKEQL---LELL--DAKAGDLILFALGEQS--TAN--
C.elegans        ------NFAICHPSQKRWFK-PFDNQKL---IDQF--GLIDEDVLIVCWGNSE--GVY--
C.jacchus        -LN--TN-RNWNSPVANFIM-ELKRQEL---IRLM--ETQEEDVVLLTAGEHN--KAC--
C.neoformans     -ITNANA-HSWLG--ESVLT-ASLGLSL---DKSLPGGVNPGDVVWLSRRKKI--AEGGW
P.tetraurelia    ------------------------------------------------------------
D.rerio          -VR--AD-GSLKSPLSRLLS-DTAKQQL---LQMA--QANAGDLIFITAGPRE--NVR--
D.discoideum     -IK--SN-NEWKSLISKSIS-EQEKTLI---TERM--NLKEGDVLLISVGP--RFQVES-
D.melanogaster   --F--GPTKDVQEKLGKLLG-EDVATEV---ADKF--DLEENDLLFLGIGSKV--ETR--
E.siliculosus    -----------------------DGIQMLRD---A--GHEAND--FDDLSG----AQEL-
G.gallus         -CR--PD-GSLKSLLTKFLG-EEQQSEL---IRVL--NMQVDDVVLLAVGDHK--RVC--
G.gorilla        -LN--AN-RNWNSPVANFIM-ESQRLEL---IRLM--ETQEEDVVLLTAGEHN--KAC--
H.sapiens        -LN--AN-RNWNSPVANFIM-ESQRLEL---IRLM--ETQEEDVVLLTAGEHN--KAC--
I.tridecemlinea  -LK--NK-ENWNSPVAKFIL-EKQRLEL---IRLM--EIQEDDVVLLTAGEHK--KAC--
M.mulatta        -LN--AN-RNWNSPVASFIM-ESQRLEL---IRLM--DTQEEDVVLLTAGEHN--KAC--
M.gallopavo      -CR--PD-GSLKSLLTKFLS-EEQQSEL---IQAL--NMQVDDVVLLAAGDHE--RVC--
M.musculus       -LN--AK-KNWSSPFAKFIM-EEERLEL---ARSM--EIQEEDIVLLTAGEHE--KAC--
O.sativa         -VMDNGE-LEGIGPLVASLK-PEKKEQL---LKHL--DAKSGDLILFALGEQS--AAN--
O.latipes        -VR--TD-GTLKSPLKKLLS-ASVTDDL---IRKT--QAQPGDLLLIAAGTLH--SVR--
P.troglodytes    -LN--AN-RNWNSPVANFIM-ESQRLEL---IRLM--ETQEEDVVLLTAGEHN--KAC--
P.falciparum     -----------------------EAIKILIKNGKL--FLKEEDILTYDLTT----DLEK-
P.abelii         ------------------------------------------------------------
S.cerevisiae     DEQ--AN-SNWFENFHAIAT-FENPHLI---TKFL--KLKKGDIVCGCTREPNHSIFENP
S.pombe          HSQ--L--QTWVKKLPQLRQLPIVAEEL---NQKL--QIGINSIVFMTNRP--KYLVSGT
S.purpuratus     -LE--ASPASWKSPIAKYIE-DDVKERL---VKSL--GAETGDLFFIAAGE-K--DIN--
S.scrofa         -LK--AN-RNWNSLLAKFIT-EEQGLGL---IRLL--GIQEEDVVLVTAGEHK--KAC--
T.rubripes       -VR--EN-GTLNSPLKKLIS-PKTTAEL---LQRT--GSRPGDLLLIAAGSLH--TVR--
X.tropicalis     -LR--AD-SSWKSPLEKFLS-AKLKESL---TEAM--EAKSEDILLFAAGEHI--RAC--
                                                                             


                       8410      8420      8430      8440      8450      8460
                 =========+=========+=========+=========+=========+=========+
A.carolinensis   LTLGKVRLEAAELLEARG-LPLRKPSAFHFLWVVDFPLFLQKEESP-----S--------
A.gambiae        NLMGRIRLATYEALEERNLAPKRQSHAHNFLWVVDFPMFSENEETG--------------
A.thaliana       KTLDRLRLFVAHDMDLI------DHSKHSILWVTDFPMFEWNEPE------Q--------
B.distachyon     QILGRLRLFIAHKLEVI------DTSAHSILWVTDFPMFEWNSDE------Q--------
C.elegans        WTL-----------EVCG-L--RSKSNVTAHWIVDFPLFSFEEG----------------
C.jacchus        SLLGKLRLECADLLETRG-VVLRDPTLFSFLWVVDFPLFLPKEENP-----R--------
C.neoformans     THLGRLRVQLMETLVAKGLMT--LPTQPHFLWITQFPLFTLADKDKIHLSRG--------
P.tetraurelia    ------------------------------------------------------------
D.rerio          PLLGKLRLQVAELLERYG-VPVRDPSVFHFLWVVDFPLFLPKEDDP-----E--------
D.discoideum     -TLGKTRIYCANLLKELN-LLKLDPQQFNFLWVVDFPLFTPSDYMN--------------
D.melanogaster   ELLGRIRLDYQDFLVENA--KIKKPNDFRFLWVIDFPLFERNRETN--------------
E.siliculosus    -ALGEL---------------VAEKFGSDFFFLDRFPT----------------------
G.gallus         SALGSLRLESADLLEAAG-MVLRDPAALHFLWVVDFPLFLPKEENP-----S--------
G.gorilla        SLLGKLRLECADLLETRG-VVLRDPTLFSFLWVVDFPLFLPKEENP-----R--------
H.sapiens        SLLGKLRLECADLLETRG-VVLRDPTLFSFLWVVDFPLFLPKEENP-----R--------
I.tridecemlinea  SLLGKLRLACADILEMNG-MVLRDPALFSFLWVVDFPLFLPKEENP-----M--------
M.mulatta        SLLGKLRLECADLLETRG-MVLRDPTLFSFLWVVDFPLFLPKEENP-----R--------
M.gallopavo      SALGSLRLESADLLEAAG-MVLRDPAALHFLWVVDFPLFLPKKENP-----S--------
M.musculus       SLLGKLRLECADLLEMRG-AVLRDPAVFSFLWVVDFPLFLAKEESP-----T--------
O.sativa         RILGRLRLFIAHKLEVI------DTSAHSILWVTDFPMFEWNSDE------Q--------
O.latipes        PLLGSLRLQCAELLESHG-RSVRDPLAFHFLWVVDFPLFLPKEDEP-----Q--------
P.troglodytes    SLLGKLRLECADLLETRG-VVLRDPTLFSFLWVVDFPLFLPKEENP-----R--------
P.falciparum     -ELGKL---------------IKLSHNTDYYIIINFPS----------------------
P.abelii         ------------------------------------------------------------
S.cerevisiae     TPLGRLRQLVLQSEHGKNIYHAVNKDVAS--WIVDFPLFSPVIIED-----KSGKKEKLA
S.pombe          TPLGKLRLLLHELLVKKKALPELDKDLLKFVWVVDFPLFSPTEE----------------
S.purpuratus     QILGHVRLQAAKRLEENG-VSVRDPSSFNFLWVEDFPLFLPR------------------
S.scrofa         SLLGKLRLECADLLEARG-VVLRDPALFSFLWVVDFPLFFPKEEHP-----Q--------
T.rubripes       TLLGHLRLQSAELLECCG-VSVRDPSVFHFLWVVDFPLFLTSEHEP-----E--------
X.tropicalis     SALGKLRLDAAAQLEEYG-VPLRDPTAFHFLWVVDFPLFLPKEDNE-----L--------
                                                                             


                       8470      8480      8490      8500      8510      8520
                 =========+=========+=========+=========+=========+=========+
A.carolinensis   -------ELESAHHPF-TAPHPSDEHLLYTEP------SKVRGQHYDLVLNGSEIGGGSI
A.gambiae        -------QIESTHHPF-TAPHPEDAAALSDGCT--ENIYKIRSLAYDLVWNGVEIGGGSI
A.thaliana       -------RLEALHHPF-TAPKPEDMDDLP----------SARALAYDMVYNGVEIGGGSL
B.distachyon     -------RYEALHHPF-TAPNPEDMNDLP----------SARALAYDMIYNGVEIGGGSL
C.elegans        -------QLVSTHHPF-TAPLEKDIDILYSNDI--DKLLQITGQHYDLVINGVEMGGGSI
C.jacchus        -------ELESAHHPF-TAPHPSDIHLLYTEP------KKVRSQHYDLVLNGNEIGGGSI
C.neoformans     -------RYASTHHPF-TAPMYEDLADLKAGK-----VDSVRGQHYDLVLDGQEIGGGSV
P.tetraurelia    ------------------------------------------------------------
D.rerio          -------RLESAHHPF-TAPVPEDAHLLYTRP------HMVRGQHYDLVLNGCEIGGGSI
D.discoideum     ----EQSALLSTHHPF-TAPHPEDIDLLLNPLSTPSDYSKIRGQHYDIVINGVELGGGSI
D.melanogaster   -------QLESVHHPF-TLPHSDDLENFATSC---ENLESIRSQAYDLVLNGQEVGGGSI
E.siliculosus    -----------QIRPFYTMPCPDD---------------KRYSNSYDLILRGQEICSGAQ
G.gallus         -------ELESAHHPF-TAPHPLDTDLLYSDP------TKVRSQHYDLVLNGSEIGGGSI
G.gorilla        -------ELESAHHPF-TAPHPSDIHLLYTEP------KKARSQHYDLVLNGNEIGGGSI
H.sapiens        -------ELESAHHPF-TAPHPSDIHLLYTEP------KKARSQHYDLVLNGNEIGGGSI
I.tridecemlinea  -------ELESAHHPF-TAPHPGDIHLLYTEP------EKVRSQHYDLVLNGSEIGGGSI
M.mulatta        -------ELESAHHPF-TAPHPSDIHLLYTEP------KKARSQHYDLVLNGNEIGGGSI
M.gallopavo      -------ELESAHHPF-TAPHPLDTNLLYSDP------TKVRSQHYDLVLNGSEIGGGSI
M.musculus       -------ELESAHHPF-TAPNSSDIHLLYTEP------EKVRGQHYDLVLNGNEIGGGSV
O.sativa         -------RYEALHHPF-TAPNPEDMNDLP----------SARALAYDMIYNGVEIGGGSL
O.latipes        -------ELESAHHPF-TAPLPEDTALLFTEP------HKVRGQHYDLVLNGCEIGGGSI
P.troglodytes    -------ELESAHHPF-TAPHPSDIHLLYTEP------KKARSQHYDLVLNGNEIGGGSI
P.falciparum     -----------SLRPFYTMYKEDD---------------PKISNSYDFFMRGEEILSGSQ
P.abelii         ------------------------------------------------------------
S.cerevisiae     YPEYEKDRLCSTHHPF-TMVKLKDYEKLEKTP------EKCLGRHYDLVVNGVELGGGST
S.pombe          ----KNQSITSTHHPF-TAPHWDDVHLLEKKP------LSVRGLHYDIVVNGIELGGGSI
S.purpuratus     -----------------------------TRS------RRVRGQHYDLVLNGIEIAGGSI
S.scrofa         -------ELESAHHPF-TAPHPSDIHLLYTEP------QKVRSQHYDLVLNGNEVGGGSI
T.rubripes       -------NLESAHHPF-TAPLPEDTHLLYTEP------QKVRGQHYDLVLNGCEVGGGSI
X.tropicalis     -------ELESAHHPF-TAPHPEDTALLHTDP------AKVRSQHYDLVLNGSEVGGGSI
                                                                             


                       8530      8540      8550      8560      8570      8580
                 =========+=========+=========+=========+=========+=========+
A.carolinensis   RIHNAKQQCFVLENVLKEDTK---PLAHLLEALDSGAPPHGGIALGLDRLICLIVGA--E
A.gambiae        RIHNSQLQRTVLKDVLKIEHA---HLHHLLDALESGAPPHGGFAIGLDRYVALLCNA--P
A.thaliana       RIYKRDVQEKVLEIIGISPEEAESKFGYLLEALDMGAPPHGGIAYGLDRMVMMLGGA--S
B.distachyon     RIYKSDVQQRIFEIIGISPEQAEEKFGYLLECFDMGAPPHGGIAYGLDRLVMLLACE--S
C.elegans        RIENSEIQRHVLKV-LGEPTD---EMEHLLNALSHGAPPHGGFALGLDRFVAMLTSDGNP
C.jacchus        RIHNAELQQYILATLLKEDVK---LLSHMLQALDYGAPPHGGIALGLDRLICVVTGS--P
C.neoformans     RIHDARLQEWVMKEVLQLDEQEMGRFDHLLRALKCGAPPHGGLALGFDRLVAILCGA--K
P.tetraurelia    ------------------------------------------------------------
D.rerio          RIHNASQQLYILDTILKEDPS---LLSHLLEALDSGAPPHGGIALGLDRLVSIIVGA--P
D.discoideum     RIHNSDVQLRVLEKVLKLEPHMVQRFNHLLTALSMGCPPHGGIALGFDRLCSLLVNS--N
D.melanogaster   RIHDRDMQHFILEQILKIPHD---HLSHLLSALESGCPPHGGIALGLDRLIAILCRA--R
E.siliculosus    RCHDADMLMQILEEK-GVPAE---PLSDYINAFRHGCPPHGGGGVGLERIVFLYLGL--D
G.gallus         RIHNAEQQRFVLEKVLKEDSE---VLSHLLEALEFGAPPHGGIALGLDRLISLIVGS--P
G.gorilla        RIHNAELQRYILATLLKEDVK---MLSHLLQALDYGAPPHGGIALGLDRLICLVTGS--P
H.sapiens        RIHNAELQRYILATLLKEDVK---MLSHLLQALDYGAPPHGGIALGLDRLICLVTGS--P
I.tridecemlinea  RIHDAELQHYILGTLLKEDVK---LLSHLLQALDYGAPPHGGIALGLDRLMCLITGA--A
M.mulatta        RIHNAELQRYILATLLKEDVK---MLSHLLQALDYGAPPHGGIALGLDRLICLVTGS--P
M.gallopavo      RIHNAEQQRFVLEKVLKEDSE---VLSHLLEALEFGAPPHGGIALGLDRLISLIVGS--P
M.musculus       RIHDAQLQRYILETLLKEDVK---LLSHLLQALDYGAPPHGGIALGLDRLVCLVTGA--P
O.sativa         RIYKSDVQQRIFEIIGISPEQAEEKFGYLLECFDMGAPPHGGIAYGLDRLVMLLAGE--N
O.latipes        RIHRASEQLYVLKNILKEDPS---QLTHLLEALDFGAPPHGGIALGLDRLVSIIVGA--P
P.troglodytes    RIHNAELQRYILATLLKEDVK---MLSHLLQALDYGAPPHGGIALGLDRLICLVTGS--R
P.falciparum     RISDMKLLLENIKLF-NLDPN---KLNFYIDSFAYSSYPHSGCGIGLERVLMLFLGL--N
P.abelii         ------------------------------------------------------------
S.cerevisiae     RIHDPRLQDYIFEDILKIDNAYEL-FGHLLNAFDMGTPPHAGFAIGFDRMCAMICET--E
S.pombe          RIHNPDIQRFVLKDVLKLPENRYATFEHLIRVLSSGCPPHGGIALGFDRLAALLTNA--P
S.purpuratus     RIHRADLQRYILEDVLKEDSS---NLSHLLEALESGCPPHGGIAIGFDRYISLLCGE--P
S.scrofa         RIHNAELQRYELPFEFQEDVK---LLSHLLQALDYGAPPHGGIALGLDRLMCLVTGA--P
T.rubripes       RIHKASEQRYVLKNILKEDPT---LLTHLLEALDSGAPPHGGIALGLDRLLSIMVAS--Q
X.tropicalis     RIHNSNLQRWVLQDVLKEDVS---LLSHLLEALQSGAPPHGGIALGLDRLIAIIVGA--P
                                                                             


                       8590      8600      8610      8620      8630      8640
                 =========+=========+=========+=========+=========+=========+
A.carolinensis   --SIRDVIAFPKSFKGRDLMSNAPDDVSPEELKPYHIQVSWPSA----------------
A.gambiae        --SIRDVIAFPKSADGKDPLSKAPVPISEEEKRIYHIQVVE-------------------
A.thaliana       --SIRDVIAFPKTTTAQCALTRTPSEVDPKQLQDLSIRTK--------------------
B.distachyon     --SIRDVIAFPKTTTAQCSLTKAPSPVDPQQLKDLGLHAP--------------------
C.elegans        LTPVRDVIAFPKTKNGKDLMSDAPATLSQKQLERYGISLLPNAVE---------------
C.jacchus        --SIRDVIAFPKSFRGHDLMSNTPDSIPPEELKPYHIQVSWPTD----------------
C.neoformans     --SIRDVIAFPKSTTGQDPVFKSPSVSGNEVLKEYGLQSLERDE----------------
P.tetraurelia    ------------------------------------------------------------
D.rerio          --SIRDVIAFPKSFRGHDLMSQAPDFVSEKDLKPYHISVVWPIA----------------
D.discoideum     --SIRDVIAFPKTSGGKELMTSSPATVTKSELDELFLIQ---------------------
D.melanogaster   --SIRDVIAFPKSLNGRDPLSNAPVPISDEEMRLYHLSVVDEEEPSTNNTSHEQEDDDPD
E.siliculosus    --NIRKASMFPRDPSRCSP-----------------------------------------
G.gallus         --SIRDVIAFPKSFKGRDLMGNAPDYVTPEELEPYHIHVSWPAA----------------
G.gorilla        --SIRDVIAFPKSFRGHDLMSNTPDSVPPEELKPYHIRVSRPTD----------------
H.sapiens        --SIRDVIAFPKSFRGHDLMSNTPDSVPPEELKPYHIRVSKPTD----------------
I.tridecemlinea  --SIRDVIAFPKSFRGHDLMSNAPDSVSPQELKPYHIQVSWPAD----------------
M.mulatta        --SIRDVIAFPKSFRGHDLMSNAPDSIPSEELKPYHIRVSWPTD----------------
M.gallopavo      --SIRDVIAFPKSFRGRDLMGNAPDYVTPEELEPYHIHVSWPAA----------------
M.musculus       --SIRDVIAFPKSYRGQDLMSNAPDSVSPEELKPYHIHVLWPAD----------------
O.sativa         --SIRDVIAFPKTTTAQCSLTKAPSPVDPQQLKDLGFRT---------------------
O.latipes        --SIRDVIAFPKSFRGHDLMSRAPDTVSDKDLTPYHICVKWPTK----------------
P.troglodytes    --SIRDVIAFPKSFRGHDLMSNTPDSVPPEELKPYHIRVSKPTD----------------
P.falciparum     --NIRKTSLFPRDPKRLIP-----------------------------------------
P.abelii         ------------------------------------------------------------
S.cerevisiae     --SIRDVIAFPKSITGADLVVKSPSVIPESILEPYNIKYSNSK-----------------
S.pombe          --GIREVIAFPKTSSGADLLIGSPSAIPEEMLKDYNVAITRQTQ----------------
S.purpuratus     --SIRDVIAFPKTVDGNDLMSGAPSTLSAQELERYNIHVAPQGK----------------
S.scrofa         --SIRDVIAFPKSFRGHDLMSNAPDSIPPEDLKPYHIQVSWPTD----------------
T.rubripes       --SIRDVIAFPKSFRGHDLMSSAPDLISEEELKSYHISVIWPM-----------------
X.tropicalis     --SIRDVIAFPKSFRGRDLMSNAPDMVSAEDLQQYHIQVIPPCT----------------
                                                                             


                       8650      8660      8670      8680      8690      8700
                 =========+=========+=========+=========+=========+=========+
A.carolinensis   -----------------------ETQ---K------------------------------
A.gambiae        ------------------------------------------------------------
A.thaliana       ------------------------------------------------------------
B.distachyon     ------------------------------------------------------------
C.elegans        ------------------------------------------------------------
C.jacchus        -----------------------SKAAKA-------------------------------
C.neoformans     --------------------------KI--------------------------------
P.tetraurelia    ------------------------------------------------------------
D.rerio          ----------------------------E-------------------------------
D.discoideum     -------------------------K----------------------------------
D.melanogaster   QERAPPSPMSATSESEQPQINVDIKQEPEGLNEDFAPESPATKSTVKADPDEPSPSNPLA
E.siliculosus    ------------------------------------------------------------
G.gallus         -----------------------ETEAKKN------------------------------
G.gorilla        -----------------------SKAERA-------------------------------
H.sapiens        -----------------------SKAERA-------------------------------
I.tridecemlinea  -----------------------SEAERSSLNHSHHPE----------------------
M.mulatta        -----------------------SKAERA-------------------------------
M.gallopavo      -----------------------ETEAKKN------------------------------
M.musculus       -----------------------SEEESASATPSKHLS----------------------
O.sativa         ------------------------------------------------------------
O.latipes        -----------------------LGGEEK-------------------------------
P.troglodytes    -----------------------SKAERA-------------------------------
P.falciparum     ------------------------------------------------------------
P.abelii         ------------------------------------------------------------
S.cerevisiae     -------------------------K----------------------------------
S.pombe          -----------------------NRN----------------------------------
S.purpuratus     -----------------------E------------------------------------
S.scrofa         -----------------------SAKES-SLSHSCHPE----------------------
T.rubripes       ----------------------------E-------------------------------
X.tropicalis     -----------------------ESSG---------------------------------
                                                                             


                       8710      8720      8730      8740      8750      8760
                 =========+=========+=========+=========+=========+=========+
A.carolinensis   ------------------------------------------------------------
A.gambiae        ------------------------------------------------------------
A.thaliana       -----------------MHLIFFFSYFLRRYLLLLCAILILRAPLAHSLIPPLTCVNTGT
B.distachyon     -----------------M------------------------------------------
C.elegans        ------------------------------------------------------------
C.jacchus        ----------------H-------------------------------------------
C.neoformans     ------------------------------------------------------------
P.tetraurelia    ------------------------------------------------------------
D.rerio          ------------------------------------------------------------
D.discoideum     -----------------MNHS---------------------------------------
D.melanogaster   SEAATPARARRTVKKKV-------------------------------------------
E.siliculosus    -----------------M------------------------------------------
G.gallus         ------------------------------------------------------------
G.gorilla        ----------------H-------------------------------------------
H.sapiens        ----------------H-------------------------------------------
I.tridecemlinea  ----------------S-------------------------------------------
M.mulatta        ----------------H-------------------------------------------
M.gallopavo      ------------------------------------------------------------
M.musculus       ----------------S-------------------------------------------
O.sativa         -----------------M------------------------------------------
O.latipes        ---------------------------------------MRSDPTTLRALIPEASQPSVP
P.troglodytes    ----------------H-------------------------------------------
P.falciparum     ------------------------------------------------------------
P.abelii         ------------------------------------------------------------
S.cerevisiae     -----------------MP-----------------------------------------
S.pombe          -----------------M------------------------------------------
S.purpuratus     ----------------S-------------------------------------------
S.scrofa         ----------------S-------------------------------------------
T.rubripes       ------------------------------------------------------------
X.tropicalis     ----------------K-------------------------------------------
                                                                             


                       8770      8780      8790      8800      8810      8820
                 =========+=========+=========+=========+=========+=========+
A.carolinensis   ----------------------MS----GPVPSRARVYTDVNTHRPREYWDYEA-H---E
A.gambiae        ----------------------------MTLPSSARVYADVNSHKPREYWDYEN-Y---I
A.thaliana       VESDVTGIRFDRCLDTDSLAKISL----STVMSKARVYTDVNVIRPKDYWDYES-L---N
B.distachyon     --------------------------------SKARVYSDVNVVRPKEYWDYEA-L---A
C.elegans        -----------------------M----PPIPSRARVYAEVNPSRPREYWDYEA-H---M
C.jacchus        ----------------------MS----GPVPSRARVYTDVNTHRPREYWDYES-H---V
C.neoformans     -----------------------M----SGGRSVARVYANVNEKLGRSWWDYDN-L---V
P.tetraurelia    ------------------------------------------------------------
D.rerio          ----------------------MS----GPVPSRSRVYPDVNTQRPREYWDYES-H---V
D.discoideum     ------------------SKKNKN----RILRNKARIYCDVNLHKPKEYWNYEA-L---N
D.melanogaster   ----------------------------MTLPSAARVYTDVNAHKPDEYWDYEN-Y---V
E.siliculosus    --------------TTRQRLPPSA----GEVRSVSRVYHSACLERGQSYWDYEN-FRLSS
G.gallus         ----------------------MS----GPVPSRARVYTDVNTHRPREYWDYES-H---V
G.gorilla        ----------------------MS----GPVPSRARVYTDVNTHRPREYWDYES-H---V
H.sapiens        ----------------------MS----GPVPSRARVYTDVNTHRPREYWDYES-H---V
I.tridecemlinea  ----------------------MS----GPVPSRARVYTDVNTHRPREYWDYES-H---V
M.mulatta        ----------------------MS----GPVPSRARVYTDVNTHRPREYWDYES-H---V
M.gallopavo      ----------------------MS----GPVPSRARVYTDVNTHRPREYWDYES-H---V
M.musculus       ----------------------MS----GPVPSRARVYTDVNTHRPREYWDYES-H---V
O.sativa         --------------------------------SKARVYTDVNVLRPKEYWDYEA-L---T
O.latipes        FHLTIYPGVNHPGDLSQEPVQNMS----GPVPSRSRVYPDVNTQRPREYWDYES-H---V
P.troglodytes    ----------------------------------------------------MC-V---S
P.falciparum     ----------------------MSVSSINKKIYIPKFYADVNIHKPKEYYDYDN-L---E
P.abelii         ----------------------MS----GPVPSRARVYTDVNTHRPREYWDYES-H---V
S.cerevisiae     --------------LPPSTLNQKS----NRVYSVARVYKNACEERPQEYWDYEQGV---T
S.pombe          -------------------NQTEA----APVVSVSRVYAHVNEEMPREYWDYEN-M---Q
S.purpuratus     ----------------------------MASMSRARVYADVNSHRPREYWDYES-H---V
S.scrofa         ----------------------MS----GPVPSRARVYTDVNTHRPREYWDYES-H---V
T.rubripes       ----------------------MS----GPVPSRARVYTEVNTHQPREYWDYES-H---V
X.tropicalis     ----------------------MV----GG------------------------------
                                                                             


                       8830      8840      8850      8860      8870      8880
                 =========+=========+=========+=========+=========+=========+
A.carolinensis   VEWGNQDDYQLVRKLGRGKYSEVFEAINITNNEKVVVKILKPVKKKKIRREIKILENLRG
A.gambiae        VDWVNQDDYQLVRKLGRGKYSEVFEAIKMTSNEKCVVKILKPVKKKKIKREIKILENLRG
A.thaliana       VQWGEQDDYEVVRKVGRGKYSEVFEGINMNNNEKCIIKILKPVKKKKIRREIKILQNLCG
B.distachyon     VQWGEQDDYEVVRKVGRGKYSEVFEGINVNNNEKCVIKILKPVKKKKIKREIKILQNLCG
C.elegans        IEWGQIDDYQLVRKLGRGKYSEVFEGFKMSTDEKVVVKILKPVKKKKIKREIKILENLRG
C.jacchus        VEWGNQDDYQLVRKLGRGKYSEVFEAINITNNEKVVVKILKPVKKKKIKREIKILENLRG
C.neoformans     VQWGVQDNYEIVRKVGRGKYSEVFESIHLPTDSKCIVKVLKPVKKKKIKREIKILQNLAG
P.tetraurelia    ------------------------------------------------------------
D.rerio          VDWGNQDDYQLVRKLGRGKYSEVFEAINITNNEKVVVKILKPVKKKKIKREIKILENLRG
D.discoideum     VKWETQDDYEIIRKIGRGKYSEVFEGANIKNNEKCVIKVLKPVKKKKIKREIKILQNLCG
D.melanogaster   VDWGNQDDYQLVRKLGRGKYSEVFEAINITTTEKCVVKILKPVKKKKIKREIKILENLRG
E.siliculosus    LGWEDVDRYEVNGRLGFGRFSEVMEGVEVDSGRKVVLKVLKPARTYKVKREIRVLQLLAG
G.gallus         VEWGNQDDYQLVRKLGRGKYSEVFEAINITNNEKVVVKILKPVKKKKIKREIKILENLRG
G.gorilla        VEWGNQDDYQLVRKLGRGKYSEVFEAINITNNEKVVVKILKPVKKKKIKREIKILENLRG
H.sapiens        VEWGNQDDYQLVRKLGRGKYSEVFEAINITNNEKVVVKILKPVKKKKIKREIKILENLRG
I.tridecemlinea  VEWGNQDDYQLVRKLGRGKYSEVFEAINITNNEKVVVKILKPVKKKKIKREIKILENLRG
M.mulatta        VEWGNQDDYQLVRKLGRGKYSEVFEAINITNNEKVVVKILKPVKKKKIKREIKILENLRG
M.gallopavo      VEWGNQDDYQLVRKLGRGKYSEVFEAINITNNEKVVVKILKPVKKKKIKREIKILENLRG
M.musculus       VEWGNQDDYQLVRKLGRGKYSEVFEAINITNNEKVVVKILKPVKKKKIKREIKILENLRG
O.sativa         VQWGEQDDYEVVRKVGRGKYSEVFEGINVNNNEKCIIKILKPVKKKKIKREIKILQNLCG
O.latipes        VEWGNQDDYQLVRKLGRGKYSEVFEAINITNNEKVVVKILKPVKKKKIKREIKILENLRG
P.troglodytes    SFYSNQDDYQLVRKLGRGKYSEVFEAINITNNERVVVKILKPVKKKKIKREVKILENLRG
P.falciparum     LQWNKPNRYEIMKKIGRGKYSEVFNGYDTECNRPCAIKVLKPVKKKKIKREIKILQNLNG
P.abelii         VEWGNQDDYQLVRKLGRGKYSEVFEAINITNNEKVVVKILKPVKKKKIKREIKILENSRG
S.cerevisiae     IDWGKISNYEIINKIGRGKYSEVFSGRCIVNNQKCVIKVLKPVKMKKIYRELKILTNLTG
S.pombe          EVFGYQDNYEIIRKVGRGKYSEVFEGLNVLNNSKCIIKVLKPVKYKKIKREIKILQNLAG
S.purpuratus     VEWGQQDDYQIVRKLGRGKYSEVFEAVNITSNEKCVIKILKPVKKKKIKREIKILENLRG
S.scrofa         VEWGNQDDYQLVRKLGRGKYSEVFEAINITNNEKVVVKILKPVKKKKIKREIKILENLRG
T.rubripes       VEWGNQDDFQLVRKLGRGKYSEVFEAINITSNEKVVVKILKPVKKKKIKREIKILENLRG
X.tropicalis     ----------HIRAFCSG-LSPFFPLL------------LQPVKKKKIKREIKILENLRG
                                                                             


                       8890      8900      8910      8920      8930      8940
                 =========+=========+=========+=========+=========+=========+
A.carolinensis   GPNIITLADIVKDPVSRTPALVFEHVNN--------------------------------
A.gambiae        GTNIITLLAVVKDPVSRTPALIFEHVNN--------------------------------
A.thaliana       GPNIVKLLDVVRDQHSKTPSLIFEYVNS--------------------------------
B.distachyon     GPNIVKLLDIVRDQHSKTPSLIFEYINN--------------------------------
C.elegans        GTNIITLLDVVKDPISRTPALIFEHVNN--------------------------------
C.jacchus        GPNIITLADIVKDPVSRTPALVFEHVNN--------------------------------
C.neoformans     GPNVVGLLDVVRDSQSKTPSIVTEYVNN--------------------------------
P.tetraurelia    ------------------------------------------------------------
D.rerio          GPNIITLLDIIKDPVSRTPALVFEHVNN--------------------------------
D.discoideum     GPNIITLYDVVRDPQSKTPSLIFEYINN--------------------------------
D.melanogaster   GTNIITLLAVVKDPVSRTPALIFEHVNN--------------------------------
E.siliculosus    GPNVLSLEGVCRDRHTGVTTLILEHLGDGVQWFGHTTASSSLPGTAGGVRTAGSPNAVDA
G.gallus         GPNIITLADIVKDPVSRTPALVFEHVNN--------------------------------
G.gorilla        GPNIITLADIVKDPVSRTPALVFEHVNN--------------------------------
H.sapiens        GPNIITLADIVKDPVSRTPALVFEHVNN--------------------------------
I.tridecemlinea  GPNIITLADIVKDPVSRTPALVFEHVNN--------------------------------
M.mulatta        GPNIITLADIVKDPVSRTPALVFEHVNN--------------------------------
M.gallopavo      GPNIITLADIVKDPVSRTPALVFEHVNN--------------------------------
M.musculus       GPNIITLADIVKDPVSRTPALVFEHVNN--------------------------------
O.sativa         GPNIVKLLDIVRDQHSKTPSLIFEYVNN--------------------------------
O.latipes        GPNIISLLDIVKDPVSRTPALVFEHVNN--------------------------------
P.troglodytes    GTNIIKLIDTVKDPVSKTPALVFEYINN--------------------------------
P.falciparum     GPNIIKLLDIVKDPVTKTPSLIFEYINN--------------------------------
P.abelii         GPNIITLADIVKDPVSRTPALVFEHVNN--------------------------------
S.cerevisiae     GPNVVGLYDIVQDADSKIPALIFEEIKN--------------------------------
S.pombe          GPNIISLLDIVRDPESKTPSLIFEFVDN--------------------------------
S.purpuratus     GPNIIALQAIVKDPVSRTPALIFEYVNN--------------------------------
S.scrofa         GPNIITLADIVKDPVSRTPALVFEHVNN--------------------------------
T.rubripes       GPNIISLIDIVKDPVSRTPALVFEHVNN--------------------------------
X.tropicalis     GPNIITLADIVKDPVSRTPALVFEHVNN--------------------------------
                                                                             


                       8950      8960      8970      8980      8990      9000
                 =========+=========+=========+=========+=========+=========+
A.carolinensis   ------------------------------------------------------------
A.gambiae        ------------------------------------------------------------
A.thaliana       ------------------------------------------------------------
B.distachyon     ------------------------------------------------------------
C.elegans        ------------------------------------------------------------
C.jacchus        ------------------------------------------------------------
C.neoformans     ------------------------------------------------------------
P.tetraurelia    ------------------------------------------------------------
D.rerio          ------------------------------------------------------------
D.discoideum     ------------------------------------------------------------
D.melanogaster   ------------------------------------------------------------
E.siliculosus    SPKEVRPPPSRGREAGAADGGGAGATASSIGQGRALPWRGASSSFGTATSRATSADRREG
G.gallus         ------------------------------------------------------------
G.gorilla        ------------------------------------------------------------
H.sapiens        ------------------------------------------------------------
I.tridecemlinea  ------------------------------------------------------------
M.mulatta        ------------------------------------------------------------
M.gallopavo      ------------------------------------------------------------
M.musculus       ------------------------------------------------------------
O.sativa         ------------------------------------------------------------
O.latipes        ------------------------------------------------------------
P.troglodytes    ------------------------------------------------------------
P.falciparum     ------------------------------------------------------------
P.abelii         ------------------------------------------------------------
S.cerevisiae     ------------------------------------------------------------
S.pombe          ------------------------------------------------------------
S.purpuratus     ------------------------------------------------------------
S.scrofa         ------------------------------------------------------------
T.rubripes       ------------------------------------------------------------
X.tropicalis     ------------------------------------------------------------
                                                                             


                       9010      9020      9030      9040      9050      9060
                 =========+=========+=========+=========+=========+=========+
A.carolinensis   ----------------------------------------------------TDFKQLY-
A.gambiae        ----------------------------------------------------TDFKQLY-
A.thaliana       ----------------------------------------------------TDFKVLY-
B.distachyon     ----------------------------------------------------TDFKVLY-
C.elegans        ----------------------------------------------------SDFKQLY-
C.jacchus        ----------------------------------------------------TDFKQLY-
C.neoformans     ----------------------------------------------------TEFKTLY-
P.tetraurelia    ------------------------------------------------------------
D.rerio          ----------------------------------------------------TDFKQLY-
D.discoideum     ----------------------------------------------------TDFKHLS-
D.melanogaster   ----------------------------------------------------TDFKQLY-
E.siliculosus    LEEGRLLSRAGGQQQQPVAAGQDPAGEHGGGAARAEGLHDSEGGGGGRGGEATEKEAVDP
G.gallus         ----------------------------------------------------TDFKQLY-
G.gorilla        ----------------------------------------------------TDFKQLY-
H.sapiens        ----------------------------------------------------TDFKQLY-
I.tridecemlinea  ----------------------------------------------------TDFKQLY-
M.mulatta        ----------------------------------------------------TDFKQLY-
M.gallopavo      ----------------------------------------------------TDFKQLY-
M.musculus       ----------------------------------------------------TDFKQLY-
O.sativa         ----------------------------------------------------TDFKVLY-
O.latipes        ----------------------------------------------------TDFKQLY-
P.troglodytes    ----------------------------------------------------TDFKQLY-
P.falciparum     ----------------------------------------------------IDFKTLY-
P.abelii         ----------------------------------------------------TDFEQLY-
S.cerevisiae     ----------------------------------------------------VDFRTLY-
S.pombe          ----------------------------------------------------IDFRTLY-
S.purpuratus     ----------------------------------------------------TDFKQLY-
S.scrofa         ----------------------------------------------------TDFKQLY-
T.rubripes       ----------------------------------------------------TDFKQLY-
X.tropicalis     ----------------------------------------------------TDFKQLY-
                                                                             


                       9070      9080      9090      9100      9110      9120
                 =========+=========+=========+=========+=========+=========+
A.carolinensis   QTLTDYDIRFYMYEILKALDYCHSMGIMHRDVKPHNVMIDHEHRKLRLIDWGLAEFYHPG
A.gambiae        QTLSDYDIRYYLYELLKALDYCHSLGIMHRDVKPHNVMIDHENRKLRLIDWGLAEFYHPG
A.thaliana       PTLTDYDIRYYIYELLKALDFCHSQGIMHRDVKPHNVMIDHELRKLRLIDWGLAEFYHPG
B.distachyon     PTLTDYDIRYYIYELLKALDYCHSQGIMHRDVKPHNVMIDHELRKLRLIDWGLAEFYHPG
C.elegans        QTLSDYDIRYYLYELLKALDFCHSQGIMHRDVKPHNVMIDAEKRELRLIDWGLAEFYHPR
C.jacchus        QTLTDYDIRFYMYEILKALDYCHSMGIMHRDVKPHNVMIDHEHRKLRLIDWGLAEFYHPG
C.neoformans     PKFSDFDVRYYIFELLKALDFCHSKGIMHRDVKPHNVMIDHEKRTLRLIDWGLAEFYHPG
P.tetraurelia    ------------------------------------------------------------
D.rerio          QTLSDYDIRFYMYEILKALDYCHSMGIMHRDVKPHNVMIDHEHRKLRLIDWGLAEFYHPN
D.discoideum     PTLTDFDVRYYIRELLHALDFCHSNGIMHRDVKPSNVMIDHQKRKLYLIDWGLAEFYHPN
D.melanogaster   QTLTDYEIRYYLFELLKALDYCHSMGIMHRDVKPHNVMIDHENRKLRLIDWGLAEFYHPG
E.siliculosus    GRLTDYEVRLYLYKLLQALDFAHSRGLMHRDVKPRNIVINRRTRSLRLIDWGLGDFYIPG
G.gallus         QTLTDYDIRFYMYEILKALDYCHSMGIMHRDVKPHNVMIDHEHRKLRLIDWGLAEFYHPG
G.gorilla        QTLTDYDIRFYMYEILKALDYCHSMGIMHRDVKPHNVMIDHEHRKLRLIDWGLAEFYHPG
H.sapiens        QTLTDYDIRFYMYEILKALDYCHSMGIMHRDVKPHNVMIDHEHRKLRLIDWGLAEFYHPG
I.tridecemlinea  QTLTDYDIRFYMYEILKALDYCHSMGIMHRDVKPHNVMIDHEHRKLRLIDWGLAEFYHPG
M.mulatta        QTLTDYDIRFYMYEILKALDYCHSMGIMHRDVKPHNVMIDHEHRKLRLIDWGLAEFYHPG
M.gallopavo      QTLTDYDIRFYMYEILKALDYCHSMGIMHRDVKPHNVMIDHEHRKLRLIDWGLAEFYHPG
M.musculus       QTLTDYDIRFYMYEILKALDYCHSMGIMHRDVKPHNVMIDHEHRKLRLIDWGLAEFYHPG
O.sativa         PTLTDYDIRYYIYELLKALDYCHSQGIMHRDVKPHNVMIDHELRKLRLIDWGLAEFYHPG
O.latipes        QTLSDFDIRFYMYEILKALDYCHSMGIMHRDVKPHNVMIDHEHRKLRLIDWGLAEFYHPN
P.troglodytes    QILTDFDIRFYMYELLKALDYCHSKGIMHRDVKPHNVMIDHQQKKLRLIDWGLAEFYHPA
P.falciparum     PKFTDKDIRYYIYQILKALDYCHSQGIMHRDVKPHNIMIDHENRQIRLIDWGLAEFYHPG
P.abelii         QTLTDYDVRFYMYEILKALDYCHSMGIMHRDVKPHNVMIDHEHRKLRLIDWGLAEFYHPG
S.cerevisiae     PTFKLPDIQYYFTQLLIALDYCHSMGIMHRDVKPQNVMIDPTERKLRLIDWGLAEFYHPG
S.pombe          PTLSDYDIRYYSYELLKALDFCHSRGIMHRDVKPHNVMIDHKKRKLRLIDWGLAEFYHAG
S.purpuratus     QTLKDYEIRYYLFELLKALDYSHSMGIMHRDVKPHNVMIDHENRRLRLIDWGLAEFYHPG
S.scrofa         QTLTDYDIRFYMYEILKALDYCHSMGIMHRDVKPHNVMIDHEHRKLRLIDWGLAEFYHPG
T.rubripes       QTLTDFDIRFYMYEILKALDYCHSMGIMHRDVKPHNVMIDHEHRKLRLIDWGLAEFYHPG
X.tropicalis     QTLTDYDIRFYMYEILKALDYCHSMGIMHRDVKPHNVMIDHEHRKLRLIDWGLAEFYHPG
                                                                             


                       9130      9140      9150      9160      9170      9180
                 =========+=========+=========+=========+=========+=========+
A.carolinensis   QEYNVRVASRYFKGPELLVDYQMYDYSLDMWSLGCMLASMIFRKEPFFHGHDNYDQLVRI
A.gambiae        QEYNVRVASRYFKGPELLVDYQMYDYSLDMWSLGCMLASMIFRKEPFFHGHDNYDQLVRI
A.thaliana       KEYNVRVASRYFKGPELLVDLQDYDYSLDMWSLGCMFAGMIFRKEPFFYGHDNQDQLVKI
B.distachyon     KEYNVRVASRYFKGPELLVDLQDYDYSLDMWSLGCMFAGMIFRKEPFFYGHDNHDQLVKI
C.elegans        QDYNVRVASRYFKGPELLVDYQCYDYSLDMWSLGCMLASMIFRKEPFFHGHDNYDQLVRI
C.jacchus        QEYNVRVASRYFKGPELLVDYQMYDYSLDMWSLGCMLASMIFRKEPFFHGHDNYDQLVRI
C.neoformans     TEYNVRVASRYFKGPELLVDFQEYDYSLDMWSLGCMFASMIFRKEPFFHGHDNADQLVKI
P.tetraurelia    ------------------------------------------------------------
D.rerio          QEYNVRVASRYFKGPELLVDYQMYDYSLDMWSLGCMLASMIFRKEPFFHGHDNYDQLVRI
D.discoideum     QDYNVRVASRPYKGPELLVDMEDYDYSLDMWSLGCMFAGMLFQKDPFFHGHDNIDQLVKI
D.melanogaster   QEYNVRVASRYFKGPELLVDYQMYDYSLDMWSLGCMLASMIFRKEPFFHGHDNYDQLVRI
E.siliculosus    RRNMARVGSRYYKAPELLVGFRFYDYAVDIFSVGCMLAGFLLNREPFFRGKDNEDQLVRI
G.gallus         QEYNVRVASRYFKGPELLVDYQMYDYSLDMWSLGCMLASMIFRKEPFFHGHDNYDQLVRI
G.gorilla        QEYNVRVASRYFKGPELLVDYQMYDYSLDMWSLGCMLASMIFRKEPFFHGHDNYDQLVRI
H.sapiens        QEYNVRVASRYFKGPELLVDYQMYDYSLDMWSLGCMLASMIFRKEPFFHGHDNYDQLVRI
I.tridecemlinea  QEYNVRVASRYFKGPELLVDYQMYDYSLDMWSLGCMLASMIFRKEPFFHGHDNYDQLVRI
M.mulatta        QEYNVRVASRYFKGPELLVDYQMYDYSLDMWSLGCMLASMIFRKEPFFHGHDNYDQLVRI
M.gallopavo      QEYNVRVASRYFKGPELLVDYQMYDYSLDMWSLGCMLASMIFRKEPFFHGHDNYDQLVRI
M.musculus       QEYNVRVASRYFKGPELLVDYQMYDYSLDMWSLGCMLASMIFRKEPFFHGHDNYDQLVRI
O.sativa         KEYNVRVASRYFKGPELLVDLQDYDYSLDMWSLGCMFAGMIFRKEPFFYGHDNHDQLVKI
O.latipes        QEYNVRVASRYFKGPELLVDYQMYDYSLDMWSLGCMLASMIFRKEPFFHGHDNYDQLVRI
P.troglodytes    QEYNVRVASRYFKGPELLVDYQMYDYSLDMWSLGCMLASMIFRREPFFHGQDNYDQLVRI
P.falciparum     QEYNVRVASRYYKGPELLIDLQLYDYSLDIWSLGCMLAGMIFKKEPFFCGHDNYDQLVKI
P.abelii         REYNVRVASRYFKGPELLVDYQMYDYSLDMWSLGCMLASMIFRKEPFFHGHDNYDQLVRI
S.cerevisiae     VDYNVRVASRYHKGPELLVNLNQYDYSLDLWSVGCMLAAIVFKKEPFFKGSSNPDQLVKI
S.pombe          MEYNVRVASRYFKGPELLVDFREYDYSLDIWSFGVMFAALIFKKDTFFRGRDNYDQLVKI
S.purpuratus     QEYNVRVASRYFKGPELLVDYQLYDYSLDMWSLGCMLASMIFRKEPFFHGHDNYDQLVRI
S.scrofa         QEYNVRVASRYFKGPELLVDYQMYDYSLDMWSLGCMLASMIFRKEPFFHGHDNYDQLVRI
T.rubripes       QEYNVRVASRYFKGPELLVDYQMYDYSLDMWSLGCMLASMIFRKEPFFHGHDNYDQLVRI
X.tropicalis     QEYNVRVASRYFKGPELLVDYQMYDYSLDMWSLGCMLASMIFRKEPFFHGHDNYDQLVRI
                                                                             


                       9190      9200      9210      9220      9230      9240
                 =========+=========+=========+=========+=========+=========+
A.carolinensis   AKVLGTEDLYDYIDKYNIELDPRFNDILGRHSRKRWERFVHG---ENQQLVSPEALDFLD
A.gambiae        AKVLGTEDLFAYLDKYNIELDPRFNDILSRHSRKRWERFVHS---ENQHLVSPEGLDFLD
A.thaliana       AKVLGTDELNAYLNKYQLELDTQLEALVGRHSRKPWSKFINA---DNRHLVSPEAIDYLD
B.distachyon     AKVLGTDGLNTYLNKYRIELDPQLEALVGRHSRKPWSKFINA---DNQHLVSPEAIDFLD
C.elegans        AKVLGTDELYEYIARYHIDLDPRFNDILGRHSRKRWERFIHA---ENQHLVTPEALDFLD
C.jacchus        AKVLGTEDLYDYIDKYNIELDPRFNDILGRHSRKRWERFVHS---ENQHLVSPEALDFLD
C.neoformans     AKVLGTDELYTYLERYDIDLDAQFDDILGRYPRKPWSRFVSS---ENQRYISSEAIDFLD
P.tetraurelia    ------------------------------------------------------------
D.rerio          AKVLGTEDLYDYIDKYNIELDPRFNDILGRHSRKRWERFVHS---ENQHLVSTEALDFLD
D.discoideum     VKILGTEEFYAYLDKYGIVVDHTILSIIGKHPKKPWSRFITK---ENQHLAVPEAIDFLE
D.melanogaster   AKVLGTEELYAYLDKYNIDLDPRFHDILQRHSRKRWERFVHS---DNQHLVSPEALDFLD
E.siliculosus    ALVLGTQGLHDFLRKYDVVLEPRLLEMLGTHRKKPWSSIVGGGGGGCGEAYGPDGLDLLD
G.gallus         AKVLGTEDLYDYIDKYNIELDPRFNDILGRHSRKRWERFVHS---ENQHLVSPEALDFLD
G.gorilla        AKVLGTEDLYDYIDKYNIELDPRFNDILGRHSRKRWERFVHS---ENQHLVSPEALDFLD
H.sapiens        AKVLGTEDLYDYIDKYNIELDPRFNDILGRHSRKRWERFVHS---ENQHLVSPEALDFLD
I.tridecemlinea  AKVLGTEDLYDYIDKYNIELDPRFNDILGRHSRKRWERFVHS---ENQHLVSPEALDFLD
M.mulatta        AKVLGTEDLYDYIDKYNIELDPRFNDILGRHSRKRWERFVHS---ENQHLVSPEALDFLD
M.gallopavo      AKVLGTEDLYDYIDKYNIELDPRFNDILGRHSRKRWERFVHS---ENQHLVSPEALDFLD
M.musculus       AKVLGTEDLYDYIDKYNIELDPRFNDILGRHSRKRWERFVHS---ENQHLVSPEALDFLD
O.sativa         AKVLGTDSLNSYLNKYRIELDPQLEALVGRHSRKPWSKFINA---DNQHLVSPEAIDFLD
O.latipes        AKVLGTEDLYDYIDKYNIELDPRFNDILGRHSRKRWERFVHS---ENQHLVSTEALDFLD
P.troglodytes    AKVLGTEELYGYLKKYHIDLDPHFNDILGQHSRKRWENFIHS---ENRHLVSPEALDLLD
P.falciparum     AKVLGTEDLHAYLKKYNIKLKPHYLNILGEYERKPWSHFLTQ---SNIDIAKDEVIDLID
P.abelii         AKVLGTEDLYDYIDKYNIELDPRFNDILGRHSRKRWERFVHS---ENQHLVSPEALDFLD
S.cerevisiae     ATVLGTKELLGYLGKYGLHLPSEYDNIMRDFTKKSWTHFITS---ETK-LAVPEVVDLID
S.pombe          AKVLGTDELFAYVQKYQIVLDRQYDNILGQYPKRDWYSFVNR---DNRSLANDEAIDLLN
S.purpuratus     AKVLGTDELYEYLDKYQIELDPRFNDILGRHSRKRWERFVHS---ENQHLVSPEALDFLD
S.scrofa         AKVLGTEDLYDYIDKYNIELDPRFNDILGRHSRKRWERFVHS---ENQHLVSPEALDFLD
T.rubripes       AKVLGTEDLYDYIDKYNIELEPRFNDILGRHSRKRWERFVHS---DNQHLVSPEALDFLD
X.tropicalis     AKVLGTEDLYDYIDKYNIELDPRFNDILGRHSRKRWERFVHS---ENQHLVSPEALDFLD
                                                                             


                       9250      9260      9270      9280      9290      9300
                 =========+=========+=========+=========+=========+=========+
A.carolinensis   KLLRYDHQSRLTAREAMEHPYFYPIVKDQVRMG-SASMPSSSTPVSSSNMLSG-------
A.gambiae        KLLRYDHFERLTAREAMEHPYFAIIVNGQMPP------------PPTSSAKGG-------
A.thaliana       KLLRYDHQDRLTAKEAMAHPYFAQVRAAESSRMR-------------T------------
B.distachyon     KLLRYDHQDRLTAREAMAHPYFLQVRAAENSRTR-------------A------------
C.elegans        KLLRYDHAERLTAQEAMGHEYFRPVVEAHARANGTEQADGQGASNSASS-QSS-------
C.jacchus        KLLRYDHQSRLTAREAMEHPYFYTVVKDQARMG-SSSMAGGSTPVSSANMMSG-------
C.neoformans     KLLRYDHQERLTAEEAKGHPYFEPVRQAAAQAS---------------------------
P.tetraurelia    ------------------------------------------------------------
D.rerio          KLLRYDHQARLTAREAMDHSYFYPIVKDQGRGAPAAGMAASSTPVSSSSLMAG-------
D.discoideum     KLLRYDPAERLTTREAMEHPYFKPLS----------------------------------
D.melanogaster   KLLRYDHVDRLTAREAMAHPYFLPIVNGQMNP------------NNQ-------------
E.siliculosus    RMLCYDHQDRISAAEALSHAFFDPVRGEG-------------------------------
G.gallus         KLLRYDHQSRLTAREAMEHPYFYPIVKDQARMG-SSNMPGGSTPVSSASMMSG-------
G.gorilla        KLLRYDHQSRLTAREAMEHPYFYTVVKDQARMG-SSSMPGGSTPVSSANMMSG-------
H.sapiens        KLLRYDHQSRLTAREAMEHPYFYTVVKDQARMG-SSSMPGGSTPVSSANMMSG-------
I.tridecemlinea  KLLRYDHQSRLTAREAMEHPYFYTVVKDQARMG-SSNMPGGSTPVSSANMMSG-------
M.mulatta        KLLRYDHQSRLTAREAMEHPYFYTVVKDQARMG-SSSMPGGSTPVSSANMMSG-------
M.gallopavo      KLLRYDHQSRLTAREAMEHPYFYPIVKDQARMG-SSNMPGGSTPVSSASMMSG-------
M.musculus       KLLRYDHQSRLTAREAMEHPYFYTVVKDQARMS-STSMAGGSTPVSSANMMSG-------
O.sativa         KLLRYDHQDRLTAREAMAHPYFLQVRAAENSRPR-------------A------------
O.latipes        KLLRYDHQARLTAREAMDHPYFFPIVKDQGRGATPGGMAASSTPVSSSSMMAG-------
P.troglodytes    KLLRYDHQQRLTAKEAMEHPYFYPVVKEQS------------QPCADNAVLSS-------
P.falciparum     KMLIYDHAKRIAPKEAMEHPYFREVREE--------------------------------
P.abelii         KLLRYDHQSRLTAREAMEHPYFYTVVKDQARMG-SSSMPGGSTPVSSANMMSG-------
S.cerevisiae     NLLRYDHQERLTAKEAMDHKFFKTKF----------------------------------
S.pombe          RLLRYDHQERLTCQEAMAHPYFQVLK----------------------------------
S.purpuratus     KLLRYDHQERVTAREAMEHPYFYPVVKEQNRSMPPVMSTQPNTSTSATN--SS-------
S.scrofa         KLLRYDHQSRLTAREAMEHPYFYTVVKDQARMG-SSSMPGGSTPVSSANMMSG-------
T.rubripes       KLLRYDHQIRLTAREAMDHPYFFPIMKDQSRVTVSASLVGGNTAVSTTSMITG-------
X.tropicalis     KLLRYDHQTRLTAREAMDHPYFYPIVKDQSRMG-GSNMPSGSTPVSSASMMSGQSRSSVR
                                                                             


                       9310      9320      9330      9340      9350      9360
                 =========+=========+=========+=========+=========+=========+
A.carolinensis   ----ISSVP-T-SSPLGPLAGS-P-----VIAAANPLGMPVPAAAGAQQ-----------
A.gambiae        -----------------------------------------------TNLHSPHR-----
A.thaliana       ------------------------------------------------Q-----------
B.distachyon     ------------------------------------------------Q-----------
[truncated: 92,686 more chars]
